# Indirect Anti‐Markovnikov Hydrofunctionalization of Terminal Alkenes via an Alkenyl Thianthrenium Intermediate

**DOI:** 10.1002/anie.202424422

**Published:** 2025-04-10

**Authors:** Bence Sóvári, Péter Angyal, István J. Babcsányi, András M. Kotschy, Ádám Dudás, Gábor Turczel, Szilárd Varga, Tibor Soós

**Affiliations:** ^1^ Organocatalysis Research Group Institute of Organic Chemistry HUN‐REN Research Centre for Natural Sciences 2 Magyar tudósok krt. Budapest H‐1117 Hungary; ^2^ Hevesy György PhD School of Chemistry Eötvös Loránd University 1/a Pázmány Péter sétány Budapest H‐1117 Hungary; ^3^ Centre for Structural Science HUN‐REN Research Centre for Natural Sciences 2 Magyar tudósok krt. Budapest H‐1117 Hungary

**Keywords:** Anti‐Markovnikov hydrofunctionalization, Isotopic labeling, Thianthrenation, Umpolung, Unactivated olefins

## Abstract

The anti‐Markovnikov hydrofunctionalization of terminal, unactivated olefins is an evergreen synthetic challenge in organic chemistry. Several direct and indirect anti‐Markovnikov methods have been developed, ranging from the classical hydroboration/oxidation protocol to state‐of‐the‐art photoredox catalytic, transition‐metal‐complex‐catalyzed, and enzymatic procedures. Despite the ever‐expanding suite of synthetic capabilities, these methods still have limited generality in their substrate scope, especially with nucleophiles. Herein, we show that terminal, unactivated olefins can be transformed into anti‐Markovnikov products via an alkenyl thianthrenium intermediate that undergoes sequential hydride and nucleophile addition. The strategic advantage of this method lies in the ability to utilize a diverse array of oxidatively sensitive nucleophiles as reaction partners. This is accomplished through a mechanistically distinct, two‐stage dication pool anti‐Markovnikov approach, where separate oxidative olefin activation by thianthrenation is followed by the selective generation of a reactive alkyl thianthrenium salt.

## Graphical abstract

A two‐stage dication pool strategy using alkenyl thianthrenium intermediates has been developed for anti‐Markovnikov hydrofunctionalization. This method differs from previous approaches in its reaction mechanism and addresses their most significant limitation, namely, the restricted generality of the nucleophile. The approach has been extended to other challenging transformations, including anti‐Wacker‐type oxidation, hydroacetylenation, hydrogenation, and deuterium labeling.

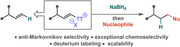

## Introduction

**Figure 1:**
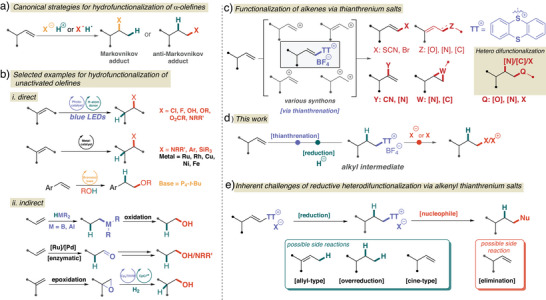
Previous and current reports on olefin hydrofunctionalization. a) Markovnikov and anti‐Markovnikov hydrofunctionalization of alkenes. b) State‐of‐the‐art methods. c) Functionalization of unactivated alkenes via thianthrenium salts. d) The alternative anti‐Markovnikov hydrofunctionalization of unactivated olefins via alkenyl thianthrenium salts. e) Inherent challenges in the reductive heterodifunctionalization of alkenyl thianthrenium salts.

Olefin hydrofunctionalization,^[^ ^1^, ^2^, ^3^, ^4^ ^]^ the addition of X─H (X─H = HO─H, RO─H, RS─H, aliphatic/aromatic amines, N─H azoles, etc.) across carbon‐carbon double bonds, is a fundamental and routinely applied strategy in organic synthesis, largely fueled by easy access to abundant and diverse olefin feedstocks (Figure 1a). While appealing in principle, they are often avoided methods in multistep synthesis because of problems with chemoselectivity and the harsh reaction conditions of Markovnikov‐type additions,^[^ ^5^ ^]^ not to mention the inherent challenge of accessing the anti‐Markovnikov series of addition products.^[^ ^6^, ^7^ ^]^ An obvious approach to alleviating synthetic constraints is to develop mechanistically divergent methods, such as Mukaiyama hydration under Markovnikov‐type addition,^[^ ^8^, ^9^ ^]^ which proceeds via lower‐energy carbon‐centered radicals rather than the high‐energy carbocations generated by strong Brønsted acids in the classical methods.

The importance of the anti‐Markovnikov functionalization of alkenes goes far beyond reversing the regioselectivity of a classical route, as it also provides a means to access essential commodities and fine chemicals for industrial, especially pharmaceutical, applications. For direct functionalization, current developments (Figure 1b) mainly utilize radical reactions, including photoredox transformations^[^ ^10^, ^11^, ^12^, ^13^, ^14^, ^15^, ^16^, ^17^, ^18^, ^19^, ^20^, ^21^, ^22^, ^23^, ^24^, ^25^, ^26^ ^]^ and transition metal‐based catalytic systems,^[^ ^25^, ^26^, ^27^, ^28^, ^29^, ^30^, ^31^ ^]^ to achieve the desired regiocontrol. Nevertheless, these innovative methodologies remain constrained in their scope due to radical stability concerns, the restricted generality of nucleophiles, or the reliance on toxic precious metal catalysts. Ionic anti‐Markovnikov addition to alkenes without transition metal catalysis remains a significant challenge, and only scattered examples have been reported by Bandar using Brønsted base catalysis; however, this methodology is confined to styrene derivatives.^[^ ^32^ ^]^

Despite the many advances in direct anti‐Markovnikov functionalization, there is still a lack of generality of scope under practical conditions, leading to the use and development of various indirect or formal approaches (Figure 1b). For example, the hydroalumination/oxidation sequence of Ziegler^[^ ^33^ ^]^ (≈ million‐ton scale annually) or the hydroboration/oxidation sequence of Brown and Zweifel^[^ ^34^ ^]^ are still the most popular protocols for obtaining hydration products with anti‐Markovnikov regioselectivity. Nevertheless, to address the challenges posed by the generation of nonrecyclable waste and safety concerns associated with these venerable reactions, some modern alternative oxidation/reduction methods have emerged, including Grubbs’^[^ ^35^ ^]^ and Feringa's^[^ ^36^ ^]^ approaches via anti‐Wacker products, Arnold's^[^ ^37^ ^]^ biocatalytic oxidation methodology, and regioselective epoxide hydrogenation by Gansäuer and Norton.^[^ ^38^ ^]^ While these indirect/formal methodologies are extensively exploited, the need to broaden the applicability of these approaches remains.

As any established anti‐Markovnikov functionalization strategy requires substantial compromises in scope and practicality, we instigated to develop a mechanistically divergent strategy, assuming that it can significantly broaden the reach of applicability. Specifically, we envisioned that the prior transformation of an unactivated olefin to a dielectrophile would provide a unique opportunity for reductive heterodifunctionalization, such that sequential hydride and nucleophile introduction would lead to anti‐Markovnikov products. This dication pool strategy^[^ ^39^ ^]^ has the benefit of separating olefin activation from nucleophile introduction, allowing not only the direct use of nucleophiles without any masking but also the use of oxidatively sensitive nucleophiles. However, adopting a dicationic‐‘hydride‐Nu’ strategy for anti‐Markovnikov reductive heterodifunctionalization is a formidable challenge, as such a strategy is absent from the literature. In addition, not only a simple dication pool, but also a dication pool approach with a two‐stage regioselective functionalization option is needed to unlock such an appealing new synthetic platform. While alkenes can be readily converted to vicinal dielectrophiles (e.g., iodonium, selenium intermediates, or dihalides), these dicationic synthons do not provide the mechanistic profile^[^ ^40^, ^41^, ^42^ ^]^ to enable the envisioned synthetic platform.

Thus, we were drawn to address this challenge by adapting the chemistry of alkenyl thianthrenium salts as a conceptually distinct approach for unactivated olefin functionalization. From α‐olefins, the corresponding alkenyl thianthrenium salts are readily accessible in a regio‐ and stereoselective manner^[^ ^43^ ^]^ and have shown diverse and unique reactivities as transient umpolung synthons (Figure 1c).^[^ ^44^, ^45^, ^46^ ^]^ Thus, recent findings have illuminated that alkenyl thianthrenium salts can be used as vinyl cationic (either ipso or cine),^[^ ^43^, ^47^, ^48^ ^]^ dicationic,^[^ ^47^, ^49^, ^50^, ^51^, ^52^ ^]^ and allyl cationic^[^ ^52^, ^53^, ^54^ ^]^ electrophiles depending on the reactants, conditions, and substrates. In addition, recent notable advancements by Wickens,^[^ ^55^ ^]^ Cozzi and Gualandi,^[^ ^56^ ^]^ Choi and Chung,^[^ ^57^ ^]^ and Saha^[^ ^58^ ^]^ indicate that the dication pool strategy can be translated into heterodifunctionalization reactions enabling the formation of various diamines, lactones, dihalides, and ether products. However, in previous examples, alkenyl thianthrenium salts have consistently undergone reactions under redox‐neutral or oxidative conditions, and their reductive transformations have yet to be explored and further investigated. Herein, our alternative thianthrenation‐based reductive heterodifunctionalization of terminal olefins is reported (Figure 1d), which proceeds via exclusive anti‐Markovnikov regioselectivity and can overcome many of the challenges of anti‐Markovnikov functionalization. This metal‐free, indirect, two‐stage dication pool strategy utilizes alkenyl thianthrenium salt intermediates and has been shown to be successful in the sequential, regio‐, and chemoselective addition of a hydride and the corresponding nucleophile to provide anti‐Markovnikov products.

## Results and Discussion

At the outset of the development, several challenges were apparent, particularly reactivity and selectivity concerns, that needed to be addressed in order to develop an anti‐Markovnikov functionalization based on thianthrenation (Figure 1e). For example, recent advances in thianthrenium chemistry have shown that a dicationic approach for olefin heterodifunctionalization can only be used for a limited range of nucleophiles to minimize or avoid the parallel sequential addition of the same nucleophiles and potential allylic functionalization.^[^ ^55^, ^56^, ^57^, ^58^ ^]^ Accordingly, the realization of the anti‐Markovnikov methodology outlined above would require the development of the partial reduction of alkenyl thianthrenium salts to alkyl thianthrenium salts, which introduces regio‐ and chemoselectivity issues, all of which need to be controlled. Therefore, our primary focus was to identify a suitable reduction method that would yield an alkyl thianthrenium salt from an alkenyl derivative without significant overreduction. Therefore, terminal olefin **1** was thianthrenated to provide model compound **2** for this critical partial reduction study (Table 1). Heterogeneous catalytic hydrogenation (entry 2) and hydrogen atom transfer (HAT) based methods^[^ ^8^ ^]^ (entry 3) were unsuccessful, only some overreduction to **4** was observed, confirming the challenge of partial reduction. We also screened various hydride reagents and were pleased to find that sodium borohydride gave the expected alkyl thianthrenium adduct **3** (entry 1). Importantly, conducting the reaction in technical grade MeCN solvent resulted in the formation of hydroxylated side product **5**, indicating the high sensitivity of alkyl thianthrenium species to competing nucleophilic attack. Furthermore, the yield can be significantly improved by the use of an inorganic proton source additive (entries 1 and 7 vs entry 8) to intercept the formed reactive sulfonium ylide intermediate, which is consistent with Wickens’^[^ ^55^ ^]^ observations during their heterodifunctionalization development. The sodium borohydride reagent clearly outperformed other borohydrides with suppressed reactivity (entries 4, 5) and borane complexes (entry 6). However, somewhat surprisingly, stronger reducing agents, such as LiAlH_4_, DIBAL‐H, L‐Selectride, and Superhydride also failed to provide the desired, partially reduced product **3** (for more details, see the SI). The fine balance between partial reduction and overreduction was clearly demonstrated by the effect of slight changes in the reaction conditions. The use of excess reagent (entries 1 and 9 vs 10) and longer reaction times (entries 1 and 11 vs 12) resulted in significant overreduction to alkane **4** (for more details, see the SI). It is also worth highlighting the practical aspects of the method developed, as the protocol bypasses the need for aqueous work‐up or column chromatography, providing higher yields and delivering products of excellent purity through only filtration and trituration. Finally, it is important to highlight that this process represents a viable alternative to the formation of alkyl thianthrenium salts from primary alcohols or their derivatives developed by Shine^[^ ^44^, ^59^, ^60^ ^]^ and Shi.^[^ ^61^, ^62^ ^]^

**Table 1:** Optimization of the partial reduction of alkenyl thianthrenium salts.

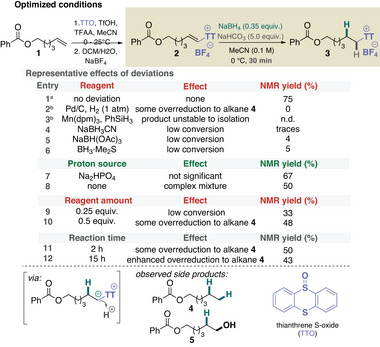

^a)^ Dried MeCN was used, otherwise, extensive formation of 5 was observed.

^b)^ No NaHCO_3_ additive was used.

**Figure 2:**
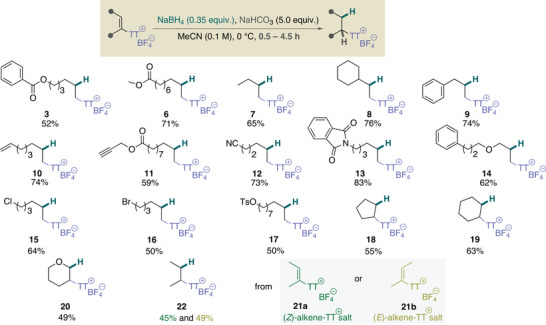
Scope of the partial reduction of alkenyl thianthrenium salts to alkyl thianthrenium salts.

With the optimized set of conditions in hand, we went on to explore the scope of the partial reduction of alkenyl thianthrenium salts and found that it was remarkably broad (Figure 2). First, we explored a range of alkyl, aryl, and unsaturated bond‐decorated terminal alkenes and found that all investigated alkenyl thianthrenium salts were viable for partial reduction to give the corresponding **7–10** alkyl thianthrenium salts. Next, we probed the partial reduction of substrates with reactive handles, such as ester, nitrile, or imide functionalities. Gratifyingly, owing to the mild reactivity of NaBH_4_, these substrates could also be accommodated, as exemplified by the synthesis of **3**, **6**, **11**–**13**. Furthermore, heteroatom connection at the allylic position was tolerated, as demonstrated by successful conversion to the ether derivative **14**. Then, partial reductions of alkenyl thianthrenes containing (pseudo)halogenide functionalities, such as chloride, bromide, and even tosylate, were explored. These substrates smoothly participated in the reaction without any side reactions. These (pseudo)halogenide alkyl thianthrenium salts **15**–**17** are particularly striking and open the door to accessing valuable bifunctional linkers frequently used in medicinal chemistry campaigns. Next, we sought to extend our methodology to internal olefins, which are still challenging substrates within the domain of alkenyl thianthrenium chemistry. The issue of site‐selective thianthrenation remains unresolved in the context of internal olefins, a problem that has been documented in previous publications. The only exception to this is 3,4‐dihydropyran, where full selectivity has been observed.^[^ ^43^ ^]^ To avoid selectivity issues, symmetric internal olefins and 3,4‐dihydropyran were probed in this reductive methodology. Although some decrease in the rate of these reactions was observed, cyclic olefins were still readily converted to the corresponding alkyl thianthrenium salts **18**, **19** in two steps. To our delight, even a less reactive cyclic, push–pull alkenyl thianthrenium salt was tolerated in the reduction step, resulting in the formation of tetrahydropyran derivative **20**, a challenging product that is chemically intractable from 3,4‐dihydropyran. Although limited in scope, **21a,b** acyclic *(E)* and *(Z)* olefins have also been studied in the thianthrenation‐partial reduction sequence. Although they are viable substrates in this reaction, the yields of partial reduction to **22** were found to be slightly lower than those of their cyclic counterparts.

**Figure 3:**
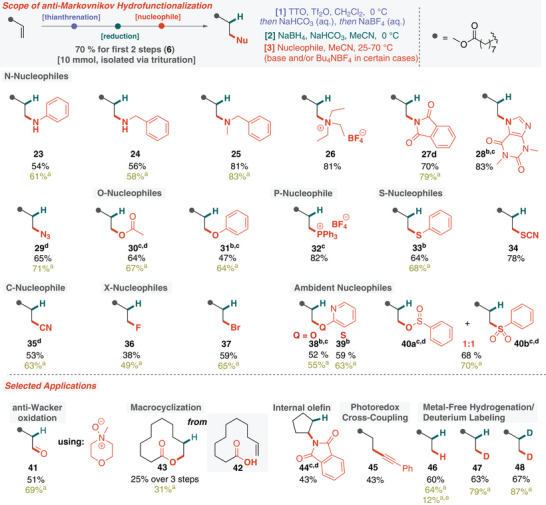
Scope of the anti‐Markovnikov hydrofunctionalization via alkyl thianthrenium salt **6** and applications in challenging transformations. Reaction conditions: alkyl thianthrenium salt **6** (0.41–0.70 mmol) in anhydrous acetonitrile (0.1 M), nucleophile (5.0 equiv), base (if indicated, 5.0 equiv), Bu_4_NBF_4_ (if indicated, 0.10 equiv) at 25–70 °C for 2.5–18 h. a) Yield determined by ^1^H NMR analysis of the crude reaction mixture. b) Additional base was used. c) The reaction was conducted at a higher temperature. d) Bu_4_NBF_4_ (10 mol%) was used. e) One‐pot reaction using electrogenerated alkenyl thianthrenium salt.

Since the second stage of the envisioned anti‐Markovnikov hydrofunctionalization platform is a nucleophilic substitution reaction, we investigated whether the formed alkyl thianthrenium salts could be substituted with a variety of nucleophiles without deleterious elimination side reactions (Figure 3). In a dual attempt to elucidate the reactivity of the alkyl thianthrenium salts in depth and to valorize a renewable feedstock, 9‐decenoic acid methyl ester (9‐DAME) was selected as the starting material.^[^ ^63^, ^64^ ^]^ The corresponding alkyl thianthrenium derivative **6** was easily synthesized on a gram scale, and excellent overall yield was obtained via only trituration as purification in both reaction steps. First, we probed various nitrogen nucleophiles, including primary, secondary, and tertiary amines, as well as phthalimide potassium salt, theophylline, and sodium azide, all yielding the desired anti‐Markovnikov products **23**–**29** in good yields. Notably, while the installation of the primary amine proved challenging owing to extensive dialkylation, it could be suppressed using excess nucleophile in the case of **24**. Importantly, no elimination side products were detected in these reactions, and no C8‐alkylated product was detected in the case of the alkylation of theophylline (**28**). This latter example may also be relevant to the toxicity of these thianthrene‐based alkylating agents and suggests that the mechanism of substitution is ionic rather than radical.^[^ ^65^, ^66^ ^]^ We then explored a variety of oxygen, phosphorus, and sulfur nucleophiles, all of which efficiently provided the envisioned anti‐Markovnikov products **30**–**34** without elimination side products. Next, we investigated carbon─carbon and carbon─halogenide bond formations, successfully utilizing nitrile (**35**), fluoride (**36**), and bromide (**37**) nucleophiles. To gain further insight into the reactivity of an alkyl thianthrenium intermediate, its reaction with ambident nucleophiles was examined. Somewhat surprisingly, the 2‐pyridone gave only the O‐alkylated product **38**, making this method an appealing alternative for the more difficult‐to‐access isomer.^[^ ^67^ ^]^ 2‐Mercaptopyridine also reacted selectively with **6** as an S‐nucleophile (**39**), whereas sodium benzenesulfinate led to a mixture of products **40a**,**b**. These experiments showed that Pearson's concept of hard and soft acids is not suitable for predicting and rationalizing the reactivity of alkyl thianthrenium salts toward ambident nucleophiles.^[^ ^68^ ^]^

Having demonstrated the versatility of our anti‐Markovnikov hydrofunctionalization with several common nucleophiles, we sought to push the boundaries of our platform further (Figure 3). First, using a suitable oxidizing nucleophile (*N*‐methylmorpholine‐*N*‐oxide), we successfully carried out a Kornblum/Ganem modification of our method to yield the corresponding aldehyde **41**. Notably, this variation represents a formal, metal‐free anti‐Wacker application^[^ ^69^ ^]^ which might have potential in aldehyde synthesis. Another advance was our demonstration of the feasibility of an intramolecular hydrofunctionalization reaction. For this, we carried out a three‐step reaction sequence starting from the inexpensive alkene **42**, which led to the macrocyclic Aldambre (**43**), an important fragrance with an intriguing note of hot iron. Next, we sought to demonstrate the feasibility of extending the partial reduction/nucleophilic substitution sequence to internal olefins. This effort was influenced by the systematic study of Shine et al.,^[^ ^70^ ^]^ who showed that the reactivity of these salts differs from that of the primary alkyl thianthrenium salts and that the elimination side reaction may be significant. Notwithstanding the anticipated elimination side reaction, it was found that the thianthrenation based hydrofunctionalization is also applicable for internal olefins. This finding is evidenced by the synthesis of **44** compounds. A further impressive feature of this hydrofunctionalization is that in the second stage, the alkyl thianthrenium salt can be further elaborated not only in ionic reactions but also in radical reactions. To illuminate this possibility, the acetylene derivative **45** was constructed in a mild photocatalytic reaction developed by Shi et al.^[^ ^61^ ^]^ Finally, the possibility of indirect olefin hydrogenation and deuteration was investigated. The alkenyl thianthrenium salt **6** was first prepared chemically or electrochemically, and various non‐deuterated (**46**), mono‐deuterated (**47**), and di‐deuterated (**48**) products were readily obtained by the judicious use of sodium borohydride or sodium deuteroborohydride. Accordingly, an intriguing metal‐free indirect hydrogenation methodology was established that allowed the saturation of unactivated alkenes using nucleophilic hydride sources, providing an unconventional, complementary and, in certain cases, cost‐effective alternative to hydrogenation and deuterium labeling.^[^ ^71^ ^]^

## Conclusion

We have developed a mechanistically distinct platform for the indirect, metal‐free anti‐Markovnikov hydrofunctionalization of unactivated alpha–olefins. The key to success was the regioselective thianthrenation of the olefinic bond, followed by chemoselective and partial reduction into readily transformable alkyl thianthrenium salts. A key advantage of this two‐stage dication pool strategy is that it allows a wide variety of nucleophiles to be accommodated in the anti‐Markovnikov reaction. Therefore, the method outlined herein not only differs from previous approaches in reaction mechanism but also addresses the most significant limitation of previous approaches, namely, the restricted generality of the nucleophiles. Furthermore, we revealed that this metal‐free olefin hydrofunctionalization platform can be expanded toward other challenging transformations, including anti‐Wacker‐type oxidation, macrocyclization, anti‐Markovnikov hydro‐acetylenation, hydrogenation, and deuterium labeling. Thus, the data presented here suggest that the advantages associated with this method far outweigh its indirect nature. This method can extend the diversification of an enormous range of commercially available nucleophiles (amines, esters, thiols) and alpha‐olefins. As such, this method is expected to greatly facilitate the expansion of chemical space and provide alternative planning options in synthesis.

## Supporting Information

The authors have cited additional references within the Supporting Information.^[^ ^72^, ^73^, ^74^ ^]^

## Conflict of Interests

B.S., P.A., A.M.K., I.J.B., Á.D., Sz.V., and T.S. are inventors in PCT/HU2025/050008 patent describing the use of this methodology to access anti‐Markovnikov products.

## Supporting information



Supporting Information

## Data Availability

The data that support the findings of this study are available from the corresponding author upon reasonable request.
