# Supplementary material for: Indirect Anti‐Markovnikov Hydrofunctionalization of Terminal Alkenes via an Alkenyl Thianthrenium Intermediate
Source: Angew Chem Int Ed Engl. 2025 Apr 10;64(21):e202424422. doi: 10.1002/anie.202424422 (PMC12087863; doi:10.1002/anie.202424422)
Supplement: Supplementary file 1 — Supporting Information [file ANIE-64-e202424422-s001.pdf]

1. General information S2
2. Preparation of alkenyl thianthren-5-ium tetrafluoroborate salts S3
    2.1. General procedure S4
    2.2. Specific procedure S7
3. Preparation of alkyl thianthren-5-ium tetrafluoroborate salts S9
    3.1. General procedure S9
    3.2. Detailed optimization and practical guidance S21
4. Reactions of alkyl thianthren-5-ium tetrafluoroborate salts S24
    4.1. General procedure S24
    4.2. Specific procedures S38
    4.3. Applications and one-pot modifications S42
5. NMR Spectra S45
6. References S118

1. General information

All reactions were carried out using oven-dried glassware and anhydrous solvents under an atmosphere of argon unless noted otherwise. All electrochemical oxidations were carried out using an IKA ElectraSyn 2.0 potentiostat (Ident. No. 0020008980, IKA), equipped with an IKA Pro-Divide divided cell (Ident. No. 0040006482, IKA) with a 10 µm frit, and standard IKA electrodes (RVC: Ident. No. 0040002860, IKA; Nickel foam: Ident. No. 0040002861, IKA). Flash silica chromatography was performed on silica gel (RediSep Gold Normal Phase Silica columns, 20-40 micron) with the indicated eluents. Thin-layer chromatography was performed on silica plates (Kieselgel 60 F_{254} Merck). Compounds were visualized by UV (254 nm) or Ceric Ammonium Molybdate (CAM) staining. HRMS spectra were obtained using Agilent 6545 LC/ Q-TOF (Agilent Technologies Inc. Santa Clara, CA USA), using electrospray ionization (ESI) or using JEOL/Agilent GC/MS JMS-T200GC/7890B using field ionization (FI). ^{1}H and ^{13}C NMR spectra were recorded using Varian NMR System spectrometers, operating at 400 and 500 MHz respectively. Chemical shifts are referenced to the residual solvent signals ($CDCl_3$: δ = 7.26 ppm for ^{1}H, δ = 77.0 ppm for ^{13}C, DMSO-d^6: δ = 2.50 ppm for ^{1}H, δ = 39.5 ppm for ^{13}C). ^{19}F NMR spectra were recorded using a Varian 300 MHz INOVA spectrometer. ^{19}F NMR spectra were referenced externally to benzotrifluoride at -63.72 ppm. Data are reported as follows: chemical shifts (ppm), multiplicity (s = singlet, d = doublet, t = triplet, q = quartet, br = broad, m = multiplet), coupling constants (Hz), and integration. All spectra were recorded with the standard spectrometer pulse sequences and settings. If indicated, low-temperature thermostat cooled reactions were carried out using a JULABO FT902 FT immersion cooler. All starting materials were purchased from Aldrich, TCI, or Fluorochem and used without further purification unless stated otherwise. Anhydrous tetrahydrofuran was distilled from sodium/benzophenone, while dichloromethane (stabilized with amylene) and acetonitrile from calcium hydride. In the case of chromatography, the percentages provided are expressed as V/V%.

2. Preparation of alkenyl thianthren-5-ium tetrafluoroborate salts

Alkenyl thianthren-5-ium salts shown below were reported by Ritter[43], Shu[52] and Soós[54]. Please see these references for characterization as well as graphical supporting information.

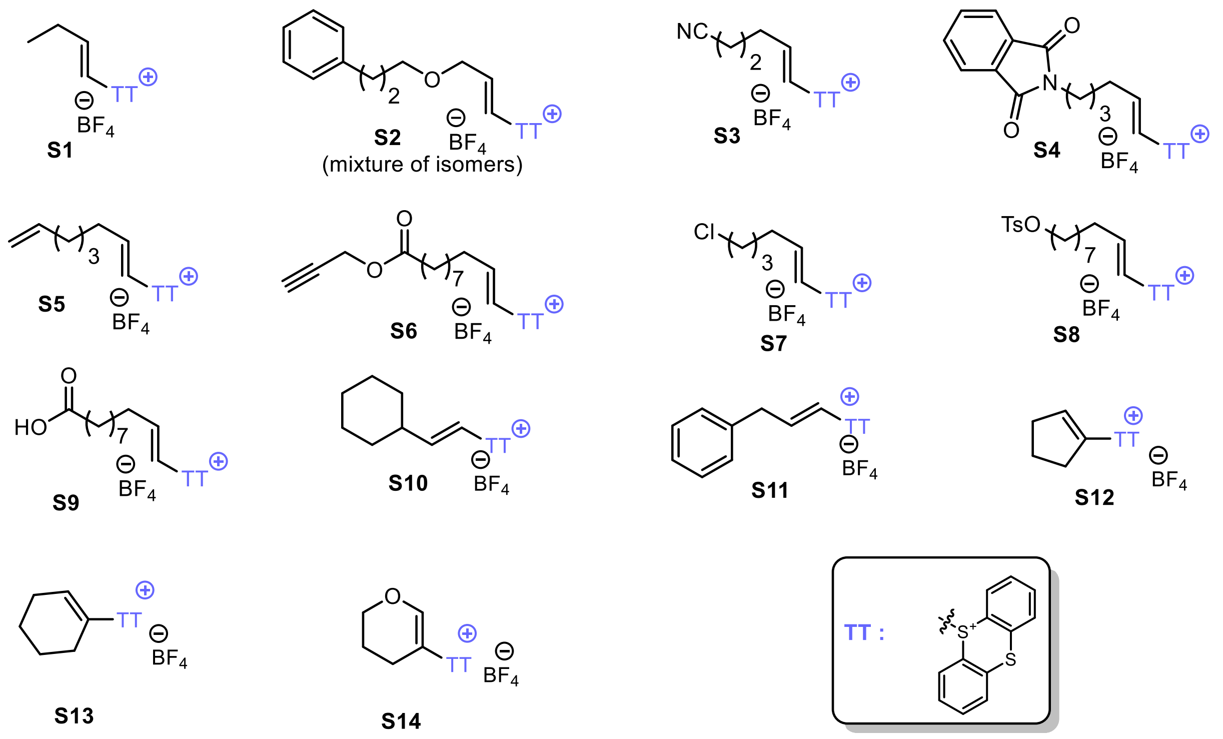

1. Figure Previously reported alkenyl thianthren-5-ium salts

2.1. General procedure

Preparation of alkenyl thianthren-5-ium tetrafluoroborate salts

The preparation of previously not reported thianthrene-5-ium tetrafluoroborate salts was carried out according to the general procedure reported by Soós et al[54]. The preparation of the starting material hex-5-en-1-yl benzoate was carried out according to previously reported methods[72].

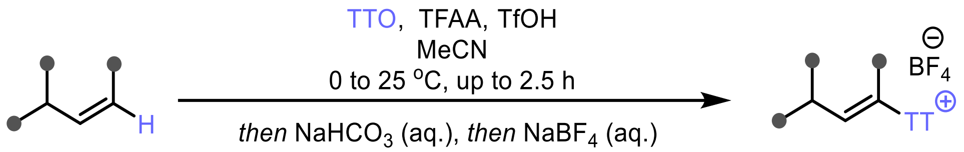

A suspension of alkene (1 equiv.) and thianthrene S-oxide (TTO, 1.03 equiv.) in anhydrous acetonitrile (0.3 M) was cooled to 0 °C under argon atmosphere. [Note 1] At this temperature, trifluoroacetic anhydride (TFAA, 3.00 equiv.) and trifluoromethanesulfonic acid (TfOH, 1.20 equiv.) were added dropwise consecutively. An immediate color change of the reaction mixture was observed at the beginning of the first addition step and the lilac/blue solution was stirred at 0 °C for a further 1 h. [Note 2] Then, the reaction mixture was warmed to 25 °C and stirred at this temperature until analysis by TLC indicated full conversion. [Note 3] At this point, the reaction mixture was concentrated under reduced pressure [Note 4] and subsequently dissolved in dichloromethane (0.1 M). The same amount of saturated aqueous sodium bicarbonate solution was added, and the two-phase mixture was vigorously stirred at 25 °C for 15 min, whereupon a color change to yellow/brown was observed and the phases were separated. [Notes 5,6] This washing step was repeated one more time, after which aqueous sodium tetrafluoroborate solution (5 wt.%) was added to the organic phase and the mixture was stirred vigorously for 15 min and the phases were separated. This washing step was repeated as well one more time. [Note 7] The layers were separated, and the organic phase was dried over sodium sulfate. The dried solution was filtered, and the filtrate was concentrated under reduced pressure. The residue was purified by flash column chromatography on silica gel (gradient elution using isopropanol and dichloromethane) to yield alkenyl thiantren-5-ium tetrafluoroborate salts. [Notes 8,9,10]

[Note 1]: An ice bath was used.
[Note 2]: In the presence of functional groups which are likely to be acetylated by trifluoroacetic anhydride (e.g., alcohols, carboxylic acids), this color change is not immediate but occurs during addition.
[Note 3]: Alkenyl thianthren-5-ium products have a characteristic appearance on TLC, as demonstrated below by a typical example.
[Note 4]: Water jet vacuum pumps were used with gentle heating by a 30 °C water bath.
[Note 5]: This step promotes the formation of alkenyl thianthren-5-ium salts from the precursor dicationic species.
[Note 6]: In some specific cases, this color change was observed before washing with saturated aqueous sodium bicarbonate solution, however, this did not affect the outcome of the reaction.
[Note 7]: This step completes the counterion exchange. Completeness can be verified by ^{19}F-NMR.

[Note 8]: Isopropanol was preferred as the use of less hindered alcohols (i.e., methanol) as eluents may promote decomposition of the product.
[Note 9]: Drying under high vacuum is often required to completely get rid of solvent residues. We consider this to be particularly important as these residues might be reactive under basic conditions (i.e., isopropanol) and promote undesired reaction pathways in the upcoming step.
[Note 10]: The procedure is stereoselective: for terminal olefins, the *E* isomer is predominantly formed, while for internal olefins, the stereochemistry is preserved.

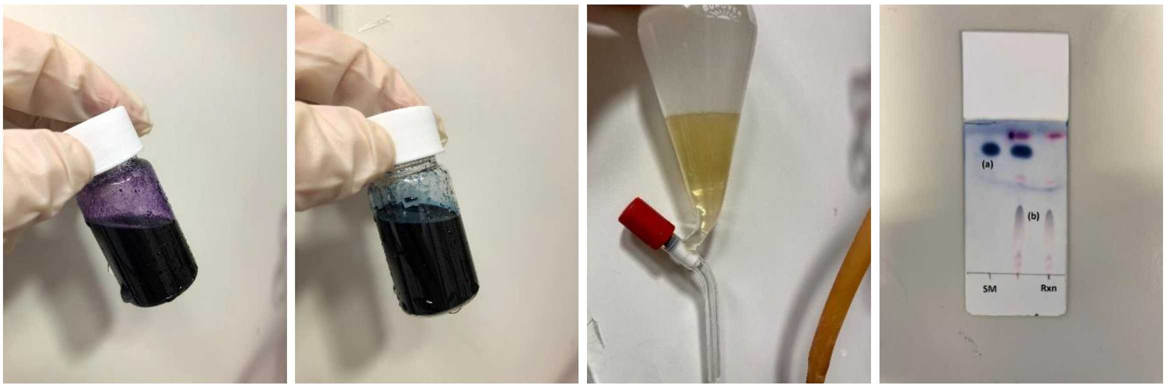

(A-B) Representative lilac/blue color of reaction mixtures; **(C)** yellow color after vigorous stirring with sat. aq. sodium bicarbonate solution; **(D)** a representative TLC of the crude mixture, stained with CAM: (a) alkene; (b) thianthren-5-ium salt

Compound S15 (5-(10-methoxy-10-oxodec-1-en-1-yl)-5*H*-thianthren-5-ium tetrafluoroborate)

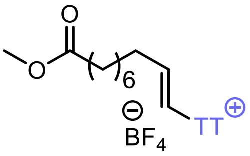

Following **General procedure** with the following specifications: carried out on a 10 mmol scale, methyl 9-decenoate (1.8 g, 10.0 mmol, 1 equiv.); TTO (2.55 g 11.0 mmol, 1.10 equiv.), Tf_2O (2.10 ml, 12.5 mmol, 1.25 equiv.). The residue was purified by precipitation using a mixture of diethyl ether and dichloromethane (104 ml, V/V = 20:1) [Note1] to yield the spectroscopically pure **S15** alkenyl-thianthren-5-ium salt.

[Note1]: In case of purification by precipitation the residue was first dissolved in dichloromethane then, while sonicating the mixture in an ultrasonic bath, diethyl ether was added dropwise to precipitate the product.

Physical State: brown oil

^{1}H-NMR (499.64 MHz, $CDCl_3$): δ = 8.38 (dd, J = 7.9, 1.4 Hz, 2H), 7.82 (dd, J = 7.9, 1.4 Hz, 2H), 7.73 (td, J = 7.7, 1.5 Hz, 2H), 7.67 (td, J = 7.7, 1.4 Hz, 2H), 7.31 – 7.26 (m, 1H), 6.55 (dt, J = 14.7, 1.5 Hz, 1H), 3.65 (s, 3H), 2.24 (dq, J = 8.4, 7.2 Hz, 4H), 1.54 (t, J = 7.4 Hz, 2H), 1.40 (t, J = 7.4 Hz, 2H), 1.26 – 1.18 ppm (m, 6H). (see Spectrum)

^{13}C-NMR (125.65 MHz, $CDCl_3$): δ = 174.1, 157.1, 135.5, 134.3, 133.8, 130.3, 130.0, 120.7, 109.2, 51.4, 33.9, 33.3, 28.8, 28.69, 28.66, 27.1, 24.7 ppm. (see Spectrum)

^{19}F-NMR (282.21 MHz, $CDCl_3$): δ = -150.70 (s), -150.75 ppm (s). (see Spectrum)

HRMS (ESI): M^+ calcd. for $[C_{23}H_{27}O_2S_2]^+$ 399.1447, found 399.1455

Compound S16 (5-(6-bromohex-1-en-1-yl)-5*H*-thianthren-5-ium tetrafluoroborate)

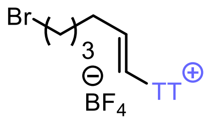

Following **General procedure** with the following specifications: carried out on a 35.0 mmol scale, 6-bromohexene (5.71 g, 30.0 mmol, 1 equiv.); TTO (8.38 g, 36.1 mmol, 1.03 equiv.), TFAA (22.1 g, 105 mmol, 3.00 equiv.), TfOH (6.30 mg, 42.0 mmol, 1.20 equiv.). Purification by flash column chromatography (isopropanol in dichloromethane 0 - 15%) afforded the title compound **S16** (11.5 g, 24.7 mmol, 70%, *E*:*Z* = 93:7)

Physical State: brown oil

***E* isomer**:

^{1}H-NMR (499.64 MHz, DMSO-d_6): δ = 8.27 (dd, J = 8.0, 1.4 Hz, 2H), 8.06 (dd, J = 7.8, 1.3 Hz, 2H), 7.86 (td, J = 7.7, 1.4 Hz, 2H), 7.77 (td, J = 7.7, 1.3 Hz, 2H), 6.86 – 6.74 (m, 2H), 3.47 (t, J = 6.7 Hz, 2H), 2.29 (td, J = 7.4, 5.4 Hz, 2H), 1.73 (dt, J = 14.3, 6.8 Hz, 2H), 1.48 ppm (p, J = 7.4 Hz, 2H). (see Spectrum)

^{13}C-NMR (125.65 MHz, DMSO-d_6): δ = 153.5, 134.4, 134.1, 133.4, 130.0, 129.6, 120.8, 111.6, 34.4, 31.3, 31.1, 25.5 ppm. (see Spectrum)

^{19}F-NMR (282.21 MHz, DMSO-d_6): δ = -144.87 (s), -144.93 ppm (s). (see Spectrum)

HRMS (ESI): M^+ calcd. for $[C_{18}H_{18}BrS_2]^+$ 377.0028, found 377.0013

Compound 2 (5-(7-(benzoyloxy)hept-1-en-1-yl)-5*H*-thianthren-5-ium tetrafluoroborate)

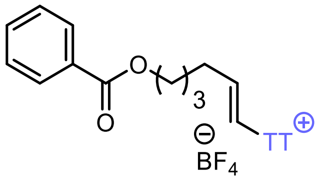

Following **General procedure** with the following specifications: carried out on a 2.45 mmol scale, hex-5-en-1-yl benzoate (**1**, 500 mg, 2.45 mmol, 1 equiv.); TTO (586 mg, 2.52 mmol, 1.03 equiv.), TFAA (1.54 g, 7.34 mmol, 3.00 equiv.), TfOH (441 mg, 2.94 mmol, 1.20 equiv.). Purification by flash column chromatography (isopropanol in dichloromethane 0 – 15 %) afforded the title compound **2** (903 mg, 1.78 mmol. 73%, *E*:*Z* = 94:6)

Physical State: yellow oil

***E* isomer**:

^{1}H-NMR (499.64 MHz, DMSO-d_6): δ = 8.26 (dd, J = 8.0, 1.4 Hz, 2H), 8.05 (dd, J = 7.9, 1.3 Hz, 2H), 7.95 – 7.92 (m, 2H), 7.85 (td, J = 7.7, 1.4 Hz, 2H), 7.77 (td, J = 7.7, 1.4 Hz, 2H), 7.70 – 7.65 (m, 1H), 7.56 – 7.51 (m, 2H), 6.89 – 6.78 (m, 2H), 4.24 (t, J = 6.4 Hz, 2H), 2.35 (q, J = 6.9 Hz, 2H), 1.65 (dt, J = 8.4, 6.5 Hz, 2H), 1.57 – 1.47 ppm (m, 2H). (see Spectrum)

^{13}C-NMR (125.65 MHz, DMSO-d_6): δ = 165.6, 153.8, 134.3, 134.1, 133.4, 133.3, 130.0, 129.7, 129.6, 129.0, 128.7, 120.9, 111.5, 64.1, 31.6, 27.4, 23.6 ppm. (see Spectrum)

^{19}F-NMR (282.21 MHz, DMSO-d_6): δ = -144.87 (s), -144.93 ppm (s). (see Spectrum)

HRMS (ESI): M^+ calcd. for $[C_{25}H_{23}O_2S_2]^+$ 419.1134, found 419.1132

2.2. Specific procedure

Preparation of 21a and 21b alkenyl thianthren-5-ium salt

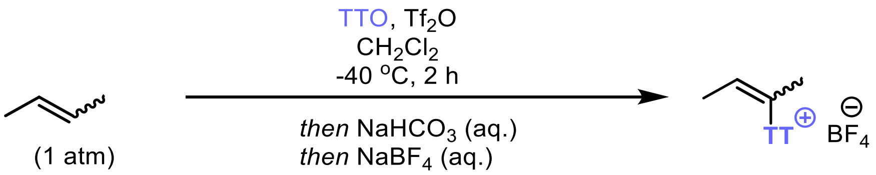

A round-bottom flask (100 mL) capped with a rubber septum and equipped with a stirring bar was charged with the solution of thianthrene S-oxide (1.00 g, 4.30 mmol, 1 equiv.) in anhydrous dichloromethane (35.0 mL, 0.125 M) and the mixture was cooled to -40 °C (internal temperature). (*Z* or *E*)-2-butane was then bubbled through the solution for 15 minutes, after which balloons filled with (*Z* or *E*)-2-butane were connected to the flask to maintain the appropriate atmosphere throughout the reaction. Trifluoromethane sulfonic anhydride (873 µL, 5.17 mmol, 1.20 equiv.) was added dropwise to the reaction, whereupon a dark purple suspension was progressively formed [Note 1]. After stirring at this temperature for further 30 min, the cooling bath was removed and the mixture was stirred at 25 °C for 1.5 h until analysis by LC-MS indicated full conversion. At this point, the balloons and the rubber septum were removed, and saturated aqueous sodium bicarbonate solution (35 mL) was added carefully. The two-phase system was vigorously shaken in a separation funnel, phases were separated and the aqueous layer was extracted with dichloromethane (2x20 mL). All organic phases were then combined, partially concentrated (to around 25 mL). Then, this organic phase was thoroughly washed with aqueous sodium tetrafluoroborate solution (5 wt.%, 5x30 mL) and dried over sodium sulfate. [Note 2] The dried solution was filtered, and the filtrate was concentrated under reduced pressure. The residue was purified by flash column chromatography (isopropanol in dichloromethane 0 -15%) to yield the spectroscopically pure alkyl-thianthren-5-ium salt [Note 3].

[Note 1]: If the suspension cannot be stirred, an additional 15 mL of solvent should be added
[Note 2]: This step promotes counterion exchange; completeness may be practically verified by ^{19}F NMR.

[Note 3]: Drying under high vacuum is often required to completely get rid of solvent residues. This is also important as these might be reactive under basic conditions (i.e. isopropanol) and promote undesired reaction pathways in the upcoming step.

Compound 21a and 21b

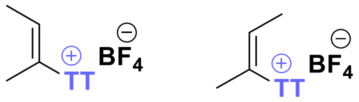

21a and **21b**

Following the above described **Specific procedure** afforded respectively the title compounds **21a** and **21b** (785 mg, 4.30 mmol. 51%).

Compound 21a

Physical State: light brown solid

^{1}H-NMR (499.64 MHz, $CDCl_3$): δ = 8.39 (d, J = 7.8 Hz, 2H), 7.81 – 7.68 (m, 6H), 6.00 (q, J = 7.0 Hz, 1H), 1.87 (s, 3H), 1.82 ppm (d, J = 7.1 Hz, 3H). (see Spectrum)

^{13}C-NMR (125.65 MHz, $CDCl_3$): δ = 139.7, 136.0, 134.9, 134.6, 130.1, 129.8, 121.5, 116.9, 15.2, 13.4 ppm. (see Spectrum)

^{19}F-NMR (282.21 MHz, $CDCl_3$): δ = -151.58, -151.63 ppm. (see Spectrum)

HRMS (ESI): M^+ calcd. for $[C_{16}H_{15}S_2]^+$ 271.0610, found 271.0605

Compound 21b

Physical State: light brown solid

^{1}H-NMR (499.64 MHz, $CDCl_3$): δ = 8.26 (d, J = 7.6 Hz, 2H), 7.80 – 7.63 (m, 6H), 6.59 (q, J = 7.4 Hz, 1H), 2.33 (d, J = 7.2 Hz, 3H), 1.99 ppm (s, 3H). (see Spectrum)

^{13}C-NMR (499.64 MHz, $CDCl_3$): δ = 146.8, 135.5, 134.3, 133.5, 130.2, 129.6, 126.4, 117.2, 20.0, 16.4 ppm. (see Spectrum)

^{19}F-NMR (282.21 MHz, $CDCl_3$): δ = -152.09, -152.15 ppm. (see Spectrum)

HRMS (ESI): M^+ calcd. for $[C_{16}H_{15}S_2]^+$ 271.0610, found 271.0607

3. Preparation of alkyl thianthren-5-ium tetrafluoroborate salts

3.1. General procedure

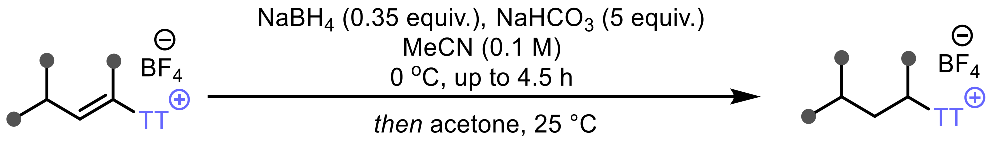

A suspension of the alkenyl thianthren-5-ium salt (1 equiv.) and sodium bicarbonate (5.00 equiv.) in anhydrous acetonitrile (0.1 M) was cooled to 0 °C under argon atmosphere. [Note 1, 2] Then sodium borohydride (0.35 equiv.) was added in one portion at this temperature. After stirring the reaction mixture for 30 min – 4.5 h, until analysis by LC-MS indicated full conversion, [Note 3] acetone (75 equiv.) was added in one portion. The resulting mixture was warmed to 25 °C and stirred at this temperature for an additional 5 min. [Note 4, 5] At this point, the reaction mixture was filtered [Note 6] and concentrated under reduced pressure. [Note 7] The residue was purified either by trituration using anhydrous diethyl ether (2 or 3 cycles) or by precipitation using a mixture of anhydrous diethyl ether and dichloromethane (V/V = 20:1 or 10:1 or 5:1 or 12:5) [Note 8, 9, 10] to yield the spectroscopically pure alkyl-thianthren-5-ium salt [Note 11].

[Note 1]: An ice bath was used.
[Note 2]: The use of oven dried glassware is recommended as alkyl thianthren-5-ium salts may be water sensitive.
[Note 3]: Using TLC analysis instead of LC-MS to indicate completion of the reaction is not reliable in most cases as R_f values of the starting materials and the products are often identical.
[Note 4]: The ice bath was changed to a water bath to ensure rapid rise of temperature.
[Note 5]: This step ensures quenching the excess of the reductant.
[Note 6]: Water jet vacuum pumps were used.
[Note 7]: The temperature of the water bath was set to 25 °C.
[Note 8]: An ultrasonic bath was used in case of performing a trituration or precipitation.
[Note 9]: Drying under high vacuum is often required to completely get rid of solvent residues.
[Note 10]: In case of purification by precipitation the residue was first dissolved in dichloromethane then, while sonicating the mixture in an ultrasonic bath, diethyl ether was added dropwise to precipitate the product.
[Note 11]: Alkyl thianthren-5-ium salts are (moisture) sensitive alkylating agents in general that cannot be purified by column chromatography without significant losses in yields due to decomposition. For the same reason, the NMR measurements have to be carried out using oven-dried NMR tubes and anhydrous (i.e. dried by molecular sieves) non-nucleophilic deuterated solvents (i.e. DMSO-d6 is reactive). Decomposition is vividly indicated by the characteristic ^{1}H-NMR signals of thianthrene.

Compound 3 (5-(6-(benzoyloxy)hexyl)-5H-thianthren-5-ium tetrafluoroborate)

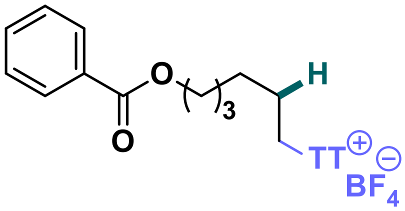

Following **General procedure** with the following modifications: carried out on a 0.75 mmol scale (377 mg , 1 equiv.) ; sodium borohydride (9.9 mg, 0.26 mmol, 0.35 equiv.) 1 h reaction time at 0 °C. Purification by precipitation with the mixture of diethyl ether and dichloromethane (10 mL, V/V = 10:1, 1 cycle) afforded the title compound **3** (196 mg, 0.386 mmol, 52%).

Physical State: white amorphous solid

^{1}H-NMR (499.64 MHz, $CDCl_3$): δ = 8.27 (dd, J = 7.8, 1.4 Hz, 2H), 7.98 (d, J = 7.2 Hz, 2H), 7.81 (dd, J = 7.9, 1.5 Hz, 2H), 7.74 (td, J = 7.7, 1.4 Hz, 2H), 7.66 (td, J = 7.6, 1.4 Hz, 2H), 7.56 – 7.51 (m, 1H), 7.42 (t, J = 7.7 Hz, 2H), 4.22 (t, J = 6.5 Hz, 2H), 3.78 – 3.71 (m, 2H), 1.67 (p, J = 6.8 Hz, 2H), 1.59 (p, J = 7.6 Hz, 2H), 1.51 – 1.42 (m, 2H), 1.37 ppm (q, J = 7.3 Hz, 2H). (see Spectrum)

^{13}C-NMR (125.65 MHz, $CDCl_3$): δ = 166.5, 135.6, 134.7, 134.5, 132.9, 130.32, 130.30, 130.0, 129.5, 128.3, 117.3, 64.5, 40.3, 28.2, 27.4, 25.3, 24.3 ppm. (see Spectrum)

^{19}F-NMR (282.21 MHz, $CDCl_3$): δ = -150.91 (s), -150.97 ppm (s). (see Spectrum)

HRMS (ESI): M^+ calcd. for $[C_{25}H_{25}O_2S_2]^+$ 421.1290, found 421.1304

Compound 6 (5-(10-methoxy-10-oxodecyl)-5H-thianthren-5-ium tetrafluoroborate)

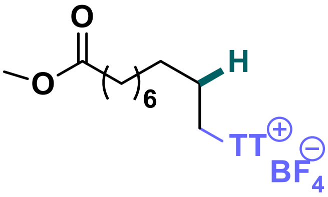

Following **General procedure** with the following modifications: carried out on a 9.25 mmol scale (4.50 g, 1 equiv.); sodium borohydride (122 mg, 3.24 mmol, 0.35 equiv.), 30 min reaction time at 0 °C. Purification by precipitation with the mixture of diethyl ether and dichloromethane (188 mL, V/V = 20:1, 1 cycle) afforded the title compound **6** (3.2 g, 6.6 mmol, 71%).

Physical State: brown oil

^{1}H-NMR (499.64 MHz, $CDCl_3$): δ = 8.29 (dd, J = 7.8, 1.4 Hz, 2H), 7.83 (dd, J = 7.9, 1.3 Hz, 2H), 7.75 (td, J = 7.7, 1.4 Hz, 2H), 7.67 (td, J = 7.6, 1.4 Hz, 2H), 3.76 – 3.69 (m, 2H), 3.64 (s, 3H), 2.26 (t, J = 7.5 Hz, 2H), 1.54 (h, J = 7.3 Hz, 4H), 1.35 (p, J = 7.1 Hz, 2H), 1.31 – 1.12 (m, 8H). (see Spectrum)

^{13}**C-NMR** (125.65 MHz, $CDCl_3$): δ = 174.2, 135.6, 134.8, 134.4, 129.99, 129.95, 117.5, 51.4, 40.4, 34.0, 28.89, 28.88, 28.80, 28.6, 27.8, 24.8, 24.4 ppm. (see Spectrum)

^{19}**F-NMR** (282.21 MHz, $CDCl_3$): δ = -150.97(s), -151.02 ppm(s). (see Spectrum)

HRMS (ESI): M^+ calcd. for $[C_{23}H_{29}O_2S_2]^+$ 401.1603, found 401.1602

Compound 7 (5-butyl-5*H*-thianthren-5-ium tetrafluoroborate)

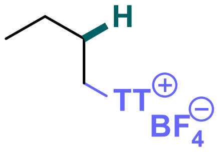

Following **General procedure** with the following modifications: carried out on a 1.72 mmol scale (616 mg, 1 equiv.); sodium borohydride (22.8 mg, 0.602 mmol, 0.35 equiv.), 2 h reaction time at 0 °C. After 1 h the reaction was not completed, therefore additional sodium borohydride (9.8 mg, 0.26 mmol, 0.15 equiv.) was added in one portion to the reaction mixture. Purification by precipitation with the mixture of diethyl ether and dichloromethane (12 mL, V/V = 5:1, 3 cycles) afforded the title compound **7** (400 mg, 1.11 mmol, 65%).

Physical State: white amorphous solid

^{1}**H-NMR** (499.64 MHz, $CDCl_3$): δ = 8.32 (dd, J = 7.8, 1.4 Hz, 2H), 7.82 (dd, J = 7.8, 1.4 Hz, 2H), 7.75 (td, J = 7.6, 1.4 Hz, 2H), 7.68 (td, J = 7.7, 1.4 Hz, 2H), 3.78 – 3.71 (m, 2H), 1.53 (tt, J = 8.0, 6.1 Hz, 2H), 1.42 (h, J = 7.3 Hz, 2H), 0.85 ppm (t, J = 7.3 Hz, 3H). (see Spectrum)
^{13}**C-NMR** (125.65 MHz, $CDCl_3$): δ = 135.6, 134.9, 134.4, 130.0, 129.9, 117.5, 40.3, 26.4, 21.2, 13.3 ppm. (see Spectrum)

^{19}**F-NMR** (282.21 MHz, $CDCl_3$): δ = -151.06 (s), -151.11 ppm (s). (see Spectrum)

HRMS (ESI): M^+ calcd. for $[C_{16}H_{17}S_2]^+$ 273.0766, found 273.0760

Compound 8 (5-(2-cyclohexylethyl)-5*H*-thianthren-5-ium tetrafluoroborate)

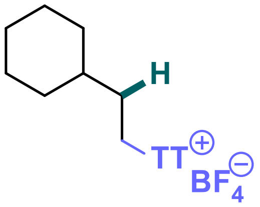

Following **General procedure** with the following modifications: carried out on a 0.75 mmol scale (311 mg, 1 equiv.); sodium borohydride (10.0 mg, 0.264 mmol, 0.35 equiv.), 1 h reaction time at 0 °C. Purification by trituration with diethyl ether (10 mL, 3 cycles) afforded the title compound **8** (236 mg, 0.570 mmol, 76%).

Physical State: white amorphous solid

^{1}H-NMR (499.64 MHz, $CDCl_3$): δ = 8.31 (dd, J = 7.9, 1.4 Hz, 2H), 7.82 (dd, J = 7.9, 1.4 Hz, 2H), 7.75 (td, J = 7.7, 1.4 Hz, 2H), 7.68 (td, J = 7.7, 1.4 Hz, 2H), 3.79 – 3.71 (m, 2H), 1.66 – 1.52 (m, 5H), 1.48 – 1.39 (m, 2H), 1.29 (ddt, J = 11.0, 7.3, 3.8 Hz, 1H), 1.18 – 0.98 (m, 3H), 0.87 – 0.74 ppm (m, 2H). (see Spectrum)

^{13}C-NMR (125.65 MHz, $CDCl_3$): δ = 135.5, 135.0, 134.4, 130.0, 129.9, 117.7, 38.8, 36.8, 32.5, 31.3, 26.0, 25.8 ppm. (see Spectrum)

^{19}F-NMR (282.21 MHz, $CDCl_3$): δ = -151.07 (s), -151.13 ppm (s). (see Spectrum)

HRMS (ESI): M^+ calcd. for $[C_{20}H_{23}S_2]^+$ 327.1236, found 327.12302

Compound 9 (5-(3-phenylpropyl)-5*H*-thianthren-5-ium tetrafluoroborate)

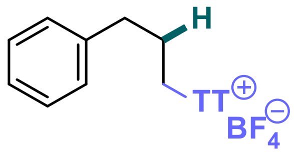

Following General procedure with the following modifications: carried out on a 0.71 mmol scale (300 mg, 1 equiv.); sodium borohydride (9.5 mg, 0.25 mmol, 0.35 equiv.), 1 h reaction time at 0 °C. Purification by precipitation with the mixture of diethyl ether and dichloromethane (11 mL, V/V = 10:1, 3 cycles) afforded the title compound **9** (220 mg, 0.530 mmol, 74%).

Physical State: yellow amorphous solid

^{1}H-NMR (499.64 MHz, $CDCl_3$): δ = 8.21 (d, J = 7.8 Hz, 2H), 7.79 – 7.75 (m, 2H), 7.73 (t, J = 7.6 Hz, 2H), 7.66 – 7.61 (m, 2H), 7.19 (dp, J = 14.2, 7.2 Hz, 4H), 7.01 (d, J = 7.3 Hz, 2H), 3.76 – 3.68 (m, 2H), 2.71 (t, J = 7.4 Hz, 2H), 1.89 ppm (p, J = 7.6 Hz, 2H).(see Spectrum)

^{13}C-NMR (125.65 MHz, $CDCl_3$): δ = 138.9, 135.6, 134.7, 134.4, 130.0, 129.9, 128.7, 128.4, 126.6, 117.2, 39.4, 33.5, 25.7 ppm. (see Spectrum)

^{19}F-NMR (282.21 MHz, $CDCl_3$): δ = -150.80 (s), -150.85 ppm(s). (see Spectrum)

HRMS (ESI): M^+ calcd. for $[C_{21}H_{19}S_2]^+$, 335.0923 found 335,09154

Compound 10 (5-(oct-5-en-1-yl)-5H-thianthren-5-ium tetrafluoroborate)

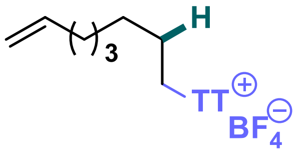

Following **General procedure** with the following modifications: carried out on a 0.73 mmol scale (300 mg, 1 equiv.); sodium borohydride (9.6 mg, 0.26 mmol, 0.35 equiv.), 30 min reaction time at 0 °C. Purification by precipitation with the mixture of diethyl ether and dichloromethane (12 mL, V/V = 5:1, 3 cycles) afforded the title compound **10** (223 mg, 0.538 mmol, 74%)

Physical State: white amorphous solid

^{1}H-NMR (499.64 MHz, $CDCl_3$): δ = 8.30 (dd, J = 7.9, 1.4 Hz, 2H), 7.82 (dd, J = 7.9, 1.3 Hz, 2H), 7.75 (td, J = 7.6, 1.4 Hz, 2H), 7.68 (td, J = 7.7, 1.4 Hz, 2H), 5.71 (ddt, J = 16.9, 10.2, 6.7 Hz, 1H), 4.96 – 4.87 (m, 2H), 3.77 – 3.70 (m, 2H), 1.98 – 1.92 (m, 2H), 1.55 (p, J = 7.7 Hz, 2H), 1.37 (p, J = 7.3 Hz, 2H), 1.30 – 1.17 ppm (m, 4H).(see Spectrum)
^{13}C-NMR (125.65 MHz, $CDCl_3$): δ = 138.5, 135.6, 134.9, 134.4, 130.0, 129.9, 117.5, 114.5 40.4, 33.4, 28.3, 28.2, 27.7, 24.4 ppm. (see Spectrum)

^{19}F-NMR (282.21 MHz, $CDCl_3$): δ = -151.08 (s), -151.13 ppm (s). (see Spectrum)

HRMS (ESI): M^+ calcd. for $[C_{20}H_{23}S_2]^+$ 327.1236, found 327.1241

Compound 11 (5-(5-oxo-5-(prop-2-yn-1-yloxy)undecyl)-5*H*-thianthren-5-ium tetrafluoroborate)

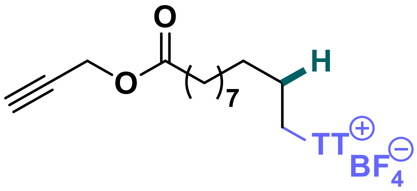

Following **General procedure** with the following modifications: carried out on a 1.5 mmol scale (810 mg, 1 equiv.); sodium borohydride (20.5 mg, 0.541 mmol, 0.35 equiv.), 30 min reaction time at 0 °C. Purification by precipitation with the mixture of diethyl ether and dichloromethane (12 mL, V/V = 5:1, 3 cycles) afforded the title compound **11** (480 mg, 0.912 mmol, 59 %).

Physical State: white amorphous solid

^{1}H-NMR (499.64 MHz, $CDCl_3$): δ = 8.29 (dd, J = 8.0, 1.4 Hz, 2H), 7.83 (dd, J = 7.9, 1.3 Hz, 2H), 7.75 (td, J = 7.7, 1.3 Hz, 2H), 7.67 (td, J = 7.7, 1.4 Hz, 2H), 4.66 (d, J = 2.5 Hz, 2H), 3.76 – 3.69 (m, 2H), 2.46 (t, J = 2.5 Hz, 1H), 2.32 (t, J = 7.5 Hz, 2H), 1.56 (dp, J = 26.6, 7.6 Hz, 4H), 1.40 – 1.10 ppm (m, 12H). (see Spectrum)
^{13}C-NMR (125.65 MHz, $CDCl_3$): δ = 172.9, 135.6, 134.9, 134.4, 130.0, 129.9, 117.6, 77.8, 74.7, 51.7, 40.4, 33.9, 29.05, 28.98, 28.94, 28.88, 28.7, 27.8, 24.7, 24.5 ppm. (see Spectrum)

^{19}F-NMR (282.21 MHz, $CDCl_3$): δ = -151.19 (s), -151.24 ppm (s). (see Spectrum)

HRMS (ESI): M$^+$ calcd. for $[C_{26}H_{31}O_2S_2]^+$ 439.1760, found 439.1763

Compound 12 (5-(4-cyanopentyl)-5*H*-thianthren-5-ium tetrafluoroborate)

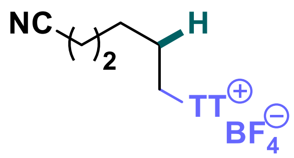

Following **General procedure** with the following modifications: carried out on a 0.77 mmol scale (305 mg, 1 equiv.); sodium borohydride (10.2 mg, 0.269 mmol, 0.35 equiv.), 1.5 h reaction time at 0 °C. After 1 h the reaction was not completed, therefore additional sodium borohydride (4.4 mg, 0.12 mmol, 0.15 equiv.) was added in one portion to the reaction mixture. Purification by trituration with diethyl ether (10 mL, 3 cycles) afforded the title compound **12** (222 mg, 0.556 mmol, 73%).

Physical State: yellow amorphous solid

^{1}H-NMR (499.64 MHz, $CDCl_3$): δ = 8.29 (dd, *J* = 7.9, 1.3 Hz, 2H), 7.84 (dd, *J* = 7.9, 1.3 Hz, 2H), 7.77 (td, *J* = 7.7, 1.4 Hz, 2H), 7.70 (td, *J* = 7.6, 1.4 Hz, 2H), 3.78 (dd, *J* = 8.8, 6.3 Hz, 2H), 2.35 (t, *J* = 6.7 Hz, 2H), 1.66 – 1.62 (m, 4H), 1.61 – 1.55 ppm (m, 2H). (see Spectrum)

^{13}C-NMR (125.65 MHz, $CDCl_3$): δ = 135.6, 134.8, 134.6, 130.1, 130.1, 119.5, 117.2, 40.1, 26.5, 24.4, 23.6, 16.6 ppm. (see Spectrum)

^{19}F-NMR (282.21 MHz, $CDCl_3$): δ = -150.77 (s), -150.82 ppm (s). (see Spectrum)

HRMS (ESI): M$^+$ calcd. for $[C_{18}H_{18}NS_2]^+$, 312.0875 found 312.0876

Compound 13 (5-(6-(1,3-dioxoisoindolin-2-yl)hexyl)-5*H*-thianthren-5-ium tetrafluoroborate)

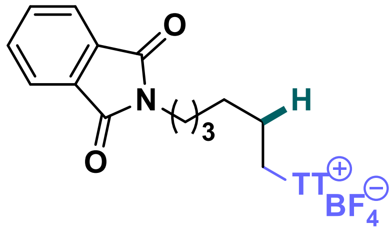

Following **General procedure** with the following modifications: carried out on a 0.60 mmol scale (319 mg, 1 equiv.); sodium borohydride (7.9 mg, 0.21 mmol, 0.35 equiv.), 30 min reaction time at 0 °C. Purification by precipitation with the mixture of diethyl ether and dichloromethane (10 mL, V/V = 20:1, 1 cycle) afforded the title compound **13** (265 mg, 0.497 mmol, 83%).

Physical State: white amorphous solid

^{1}H-NMR (499.64 MHz, $CDCl_3$): δ = 8.32 (dd, J = 7.9, 1.4 Hz, 2H), 7.83 – 7.79 (m, 4H), 7.76 – 7.65 (m, 6H), 3.77 – 3.71 (m, 2H), 3.60 (t, J = 7.0 Hz, 2H), 1.58 – 1.52 (m, 4H), 1.43 (p, J = 7.5 Hz, 2H), 1.29 – 1.23 ppm (m, 2H). (see Spectrum)

^{13}C-NMR (125.65 MHz, $CDCl_3$): δ = 168.3, 135.6, 134.9, 134.4, 133.9, 132.1, 130.01, 129.97, 123.2, 117.4, 40.2, 37.5, 28.0, 27.2, 25.9, 24.3 ppm. (see Spectrum)

^{19}F-NMR (282.21 MHz, $CDCl_3$): δ = -151.13 (s), -151.18 ppm (s). (see Spectrum)

HRMS (ESI): M^+ calcd. for $[C_{26}H_{24}NO_2S_2]^+$ 446.1243, found 446.1244

Compound 14 (5-(3-(3-phenylpropoxy)propyl)-5*H*-thianthren-5-ium tetrafluoroborate)

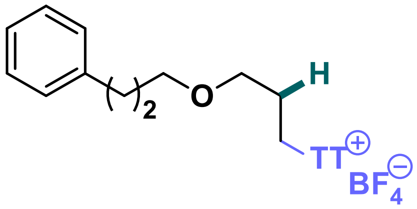

Following **General procedure** with the following modifications: carried out on a 0.87 mmol scale (417 mg, 1 equiv.); sodium borohydride (11.5 mg, 0.305 mmol, 0.35 equiv.), 1.5 h reaction time at 0 °C. After 1 h the reaction was not completed, therefore additional sodium borohydride (5.0 mg, 0.13 mmol, 0.15 equiv.) was added in one portion to the reaction mixture. Purification by precipitation with the mixture of diethyl ether and dichloromethane (11 mL, V/V = 10:1, 3 cycles) afforded the title compound **14** (260 mg, 0.541 mmol, 62%)

Physical State: white amorphous solid

^{1}H-NMR (499.64 MHz, $CDCl_3$): δ = 8.30 (dd, J = 7.9, 1.4 Hz, 2H), 7.79 (dd, J = 7.9, 1.3 Hz, 2H), 7.72 (td, J = 7.7, 1.4 Hz, 2H), 7.66 (td, J = 7.7, 1.4 Hz, 2H), 7.28 (d, J = 7.7 Hz, 3H), 7.19 – 7.15 (m, 3H), 3.89 (t, J = 7.1 Hz, 2H), 3.55 (t, J = 5.5 Hz, 2H), 3.46 (t, J = 6.6 Hz, 2H), 2.68 – 2.62 (m, 2H), 1.93 – 1.86 (m, 2H), 1.83 ppm (p, J = 6.3 Hz, 2H).(see Spectrum)

^{13}C-NMR (100.56 MHz, $CDCl_3$): δ =141.8, 135.6, 135.1, 134.3, 129.9, 129.8, 128.4, 125.8, 117.6, 77.3, 77.0, 76.7, 70.5, 68.1, 38.9, 32.3, 31.2, 24.9 ppm.(see Spectrum)

^{19}F-NMR (282.21 MHz, $CDCl_3$): δ = -151.19 (s), -151.24 ppm (s). (see Spectrum)

HRMS (ESI): M^+ calcd. for $[C_{24}H_{25}OS_2]^+$ 393,1341, found 393.1330

Compound 15 (5-(6-chlorohexyl)-5H-thianthren-5-ium tetrafluoroborate)

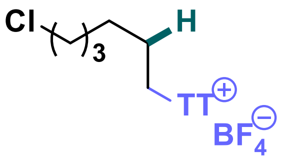

Following **General procedure** with the following modifications: carried out on a 0.86 mmol scale (360 mg, 1 equiv.); sodium borohydride (11.3 mg, 0.299 mmol, 0.35 equiv.), 30 min reaction time at 0 °C. Purification by precipitation with the mixture of diethyl ether and dichloromethane (11 mL, V/V = 10:1, 3 cycles) afforded the title compound **15** (232 mg, 0.548 mmol, 64 %).

Physical State: white solid

^{1}H-NMR (499.64 MHz, $CDCl_3$): δ = 8.22 (dd, J = 7.9, 1.4 Hz, 2H), 7.84 (dd, J = 7.9, 1.3 Hz, 2H), 7.77 (td, J = 7.7, 1.4 Hz, 2H), 7.66 (td, J = 7.7, 1.3 Hz, 2H), 3.75 – 3.69 (m, 2H), 3.44 (t, J = 6.5 Hz, 2H), 1.65 (p, J = 6.7 Hz, 2H), 1.55 (p, J = 7.3 Hz, 2H), 1.45 – 1.30 ppm (m, 4H).(see Spectrum)
^{13}C-NMR (125.65 MHz, $CDCl_3$): δ = 135.6, 134.9, 134.4, 130.0, 130.0, 117.5, 44.8, 40.3, 31.8, 26.9, 25.9, 24.3 ppm. (see Spectrum)

^{19}F-NMR (282.21 MHz, $CDCl_3$): δ = -151.01 (s), -151.06 ppm (s). (see Spectrum)

HRMS (ESI): M^+ calcd. for $[C_{18}H_{20}ClS_2]^+$ 335.0689, found 335.0687

Compound 16 (5-(6-bromohexyl)-5H-thianthren-5-ium tetrafluoroborate)

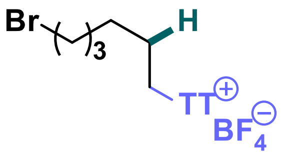

Following **General procedure** with the following modifications: carried out on a 1.8 mmol scale (830 mg, 1 equiv.); sodium borohydride (23.6 mg, 0.625 mmol, 0.35 equiv.), 1.5 h reaction time at 0 °C. After 1 h the reaction was not completed, therefore additional sodium borohydride (10.1 mg, 0.268 mmol, 0.15 equiv.) was added in one portion to the reaction mixture. Purification by precipitation with the mixture of diethyl ether and dichloromethane (11 mL, V/V = 10:1, 3 cycles) afforded the title compound **16** (420 mg, 0.899 mmol, 50 %).

Physical State: white solid

^{1}H-NMR (499.64 MHz, $CDCl_3$): δ = 8.31 (dd, J = 7.8, 1.3 Hz, 2H), 7.82 (dd, J = 7.8, 1.3 Hz, 2H), 7.75 (td, J = 7.7, 1.4 Hz, 2H), 7.69 (td, J = 7.7, 1.4 Hz, 2H), 3.79 – 3.74 (m, 2H), 3.36 (t, J = 6.5 Hz, 2H), 1.77 (p, J = 6.7 Hz, 2H), 1.58 (p, J = 7.4 Hz, 3H), 1.47 – 1.34 ppm (m, J = 21.8, 6.8 Hz, 4H).(see Spectrum)
^{13}C-NMR (125.65 MHz, $CDCl_3$): δ = 135.6, 134.9, 134.4, 130.01, 129.98, 117.4, 40.3, 33.6, 31.9, 27.1, 26.8, 24.3 ppm. (see Spectrum)

^{19}F-NMR (282.21 MHz, $CDCl_3$): δ = -150.97 (s), -151.03 ppm (s). (see Spectrum)

HRMS (ESI): M$^+$ calcd. for $[C_{18}H_{20}BrS_2]^+$ 379.0184, found 379.0174

Compound 17 (5-(6-(1,3-dioxoisoindolin-2-yl)hexyl)-5*H*-thianthren-5-ium tetrafluoroborate)

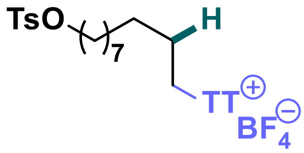

Following **General procedure** with the following modifications: carried out on a 0.74 mmol scale (450 mg, 1 equiv.); sodium borohydride (9.7 mg, 0.26 mmol, 0.35 equiv.), 1.5 h reaction time at 0 °C. After 1 h the reaction was not completed, therefore additional sodium borohydride (4.2 mg, 0.11 mmol, 0.15 equiv.) was added in one portion to the reaction mixture. Purification by precipitation with the mixture of diethyl ether and dichloromethane (11 mL, V/V = 10:1, 3 cycles) afforded the title compound **17** (226 mg, 0.367 mmol, 50%)

Physical State: white amorphous solid

^{1}H-NMR (499.64 MHz, $CDCl_3$): δ = 8.31 (dd, *J* = 7.9, 1.4 Hz, 2H), 7.82 (dd, *J* = 7.9, 1.4 Hz, 2H), 7.79 – 7.72 (m, 4H), 7.68 (td, *J* = 7.7, 1.3 Hz, 2H), 7.35 (d, *J* = 8.0 Hz, 2H), 4.00 (t, *J* = 6.5 Hz, 2H), 3.77 – 3.71 (m, 2H), 2.45 (s, 3H), 1.66 – 1.50 (m, 4H), 1.40 – 1.12 ppm (m, 12H).(see Spectrum)
^{13}C-NMR (125.65 MHz, $CDCl_3$): δ = 144.7, 135.6, 134.7, 134.5, 130.0, 129.8, 128.7, 127.8, 127.6, 117.4, 70.7, 40.4, 28.9, 28.8, 28.7, 28.6, 28.5, 27.7, 25.2, 24.4, 21.6 ppm. (see Spectrum)

^{19}F-NMR (282.21 MHz, $CDCl_3$): δ = -151.00 (s), -151.06 ppm (s). (see Spectrum)

HRMS (ESI): M$^+$ calcd. for $[C_{29}H_{35}O_3S_3]^+$ 527.1743, found 527.1746

Compound 18 (5-cyclopentyl-5*H*-thianthren-5-ium tetrafluoroborate)

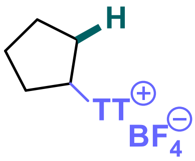

Following **General procedure** with the following modifications: carried out on a 1.7 mmol scale (620 mg, 1 equiv.); sodium borohydride (22.2 mg, 0.586 mmol, 0.35 equiv.), 1.5 h reaction time at 0 °C. After 1 h the reaction was not completed, therefore additional sodium borohydride (9.5 mg, 0.25 mmol, 0.15 equiv.) was added in one portion to the reaction mixture. Purification by precipitation with the mixture of diethyl ether and dichloromethane (12 mL, V/V = 5:1, 3 cycles) afforded the title compound **18** (340 mg, 0.913 mmol, 55 %).

Physical State: white solid

^{1}H-NMR (499.64 MHz, $CDCl_3$): δ = 8.38 (dd, *J* = 7.8, 1.3 Hz, 2H), 7.80 (dd, *J* = 7.9, 1.3 Hz, 2H), 7.74 (td, *J* = 7.6, 1.4 Hz, 2H), 7.68 (td, *J* = 7.6, 1.4 Hz, 2H), 4.62 (q, *J* = 6.7 Hz, 1H), 2.07 (d, *J* = 13.3 Hz, 2H), 1.99 (d, *J* = 6.4 Hz, 2H), 1.69 (d, *J* = 4.2 Hz, 4H). (see Spectrum)

^{13}C-NMR (125.65 MHz, $CDCl_3$): δ = 135.6, 134.8, 134.5, 129.9, 129.9, 118.0, 52.9, 29.5, 24.8 ppm. (see Spectrum)

^{19}F-NMR (282.21 MHz, $CDCl_3$): δ = -150.93 (s), -150.98 ppm (s). (see Spectrum)

HRMS (ESI): M^+ calcd. for $[C_{17}H_{17}S_2]^+$ 285.0766, found 285.0757

Compound 19 (5-cyclohexyl-5*H*-thianthren-5-ium tetrafluoroborate)

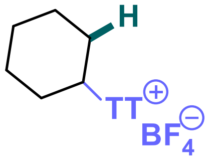

Following **General procedure** with the following modifications: carried out on a 1.6 mmol scale (630 mg, 1 equiv.); sodium borohydride (21.7 mg, 0.574 mmol, 0.35 equiv.), 1.5 h reaction time at 0 °C. After 1 h the reaction was not completed, therefore additional sodium borohydride (9.30 mg, 0.246 mmol, 0.15 equiv.) was added in one portion to the reaction mixture. Purification by precipitation with the mixture of diethyl ether and dichloromethane (12 mL, V/V = 5:1, 3 cycles) afforded the title compound **19** (400 mg, 1.04 mmol, 63 %).

Physical State: white solid

^{1}H-NMR (499.64 MHz, $CDCl_3$): δ = 8.29 (dd, J = 7.9, 1.4 Hz, 2H), 7.82 (dd, J = 7.9, 1.4 Hz, 2H), 7.76 (td, J = 7.7, 1.4 Hz, 2H), 7.66 (td, J = 7.7, 1.4 Hz, 2H), 4.22 (tt, J = 11.4, 3.9 Hz, 1H), 1.93 – 1.78 (m, 4H), 1.61 (dt, J = 14.5, 3.8 Hz, 1H), 1.56 – 1.48 (m, 2H), 1.43 – 1.32 (m, 1H), 1.20 ppm (q, J = 12.4 Hz, 2H).(see Spectrum)

^{13}C-NMR (125.65 MHz, $CDCl_3$): δ = 135.6, 135.5, 134.4, 129.9, 129.8, 116.7, 54.3, 27.7, 25.1, 24.2 ppm. (see Spectrum)

^{19}F-NMR (282.21 MHz, $CDCl_3$): δ = -150.75 (s), -150.80 ppm (s). (see Spectrum)

HRMS (ESI): M^+ calcd. for $[C_{18}H_{19}S_2]^+$ 299,0923, found 299,0913

Compound 20 (5-(tetrahydro-2*H*-pyran-3-yl)-5*H*-thianthren-5-ium tetrafluoroborate)

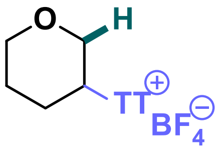

Following **General procedure** with the following modifications: carried out on a 2.1 mmol scale (800 mg, 1 equiv.); sodium borohydride (27.4 mg, 0.725 mmol, 0.35 equiv.), 4.5 h reaction time at 0 °C. After 1 h the reaction was not completed, therefore additional sodium borohydride (11.8 mg, 0.311 mmol, 0.15 equiv.) was added in one portion to the reaction mixture. This cycle was repeated 4 times until full conversion was observed. Purification by precipitation with the mixture of diethyl ether and dichloromethane (34 mL, V/V = 12:5, 3 cycles) afforded the title compound **20** (390 mg, 1.00 mmol, 49 %).

Physical State: white solid

^{1}H-NMR (499.64 MHz, $CDCl_3$): δ = 8.35 (dd, *J* = 7.9, 1.4 Hz, 1H), 8.22 (dd, *J* = 7.9, 1.4 Hz, 1H), 7.85 (dt, *J* = 7.9, 1.4 Hz, 2H), 7.77 (tt, *J* = 7.7, 1.7 Hz, 2H), 7.69 (dtd, *J* = 18.8, 7.7, 1.4 Hz, 2H), 4.51 (tt, *J* = 7.0, 3.6 Hz, 1H), 3.86 (ddd, *J* = 11.0, 6.8, 3.7 Hz, 1H), 3.72 (m, 2H), 3.65 (dd, *J* = 12.9, 2.8 Hz, 1H), 2.17 – 2.07 (m, 1H), 2.07 – 2.00 (m, 1H), 1.92 – 1.84 (m, 1H), 1.70 – 1.62 ppm (m, 1H). (see Spectrum)

^{13}C-NMR (125.65 MHz, $CDCl_3$): δ = 136.2, 136.0, 135.4, 135.0, 134.73, 134.68, 130.3, 130.2, 130.1, 129.9, 116.0, 115.9, 68.2, 66.1, 51.7, 24.3, 23.2 ppm. (see Spectrum)

^{19}F-NMR (282.21 MHz, $CDCl_3$): δ = -151.19 (s), -151.24 ppm (s). (see Spectrum)

HRMS (ESI): M^+ calcd. for $[C_{17}H_{17}OS_2]^+$ 301.0715, found 301.0711

Compound 22 (5-(sec-butyl)-5*H*-thianthren-5-ium tetrafluoroborate)

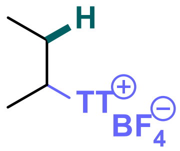

from the (Z)-alkene:

Following **General procedure** with the following modifications: carried out on a 2.79 mmol scale (1.00 g, 1 equiv.); sodium borohydride (37.0 mg, 0.977 mmol, 0.35 equiv.), 1.5 h reaction time at 0 °C. After 1 h the reaction was not completed, therefore additional sodium borohydride (15.8 mg, 0.419 mmol, 0.15 equiv.) was added in one portion to the reaction mixture. Purification by precipitation with the mixture of diethyl ether and dichloromethane (36 mL, V/V = 5:1, 3 cycles) afforded the title compound **22** (450 mg, 1.25 mmol, 45 %).

from the (E)-alkene:

Following **General procedure** with the following modifications: carried out on a 0.98 mmol scale (351 mg, 1 equiv.); sodium borohydride (13.0 mg, 0.343 mmol, 0.35 equiv.), 1.5 h reaction time at 0 °C. After 1 h the reaction was not completed, therefore additional sodium borohydride (5.6 mg, 0.15 mmol, 0.15 equiv.) was added in one portion to the reaction mixture. Purification by precipitation with the mixture of diethyl ether and dichloromethane (12 mL, V/V = 10:2, 3 cycles) afforded the title compound **22** (173 mg, 0.481 mmol, 49 %).

Physical State: white solid

^{1}H-NMR (499.64 MHz, $CDCl_3$): δ = 8.36 (dd, *J* = 7.9, 1.3 Hz, 1H), 8.32 (dd, *J* = 8.0, 1.3 Hz, 1H), 7.85 – 7.80 (m, 2H), 7.79 – 7.73 (m, 2H), 7.68 (t, *J* = 7.7 Hz, 2H), 4.36 – 4.26 (m, 1H), 1.88 – 1.77 (m, 1H), 1.63 – 1.52 (m, 1H), 1.33 (d, *J* = 6.8 Hz, 3H), 0.98 ppm (t, *J* = 7.4 Hz, 3H).(see Spectrum)

^{13}C-NMR (125.65 MHz, $CDCl_3$): δ = 135.8, 135.7, 134.5, 134.4, 130.0, 130.0, 129.9, 129.8, 128.7, 127.7, 117.41, 117.35, 52.9, 24.5, 14.6, 10.3 ppm. (see Spectrum)

^{19}F-NMR (282.21 MHz, $CDCl_3$): δ = -150.62 (s), -150.67 (s) ppm. (see Spectrum)

HRMS (ESI): M^+ calcd. for $[C_{16}H_{17}S_2]^+$ 273.0766, found 273.0762

3.2. Detailed optimization and practical guidance

For extended optimization data and a summary of useful knowledge that may be helpful in case of application to a new substrate, see below:

a) Reagent

Sodium borohydride proved to be the best hydride source to reduce alkenyl thianthren-5-ium salts. Using this reagent allows a simple water free work up, which is important since alkyl thianthren-5-ium salts are unstable under the conditions of most aqueous work ups.

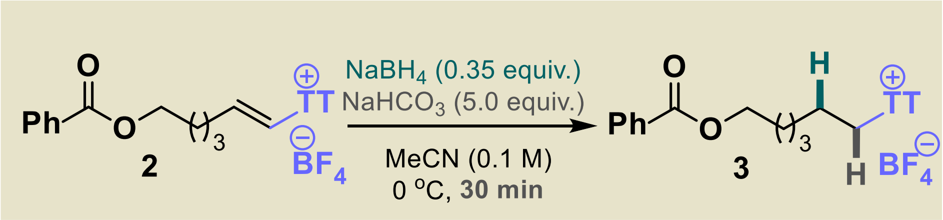

observed side products:

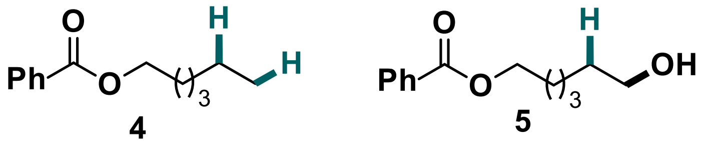

entry	reagent	effect	NMR yield (%)
1[a]	no deviation	none	75
2[b]	Pd/C, H_2 (atm)	some overreduction to alkane **4**	0
3[b]	$Mn(dpm)_3$, $PhSiH_3$	product unstable to isolation	n.d.
4	$NaBH_3CN$	low conversion	traces
5	$NaBH(OAc)_3$	low conversion	4
6	BH_3•Me_2S	low conversion	5

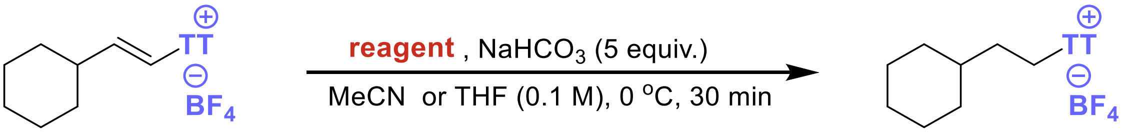

Representative effects of deviations

entry	reagent	effect	NMR yield (%)
1[b]	$LiAlH_4$	some overreduction to alkane	5
2	BH_3•THF	low conversion	6
3[b]	Super-Hydride	complex mixture	0
4[b]	L-Selectride	overreduction to alkane	traces
5[b]	9-BBN	low conversion	traces
6[b]	DIBAL-H	low conversion	10
7[b]	$LiBH_4$	low conversion	32

a, Dry MeCN was used, otherwise extensive formation of **5** was observed. **b**, No $NaHCO_3$ additive was used.

b) Reagent amount

Sodium borohydride serves as a quadruple hydride donor; however, it exhibits varying reactivity and different rates. Through practical observation, it has been noted that the initial three reductions take place within the reaction's time frame. Consequently, reducing the quantity of reagents, both in terms of excess and deficiency, leads to decreased yields.

	reagent amount	effect	NMR yield (%)
1	0.25 equiv.	slow conversion	33
2	0.5 equiv.	some overreduction to alkane	48

c) Additives

Consistent with our working hypothesis, the facilitated protonation of the ylide intermediate proves advantageous, leading to enhanced yields.

entry	additives	effect	NMR yield (%)
1	$KHCO_3$	full conversion	60
2	Na_2HPO_4	full conversion	67
3	$KHSO_4$	full conversion, sideproduct	31
4	nothing	full conversion, sideproduct	50

d) Reaction time

Empirical observations have revealed that overreduction resulting from an excess of reagent occurs gradually. Conversely, extended reaction times tend to lead to a gradual decline in yields.

	reaction time	effect	NMR yield (%)
1	2 h	some overreduction to alkane	50
2	15 h	enhanced overreduction to alkane	43

e) Solvent

Using anhydrous solvent may be beneficial but not crucial.

	solvent	effect	NMR yield (%)
1	technical MeCN	full conversion, sideproduct **5**	50
2	MeOH	full conversion, sideproduct	44

f) Concentration

Changes in concentration are well tolerated.

	concentration	effect	NMR yield (%)
1	0.05 M	full conversion	50
2	0.25 M	full conversion	45

g) Reaction with the triflate salt

Interestingly the counter-ion has a significant effect on the outcome of the reduction.

entry	Triflate (purified)	effect	NMR yield (%)
1	Triflate	full conversion, sideproduct	50

h) Work-up, isolation

It is noted, that if an aqueous work up is needed, the use of an acidic aqueous solution of HBF_4/$NaBF_4$ can be tolerated.

4. Reactions of alkyl thianthren-5-ium tetrafluoroborate salts

4.1. General procedure

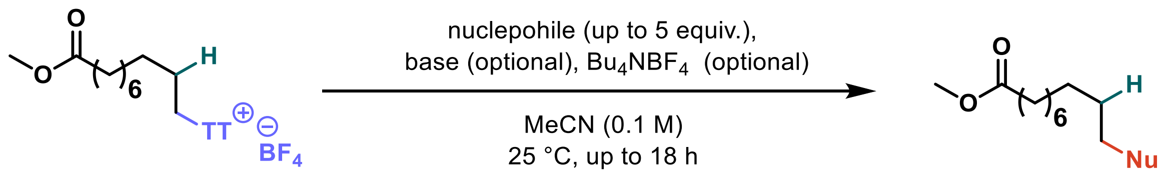

To the solution of the alkyl thianthren-5-ium salt [Note 1] (0.41-0.70 mmol, 1 equiv.) in anhydrous acetonitrile (0.1 M) were added the indicated nucleophile (5 equiv.), base (if indicated, 5.00 equiv.) and tetrabutylammonium tetrafluoroborate (if indicated, 0.10 equiv.) and the reaction mixture was stirred at 25-70 °C for 2.5-18 h. [Note 3] After analysis by TLC or LC-MS indicated the completion of the reaction, [Note 4] dichloromethane (50 ml) and saturated brine (50 ml) were added to the reaction mixture. The phases were separated, and the aqueous layer was extracted with dichloromethane (3x50 mL). The combined organic layers were dried using anhydrous Na_2SO_4, filtered, and concentrated under reduced pressure [Notes 5-7]. The residue was purified by flash column chromatography to afford the pure products.

[Note 1]: The use of oven dried glassware is recommended as alkyl thianthren-5-ium salts may be water sensitive.
[Note 2]: A heating block was used.
[Note 3]: The exact external temperature is noted in every case.
[Note 4]: The reaction can usually be monitored by TLC, however in some cases the products are not UV active, and a stain should be used. The reaction can also be followed by LC-MS.
[Note 5]: The temperature of the water bath was set to 25 °C
[Note 6]: In the cases, where an external base was not used (e.g. amine nucleophiles, phosphines), no aqueous work up was carried out and the crude reaction mixture was directly concentrated under reduced pressure.
[Note 7]: At this point, NMR yield was measured as follows: 1,3,5-trimethylbenzene was added as an internal standard to the residue and the mixture formed was fully dissolved in deuterated chloroform. ^{1}H NMR yield was then measured using samples from this solution.

Compound 23 (methyl 10-(phenylamino)decanoate)

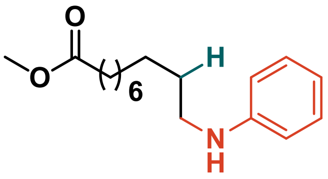

Following **General Procedure** with the following modifications: carried out on a 0.41 mmol scale using aniline (189 µl, 2.07 mmol, 5.00 equiv.) as the nucleophile in acetonitrile (4.1 mL, 0.1 M) for 18 h at 25 °C. The reaction mixture was directly concentrated under reduced pressure and no aqueous workup was carried out. Purification by flash column chromatography on flash silica gel (0% ethyl-acetate in hexanes grading to 25% ethyl-acetate in hexanes) afforded the title compound **23** (62 mg, 0.22 mmol, 54 %, 61% NMR yield).

Physical State: pale yellow solid

^{1}H-NMR (499.64 MHz, $CDCl_3$): δ = 7.19 – 7.14 (m, 2H), 6.68 (tt, J = 7.3, 1.1 Hz, 1H), 6.62 – 6.58 (m, 2H), 3.67 (s, 3H), 3.58 (s, 1H), 3.10 (t, J = 7.1 Hz, 2H), 2.30 (t, J = 7.5 Hz, 2H), 1.61 (p, J = 7.1 Hz, 4H), 1.39 ppm (t, J = 7.5 Hz, 2H), 1.36 – 1.31 (m, 8H). (see Spectrum)

^{13}C-NMR (125.65 MHz, $CDCl_3$): δ = 174.3, 148.5, 129.2, 117.1, 112.7, 51.4, 44.0, 34.1, 29.6, 29.3 (2C), 29.2, 29.1, 27.1, 24.9 ppm. (see Spectrum)

HRMS (ESI): $[M+H]^+$ calcd. for $[C_{17}H_{28}NO_2]^+$ 278.2115, found 278.2111

TLC: Rf = 0.43 (13 % ethyl-acetate in hexanes, CAM)

Compound 24 (methyl 10-(benzylamino)decanoate)

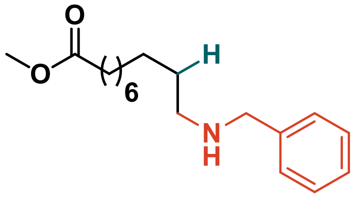

Following **General Procedure** with the following modifications: carried out on a 0.49 mmol scale using benzylamine (267 µl, 2.45 mmol, 5.00 equiv.) as the nucleophile in acetonitrile (4.9 mL, 0.1 M) for 18 h at 25 °C. The reaction mixture was directly concentrated under reduced pressure and no aqueous workup was carried out. Purification by flash column chromatography on flash alumina gel (0% methanol in dichloromethane grading to 10% methanol in dichloromethane) afforded the title compound **24** (80 mg, 0.27 mmol, 56 %, 58% NMR yield.)

Physical State: yellow oil

^{1}H-NMR (499.64 MHz, $CDCl_3$): δ = 7.35 – 7.30 (m, 4H), 7.28 – 7.23 (m, 1H), 3.94 (s, 1H), 3.81 (s, 2H), 3.66 (s, 3H), 2.67 – 2.60 (m, 2H), 2.29 (t, J = 7.5 Hz, 2H), 1.61 (p, J = 7.1 Hz, 2H), 1.53 (p, J = 7.2 Hz, 2H), 1.31 – 1.25 ppm (d, J = 7.1 Hz, 10H). (see Spectrum)

^{13}C-NMR (125.65 MHz, $CDCl_3$): δ = 174.2, 138.8, 128.5 (2C), 127.2, 53.3, 51.4, 48.7, 34.1, 29.34, 29.29, 29.2, 29.12, 29.07, 27.2, 24.9 ppm. (see Spectrum)

HRMS (ESI): $[M+H]^+$ calcd. for $[C_{18}H_{30}NO_2]^+$ 292.2271 , found 292.2270

TLC: Rf = 0.75 (3 % methanol in dichloromethane, iodine chamber, then o-toluidine)

Compound 25 (methyl 10-(benzyl(methyl)amino)decanoate)

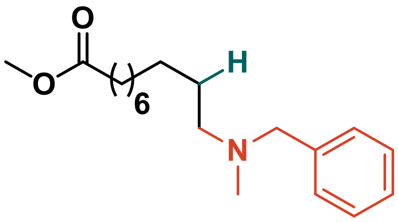

Following **General Procedure** with the following modifications: carried out on a 0.51 mmol scale using *N*-benzylmethylamine (330 µl, 2.54 mmol, 5.00 equiv.) as the nucleophile in acetonitrile (5.1 mL, 0.1 M) for 18 h at 25 °C. The reaction mixture was directly concentrated under reduced pressure and no aqueous workup was carried out. Purification by flash column chromatography on flash silica gel (0% acetone in hexanes grading to 25% acetone in hexanes) afforded the title compound **25** (126 mg, 0.412 mmol, 81 %, 83% NMR yield).

Physical State: yellow oil

^{1}H-NMR (499.64 MHz, $CDCl_3$): δ = 7.31 (d, J = 4.3 Hz, 4H), 7.23 (dt, J = 8.8, 4.3 Hz, 1H), 3.67 (s, 3H), 3.48 (s, 2H), 2.39 – 2.33 (m, 2H), 2.30 (t, J = 7.5 Hz, 2H), 2.18 (s, 3H), 1.66 – 1.56 (m, 2H), 1.50 (p, J = 7.1 Hz, 2H), 1.35 – 1.23 ppm (m, 10H). (see Spectrum)

^{13}C-NMR (125.65 MHz, $CDCl_3$): δ = 174.3, 138.8 (br s), 129.1, 128.2, 127.0, 62.2, 57.4, 51.4, 42.1, 34.1, 29.44, 29.36, 29.2, 29.1, 27.4, 27.2, 24.9 ppm. (see Spectrum)

HRMS (ESI): $[M+H]^+$ calcd. for $[C_{19}H_{32}NO_2]^+$ 306.2428, found 306.2425

TLC: Rf = 0.38 (16 % acetone in hexanes, iodine chamber, then o-toluidine)

Compound 26 (*N*,*N*,*N*-triethyl-10-methoxy-10-oxodecan-1-aminium tetrafluoroborate)

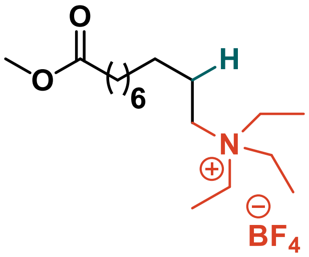

Following **General Procedure** with the following modifications: carried out on a 0.46 mmol scale using triethylamine (324 µl, 2.32 mmol, 5.00 equiv.) as the nucleophile in acetonitrile (4.6 mL, 0.1 M) for 2.5 h at 25 °C. The reaction mixture was directly concentrated under reduced pressure and no aqueous workup was carried out. Purification by precipitation with the

mixture of diethyl ether and dichloromethane (V/V = 5:1), and the residue was washed with diethyl ether (3 × 10 mL) afforded the title compound **26** (140 mg, 0.375 mmol, 81 %).

Physical State: pale brown solid

^{1}H-NMR (499.64 MHz, $CDCl_3$): δ = 3.66 (s, 3H), 3.32 (q, J = 7.3 Hz, 6H), 3.16 – 3.10 (m, 2H), 2.30 (t, J = 7.5 Hz, 2H), 1.71 – 1.58 (m, 4H), 1.44 – 1.22 ppm (m, 19H). (see Spectrum)

^{13}C-NMR (125.65 MHz, $CDCl_3$): δ = 174.2, 57.0, 52.9, 51.3, 33.9, 29.0, 28.90, 28.88, 28.86, 26.2, 24.8, 21.6, 7.3 ppm (see Spectrum)

^{19}F-NMR (282.21 MHz, $CDCl_3$): δ = -152.19 (s), -152.24 ppm (s). (see Spectrum)

HRMS (ESI): M^+ calcd. for $[C_{17}H_{36}NO_2]^+$ 286.2741, found 286.2741

TLC: Rf = 0.60 (10 % methanol in dichloromethane, CAM)

Compound 27 (methyl 10-(1,3-dioxoisoindolin-2-yl)decanoate)

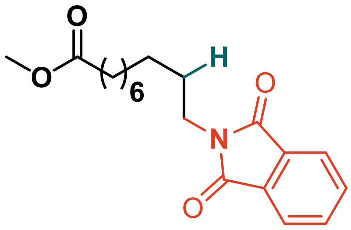

Following **General Procedure** with the following modifications: carried out on a 0.49 mmol scale using potassium phthalimide (465 mg, 2.46 mmol, 5.00 equiv.) as the nucleophile and tetrabutylammonium tetrafluoroborate (16 mg, 0.049 mmol, 0.10 equiv.) as additive in acetonitrile (4.9 mL, 0.1 M) for 18 h at 25 °C. Purification by flash column chromatography on flash silica gel (0% ethyl-acetate in hexanes grading to 20 % ethyl-acetate in hexanes) afforded the title compound **27** (114 mg, 0.344 mmol, 70 %, 79 % NMR yield)

Physical State: pale yellow solid

^{1}H-NMR (499.64 MHz, $CDCl_3$): δ = 7.81 (dd, J = 5.4, 3.0 Hz, 2H), 7.68 (dd, J = 5.5, 3.0 Hz, 2H), 3.68 – 3.62 (m, 5H), 2.27 (t, J = 7.5 Hz, 2H), 1.65 (q, J = 7.4 Hz, 2H), 1.57 (p, J = 7.3 Hz, 2H), 1.34 – 1.22 ppm (m, 10H). (see Spectrum)

^{13}C-NMR (125.65 MHz, $CDCl_3$): δ = 174.2, 168.4, 133.7, 132.2, 123.1, 51.3, 38.0, 34.0, 29.2, 29.1, 29.0 (2C), 28.5, 26.7, 24.9 ppm. (see Spectrum)

HRMS (ESI): $[M+H]^+$ calcd. for $[C_{19}H_{26}NO_4]^+$ 332.1856, found 332.1858

TLC: Rf = 0.69 (25 % ethyl-acetate in hexanes , $KMnO_4$)

Compound 28 (methyl 10-(1,3-dimethyl-2,6-dioxo-1,2,3,6-tetrahydro-7*H*-purin-7-yl)decanoate)

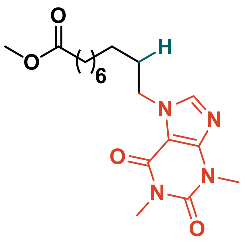

Following **General Procedure** with the following modifications: carried out on a 0.61 mmol scale using theophylline (110 mg, 0.609 mmol, 1.00 equiv.) as the nucleophile and potassium carbonate (421 mg, 3.05 mmol, 5.00 equiv.) as the base in acetonitrile (6.1 mL, 0.1 M) for 18 h at 50 °C. Purification by flash column chromatography on flash silica gel (0% methanol in dichloromethane grading to 10% methanol in dichloromethane) afforded the title compound **28** (184 mg, 0.505 mmol, 83 %).

Physical State: brown solid

^{1}H-NMR (599.63 MHz, $CDCl_3$): δ = 7.52 (s, 1H), 4.26 (t, J = 7.2 Hz, 2H), 3.65 (s, 3H), 3.58 (s, 3H), 3.40 (s, 3H), 2.28 (t, J = 7.5 Hz, 2H), 1.85 (p, J = 7.2 Hz, 2H), 1.60 (q, J = 7.1 Hz, 2H), 1.35 – 1.21 ppm (m, 10H). (see Spectrum)

^{13}C-NMR (150.79 MHz, $CDCl_3$): δ = 174.2, 155.1, 151.7, 148.9, 140.7, 106.9, 51.4, 47.3, 34.0, 30.8, 29.7, 29.2, 29.05, 29.01, 28.9, 28.0, 26.3, 24.8 ppm. (see Spectrum)

^{15}N-NMR (40.52 MHz, $CDCl_3$): δ = -151.1, -211.9, -229.8, -266.8 ppm. (see Spectrum)

HRMS (ESI): $[M+Na]^+$ calcd. for $[C_{18}H_{28}N_4O_4Na]^+$ 387.2003, found 387.1994

TLC: Rf = 0.82 (5 % methanol in dichloromethane, $KMnO_4$)

Compound 29 (methyl 10-azidodecanoate)

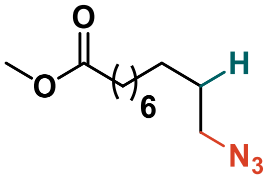

Following **General Procedure** with the following modifications: carried out on a 0.42 mmol scale using sodium azide (135 mg, 2.08 mmol, 5.00 equiv.) as the nucleophile and tetrabutylammonium tetrafluoroborate (20.6 mg, 62.5 µmol, 0.15 equiv.) as additive in acetonitrile (4.2 mL, 0.1 M) for 18 h at 25 °C. Purification by flash column chromatography on flash silica gel (0% ethyl-acetate in hexanes grading to 20 % ethyl-acetate in hexanes) afforded the title compound **29** (62.0 mg, 273 µmol, 65 %, 71% NMR yield).

Physical State: colorless oil

^{1}H-NMR (499.64 MHz, $CDCl_3$): δ = 3.66 (s, 3H), 3.25 (t, J = 7.0 Hz, 2H), 2.30 (t, J = 7.5 Hz, 2H), 1.66 – 1.55 (m, 4H), 1.40 – 1.27 ppm (m, 10H). (see Spectrum)

^{13}C-NMR (125.65 MHz, $CDCl_3$): δ = 174.2, 51.5, 51.4, 34.1, 29.2, 29.10, 29.06, 29.0, 28.8, 26.7, 24.9 ppm. (see Spectrum)

HRMS (ESI): $[M\text{-}N_2\text{+}H]^+$ calcd. for $[C_{11}H_{21}NO_2]^+$ 200.1606 , found 200.1640 $[M\text{+}H\ –N_2]^+$

TLC: Rf = 0.54 (13 % ethyl-acetate in hexanes , $KMnO_4$)

Compound 30 (methyl 10-acetoxydecanoate)

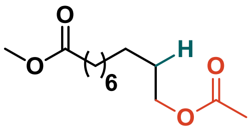

Following **General Procedure** with the following modifications: carried out on a 0.57 mmol scale using potassium acetate (282 mg, 2.87 mmol, 5.00 equiv.) as the nucleophile and tetrabutylammonium tetrafluoroborate (19 mg, 0.057 mmol, 0.10 equiv.) as additive in acetonitrile (5.7 mL, 0.1 M) for 18 h at 25 °C. Purification by flash column chromatography on flash silica gel (0% ethyl-acetate in hexanes grading to 20 % ethyl-acetate in hexanes) afforded the title compound **30** (90 mg, 0.37 mmol, 64 %, 67 % NMR yield).

Physical State: colorless oil

^{1}H-NMR (499.64 MHz, $CDCl_3$): δ = 4.03 (t, J = 6.8 Hz, 2H), 3.64 (s, 3H), 2.28 (t, J = 7.5 Hz, 2H), 2.02 (s, 3H), 1.64 – 1.53 (m, 4H), 1.35 – 1.24 ppm (m, 10H). (see Spectrum)

^{13}C-NMR (125.65 MHz, $CDCl_3$): δ = 174.2, 171.1, 64.5, 51.3, 34.0, 29.2, 29.10, 29.08, 29.0, 28.5, 25.8, 24.9, 20.9 ppm. (see Spectrum)

HRMS (ESI): $[M\text{+}H]^+$ calcd. for $[C_{13}H_{25}O_4]^+$ 245.1747, found 245.1745

TLC: Rf = 0.38 (13 % ethyl-acetate in hexanes, $KMnO_4$)

Compound 31 (methyl 10-phenoxydecanoate)

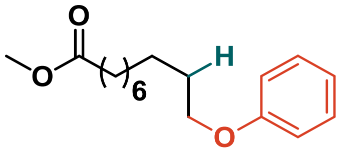

Following **General Procedure** with the following modifications: carried out on a 0.60 mmol scale using phenol (283 mg, 3.01 mmol, 5.00 equiv.) as the nucleophile and sodium carbonate (319 mg, 3.01 mmol, 5.00 equiv.) as the base in acetonitrile (6.0 mL, 0.1 M) for 18 h at 70 °C. Purification by flash column chromatography on flash silica gel (0% ethyl-acetate in hexanes

grading to 20 % ethyl-acetate in hexanes) afforded the title compound **31** (79 mg, 0.28 mmol, 47 %, 64 % NMR yield).

Physical State: colorless oil

^{1}H-NMR (499.64 MHz, $CDCl_3$): δ = 7.30 – 7.25 (m, 2H), 6.95 – 6.88 (m, 3H), 3.95 (t, J = 6.6 Hz, 2H), 3.67 (s, 3H), 2.31 (t, J = 7.5 Hz, 2H), 1.82 – 1.73 (m, 2H), 1.63 (p, J = 7.1 Hz, 2H), 1.45 (td, J = 8.8, 4.8 Hz, 2H), 1.32 ppm (q, J = 4.3 Hz, 8H). (see Spectrum)

^{13}C-NMR (125.65 MHz, $CDCl_3$): δ = 174.2, 159.1, 129.3, 120.4, 114.5, 67.8, 51.4, 34.1, 29.31, 29.27, 29.26, 29.14, 29.09, 26.0, 24.9 ppm. (see Spectrum)

HRMS (ESI): $[M+H]^+$ calcd. for $[C_{17}H_{27}O_3]^+$ 279.1955, found 279.1949

TLC: Rf = 0.52 (10 % ethyl-acetate in hexanes , $KMnO_4$)

Compound 32 ((10-methoxy-10-oxodecyl)triphenylphosphonium tetrafluoroborate)

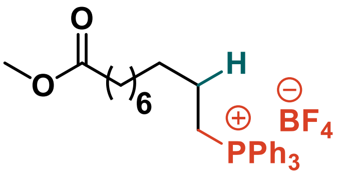

Following **General Procedure** with the following modifications: carried out on a 0.50 mmol scale using triphenylphosphine (393 mg, 1.50 mmol, 3.00 equiv.) as the nucleophile in acetonitrile (5.0 mL, 0.1 M) for 6 h at 70 °C. The reaction mixture was directly concentrated under reduced pressure and no aqueous workup was carried out. Purification by precipitation with the mixture of diethyl-ether and dichloromethane (V/V = 20:1, 10 mL) afforded the title compound **32** (220 mg, 0.412 mmol, 82%).

Physical State: pale brown solid

^{1}H-NMR (499.64 MHz, $CDCl_3$): δ = 7.79 (tt, J = 5.7, 2.8 Hz, 3H), 7.73 – 7.67 (m, 12H), 3.63 (s, 3H), 3.28 – 3.19 (m, 2H), 2.26 (t, J = 7.5 Hz, 2H), 1.64 – 1.50 (m, 4H), 1.29 – 1.16 (m, 10H) ppm. (see Spectrum)

^{13}C-NMR (125.65 MHz, $CDCl_3$): δ = 174.3, 135.1 (d, J = 2.9 Hz), 133.4 (d, J = 10.0 Hz), 130.5 (d, J = 12.7 Hz), 118.1 (d, J = 85.9 Hz), 51.4, 34.0, 30.2 (d, J = 15.8 Hz), 29.0, 28.91, 28.88, 28.8, 24.8, 22.5 (d, J = 4.5 Hz), 22.0 ppm (d, J = 50.9 Hz). (see Spectrum)

^{19}F-NMR (282.21 MHz, $CDCl_3$): δ = -152.30 (s), -152.35 ppm (s). (see Spectrum)

^{31}P-NMR (202.27 MHz, $CDCl_3$): δ = 23.7 ppm (s). (see Spectrum)

HRMS (ESI): M^+ calcd. for $[C_{29}H_{36}O_2P]^+$ 447.2447, found 447.2428

TLC: Rf = 0.71 (5 % methanol in dichloromethane, CAM)

Compound 33 (methyl 10-(phenylthio)decanoate)

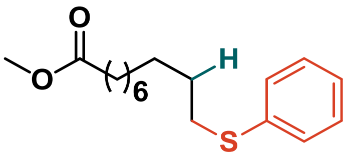

Following **General Procedure** with the following modifications: carried out on a 0.50 mmol scale using thiophenol (258 µl, 2.51 mmol, 5.00 equiv.) as the nucleophile and sodium bicarbonate (211 mg, 2.51 mmol, 5.00 equiv.) as the base in acetonitrile (5.0 mL, 0.1 M) for 18 h at 25 °C. Purification by flash column chromatography on flash silica gel (0% ethyl-acetate in hexanes grading to 5 % ethyl-acetate in hexanes) afforded the title compound **33** (95 mg, 0.32 mmol, 64 %, 68 % NMR yield).

Physical State: colorless oil

^{1}H-NMR (499.64 MHz, $CDCl_3$): δ = 7.34 – 7.30 (m, 2H), 7.29 – 7.25 (m, 2H), 7.15 (ddt, J = 8.6, 7.7, 1.3 Hz, 1H), 3.66 (s, 3H), 2.95 – 2.87 (m, 2H), 2.30 (t, J = 7.5 Hz, 2H), 1.69 – 1.56 (m, 4H), 1.41 (p, J = 7.0 Hz, 2H), 1.35 – 1.24 ppm (m, 8H). (see Spectrum)

^{13}C-NMR (125.65 MHz, $CDCl_3$): δ = 174.2, 137.0, 128.9, 128.8, 125.6, 51.4, 34.1, 33.6, 29.2, 29.11 (2C), 29.07, 29.0, 28.7, 24.9 ppm. (see Spectrum)

HRMS (ESI): $[M+H]^+$ calcd. for $[C_{17}H_{27}O_2S]^+$ 295.1726, found 295.1721

TLC: Rf = 0.43 (10 % ethyl-acetate in hexanes , $KMnO_4$)

Compound 34 (methyl 10-thiocyanatodecanoate)

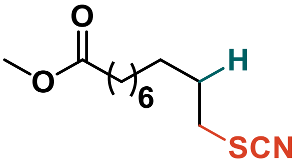

Following **General Procedure** with the following modifications: carried out on a 0.50 mmol scale using potassium thiocyanate (243 mg, 2.50 mmol, 5.00 equiv.) as the nucleophile in acetonitrile (5.0 mL, 0.1 M) for 18 h at 25 °C. Purification by flash column chromatography on flash silica gel (0% ethyl-acetate in hexanes grading to 15 % ethyl-acetate in hexanes) afforded the title compound **34** (95 mg, 0.39 mmol, 78 %).

Physical State: colorless oil

^{1}H-NMR (499.64 MHz, $CDCl_3$): δ = 3.65 (s, 3H), 2.93 (t, J = 7.3 Hz, 2H), 2.29 (t, J = 7.5 Hz, 2H), 1.81 (p, J = 7.3 Hz, 2H), 1.61 (p, J = 7.3 Hz, 2H), 1.42 (dd, J = 9.4, 5.6 Hz, 2H), 1.35 – 1.27 ppm (m, 8H). (see Spectrum)

^{13}C-NMR (125.65 MHz, $CDCl_3$): δ = 174.2, 112.3, 51.4, 34.02, 34.00, 29.8, 29.1, 29.02, 28.98, 28.7, 27.9, 24.8 ppm. (see Spectrum)

HRMS (ESI): $[M+H]^+$ calcd. for $[C_{12}H_{22}NO_2S]^+$ 244.1366, found 244.1377

TLC: Rf = 0.26 (13 % ethyl-acetate in hexanes, $KMnO_4$)

Compound 35 (methyl 10-cyanodecanoate)

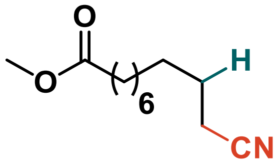

Following **General Procedure** with the following modifications: carried out on a 0.58 mmol scale using potassium cyanide (190 mg, 2.92 mmol, 5.00 equiv.) as the nucleophile and tetrabutylammonium tetrafluoroborate (19 mg, 0.058 mmol, 0.10 equiv.) as additive in acetonitrile (5.8 mL, 0.1 M) for 18 h at 25 °C. After analysis by TLC or LC-MS indicated the completion of the reaction, a modified aqueous workup was carried out as follows: dichloromethane (50 mL) and saturated sodium bicarbonate solution (50 mL) were added to the reaction mixture. The phases were separated and the organic layer was washed with saturated sodium bicarbonate solution (4x50 mL). Purification by flash column chromatography on flash silica gel (0% ethyl-acetate in hexanes grading to 20 % ethyl-acetate in hexanes) afforded the title compound **35** (65 mg, 0.31 mmol, 53 %, 67% NMR yield).

Physical State: colorless oil

^{1}H-NMR (499.64 MHz, $CDCl_3$): δ = 3.63 (s, 3H), 2.29 (dt, J = 16.1, 7.4 Hz, 4H), 1.61 (ddt, J = 16.6, 14.5, 7.2 Hz, 4H), 1.45 – 1.37 (m, 2H), 1.33 – 1.24 ppm (m, 8H). (see Spectrum)

^{13}C-NMR (125.65 MHz, $CDCl_3$): δ = 174.1, 119.7, 51.3, 33.9, 29.0 (2C), 28.9, 28.6, 28.5, 25.3, 24.8, 17.0 ppm. (see Spectrum)

HRMS (ESI): $[M+Na]^+$ calcd. for $[C_{12}H_{21}NO_2Na]^+$ 234.1465, found 234.1459

TLC: Rf = 0.21 (10 % ethyl-acetate in hexanes, $KMnO_4$)

Compound 36 (methyl 10-fluorodecanoate)

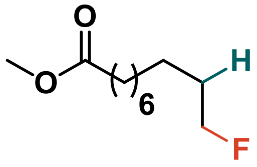

Following **General Procedure** with the following modifications: carried out on a 0.47 mmol scale using tetrabutylammonium fluoride in tetrahydrofuran solution (1.0 M, 2.32 mmol, 2.32 mL, 5.00 equiv.) as the nucleophile in acetonitrile (4.7 mL, 0.1 M) for 18 h at 25 °C. Purification by flash column chromatography on flash silica gel (0% ethyl-acetate in hexanes

grading to 20 % ethyl-acetate in hexanes) afforded the title compound **36** (36 mg, 0.18 mmol, 38 %, 49% NMR yield). *Note*: The lower yield observed was a result of an increased formation of the side product alkene through elimination.

Physical State: colorless oil

^{1}H-NMR (499.64 MHz, $CDCl_3$): δ = 4.42 (dt, J = 47.4, 6.2 Hz, 2H), 3.66 (s, 3H), 2.30 (t, J = 7.6 Hz, 2H), 1.74 – 1.57 (m, 4H), 1.42 – 1.34 (m, 2H), 1.34 – 1.26 ppm (m, 8H). (see Spectrum)

^{13}C-NMR (125.65 MHz, $CDCl_3$): δ = 174.2, 84.2 (d, J = 164.0 Hz), 51.4, 34.1, 30.4 (d, J = 19.6 Hz), 29.3, 29.13, 29.10, 29.07, 25.1 (d, J = 5.4 Hz), 24.9 ppm. (see Spectrum)

^{19}F-NMR (282.21 MHz, $CDCl_3$): δ = -218.08 ppm (tt, J = 47.3, 24.9 Hz). (see Spectrum)

HRMS (ESI): $[M+Na]^+$ calcd. for $[C_{11}H_{21}FO_2Na]^+$ 227.1418, found 227.1413

TLC: Rf = 0.46 (13 % ethyl-acetate in hexanes, $KMnO_4$)

Compound 37 (methyl 10-bromodecanoate)

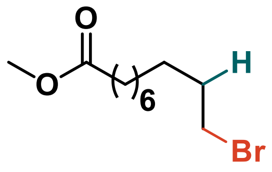

Following **General Procedure** with the following modifications: carried out on a 0.44 mmol scale using tetrabutylammonium bromide (705 mg, 2.19 mmol, 5.00 equiv.) as the nucleophile in acetonitrile (4.4 mL, 0.1 M) for 18 h at 25 °C. Purification by flash column chromatography on flash silica gel (0% ethyl-acetate in hexanes grading to 20 % ethyl-acetate in hexanes) afforded the title compound **37** (69 mg, 0.18 mmol, 59 %, 65% NMR yield).

Physical State: colorless oil

^{1}H-NMR (499.64 MHz, $CDCl_3$): δ = 3.64 (s, 3H), 3.38 (t, J = 6.9 Hz, 2H), 2.28 (t, J = 7.5 Hz, 2H), 1.83 (dt, J = 14.5, 7.0 Hz, 2H), 1.60 (p, J = 7.1 Hz, 2H), 1.39 (q, J = 7.1 Hz, 2H), 1.33 – 1.24 ppm (m, 8H). (see Spectrum)

^{13}C-NMR (125.65 MHz, $CDCl_3$): δ = 174.1, 51.3, 34.0, 33.8, 32.7, 29.14, 29.06, 29.0, 28.6, 28.1, 24.9 ppm. (see Spectrum)

HRMS (ESI): $[M+H]^+$ calcd. for $[C_{11}H_{22}BrO_2]^+$ 265.0798, found 265.0793

TLC: Rf = 0.60 (10 % ethyl-acetate in hexanes, $KMnO_4$)

Compound 38 (methyl 10-(pyridin-2-yloxy)decanoate)

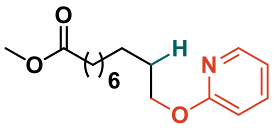

Following **General Procedure** with the following modifications: carried out on a 0.59 mmol scale using 2-pyridone (281 mg, 2.95 mmol, 5.00 equiv.) as the nucleophile and sodium bicarbonate (248 mg, 2.95 mmol, 5.00 equiv.) as the base in acetonitrile (5.9 mL, 0.1 M) for 18 h at 70 °C. Purification by flash column chromatography on flash silica gel (0% ethyl-acetate in hexanes grading to 25 % ethyl-acetate in hexanes) afforded the title compound **38** (86 mg, 0.31 mmol, 52 %, 55 % NMR yield,).

Physical State: yellow oil

^{1}H-NMR (499.64 MHz, $CDCl_3$): δ = 8.13 (ddd, J = 5.2, 2.0, 0.8 Hz, 1H), 7.54 (ddd, J = 8.8, 7.1, 2.0 Hz, 1H), 6.82 (ddd, J = 7.1, 5.0, 1.0 Hz, 1H), 6.71 (dt, J = 8.3, 0.9 Hz, 1H), 4.26 (t, J = 6.7 Hz, 2H), 3.65 (s, 3H), 2.29 (t, J = 7.5 Hz, 2H), 1.80 – 1.72 (m, 2H), 1.61 (p, J = 7.2 Hz, 2H), 1.43 (td, J = 8.6, 4.8 Hz, 2H), 1.37 – 1.28 ppm (m, 8H). (see Spectrum)

^{13}C-NMR (125.65 MHz, $CDCl_3$): δ = 174.2, 164.1, 146.9, 138.4, 116.4, 111.0, 65.9, 51.4, 34.1, 29.33, 29.29, 29.15, 29.10, 29.0, 26.0, 24.9 ppm. (see Spectrum)

^{15}N-NMR (50.63 MHz, $CDCl_3$): δ = -114.4 ppm

HRMS (ESI):): $[M+H]^+$ calcd. for $[C_{16}H_{26}NO_3]^+$ 280.1907, found 280.1898

TLC: Rf = 0.33 (13 % ethyl-acetate in hexanes, $KMnO_4$)

Compound 39 (methyl 10-(pyridin-2-ylthio)decanoate)

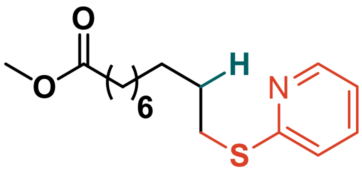

Following **General Procedure** with the following modifications: carried out on a 0.65 mmol scale using 2-mercaptopyridine (364 mg, 3.27 mmol, 5.00 equiv.) as the nucleophile and sodium bicarbonate (275 mg, 3.27 mmol, 5.00 equiv.) as the base in acetonitrile (6.5 mL, 0.1 M) for 18 h at 25 °C. Purification by flash column chromatography on flash silica gel (0% ethyl-acetate in hexanes grading to 25 % ethyl-acetate in hexanes) afforded the title compound **39** (115 mg, 0.388 mmol, 59 %, 63 % NMR yield).

Physical State: colorless oil

^{1}**H-NMR** (599.63 MHz, $CDCl_3$): δ = 8.41 (dd, J = 4.2, 2.1 Hz, 1H), 7.46 (td, J = 7.7, 1.9 Hz, 1H), 7.16 (d, J = 8.1 Hz, 1H), 6.96 (dd, J = 7.3, 4.9 Hz, 1H), 3.66 (s, 3H), 3.15 (t, J = 7.4 Hz, 2H), 2.29 (t, J = 7.5 Hz, 2H), 1.69 (p, J = 7.4 Hz, 2H), 1.61 (p, J = 7.0 Hz, 2H), 1.43 (p, J = 7.0 Hz, 2H), 1.35 – 1.27 ppm (m, 8H). (see Spectrum)

^{13}**C-NMR** (150.79 MHz, $CDCl_3$): δ = 174.3, 159.6, 149.3, 135.9, 122.2, 119.2, 51.4, 34.1, 30.1, 29.27, 29.26, 29.15, 29.09, 28.9, 24.9 ppm. (see Spectrum)

^{15}**N-NMR** (40.52 MHz, $CDCl_3$): δ = -81.9 ppm

HRMS (ESI): $[M+H]^+$ calcd. for $[C_{16}H_{26}NO_2S]^+$ 296.1679, found 296.1671

TLC: Rf = 0.71 (20 % ethyl-acetate in hexanes, $KMnO_4$)

Compound 40a and 40b

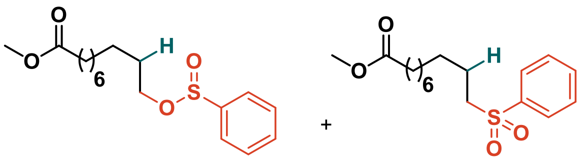

40a **40b**

1:1

Following **General Procedure** with the following modifications: carried out on a 0.70 mmol scale using benzenesulfinic acid sodium salt (582 mg, 3.48 mmol, 5.00 equiv.) as the nucleophile and tetrabutylammonium tetrafluoroborate (23 mg, 0.070 mmol, 0.10 equiv.) additive in acetonitrile (7.0 mL, 0.1 M) for 18 h at 70 °C. Purification by flash column chromatography on flash silica gel (0% ethyl-acetate in hexanes grading to 20 % ethyl-acetate in hexanes) afforded the title compound **40a** and **40b** (155 mg, 0.475 mmol, 68 %, 70 % NMR yield 40a:40b ratio = 1:1).

Physical State: colorless oil

40a

^{1}**H-NMR** (399.87 MHz, $CDCl_3$): δ = 7.73 – 7.68 (m, 2H), 7.54 (dd, J = 5.1, 2.0 Hz, 3H), 4.03 (dt, J = 9.9, 6.7 Hz, 1H), 3.66 (s, 3H), 3.60 (dt, J = 9.9, 6.6 Hz, 1H), 2.29 (t, J = 7.5 Hz, 2H), 1.65 – 1.57 (m, 4H), 1.34 – 1.21 ppm (m, 10H). (see Spectrum)

^{13}**C-NMR** (100.56 MHz, $CDCl_3$): δ = 174.3, 144.8, 132.0, 129.0, 125.2, 64.8, 51.4, 34.1, 29.6, 29.2, 29.09, 29.06, 29.00, 25.6, 24.9 ppm. (see Spectrum)

HRMS (ESI): $[M+H]^+$ calcd. for $[C_{17}H_{27}O_4S]^+$ 327.1625, found 327.1625

40b

^{1}**H-NMR** (499.64 MHz, $CDCl_3$): δ = 7.93 – 7.88 (m, 2H), 7.68 – 7.62 (m, 1H), 7.59 – 7.53 (m, 2H), 3.65 (s, 3H), 3.11 – 3.02 (m, 2H), 2.27 (t, J = 7.5 Hz, 2H), 1.69 (tt, J = 7.9, 6.5 Hz, 2H), 1.58 (p, J = 7.4 Hz, 2H), 1.33 (t, J = 7.4 Hz, 2H), 1.30 – 1.20 ppm (m, 8H). (see Spectrum)

^{13}**C-NMR** (125.65 MHz, $CDCl_3$): δ = 174.1, 139.2, 133.5, 129.1, 127.9, 56.2, 51.3, 33.9, 28.92, 28.88, 28.87, 28.8, 28.1, 24.8, 22.5 ppm. (see Spectrum)

HRMS (ESI): $[M+H]^+$ calcd. for $[C_{17}H_{27}O_4S]^+$ 327.1625, found 327.1632

TLC: 40a: Rf = 0.15 (13 % ethyl-acetate in hexanes, $KMnO_4$)

40b: Rf = 0.33 (25 % ethyl-acetate in hexanes, $KMnO_4$)

Compound 41 (methyl 10-oxodecanoate)

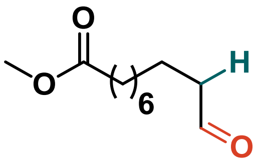

Following **General Procedure** with the following modifications: carried out on a 0.50 mmol scale using 4-Methylmorpholine *N*-oxide (293 mg, 2.50 mmol, 5.00 equiv.) as the nucleophile and sodium bicarbonate (210 mg, 2.50 mmol, 5.00 equiv.) as the base in acetonitrile (5.0 mL, 0.1 M) for 18 h at 50 °C. Purification by flash column chromatography on flash silica gel (0% ethyl-acetate in hexanes grading to 20% ethyl-acetate in hexanes) afforded the title compound **41** (51 mg, 0.25 mmol, 51%, 69% NMR yield).

Physical State: colorless oil

^{1}H-NMR (499.64 MHz, $CDCl_3$): δ = 9.74 (t, J = 1.9 Hz, 1H), 3.64 (s, 3H), 2.39 (td, J = 7.3, 1.8 Hz, 2H), 2.28 (t, J = 7.5 Hz, 2H), 1.63 – 1.58 (m, 4H), 1.32 – 1.29 ppm (m, 8H). (see Spectrum)

^{13}C-NMR (125.65 MHz, $CDCl_3$): δ = 202.7, 174.2, 51.4, 43.8, 34.0, 29.1, 29.02, 28.98, 28.96, 24.8, 22.0 ppm. (see Spectrum)

HRMS (ESI): $[M+H]^+$ calcd. for $[C_{11}H_{21}O_3]^+$ 201.1485, found 201.1481

TLC: Rf = 0.23 (20 % ethyl-acetate in hexanes, $KMnO_4$)

Compound 47 (methyl decanoate-10-*d*)

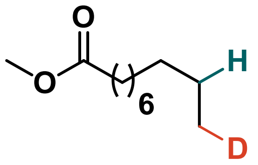

Following **General Procedure** with the following modifications: carried out on a 0.50 mmol scale using sodium tetrahydroborate-d4 (20.9 mg, 0.50 mmol, 1.00 equiv.) as the nucleophile for 6 h at 25 °C. Purification by flash column chromatography on flash silica gel (0% ethyl-acetate in hexanes grading to 20% ethyl-acetate in hexanes) afforded the title compound **47** (59.0 mg, 0.315 mmol, 63%, 79 % NMR yield).

Physical State: colorless oil

^{1}H-NMR (499.64 MHz, $CDCl_3$): δ = 3.65 (s, 3H), 2.29 (t, J = 7.5 Hz, 2H), 1.65 – 1.55 (m, 2H), 1.34 – 1.19 (m, 12H), 0.89 – 0.81 ppm (m, 2H). (see Spectrum)

^{13}C-NMR (125.65 MHz, $CDCl_3$): δ = 174.3, 51.3, 34.1, 31.8, 29.4, 29.2 (2C), 29.1, 25.0, 22.5, 13.74 (t, J = 19.1 Hz) ppm. (see Spectrum)

HRMS (ESI): $[M+H]^+$ calcd. for $[C_{11}H_{22}DO_2]^+$ 188.1755, found 188.1751

TLC: Rf = 0.55 (5 % ethyl-acetate in hexanes, $KMnO_4$)

4.2. Specific procedures

Compound 46 (methyl decanoate)

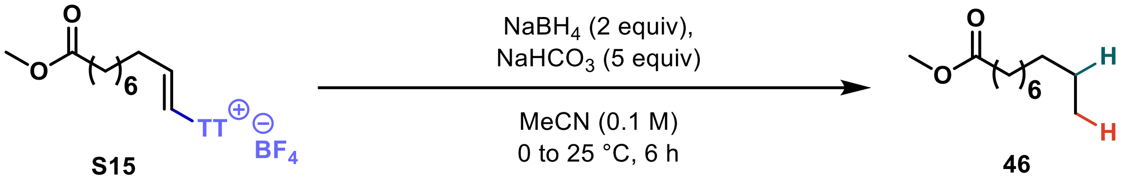

A suspension of the **S15** alkenyl thianthren-5-ium salt (243 mg, 0.500 mmol, 1 equiv.) and sodium bicarbonate (210 mg, 2.50 mmol, 5.00 equiv.) in anhydrous acetonitrile (5.0 mL, 0.1 M) were cooled to 0 °C under argon atmosphere. Then sodium borohydride (37.8 mg, 1.00 mmol mmol, 2.00 equiv.) was added in one portion at this temperature. After stirring the reaction mixture for 30 min, the mixture was warmed to 25 °C and stirred at this temperature for an additional 6 h, when analysis by LC-MS indicated full conversion. Then, saturated aqueous sodium bicarbonate solution (30 ml) and diethyl ether (30 mL) were added to the reaction mixture. The phases were separated and the aqueous layer was extracted with diethyl ether (30 mL). The combined organic layers were dried with anhydrous Na_2SO_4, filtered, and concentrated under reduced pressure. The residue was purified by flash column chromatography on silica gel (0% ethyl-acetate in hexanes grading to 20% ethyl-acetate in hexanes) to afford the pure product **46** (56.3 mg, 0.302 mmol, 60%, 64% NMR yield).

Physical State: colorless oil

^{1}H-NMR (499.64 MHz, $CDCl_3$): δ = 3.65 (s, 3H), 2.29 (t, J = 7.6 Hz, 2H), 1.61 (p, J = 7.4 Hz, 2H), 1.33 – 1.18 (m, 12H), 0.87 ppm (t, J = 6.9 Hz, 3H). (see Spectrum)

^{13}C-NMR (125.65 MHz, $CDCl_3$): δ = 174.3, 51.3, 34.1, 31.8, 29.4, 29.23, 29.22, 29.1, 24.9, 22.6, 14.0 ppm. (see Spectrum)

HRMS (ESI): $[M+H]^+$ calcd. for $[C_{11}H_{23}O_2]^+$ 187.1693, found 187.1689

TLC: Rf = 0.55 (5 % ethyl-acetate in hexanes, $KMnO_4$)

Compound 48 (methyl decanoate-9,10-d_2)

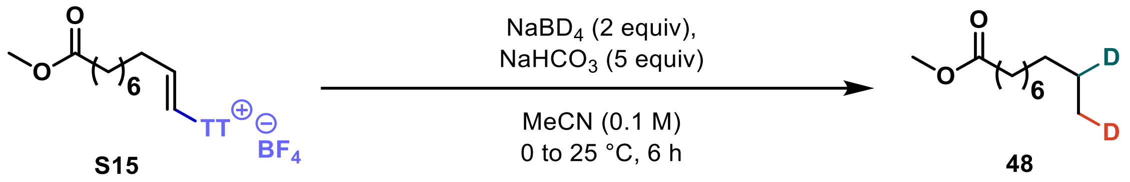

A suspension of the **S15** alkenyl thianthren-5-ium salt (304 mg, 0.626 mmol, 1 equiv.) and sodium bicarbonate (263 mg, 3.13 mmol, 5.00 equiv.) in anhydrous acetonitrile (5.5 mL, 0.1 M) were cooled to 0 °C under argon atmosphere. Then sodium borohydride-d4 (52.4 mg, 1.25 mmol, 2.00 equiv.) was added in one portion at this temperature. After stirring the reaction mixture for 30 min, the mixture was warmed to 25 °C and stirred at this temperature for an additional 6 h, when analysis by LCMS indicated full conversion. Then, saturated aqueous sodium bicarbonate solution (30 ml) and diethyl ether (30 mL) were added to the reaction mixture. The phases were separated and the aqueous layer was extracted with diethyl ether (30 mL). The combined organic layers were dried with anhydrous Na_2SO_4, filtered, and concentrated under reduced pressure. The residue was purified by flash column chromatography on silica gel (0% ethyl-acetate in hexanes grading to 20% ethyl-acetate in hexanes) to afford the pure product **48** (79 mg, 0.42 mmol, 67%, 87% NMR yield).

Physical State: colorless oil

^{1}H-NMR (499.64 MHz, $CDCl_3$): δ = 3.67 (s, 3H), 2.30 (t, J = 7.6 Hz, 2H), 1.62 (h, J = 6.4 Hz, 2H), 1.36 – 1.18 (m, 11H), 0.85 ppm (dt, J = 4.8, 2.4 Hz, 2H). (see Spectrum)

^{13}C-NMR (125.65 MHz, $CDCl_3$): δ = 174.3, 51.3, 34.1, 31.7, 29.4, 29.23, 29.20, 29.1, 24.9, 22.1 (t, J = 19.0 Hz), 13.6 ppm (t, J = 19.0 Hz). (see Spectrum)

HRMS (ESI): $[M+H]^+$ calcd. for $[C_{11}H_{21}D_2O_2]^+$ 189.1818, found 189.1814

TLC: Rf = 0.55 (5 % ethyl-acetate in hexanes, $KMnO_4$)

Compound 43 (oxacyclododecan-2-one)

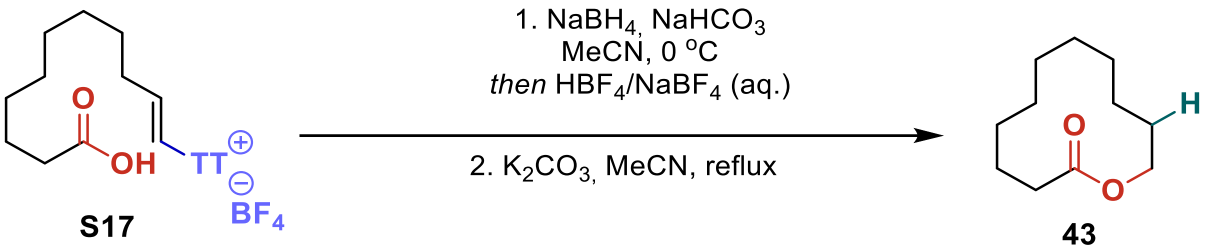

Alkenyl thianthrene-5-ium salt **S17** was prepared from undec-10-enoic acid using literature known procedures[54]. (1.24 g, 81% yield). A suspension of alkenyl thianthren-5-ium salt (243 mg, 0.500 mmol, 1 equiv.) and sodium bicarbonate (210 mg, 2.50 mmol, 5.00 equiv.) in anhydrous acetonitrile (5.0 mL, 0.1 M) was cooled to 0 °C under argon atmosphere. At this temperature well powdered sodium borohydride (13.2 mg, 0.70 mmol, 0.70 equiv.) was added in one portion. After stirring 60 min when analysis by LC-MS indicated full conversion, a mixture of $HBF_4.OEt_2$ (0.5 mL) and saturated aqueous $NaBF_4$ (20 mL) and dichloromethane (20 mL) were subsequently added to the mixture. The layers were separated, and the organic phase was dried over sodium sulfate. The dried solution was filtered, and the filtrate was concentrated under reduced pressure to yield the crude alkyl thiantren-5-ium tetrafluoroborate salt.

A suspension of potassium carbonate (207 mg, 1.50 mmol, 3 equiv.) in anhydrous acetonitrile (500 mL) was heated under reflux under argon atmosphere. At this temperature, the crude alkyl thianthrene-5-ium salt in acetonitrile (15 mL) was added dropwise via syringe pump over 24 h. Then, after stirring for an additional 24 h at this temperature, the suspension was cooled to ambient temperature and filtered. The filtrate was concentrated under reduced pressure to yield the crude macrocycle **43**. The residue was purified by flash column chromatography on silica gel (gradient elution from hexanes to 20% ethyl acetate in hexanes) to yield the pure macrocycle (28.5 mg, 0.155 mmol, 25% over 3 steps from alkene, 31% NMR yield).

Physical State: colorless oil

^{1}H-NMR (499.64 MHz, $CDCl_3$): δ = 4.22 – 4.16 (m, 2H), 2.40 – 2.32 (m, 2H), 1.75 – 1.69 (m, 2H), 1.66 (qd, J = 6.4, 4.6 Hz, 2H), 1.53 (dq, J = 7.3, 6.2 Hz, 2H), 1.44 – 1.29 ppm (m, 10H). (see Spectrum)

^{13}C-NMR (125.65 MHz, $CDCl_3$): δ = 173.9, 64.6, 34.4, 26.1, 24.89, 24.88, 24.5, 24.1, 23.9, 23.5, 23.3 ppm. (see Spectrum)

TLC: Rf = 0.30 (5 % ethyl-acetate in hexanes, $KMnO_4$)

Compound 44

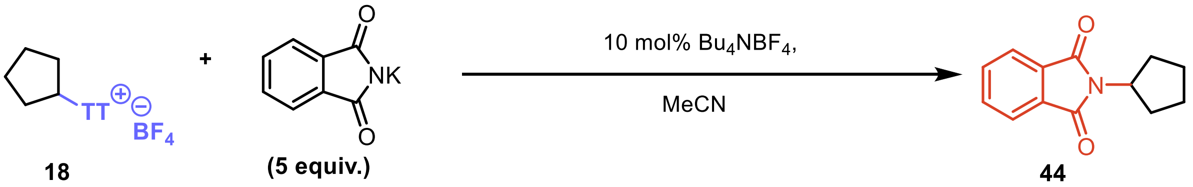

To the solution of the alkyl thianthren-5-ium salt **18** [Note 1] (0.35 g, 0.94 mmol, 1 equiv.) in anhydrous acetonitrile (0.1 M) were added potassium phthalimide (871 mg, 4.70 mmol, 5.00 equiv.), and tetrabutylammonium tetrafluoroborate (31 mg, 0.094 mmol, 0.1 equiv.) and the reaction mixture was stirred at 60 °C for 18 h. [Note 2] After analysis by TLC and LC-MS indicated the completion of the reaction, dichloromethane (50 mL) and saturated brine (50 mL) were added to the reaction mixture. The phases were separated, and the aqueous layer was extracted with dichloromethane (3x50 mL). The combined organic layers were dried using anhydrous Na_2SO_4, filtered, and concentrated under reduced pressure. The residue was purified by flash column chromatography on silica gel (gradient elution from hexanes to 20% ethyl acetate in hexanes) to yield **44** (80 mg, 0.37 mmol, 40%). The NMR spectra are in accordance with literature data.[73]

[Note 1]: The use of oven dried glassware is recommended as alkyl thianthren-5-ium salts may be water sensitive.
[Note 2]: A heating block was used. The exact external temperature is noted.

Physical State: white solid

^{1}H-NMR (499.64 MHz, $CDCl_3$): *d* = 7.81 (dd, *J* = 5.4, 3.1 Hz, 2H), 7.69 (dd, *J* = 5.5, 3.0 Hz, 2H), 4.63 (p, *J* = 8.5 Hz, 1H), 2.19 – 2.04 (m, 2H), 2.03 – 1.85 (m, 4H), 1.70 – 1.59 ppm (m, 2H). (see Spectrum)

^{13}C-NMR (125.65 MHz, $CDCl_3$): *d* = 168.5, 133.7, 132.2, 123.0, 51.0, 29.6, 25.1 ppm. (see Spectrum)

4.3. Applications and one-pot modifications

Compound 45 (methyl 12-phenyldodec-11-ynoate)

The preparation of **45** was carried out according to the general procedure reported by Shi[61].

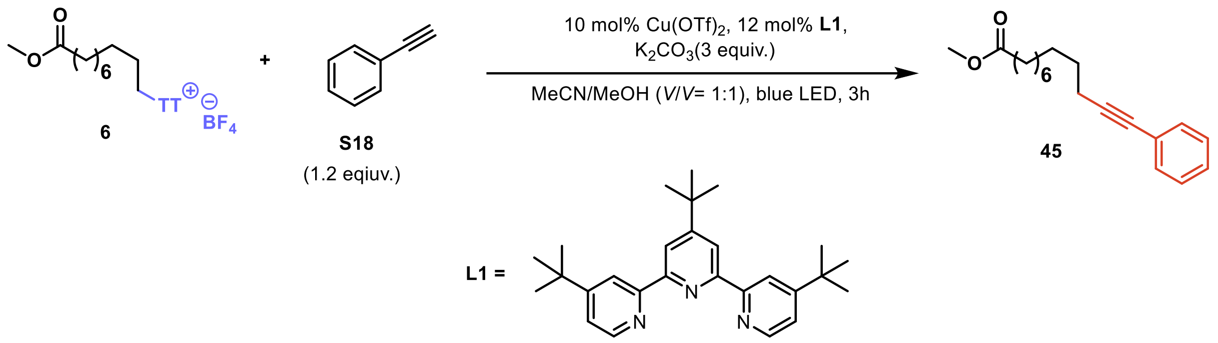

In a nitrogen-filled glovebox, to a 4.0 ml vial equipped with a stirring bar, $Cu(OTf)_2$ (7.41 mg, 20.5 µmol, 0.1 equiv.), K_2CO_3 (84.9 mg, 614 µmol, 3 equiv.) [Note 1], and 4,4',4"-tri-tert-butyl-2,2':6',2"-terpyridine (**L1**) (9.87 mg, 24.6 µmol, 0.12 equiv.) were added. Next, a solution of **6** (100 mg, 205 µmol, 1 equiv.) in acetonitrile (0.4 M) [Note 2] was added. Finally, methanol (0.5 mL) [Note 2] and ethynylbenzene (**S18**)(25.1 mg, 246 µmol, 27 µL, 1.2 equiv) were added to the mixture. The vial was taken out from the glovebox, equipped with an argon filled balloon, and the reaction mixture was stirred under blue light irradiation for 3 h [Note 3]. Subsequently, the mixture was diluted with hexanes (10 mL) and filtered through a short silica plug. The silica layer was further washed with hexanes/ethyl-acetate 10:1 (50 mL). The combined filtrate was concentrated under reduced pressure. The residue was purified by flash column chromatography on silica gel (0% ethyl-acetate in hexanes grading to 30% ethyl-acetate in hexanes) to afford the pure product **45** (25 mg, 205 µmol, 43%).

[Note 1] The anhydrous potassium carbonate was stored in a 120°C oven to keep it dry.

[Note 2] The solvents were freshly distilled from CaH_2 and flushed with argon.

[Note 3] For the photochemical reaction, a 3D printed reactor was used based on the Wisconsin Photoreactor Platform[74], equipped with a 9 W, 455 nm blue LED. The reaction mixture was only cooled by the fan inside the reactor.

Physical State: yellow oil

^{1}H-NMR (499.64 MHz, $CDCl_3$): δ = 7.41 – 7.37 (m, 2H), 7.29 – 7.24 (m, 3H), 3.66 (s, 3H), 2.40 (t, J = 7.1 Hz, 2H), 2.30 (t, J = 7.5 Hz, 2H), 1.66 – 1.55 (m, 4H), 1.45 (dq, J = 13.8, 6.6 Hz, 2H), 1.37 – 1.22 ppm (m, 8H). (see Spectrum)

^{13}C-NMR (125.65 MHz, $CDCl_3$): δ = 174.3, 131.5, 128.1, 127.4, 124.1, 90.4, 80.6, 51.4, 34.1, 29.3, 29.2, 29.11, 29.06, 28.9, 28.7, 24.9, 19.4 ppm. (see Spectrum)

HRMS (FI): M^+ calcd. for $[C_{19}H_{26}O_2]^+$ 286.1927, found 286.1937

TLC: Rf = 0.7 (hexanes/ethyl acetate 10:1, 254 nm UV)

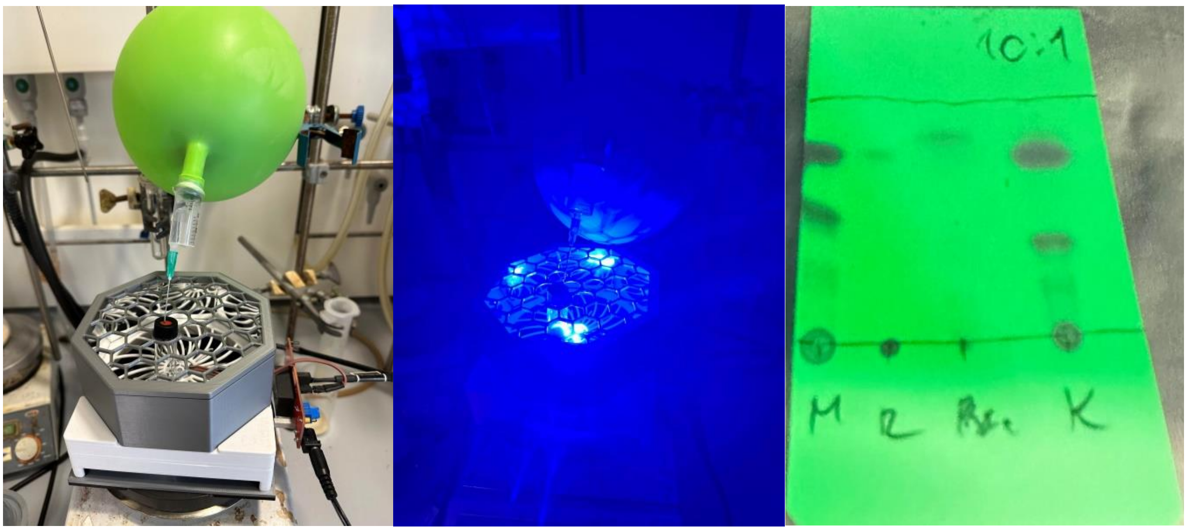

(A) Reaction setup **(B)** The TLC of the reaction after 3 h (spots: M (reaction mixture), R (alkyl-thiantrenium salt), PhAc (ethynylbenzene), K (co-spot), eluent: hexanes/ethyl acetate 10:1, visualisation: 254 nm UV)

Compound 46 (methyl decanoate)

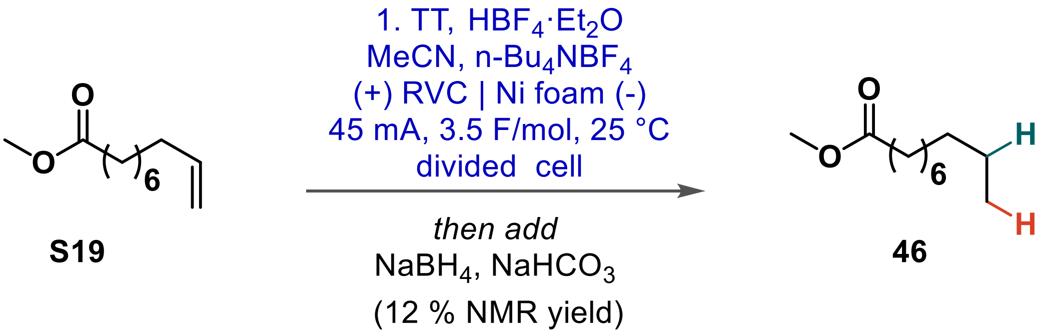

Tetrafluoroboric acid diethyl ether complex (1.62 g, 1.36 mL, 5 equiv., 10.0 mmol) was added to a solution of tetra-*n*-butylammonium tetrafluoroborate (527 mg, 0.8 equiv., 1.60 mmol) in anhydrous acetonitrile (5 mL). This solution was transferred to the cathodic compartment of an IKA Pro-Divide divided electrochemical cell equipped with magnetic stirring bars. Next, methyl dec-9-enoate **S19** (9-DAME, 369 mg, 2.00 mmol ,1 equiv.) was added to a separate solution of tetra-*n*-butylammonium tetrafluoroborate (527 mg, 1.60 mmol , 0.8 equiv.) in anhydrous acetonitrile (5 mL). This solution in turn was transferred to the anodic compartment of the electrochemical cell. Finally, under stirring, thianthrene (TT, 649 mg, , 3.00 mmol, 1.5 equiv.) was added to the anodic compartment [Note 1]. The closing cap of the cell was equipped with a standard IKA RVC anode and a standard IKA nickel foam cathode, and the cell was sealed. Both compartments were flushed with argon gas through the septa on the top of the cell, after which argon-filled balloons were inserted into them. The cell was inserted into the IKA ElectraSyn 2.0 potentiostat and the reaction mixture was electrolyzed at a constant current of

45 mA for 3.5 F/mol of alkene, with stirring set to 400 RPM [Note 2]. Complete conversion of the alkene was achieved based on TLC or ^{1}H NMR measurement. Next, the cell was opened, and the lilac anodic solution was transferred into a 50 mL flask. The RVC anode and the anodic compartment were further washed with 5 mL anhydrous acetonitrile into the flask.At this point, solid sodium bicarbonate (6.72 g, 80.0 mmol, 8.00 equiv) was added in one portion, and the two-phase mixture was vigorously stirred at 25 °C for 3 min, whereupon an immediate color change to yellow was observed. Then, the two-phase mixture was cooled to 2 °C with an ice bath and sodium borohydride (1.89 g, 50.0 mmol, 5.00 equiv) was added in small portions, whereupon the temperature began to rise. The cooling was maintained for 5 min. then it was left to warm to 25 °C and stirred at this temperature for 4 h until analysis by LCMS indicated full conversion. Then, saturated aqueous sodium bicarbonate solution (120 ml) and diethyl ether (120 mL) were added to the reaction mixture. The phases were separated and the aqueous layer was extracted with diethyl ether (3x120 mL). The combined organic layers were dried with anhydrous Na_2SO_4, filtered, and concentrated under reduced pressure to afford the crude product **46** (12 % NMR yield).

[Note 1]: As the solubility of thianthrene is low in acetonitrile, complete dissolution of the solids will take place only as the electrochemical reaction consumes the thianthrene. Intensive stirring is needed to suspend the thianthrene so that the stirring bars of the Pro-Divide cell don't get stuck in the solid material.

[Note 2]: The IKA ElectraSyn was set up as follows: New experiments → Constant current → 45 mA → No reference electrode → Total charge → 2.0 mmol, 3.5 F/mol → No alternating polarity → Start → 400 RPM stirring.

For NMR spectra see Compound 46.

5. NMR Spectra

f1H-NMR (499.64 MHz, $CDCl_3$): of **S15** (see Procedure)

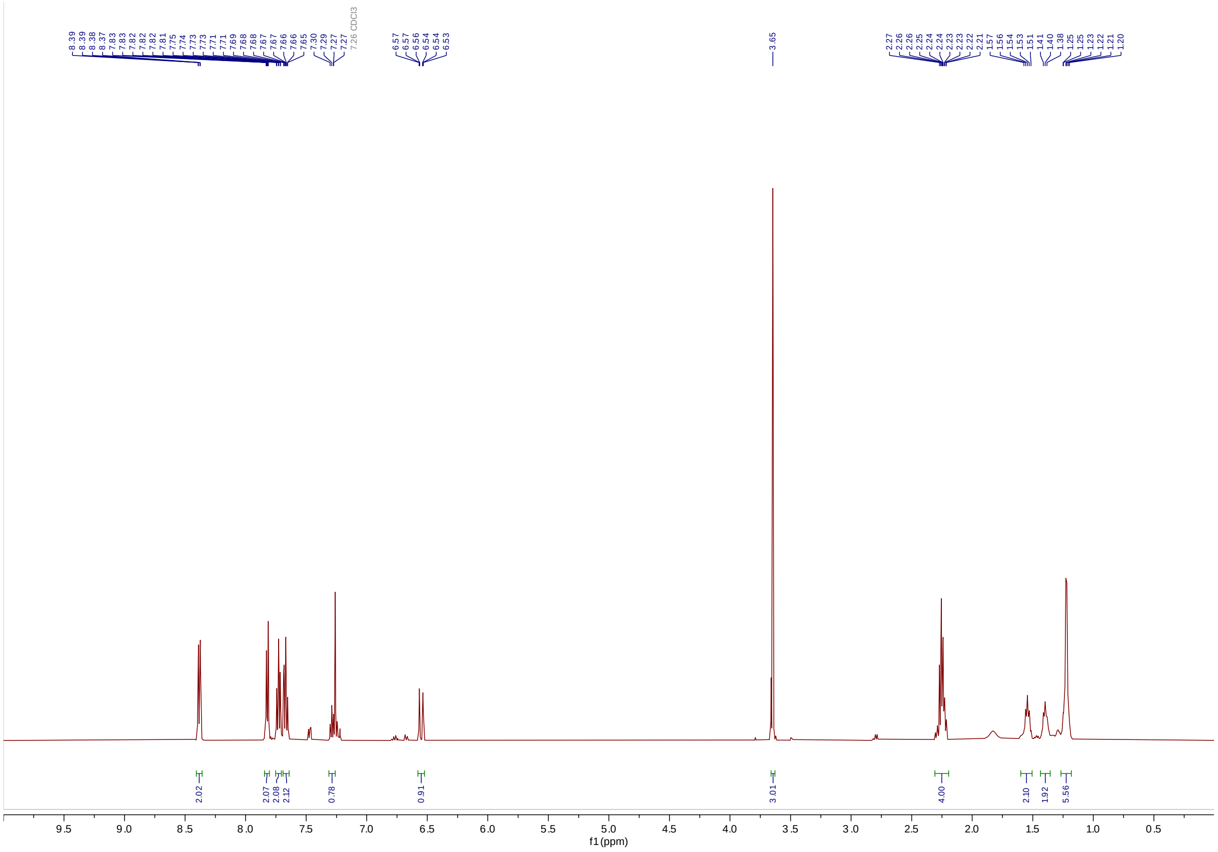

^{13}**C-NMR** (125.65 MHz, $CDCl_3$) of **S15** (see Procedure)

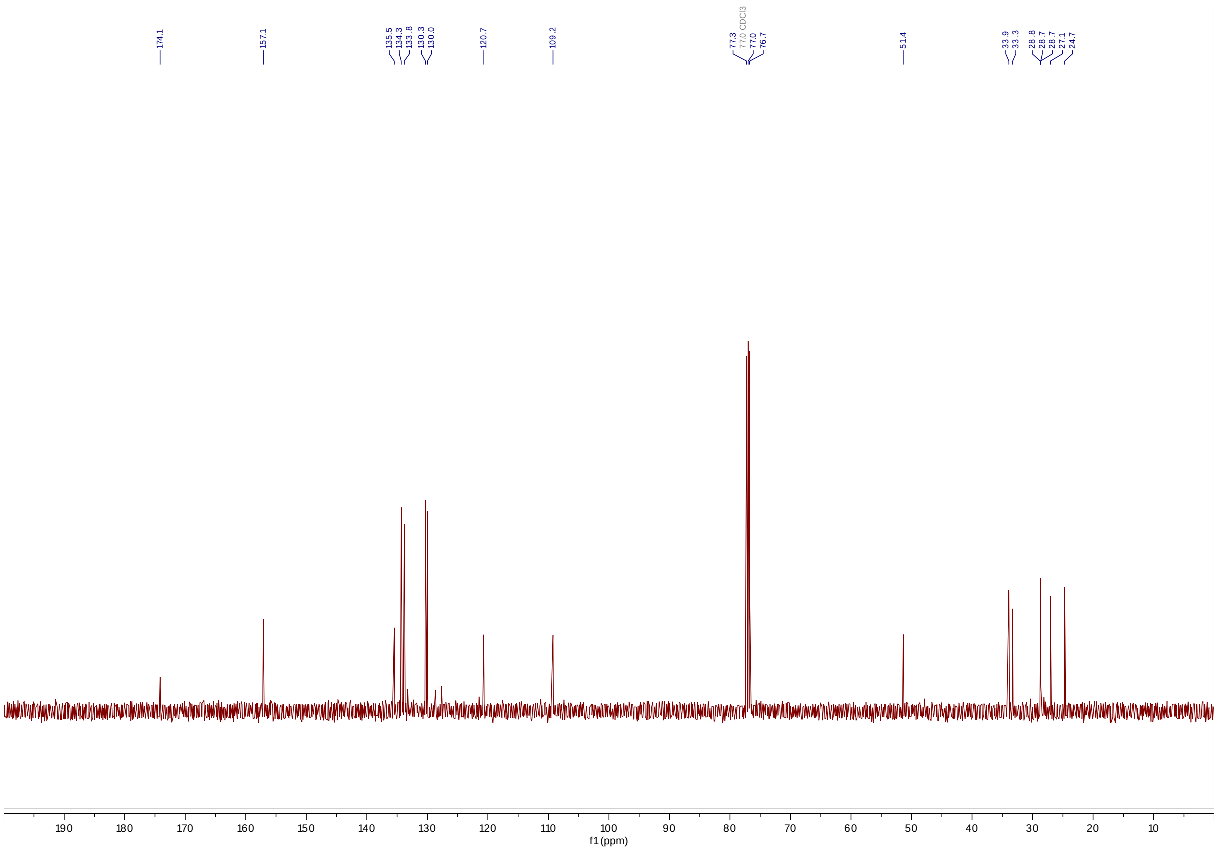

^{19}**F-NMR** (282.21 MHz, $CDCl_3$) of **S15** (see Procedure)

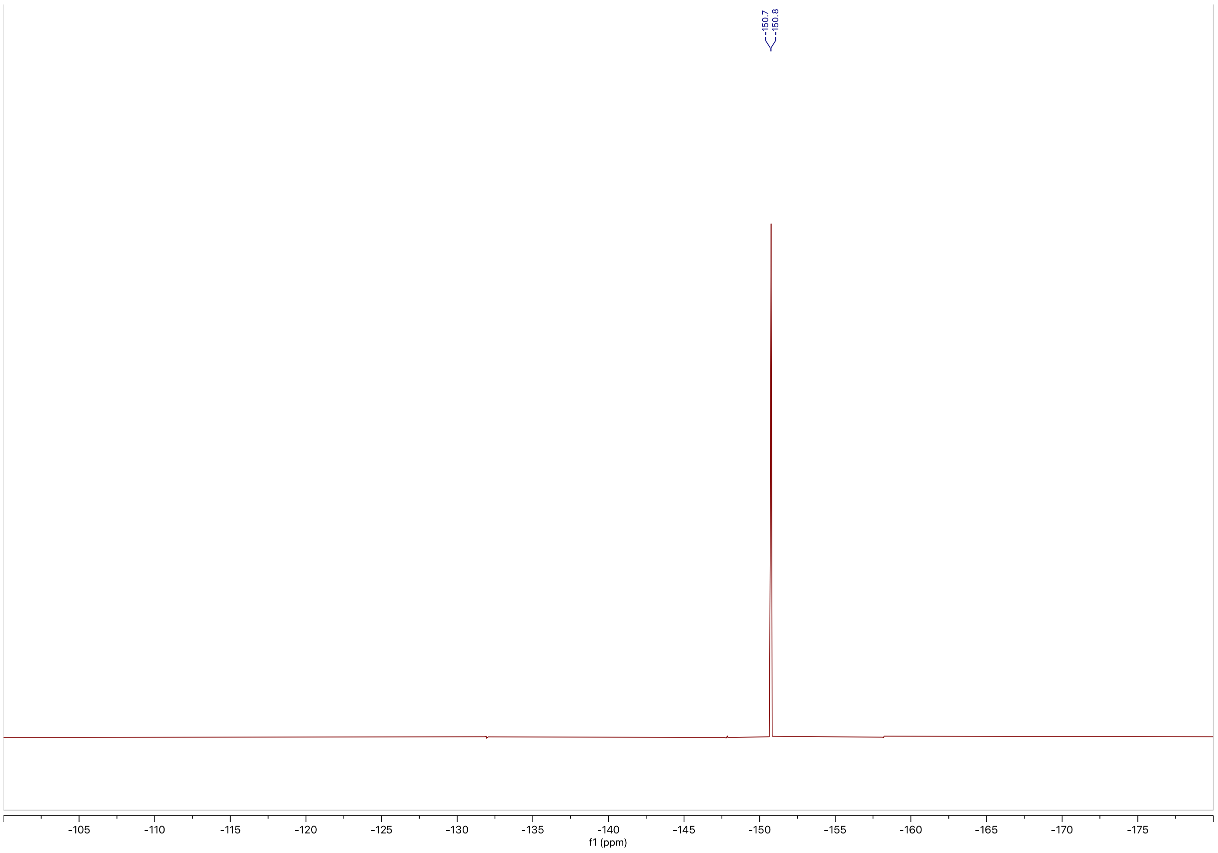

[1]**H-NMR** (499.64 MHz, DMSO-d_6): of **S16** (see Procedure)

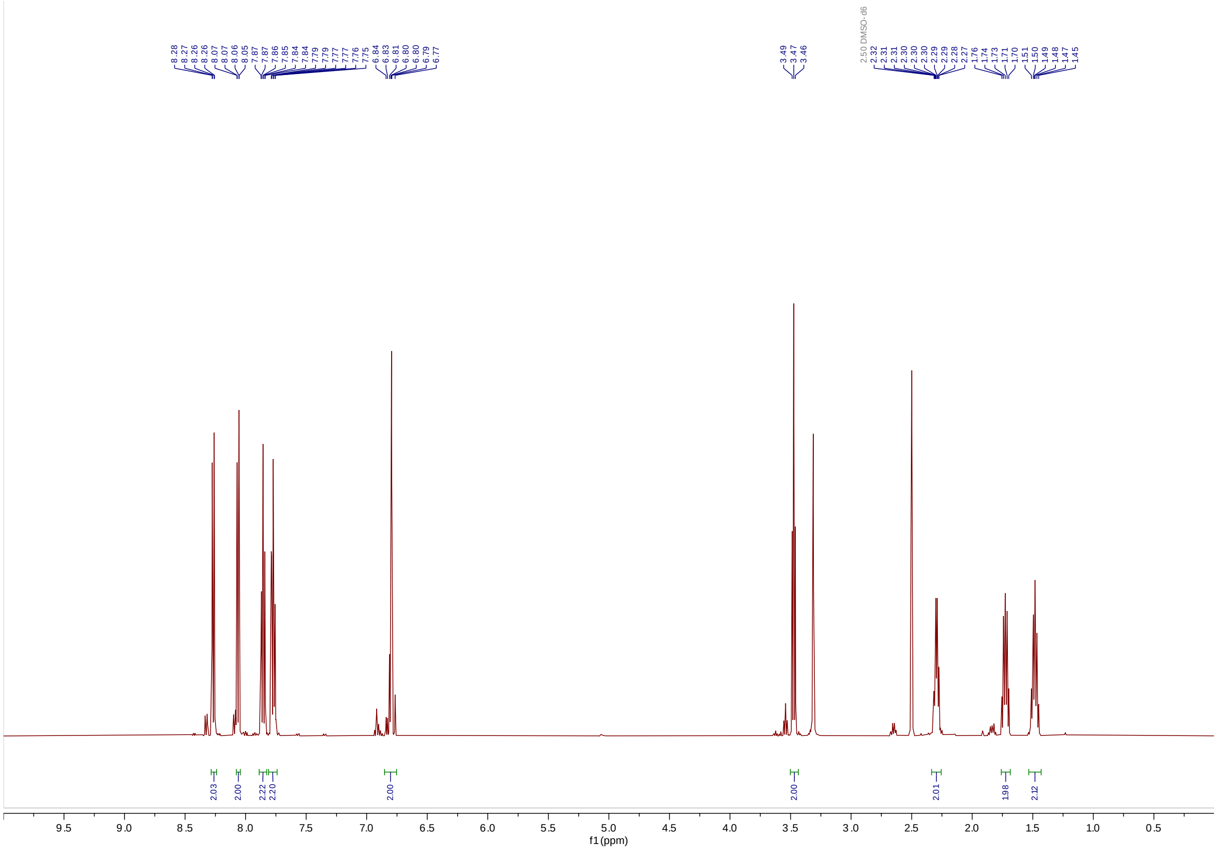

[13]**C-NMR** (125.65 MHz, DMSO-d_6) of **S16** (see Procedure)

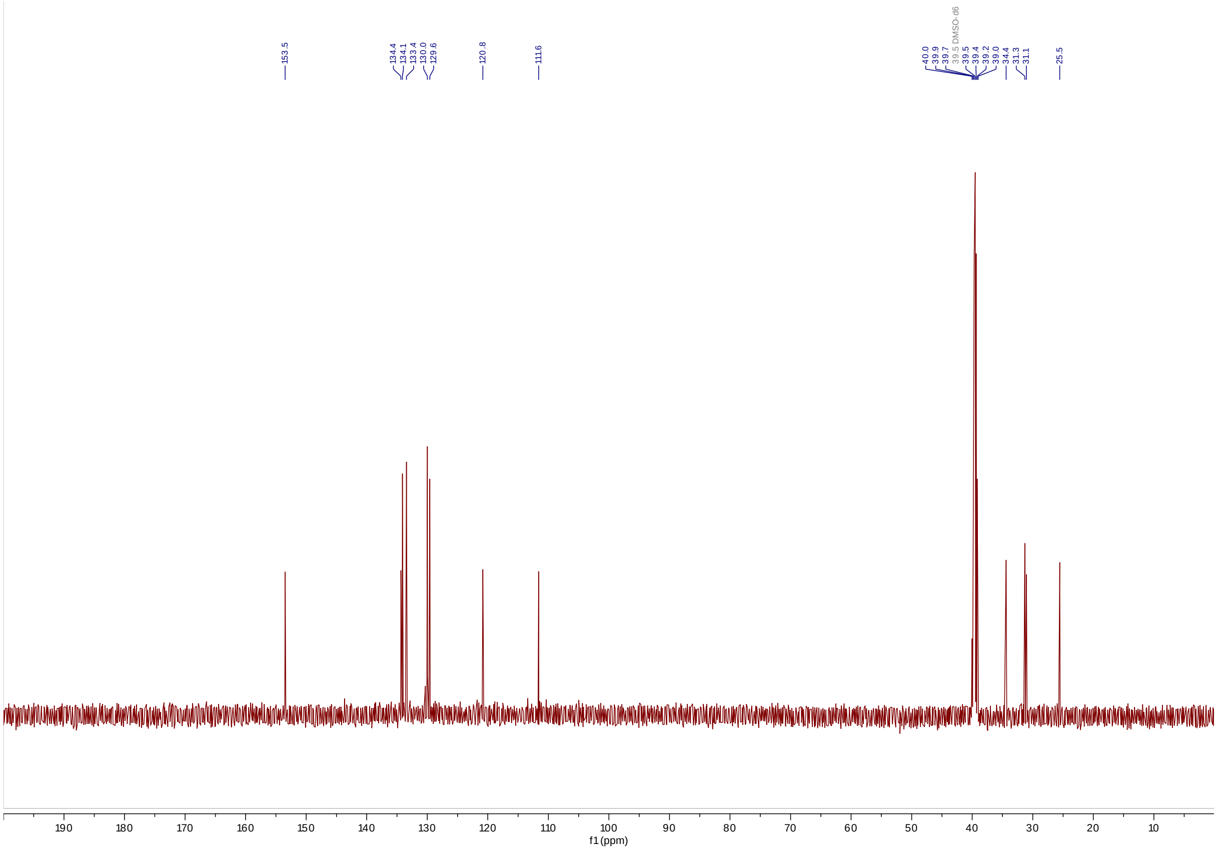

19F-NMR (282.21 MHz, DMSO-d_6) of **S16** (see Procedure)

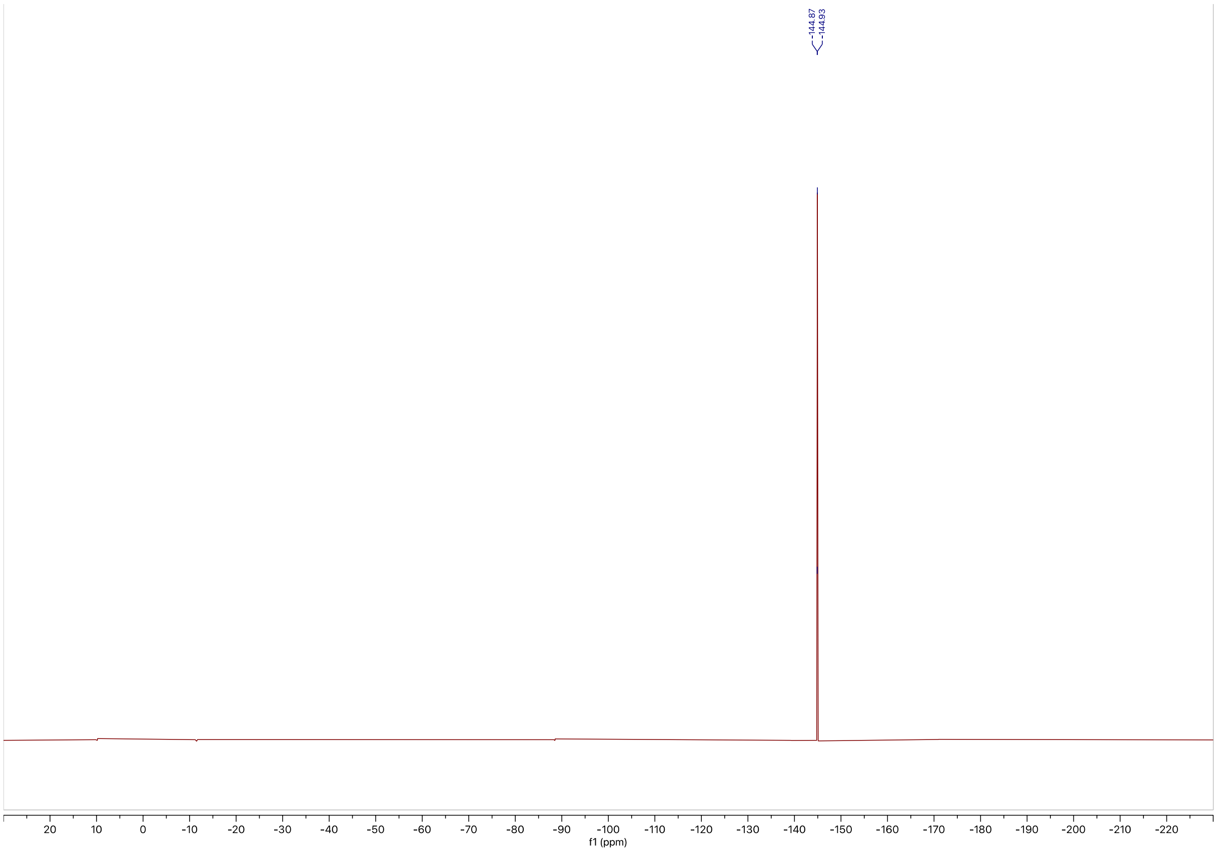

[1]**H-NMR** (499.64 MHz, DMSO-d_6): of **Compound 2** (see Procedure)

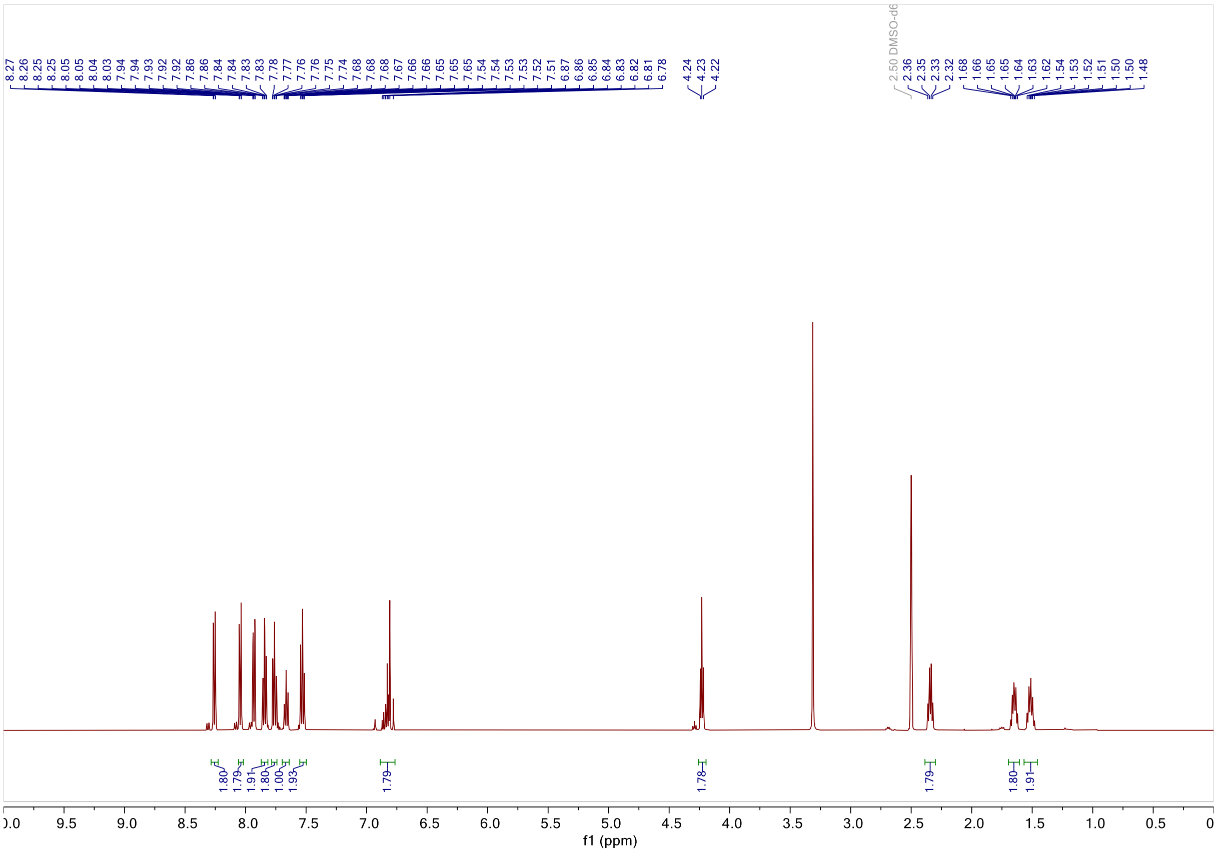

[13]**C-NMR** (125.65 MHz, DMSO-d_6) of **Compound 2** (see Procedure)

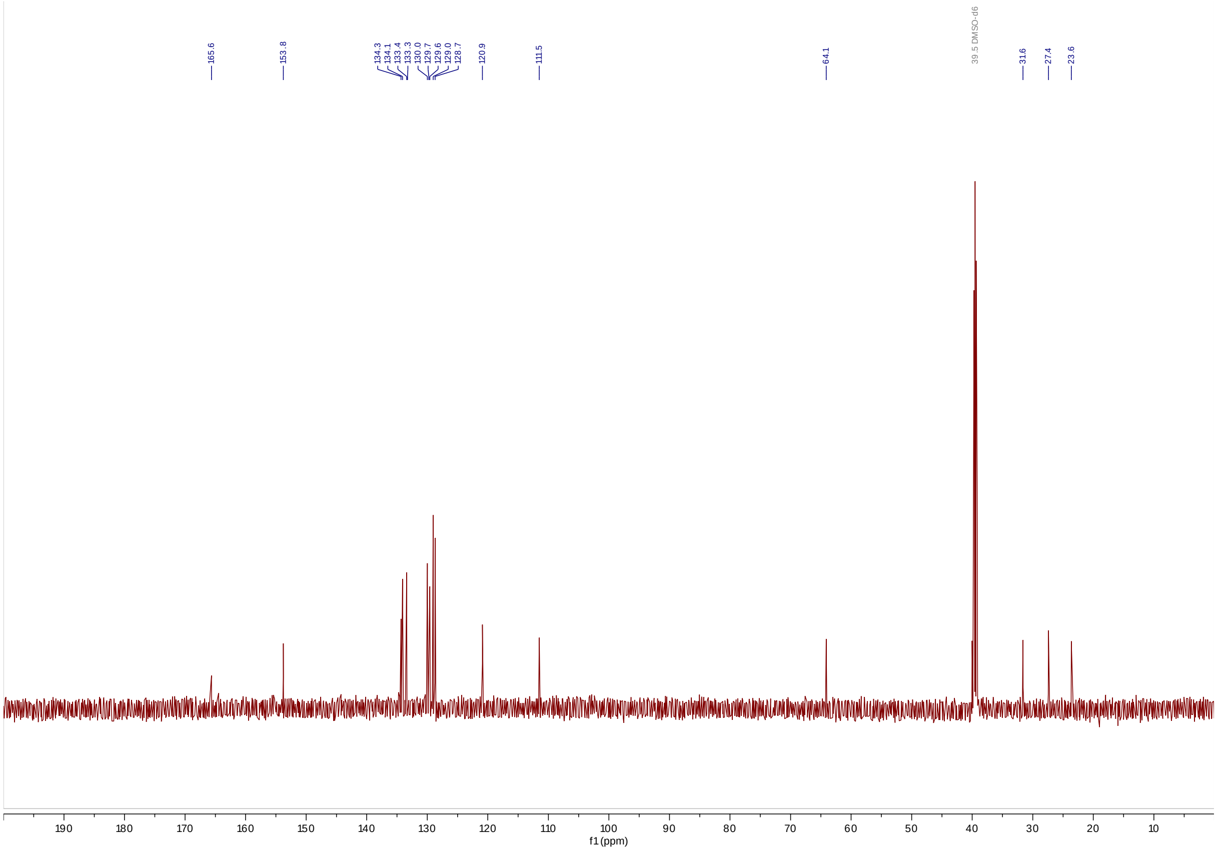

^{19}F-NMR (282.21 MHz, DMSO-d_6) of **Compound 2** (see Procedure)

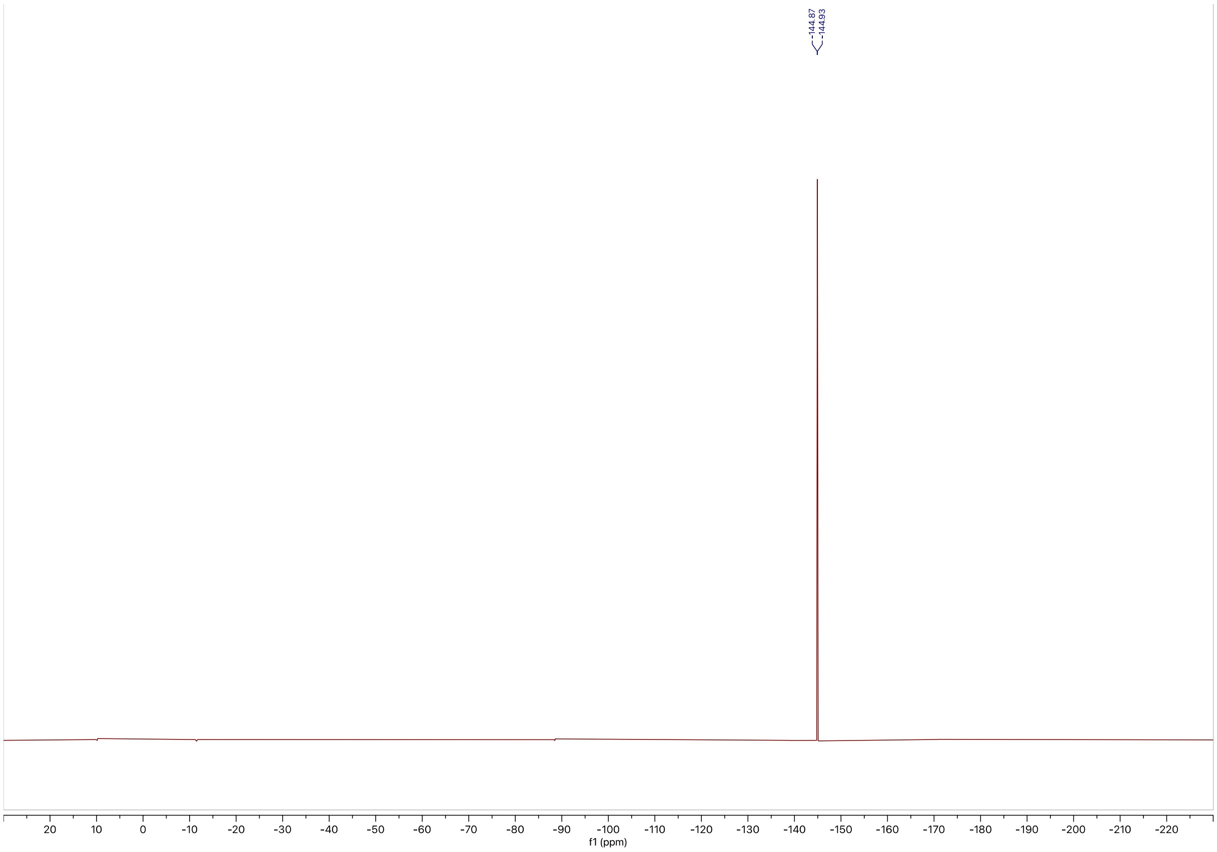

¹H-NMR (499.64 MHz, $CDCl_3$): of **Compound 21a** (see Procedure)

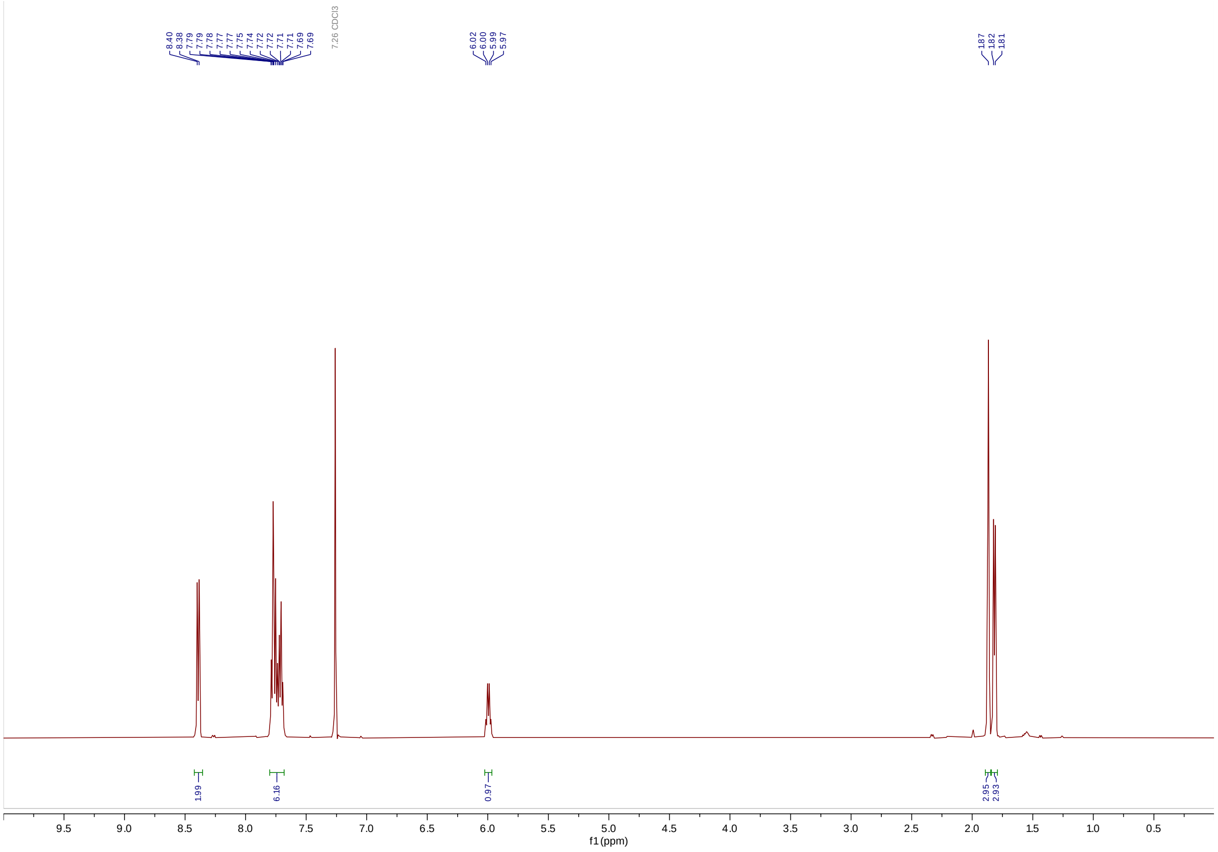

¹³C-NMR (125.65 MHz, $CDCl_3$) of **Compound 21a** (see Procedure)

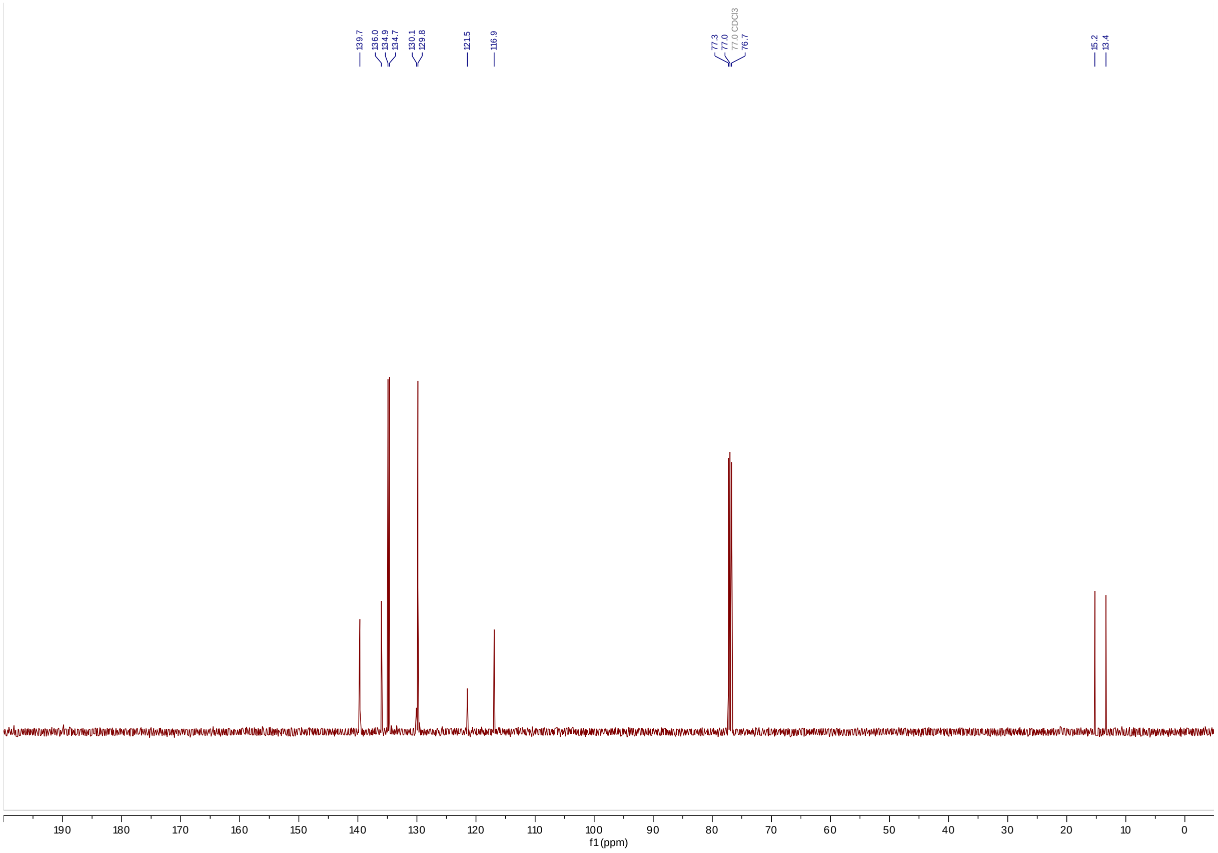

^{19}F-NMR (282.21 MHz, $CDCl_3$) of **Compound 21a** (see Procedure)

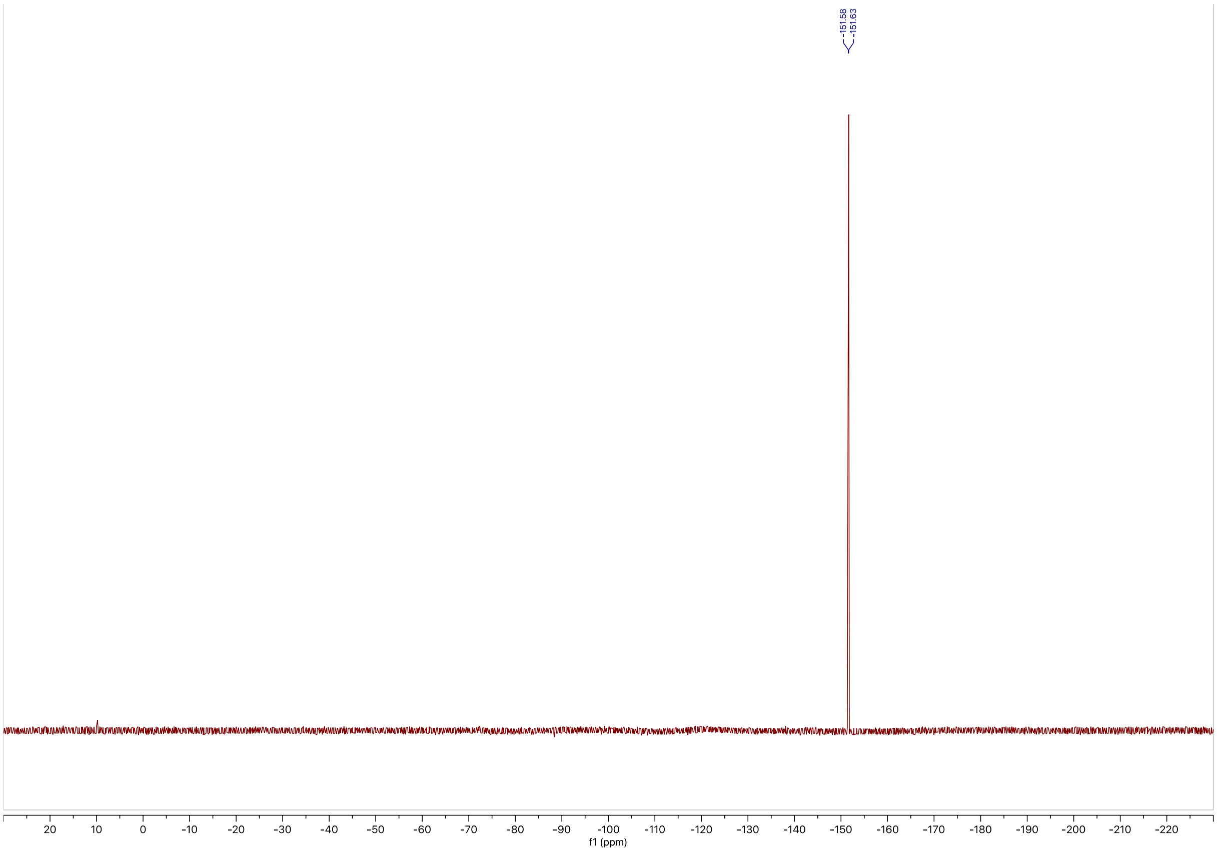

^{1}H-NMR (499.64 MHz, $CDCl_3$): of **Compound 21b** (see Procedure)

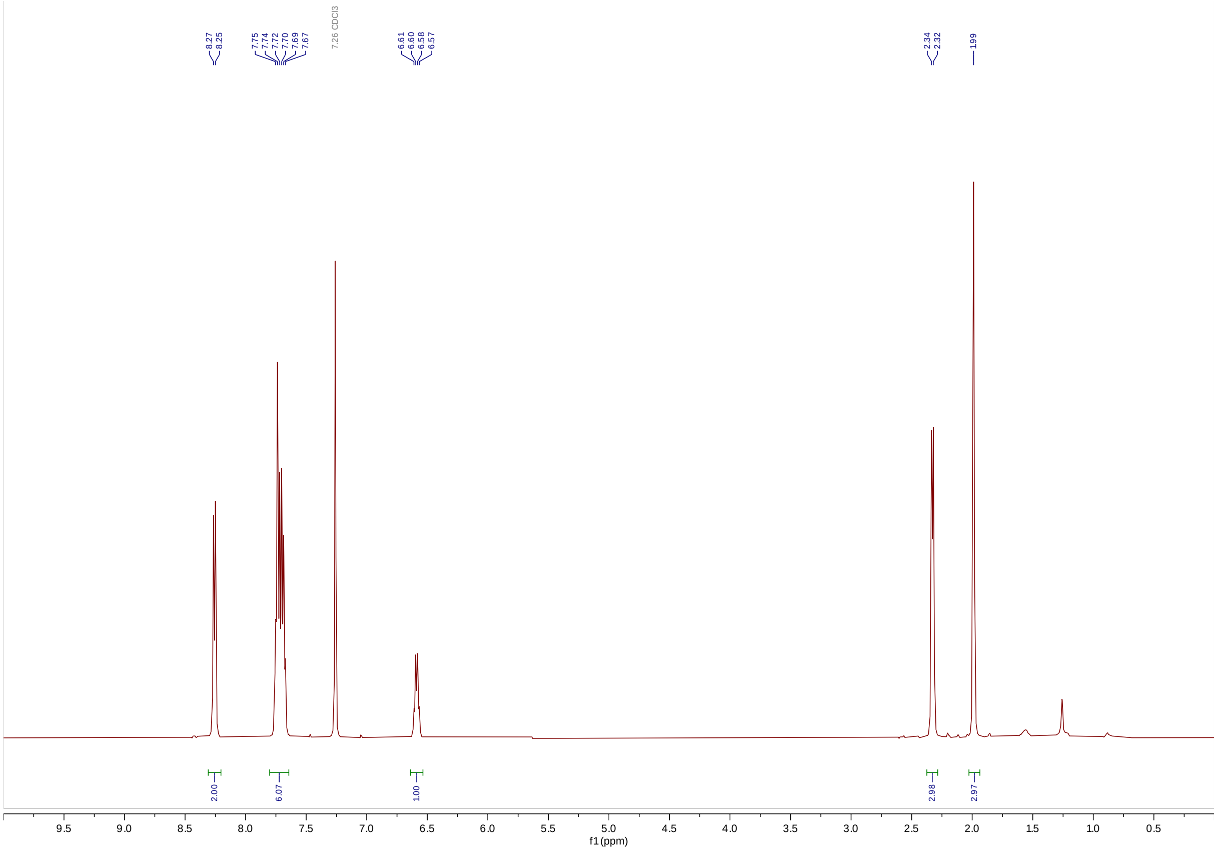

^{13}C-NMR (125.65 MHz, $CDCl_3$) of **Compound 21b** (see Procedure)

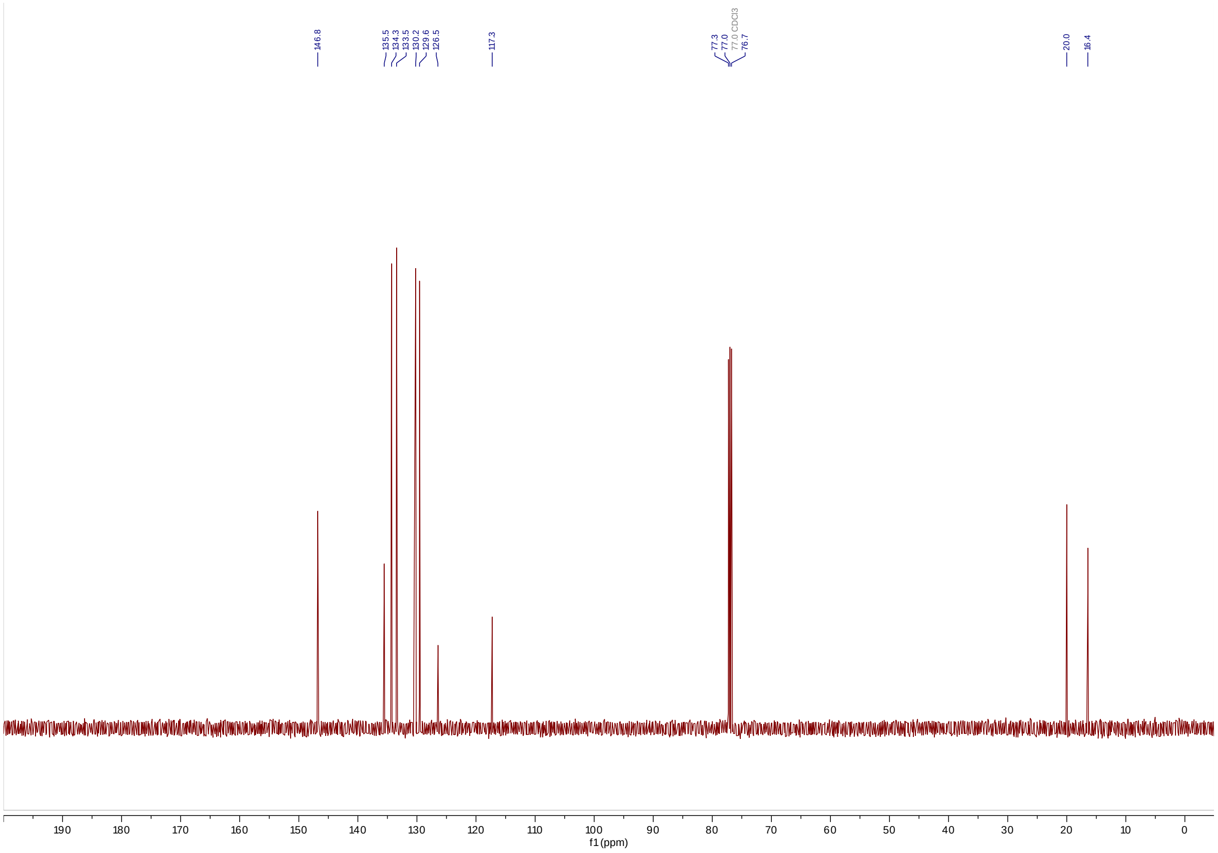

^{19}F-NMR (282.21 MHz, $CDCl_3$) of **Compound 21b** (see Procedure)

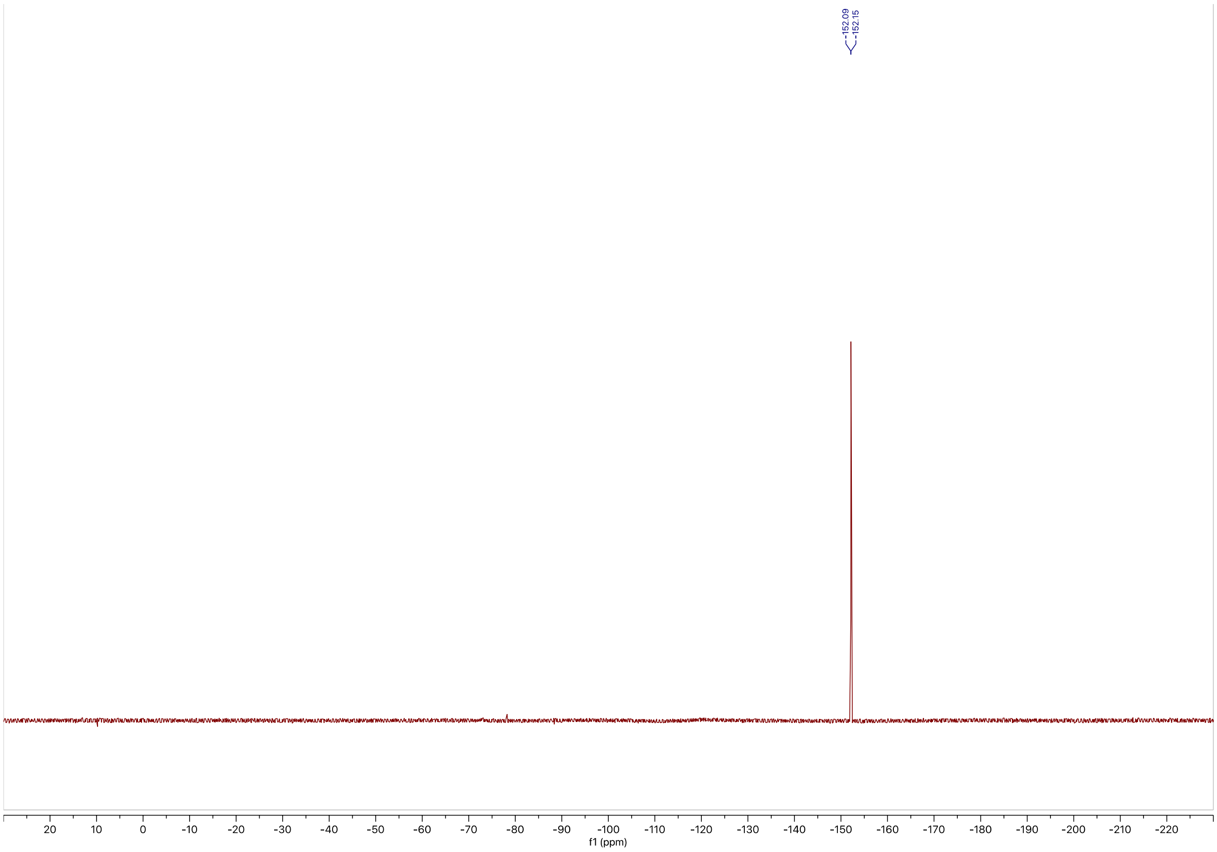

^{1}H-NMR (499.64 MHz, $CDCl_3$): of **Compound 3** (see Procedure)

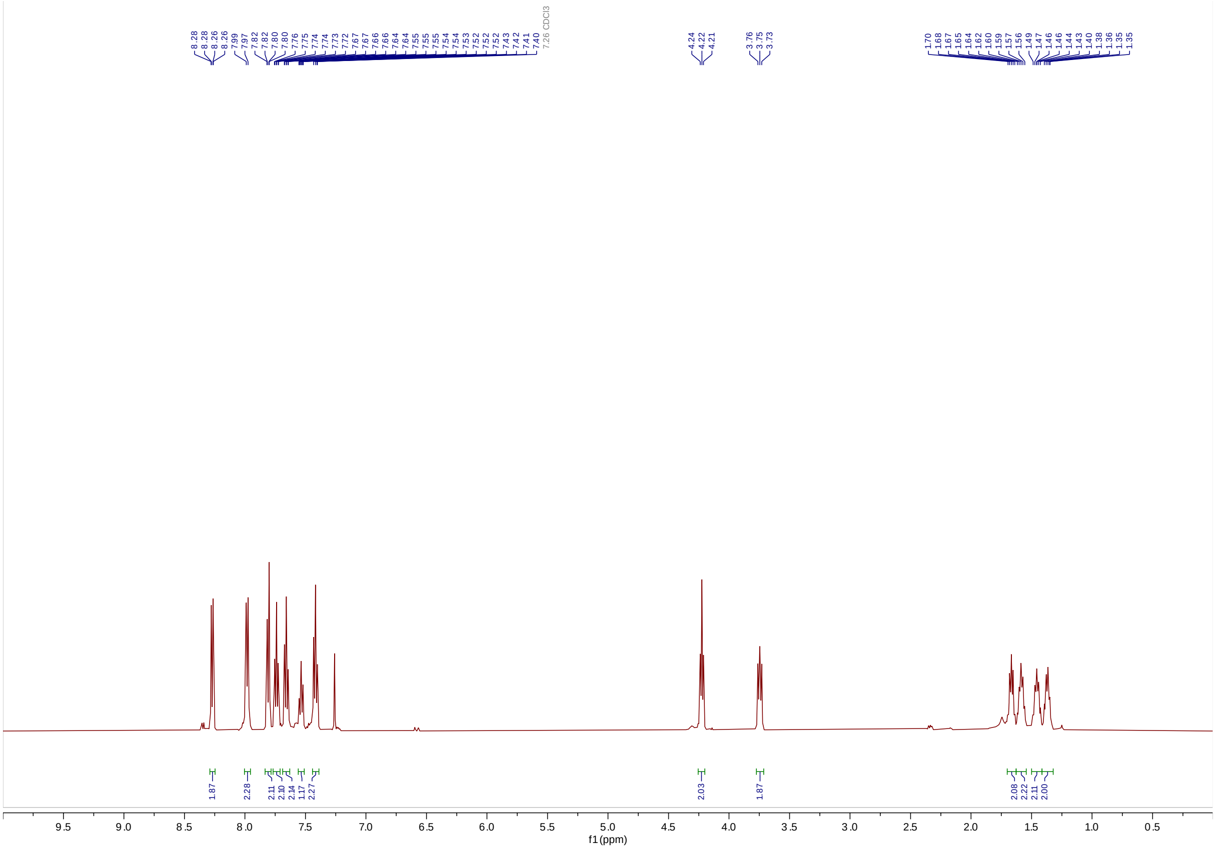

^{13}C-NMR (125.65 MHz, $CDCl_3$) of **Compound 3** (see Procedure)

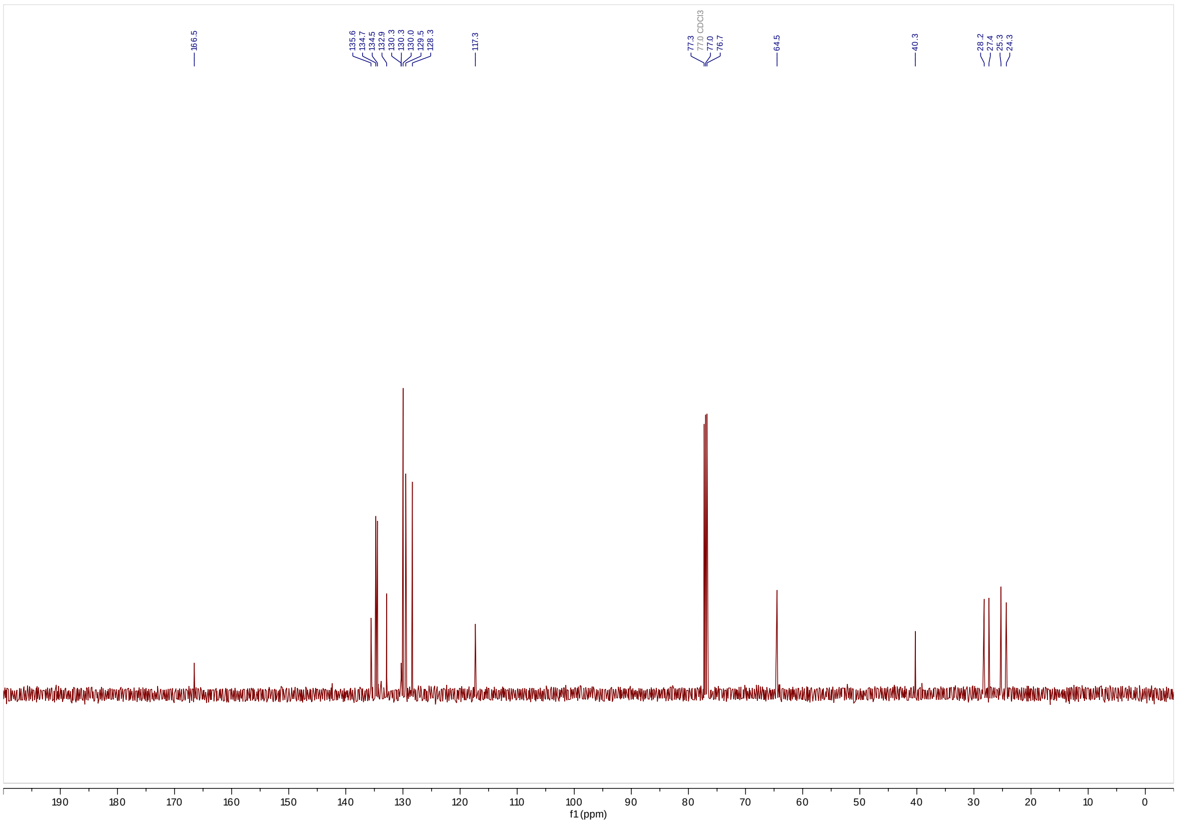

^{19}F-NMR (282.21 MHz, $CDCl_3$) of **Compound 3** (see Procedure)

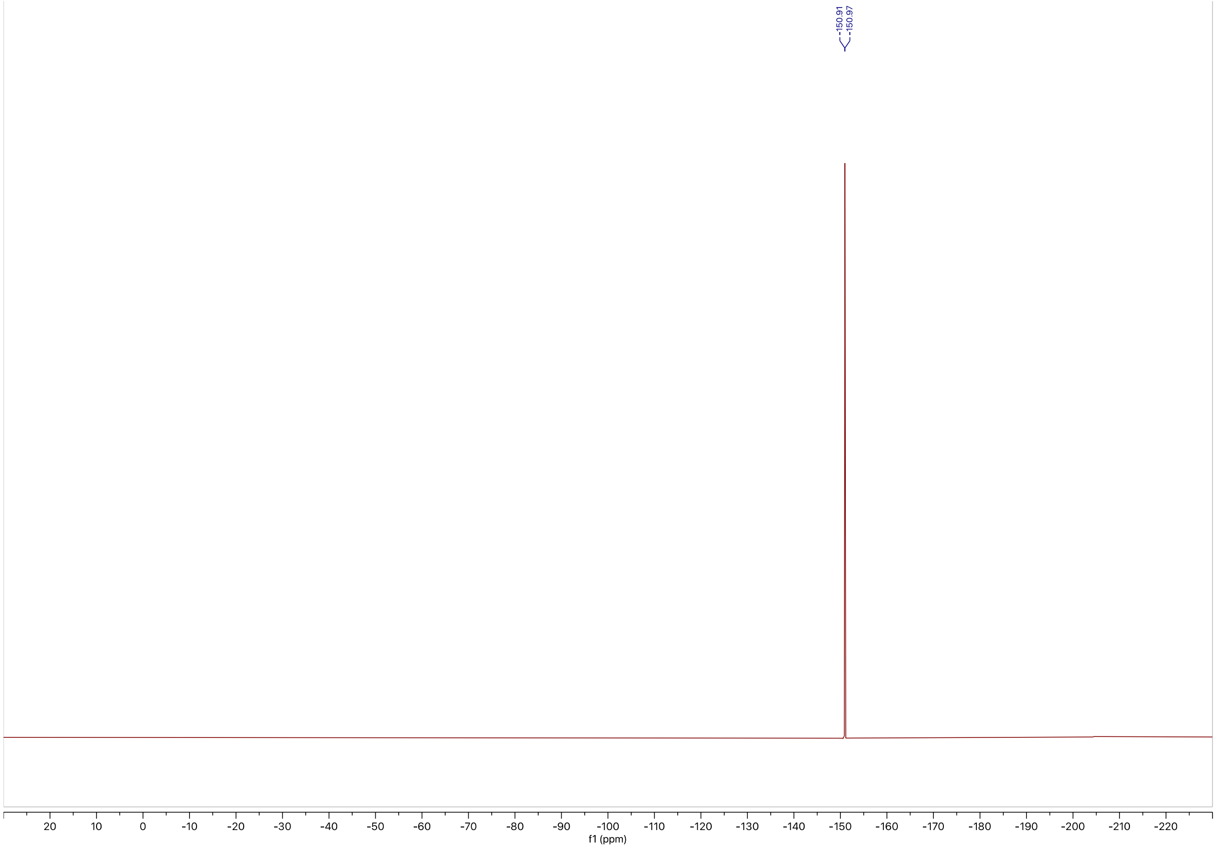

[1]**H-NMR** (499.64 MHz, $CDCl_3$): of **Compound 6** (see Procedure)

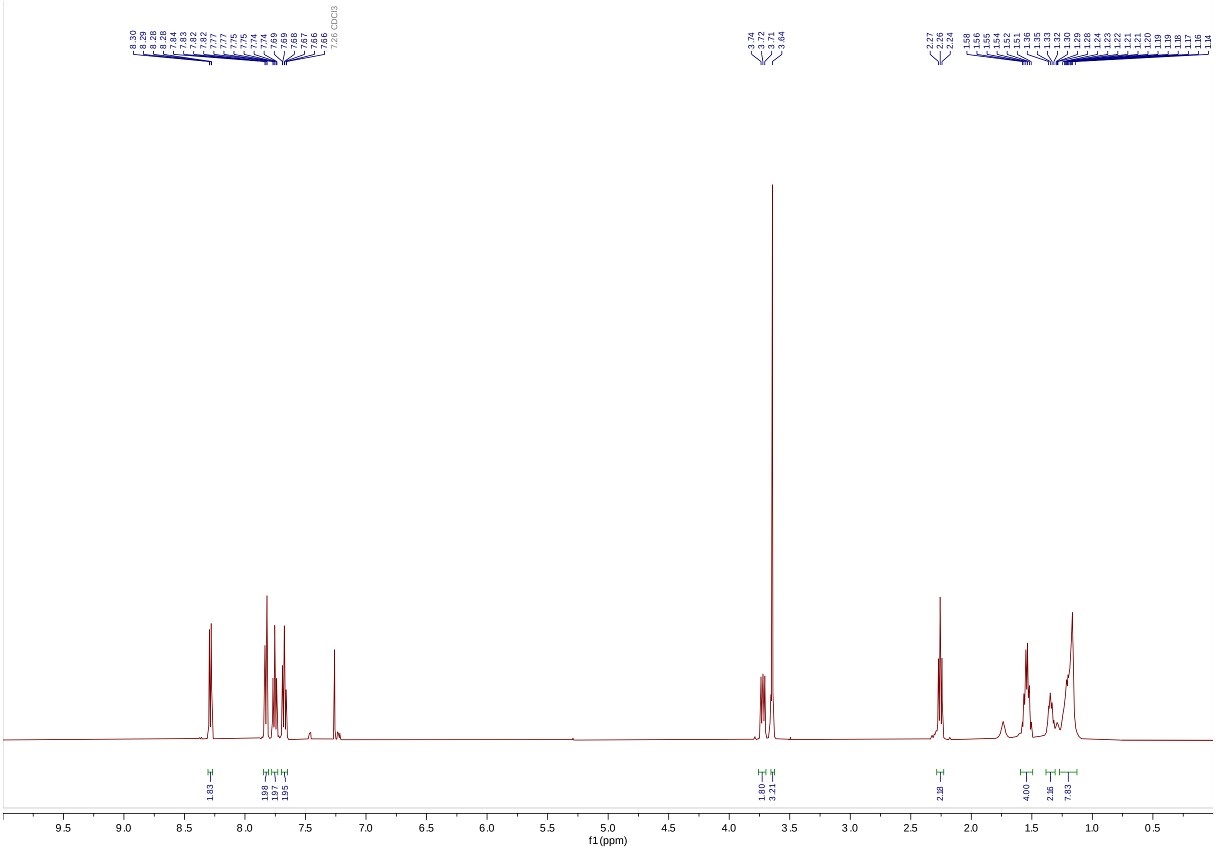

[13]**C-NMR** (125.65 MHz, $CDCl_3$) of **Compound 6** (see Procedure)

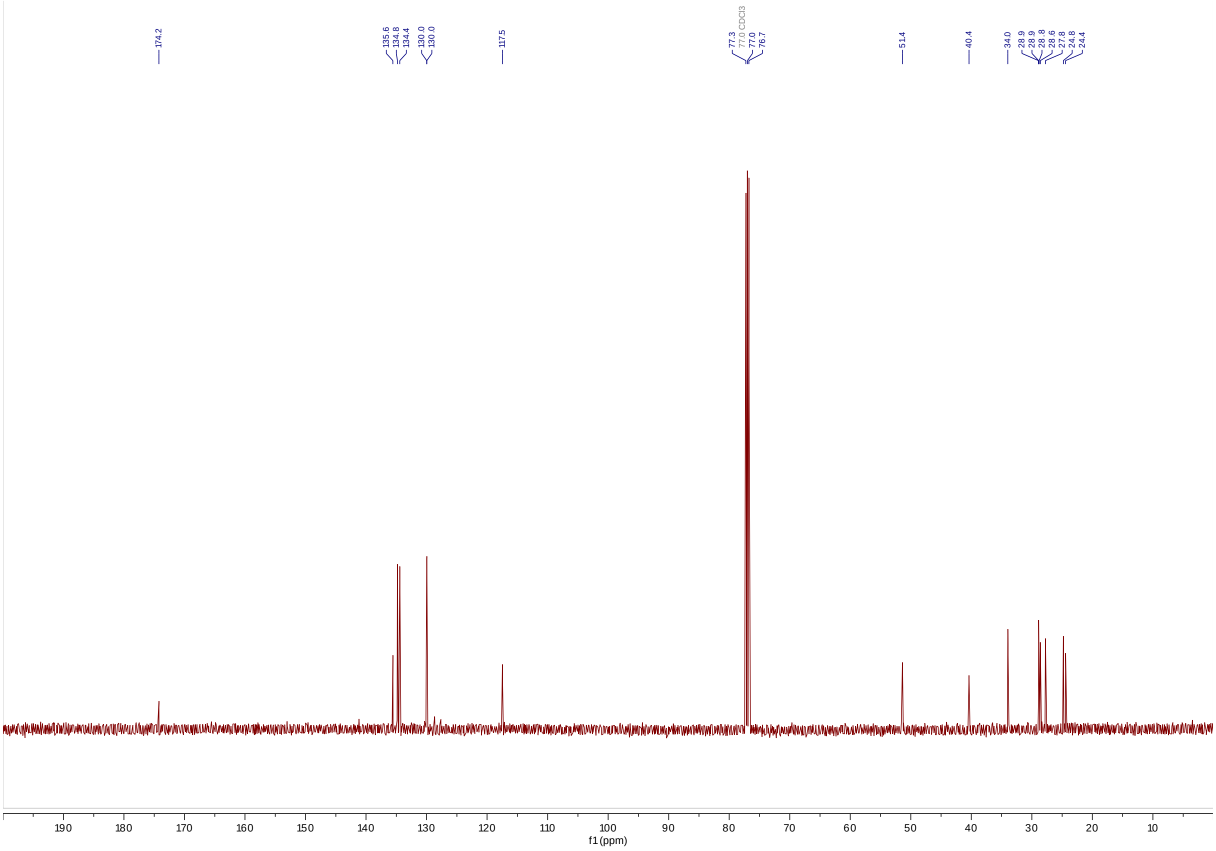

^{19}F-NMR (282.21 MHz, $CDCl_3$) of **Compound 6** (see Procedure)

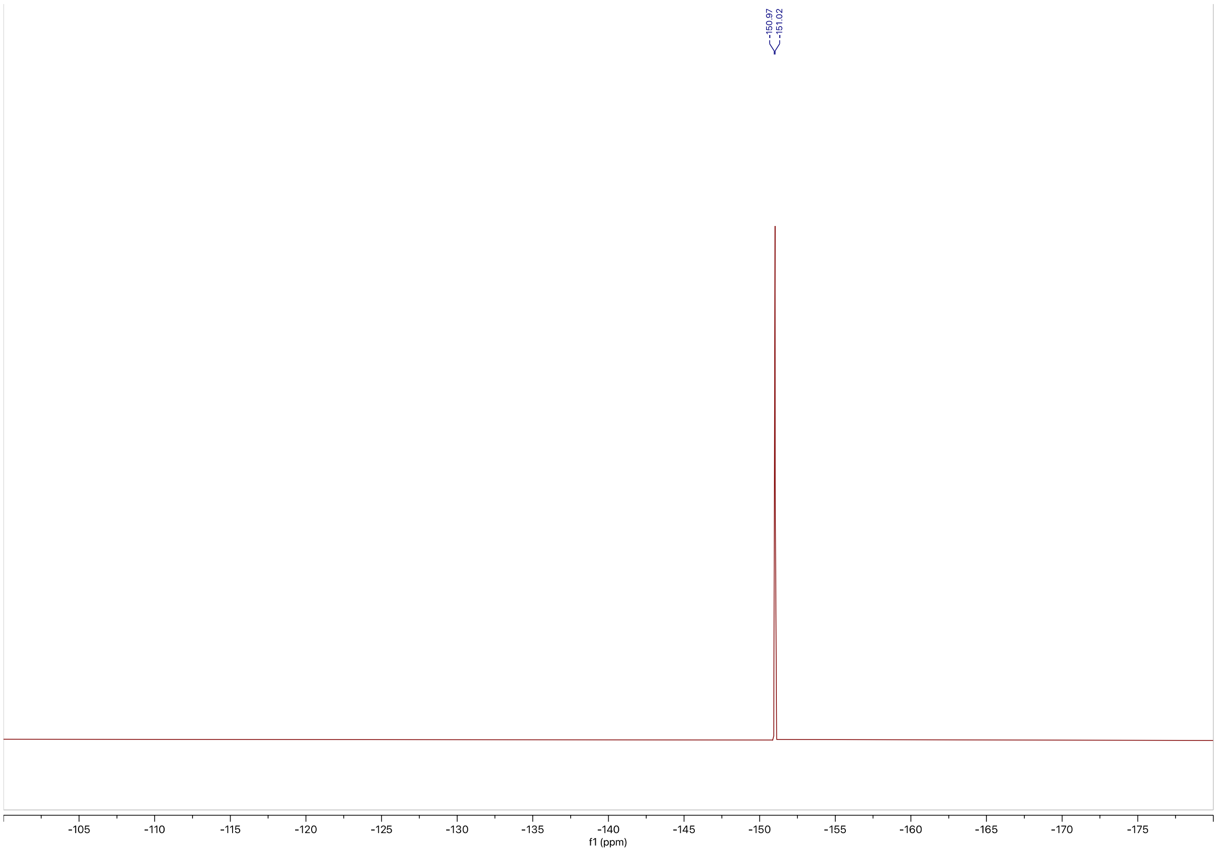

^{1}H-NMR (499.64 MHz, $CDCl_3$): of **Compound 7** (see Procedure)

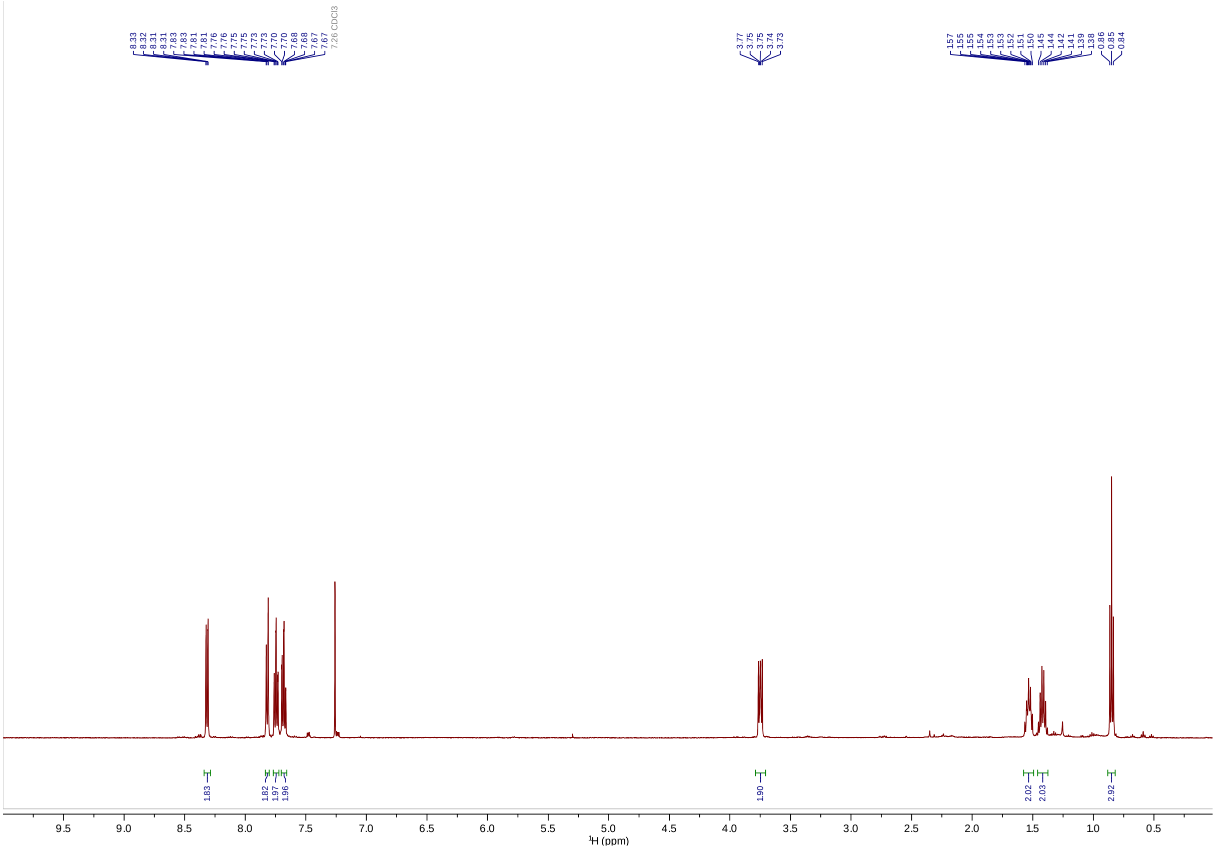

^{13}C-NMR (125.65 MHz, $CDCl_3$) of **Compound 7** (see Procedure)

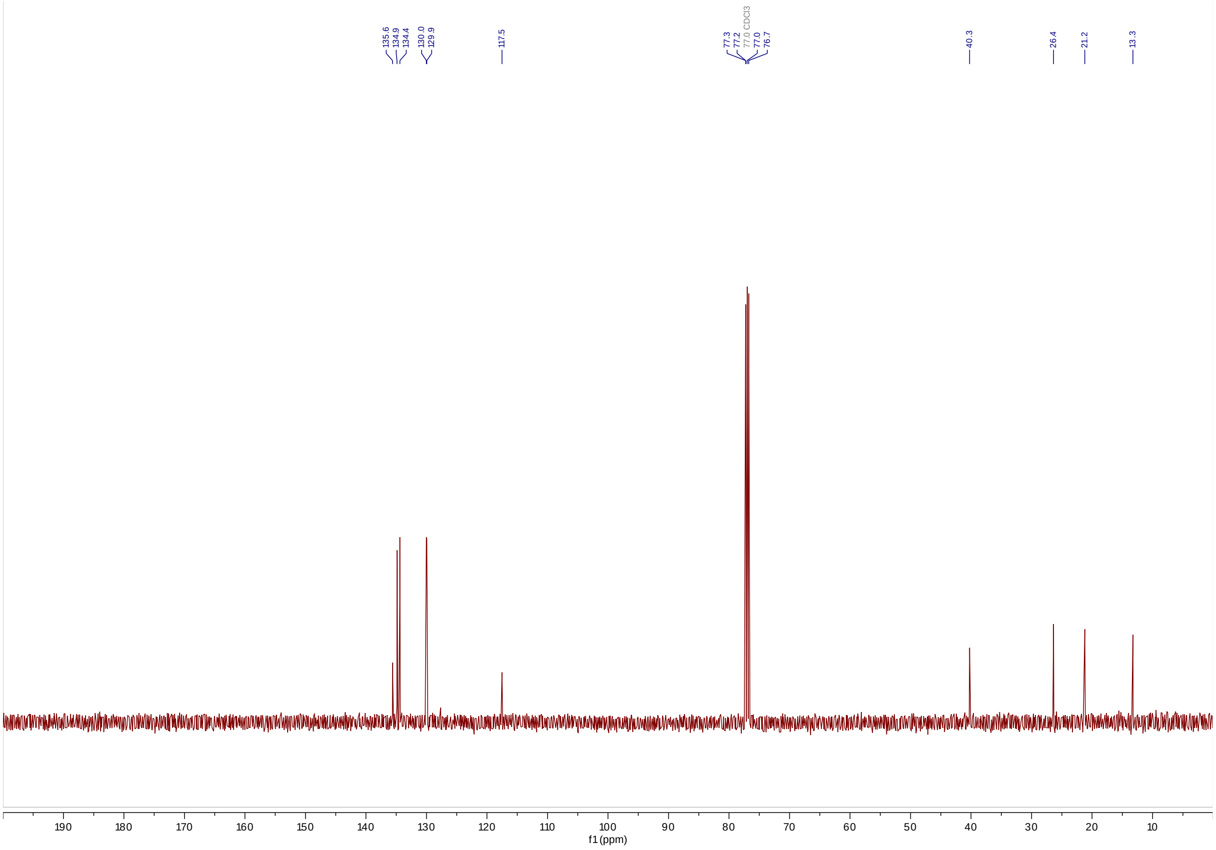

^{19}F-NMR (282.21 MHz, $CDCl_3$) of **Compound 7** (see Procedure)

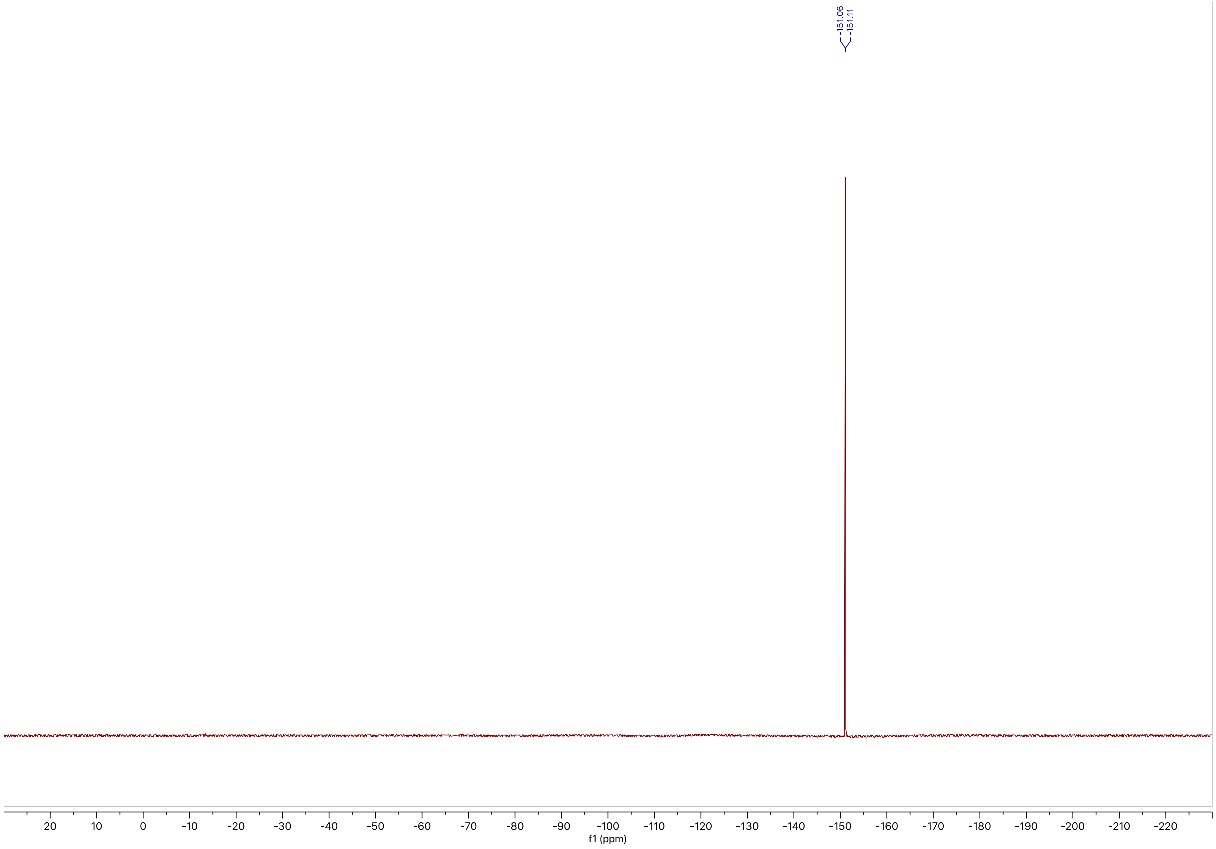

^{1}H-NMR (499.64 MHz, $CDCl_3$): of **Compound 8** (see Procedure)

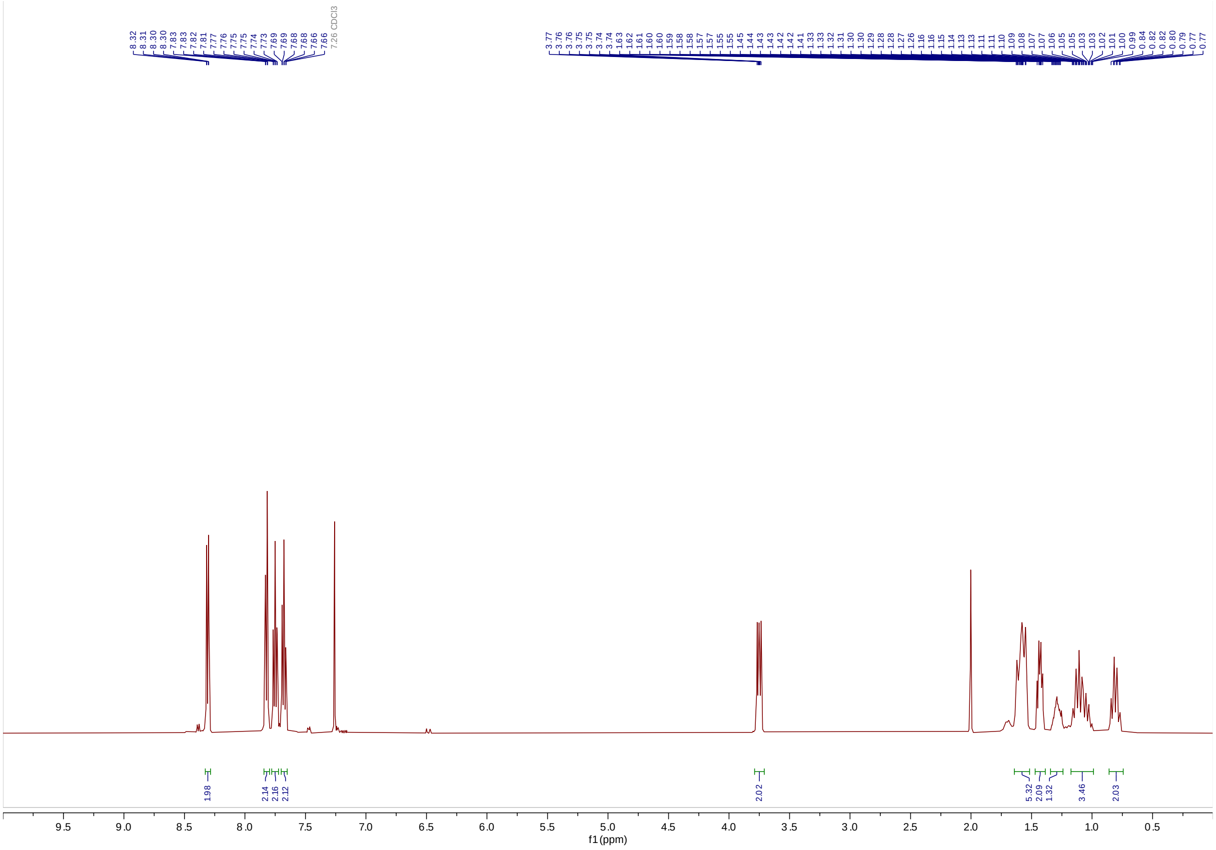

^{13}C-NMR (125.65 MHz, $CDCl_3$) of **Compound 8** (see Procedure)

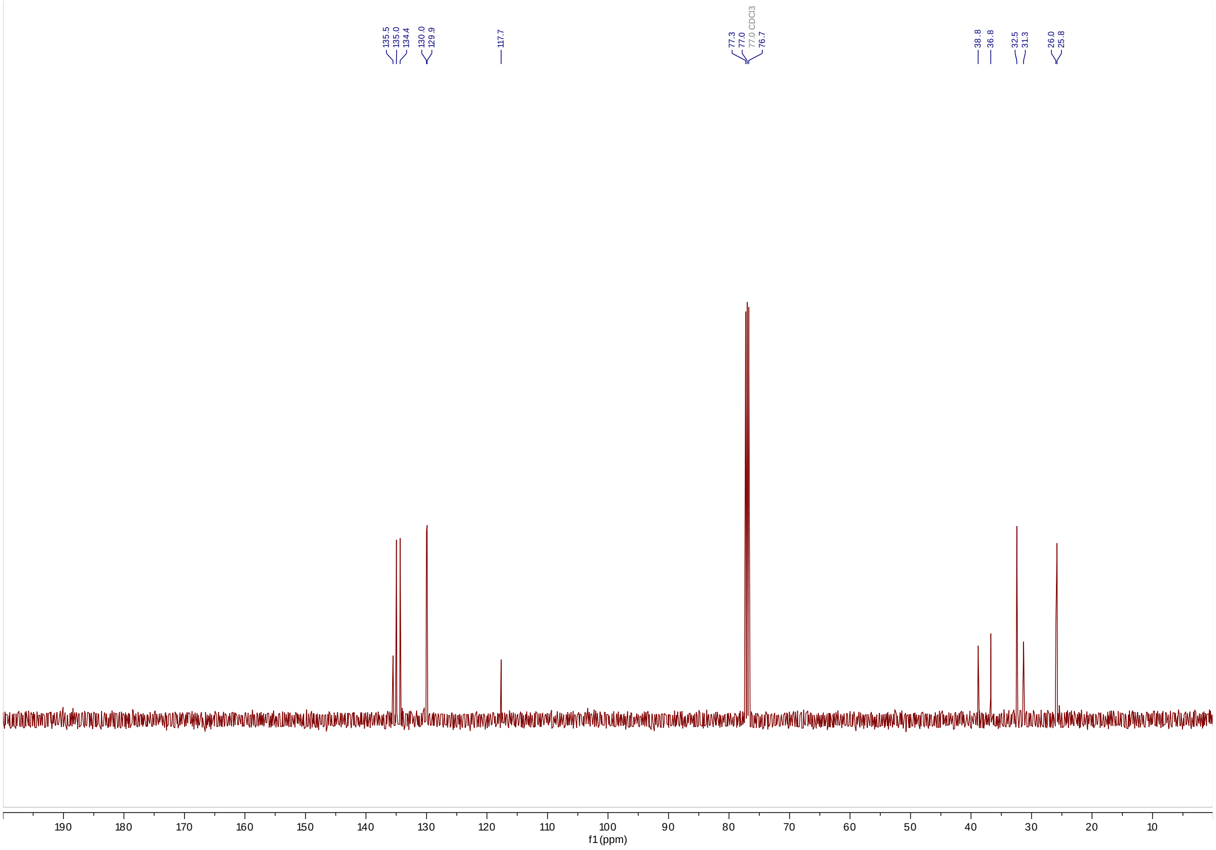

19F-NMR (282.21 MHz, $CDCl_3$) of **Compound 8** (see Procedure)

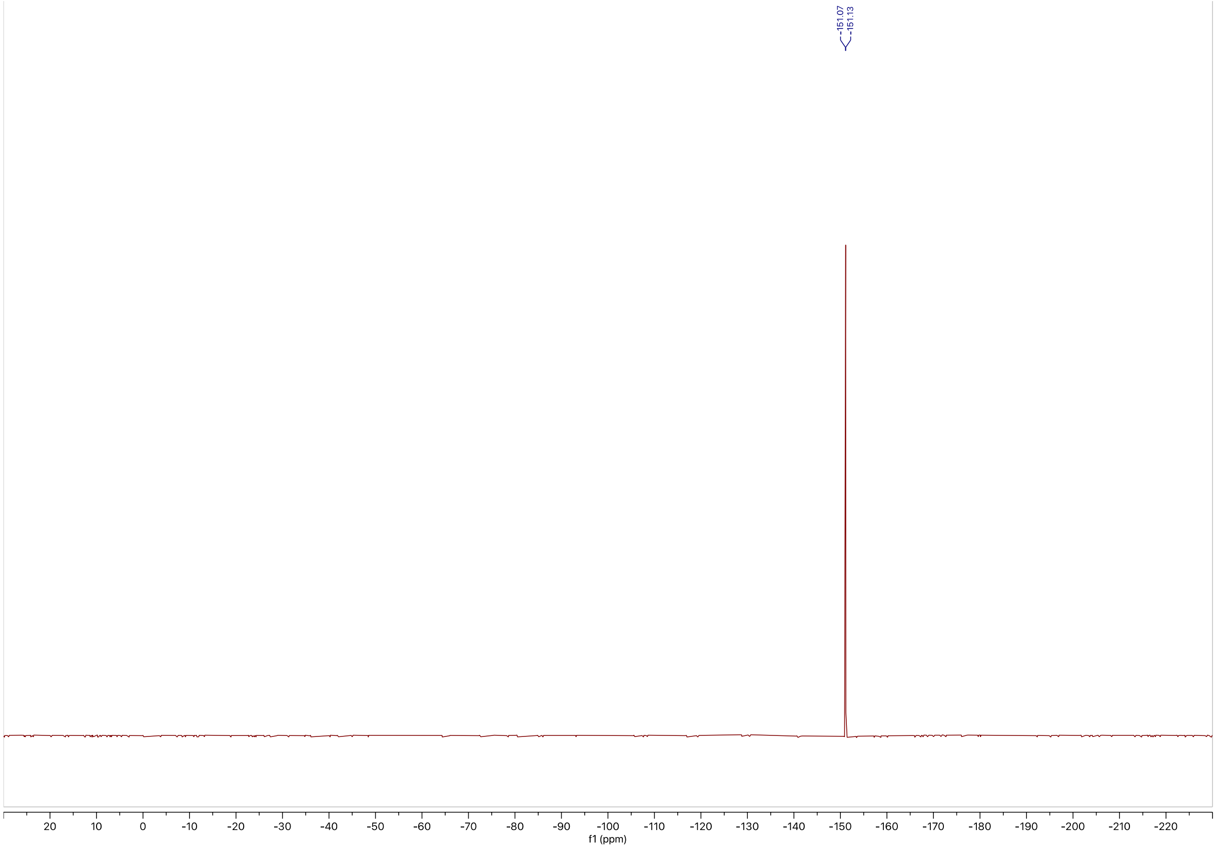

^{1}H-NMR (499.64 MHz, $CDCl_3$): of **Compound 9** (see Procedure)

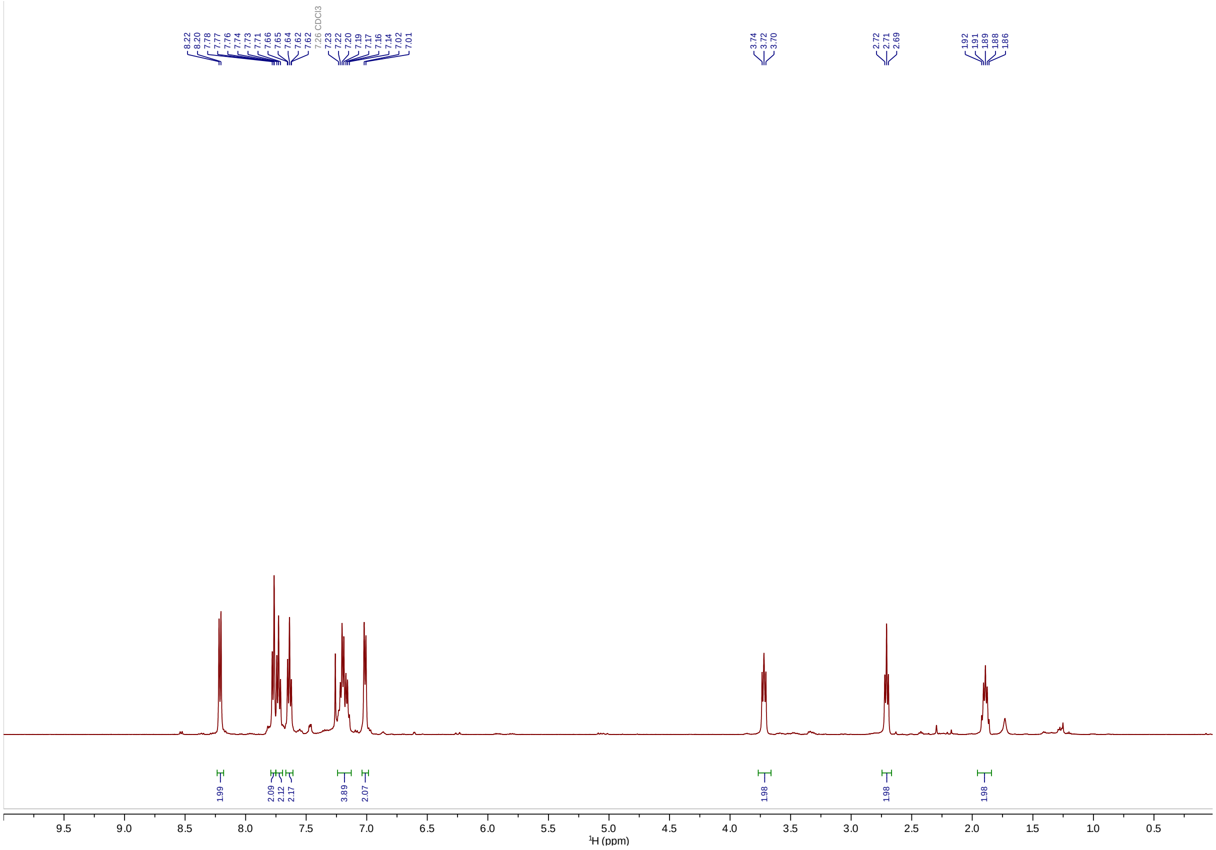

^{13}C-NMR (125.65 MHz, $CDCl_3$) of **Compound 9** (see Procedure)

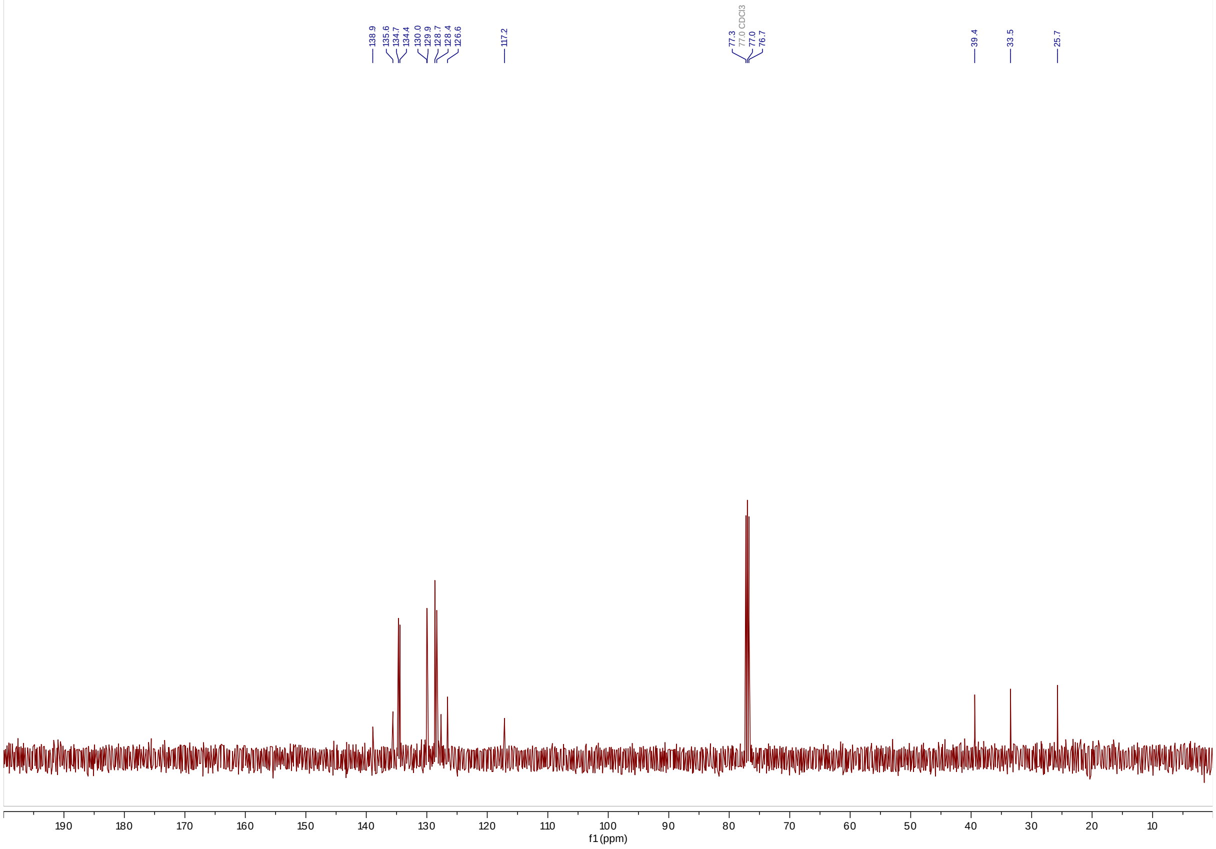

^{19}F-NMR (282.21 MHz, $CDCl_3$) of **Compound 9** (see Procedure)

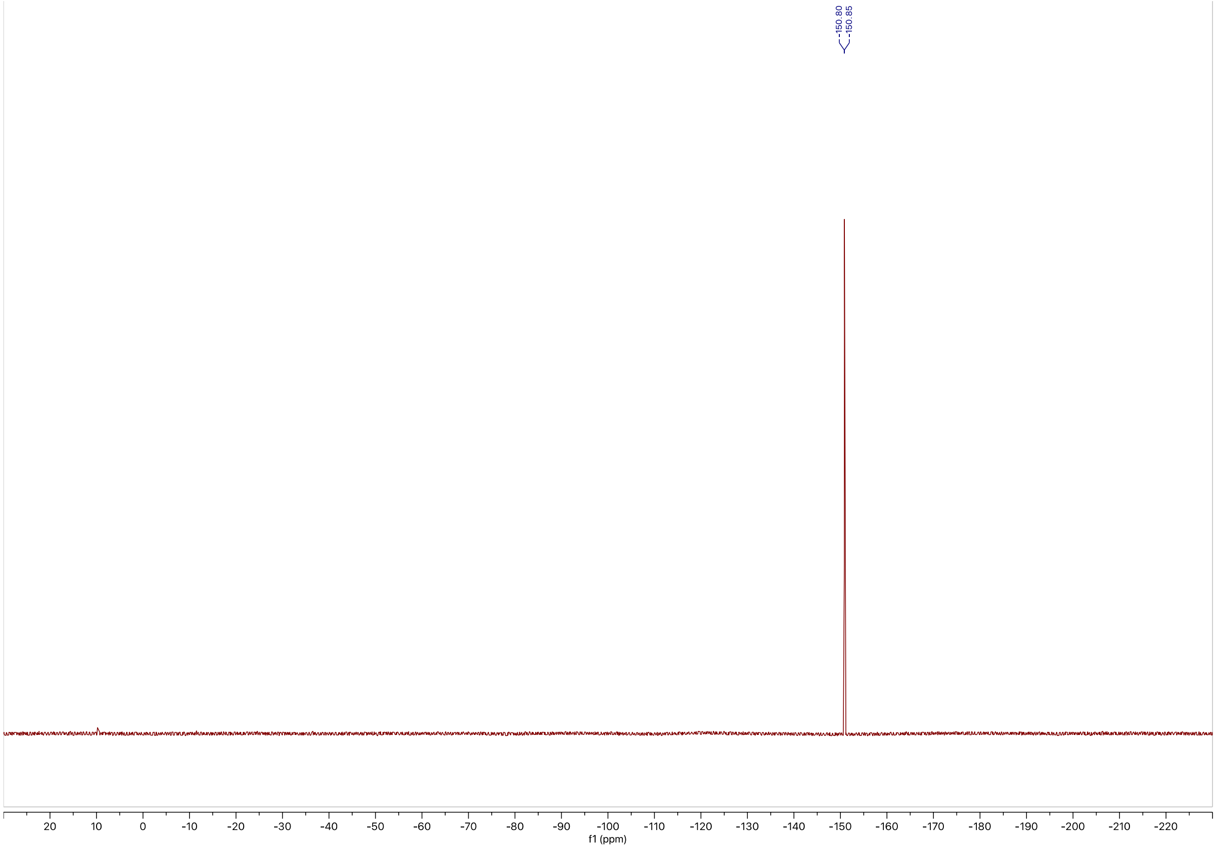

^{1}H-NMR (499.64 MHz, $CDCl_3$): of **Compound 10** (see Procedure)

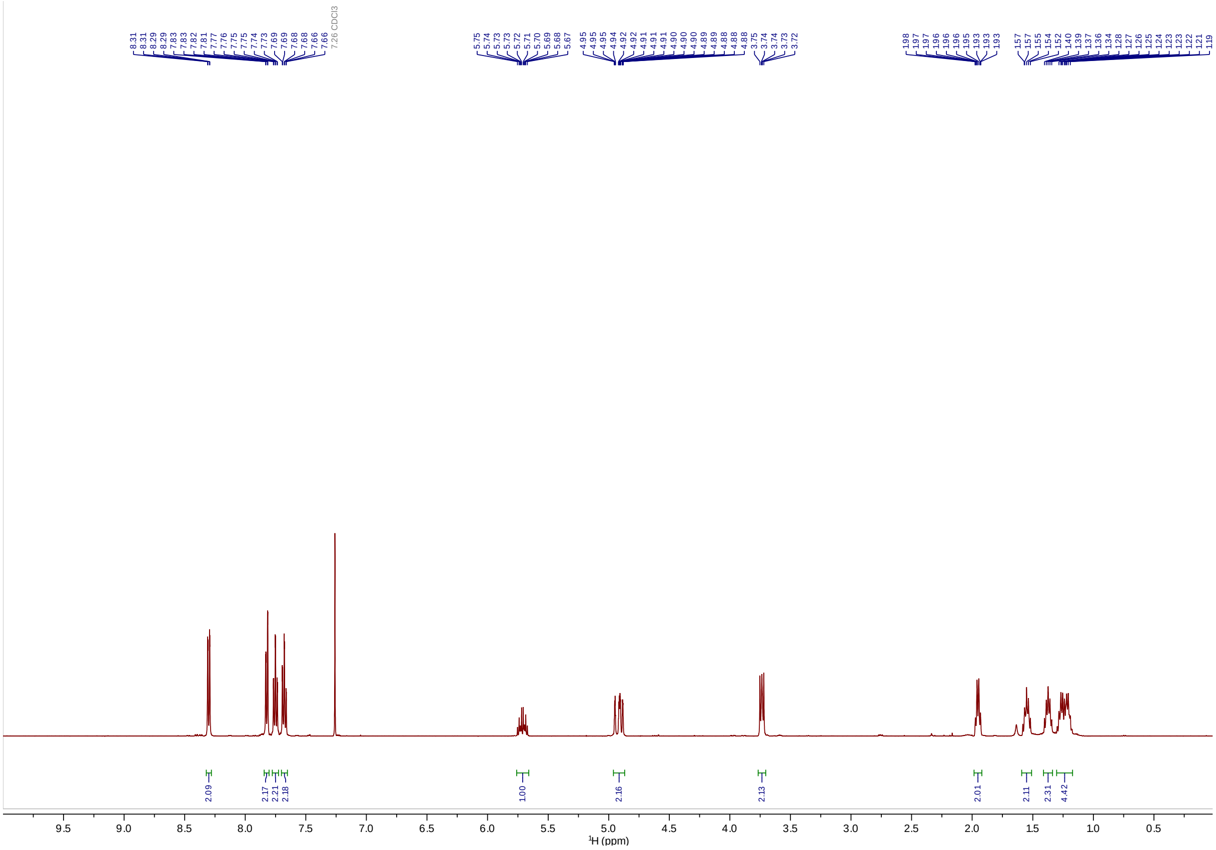

^{13}C-NMR (125.65 MHz, $CDCl_3$) of **Compound 10** (see Procedure)

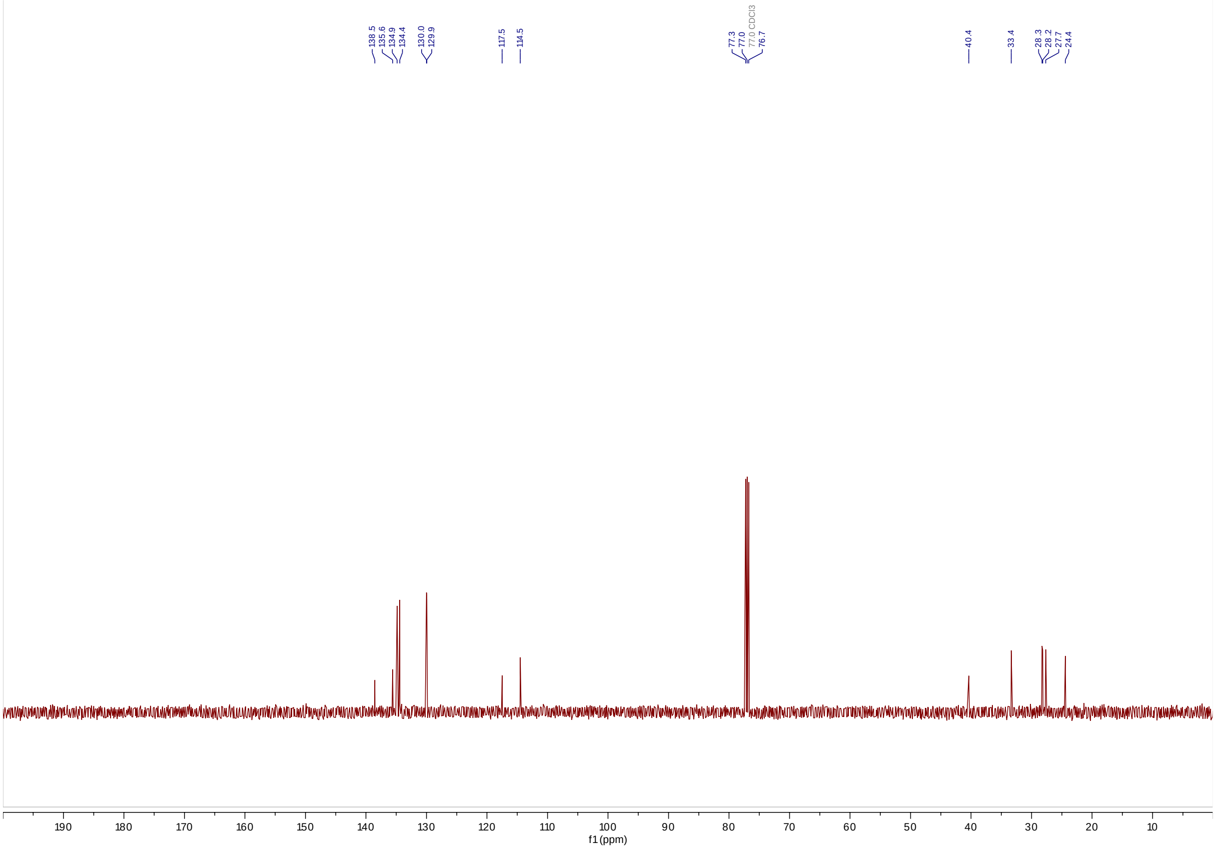

^{19}F-NMR (282.21 MHz, $CDCl_3$) of **Compound 10** (see Procedure)

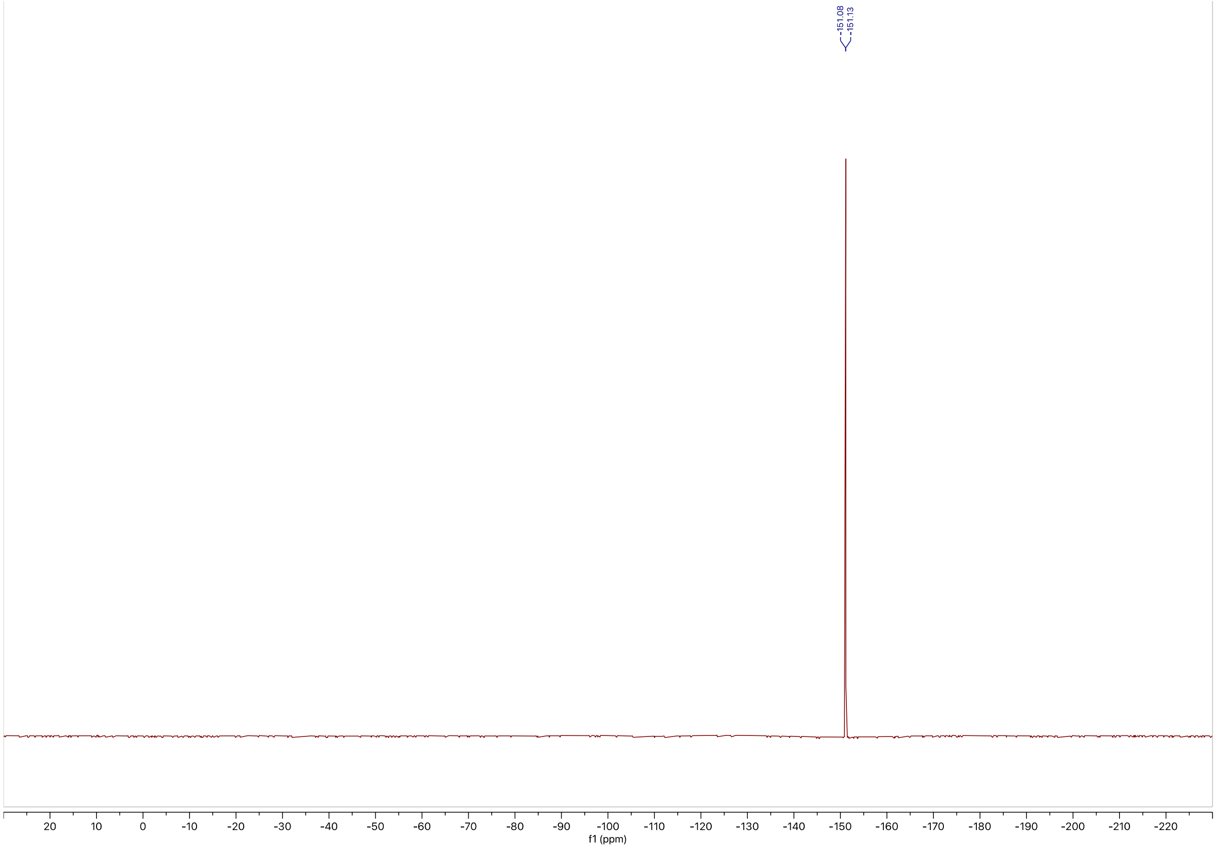

^{1}H-NMR (499.64 MHz, $CDCl_3$): of **Compound 11** (see Procedure)

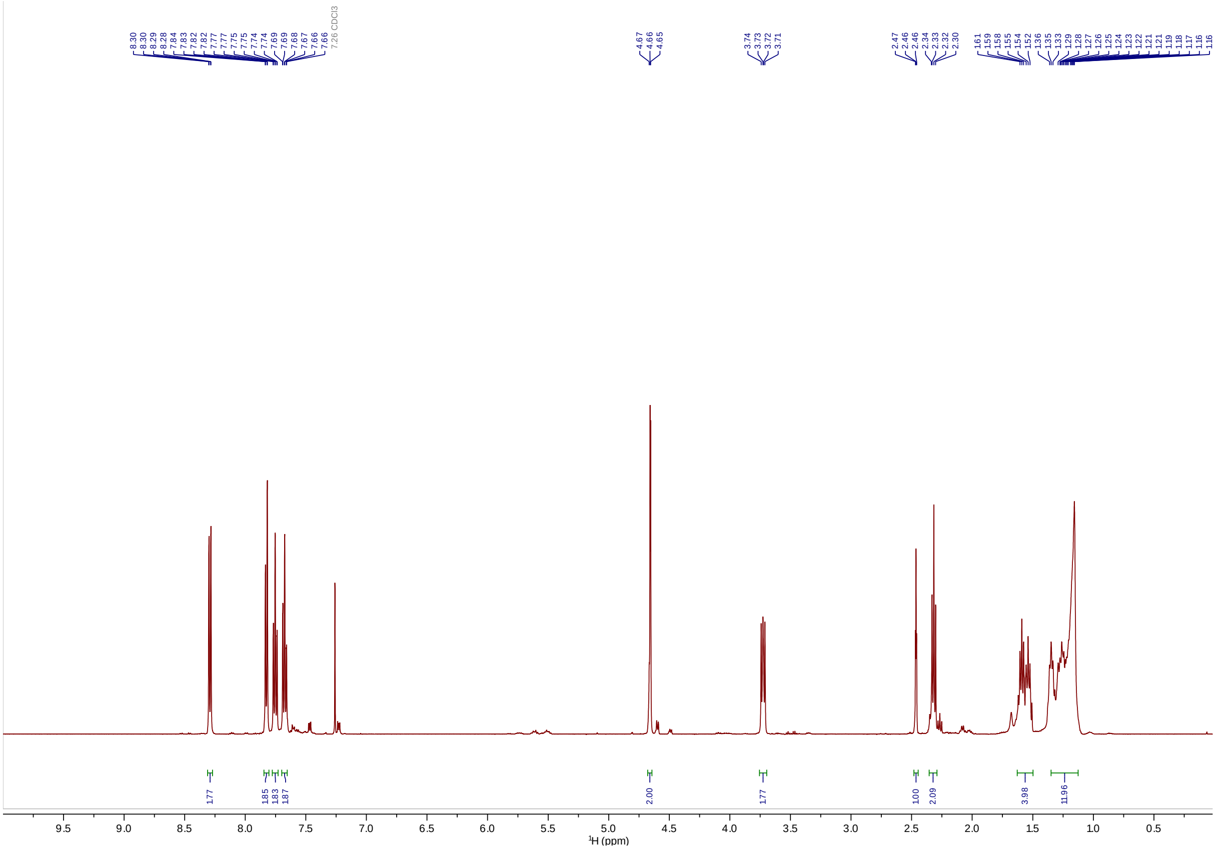

^{13}C-NMR (125.65 MHz, $CDCl_3$) of **Compound 11** (see Procedure)

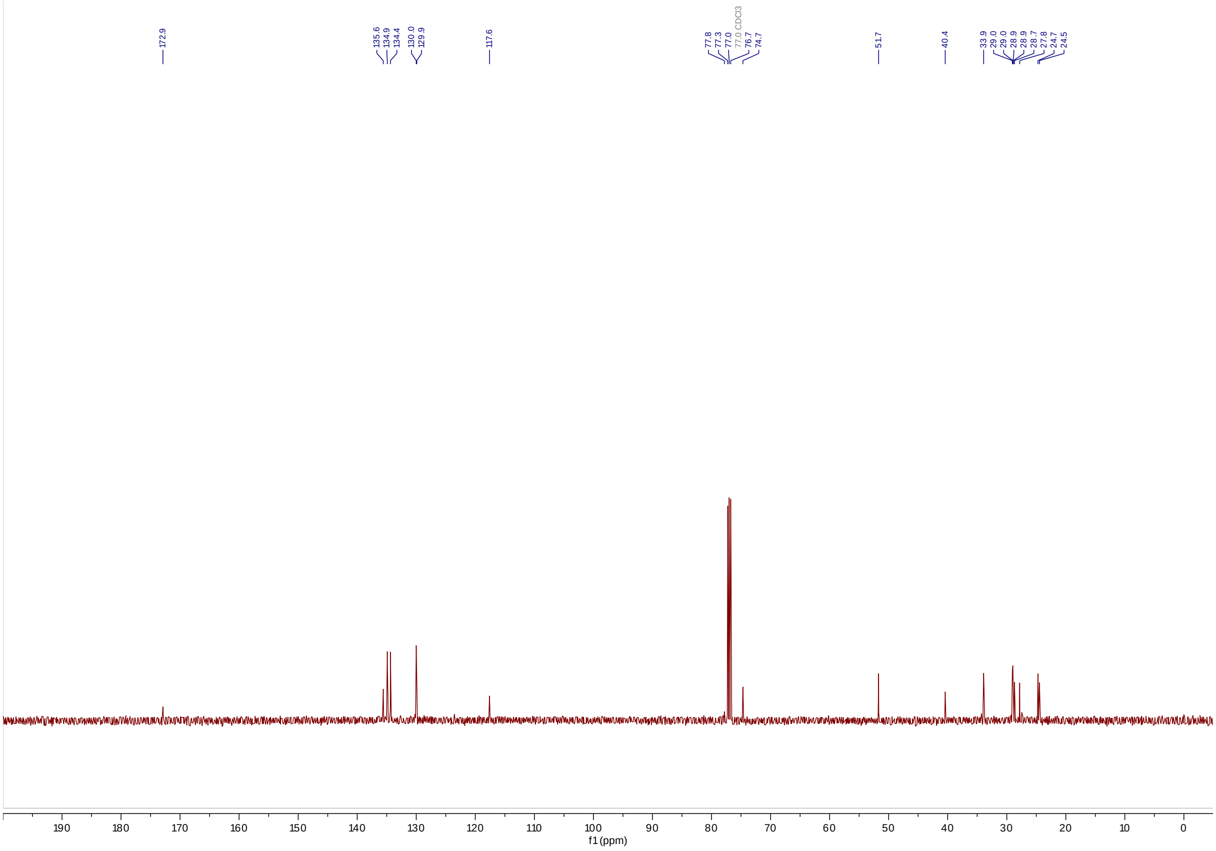

^{19}F-NMR (282.21 MHz, $CDCl_3$) of **Compound 11** (see Procedure)

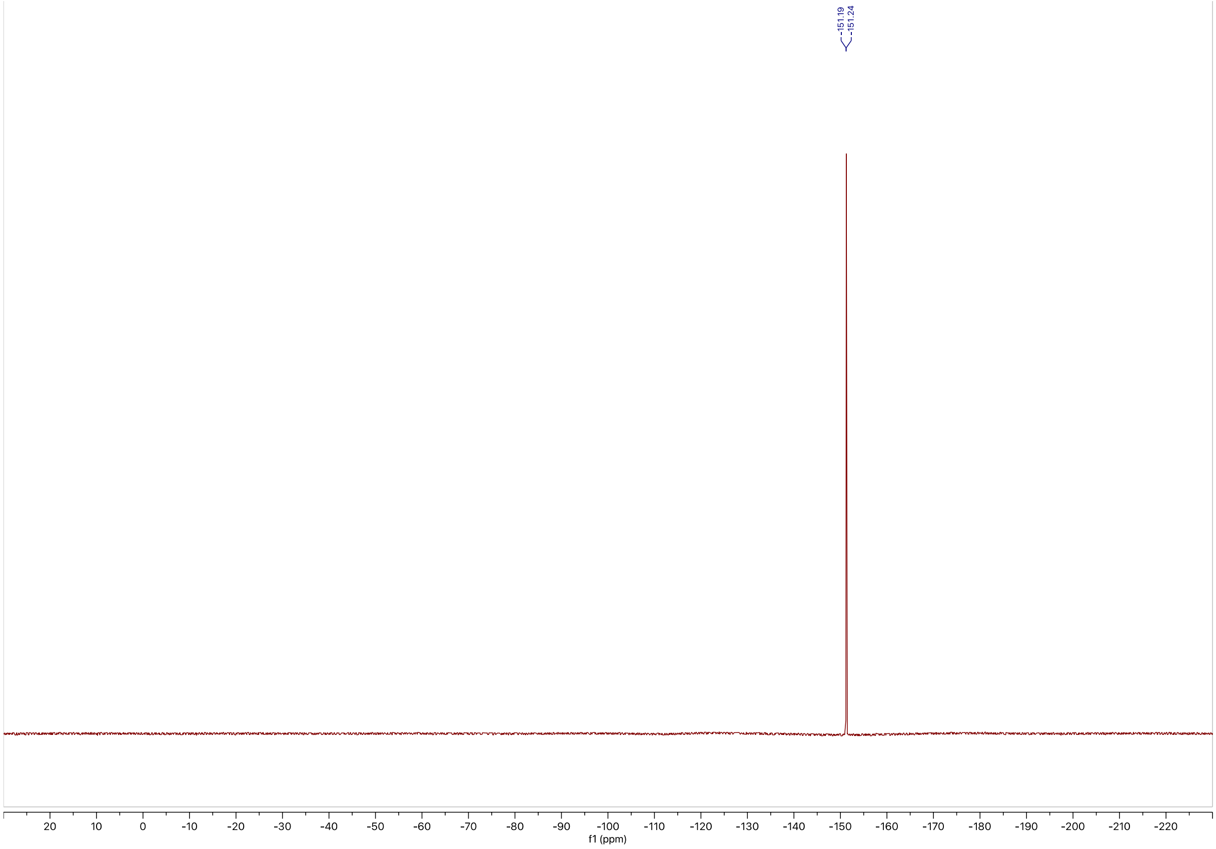

^{1}H-NMR (499.64 MHz, $CDCl_3$): of **Compound 12** (see Procedure)

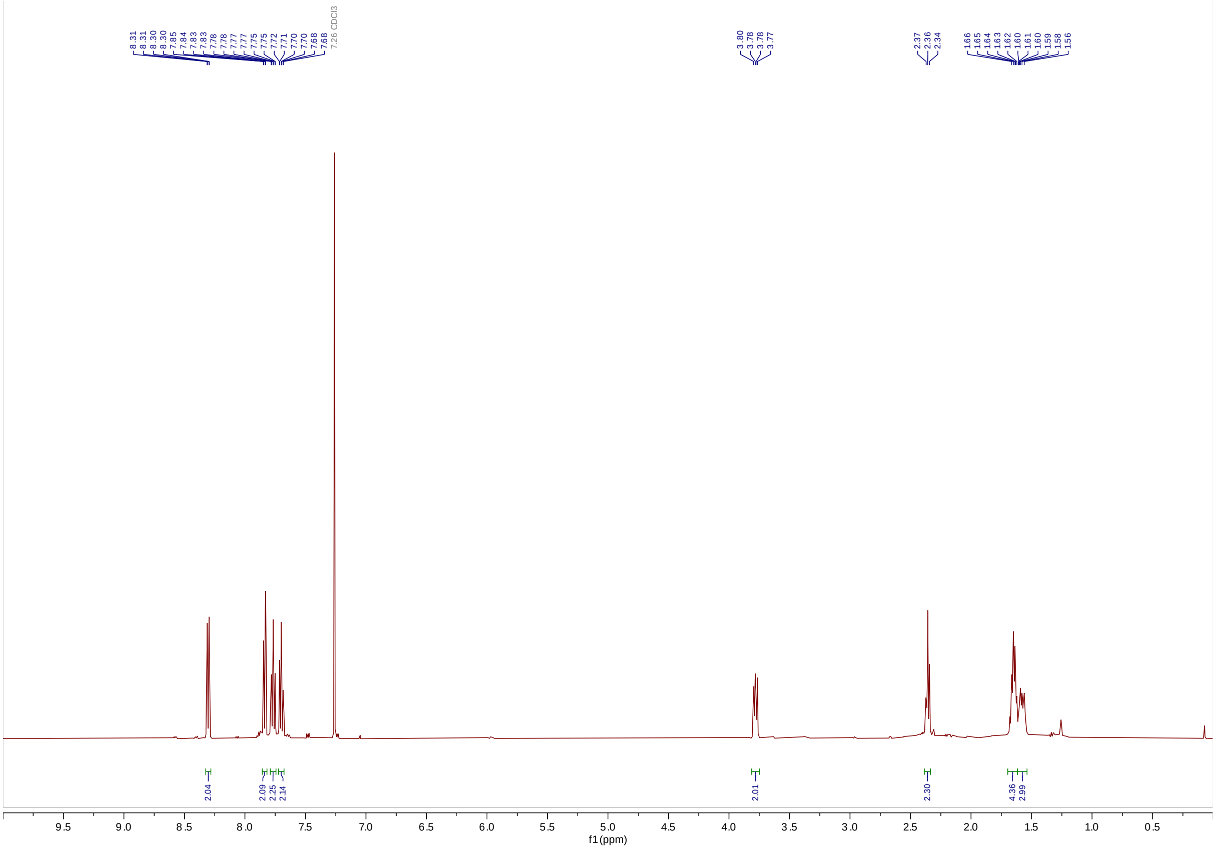

^{13}C-NMR (125.65 MHz, $CDCl_3$) of **Compound 12** (see Procedure)

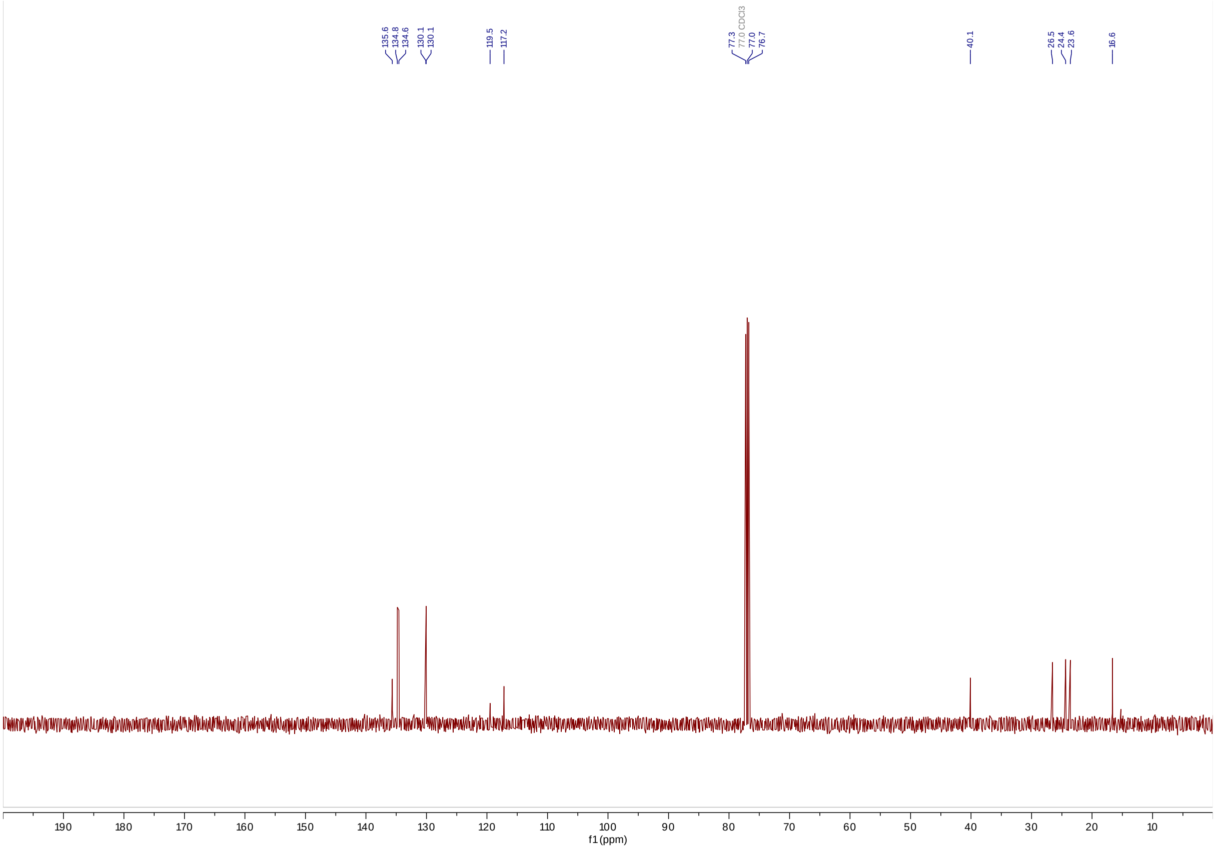

^{19}**F-NMR** (282.21 MHz, $CDCl_3$) of **Compound 12** (see Procedure)

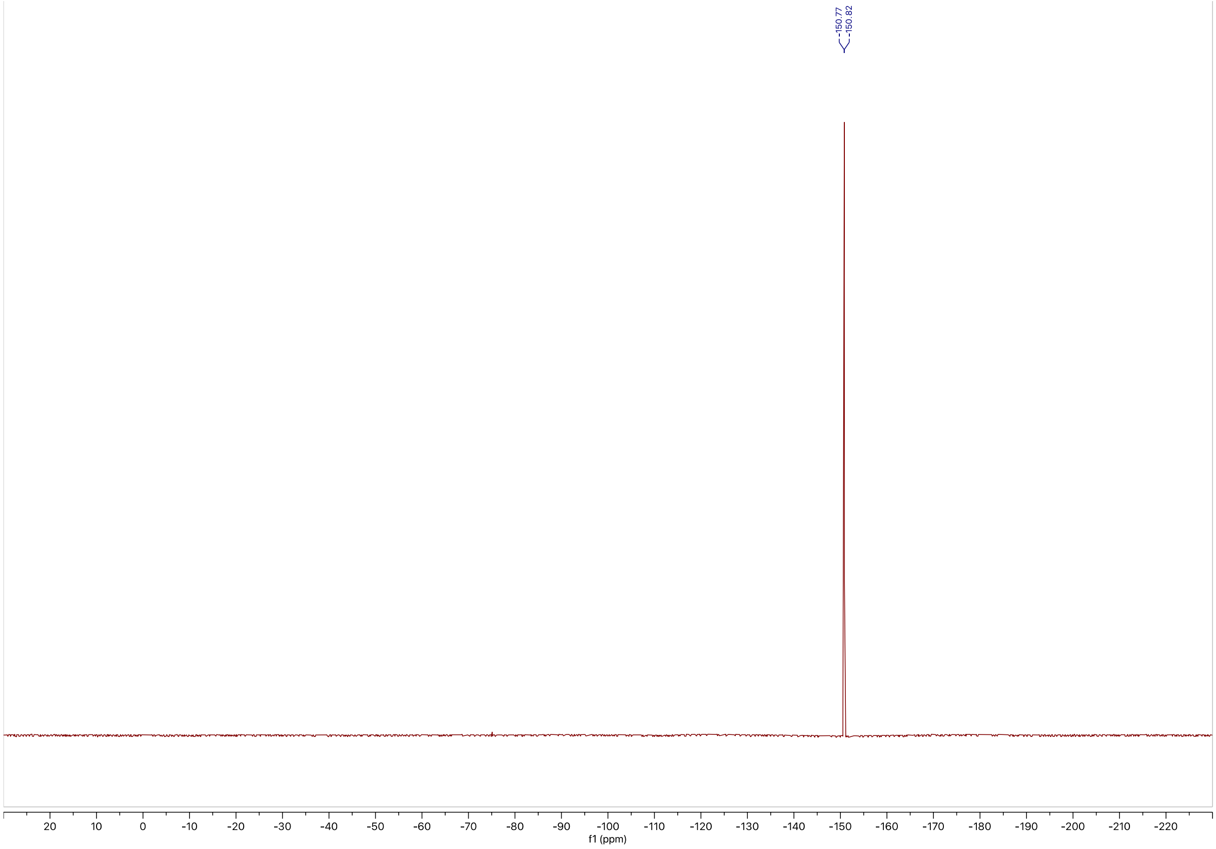

^{1}H-NMR (499.64 MHz, CDCl3): of **Compound 13** (see Procedure)

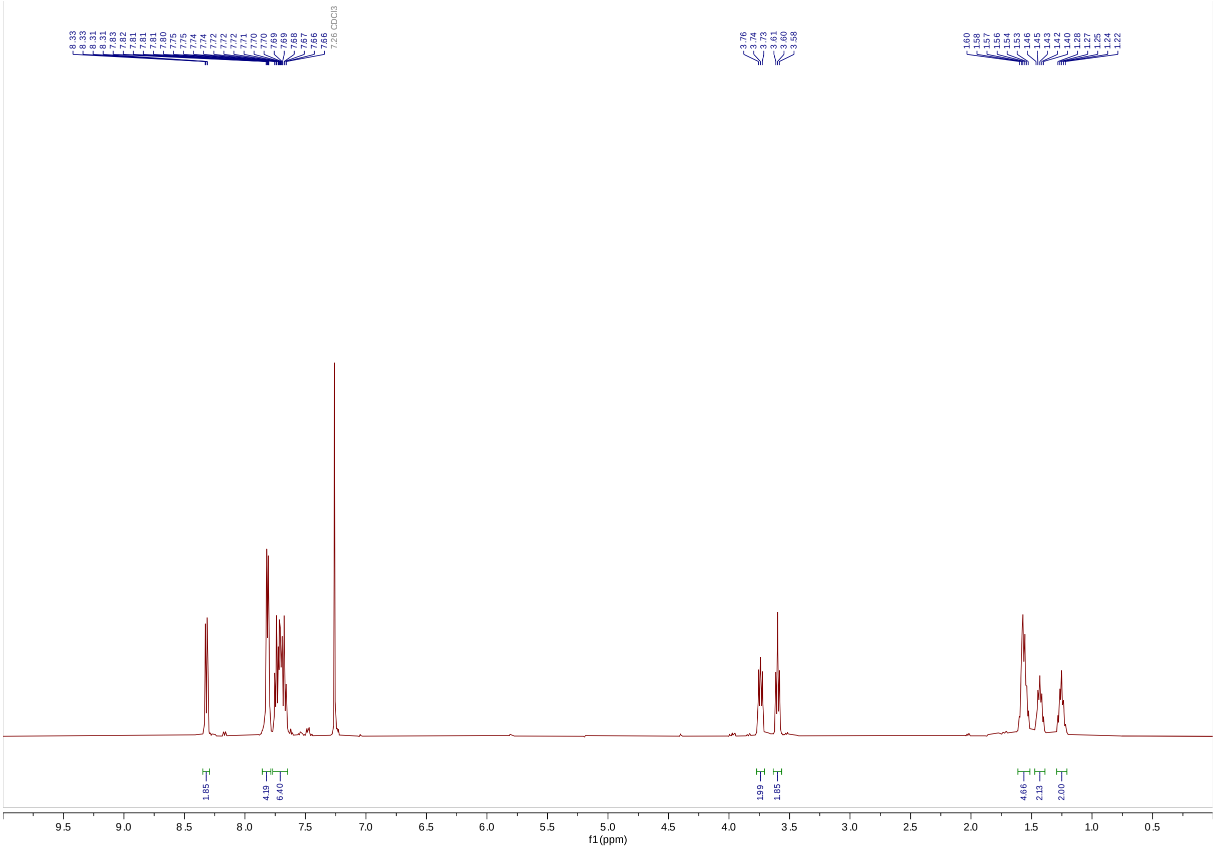

^{13}C-NMR (125.65 MHz, CDCl3) of **Compound 13** (see Procedure)

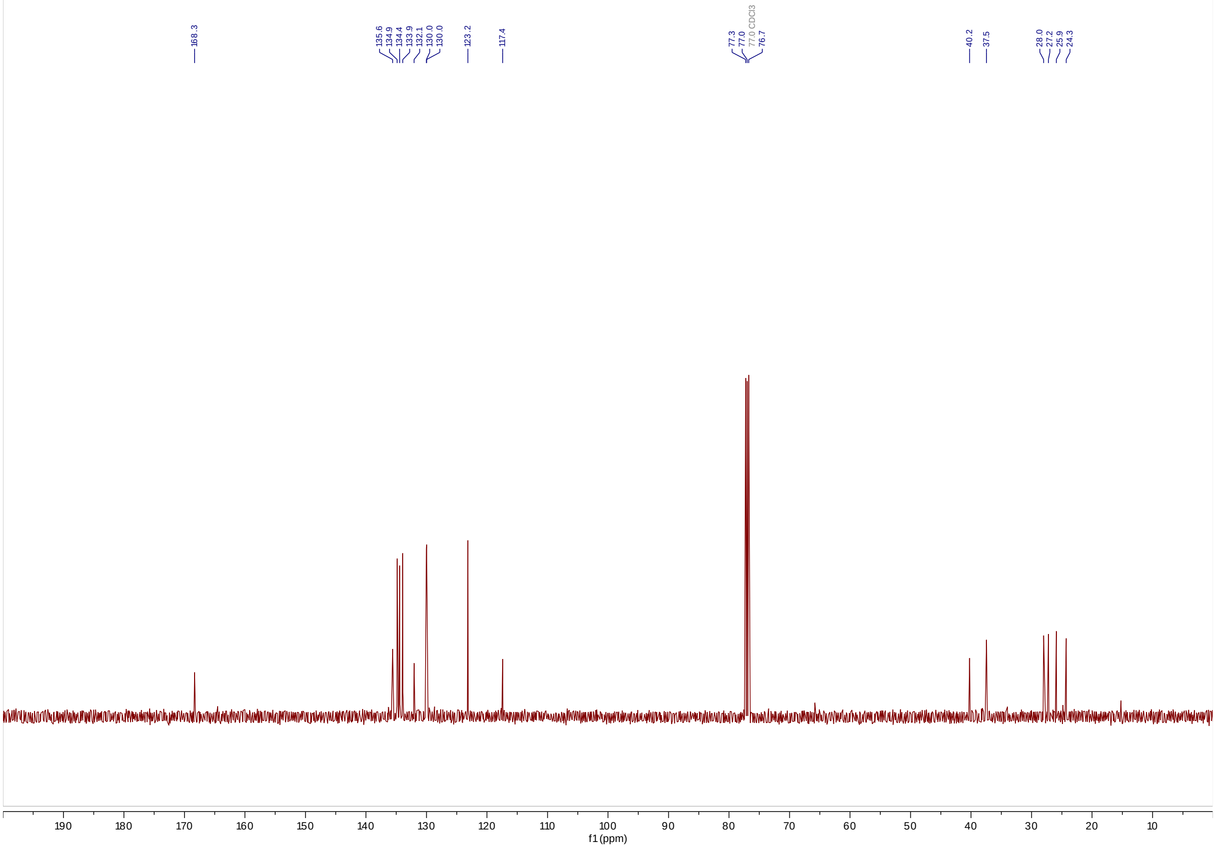

^{19}F-NMR (282.21 MHz, $CDCl_3$) of **Compound 13** (see Procedure)

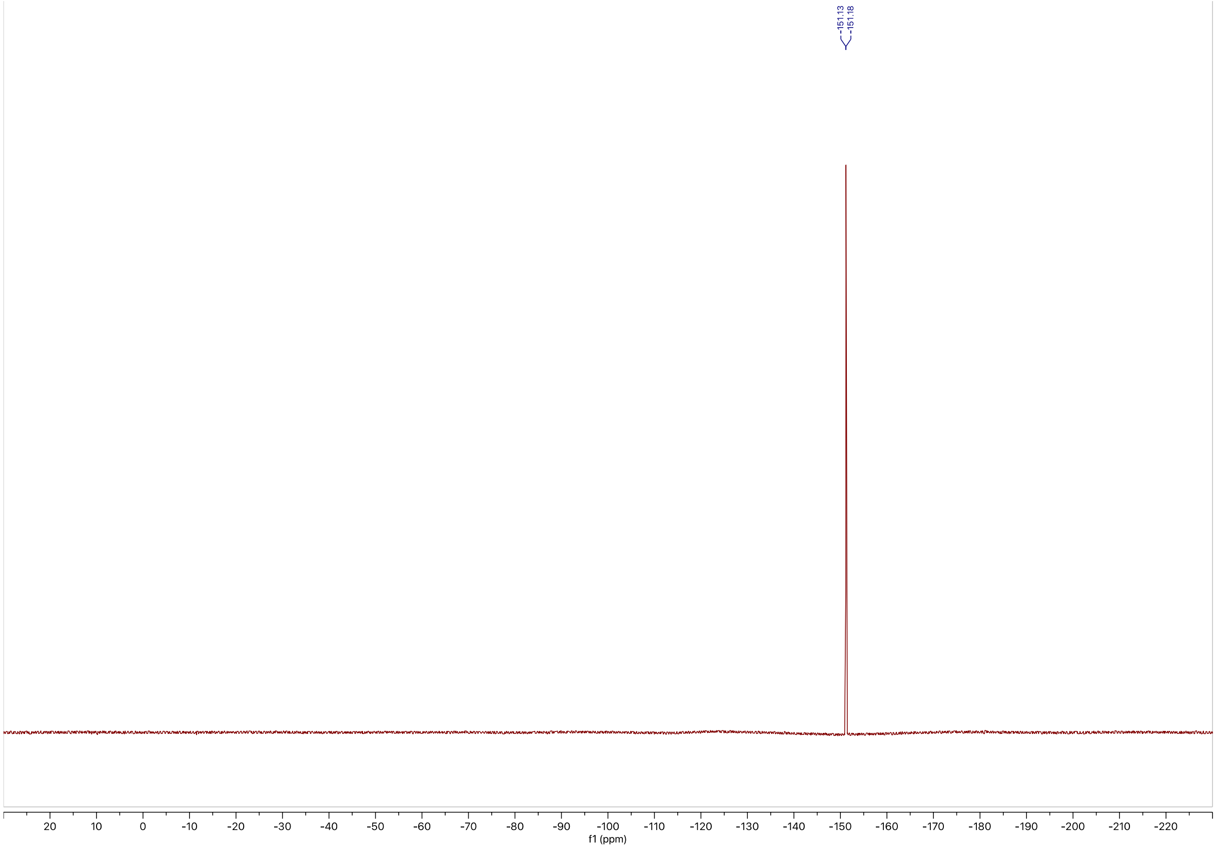

[1]**H-NMR** (499.64 MHz, $CDCl_3$): of **Compound 14** (see Procedure)

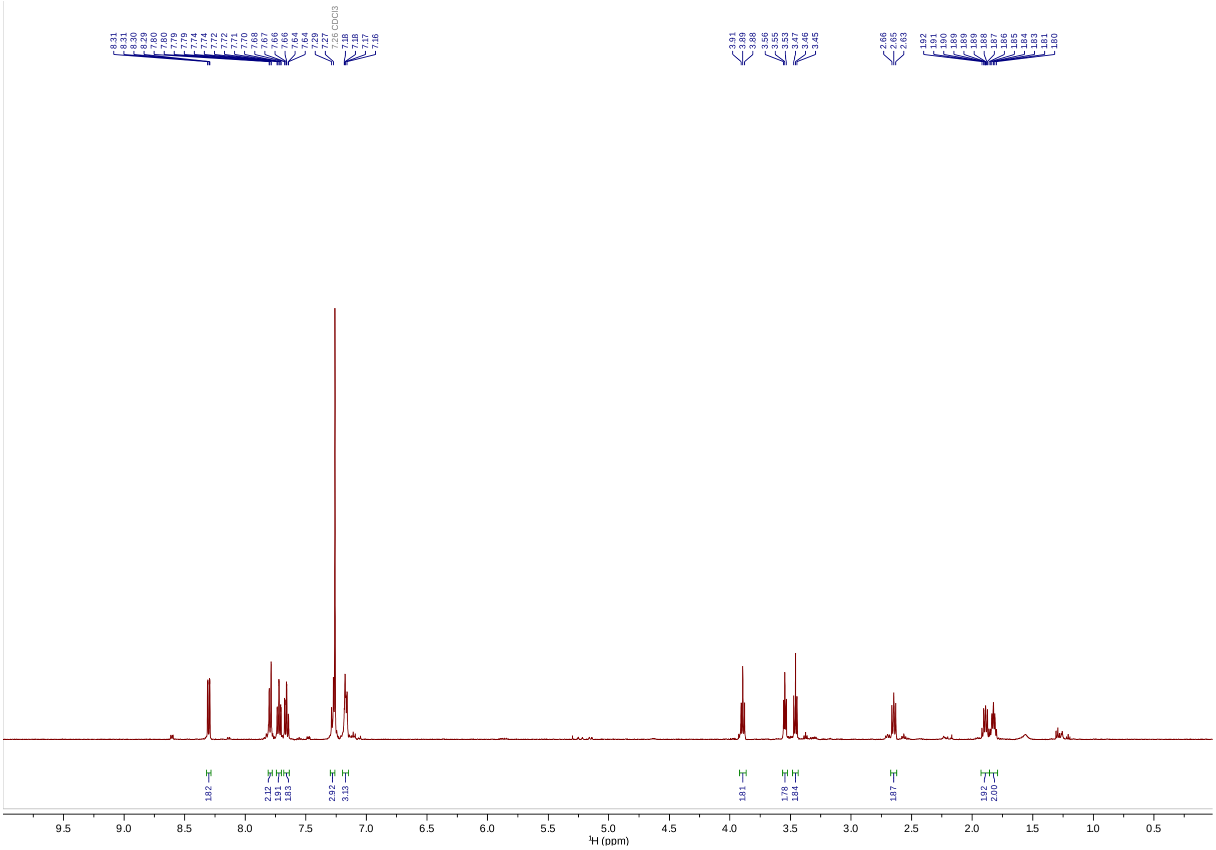

[13]**C-NMR** (100.56 MHz, $CDCl_3$) of **Compound 14** (see Procedure)

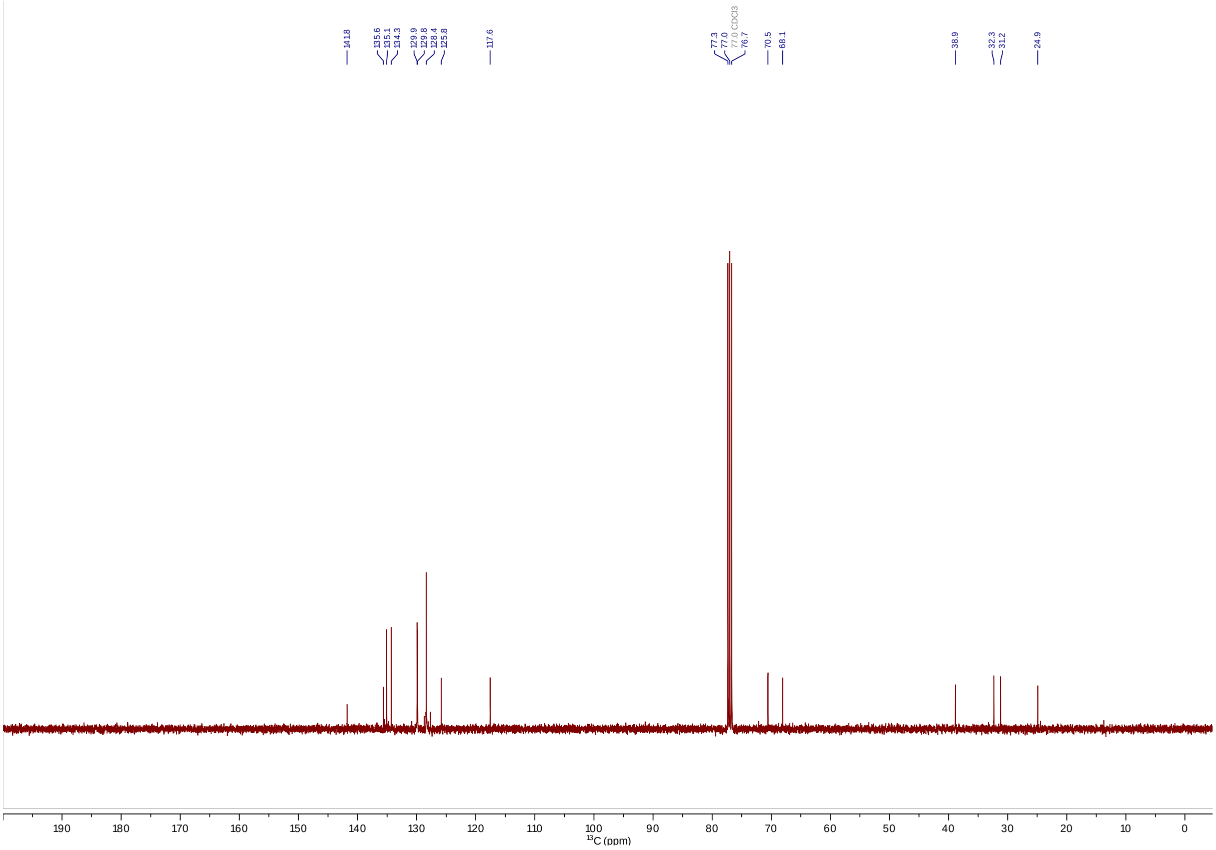

^{19}F-NMR (282.21 MHz, $CDCl_3$) of **Compound 14** (see Procedure)

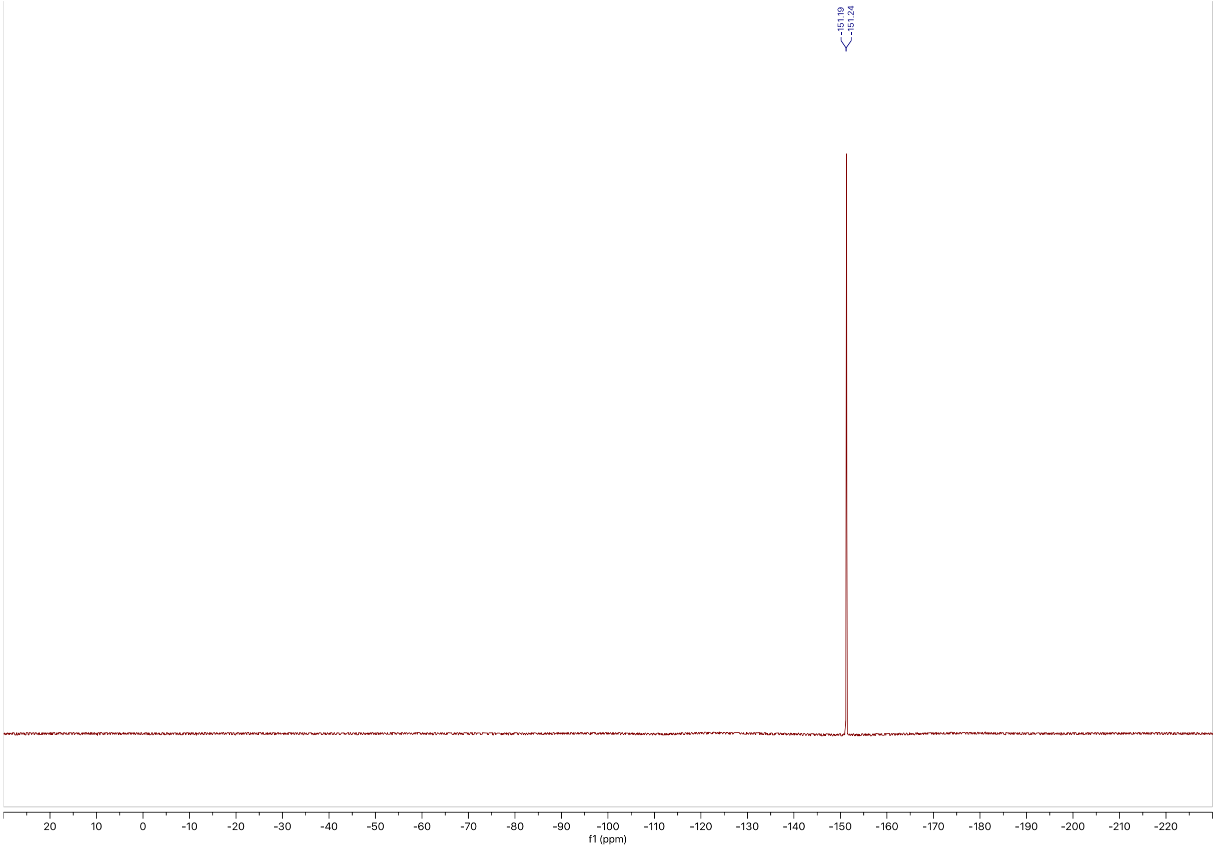

¹H-NMR (499.64 MHz, $CDCl_3$): of **Compound 15** (see Procedure)

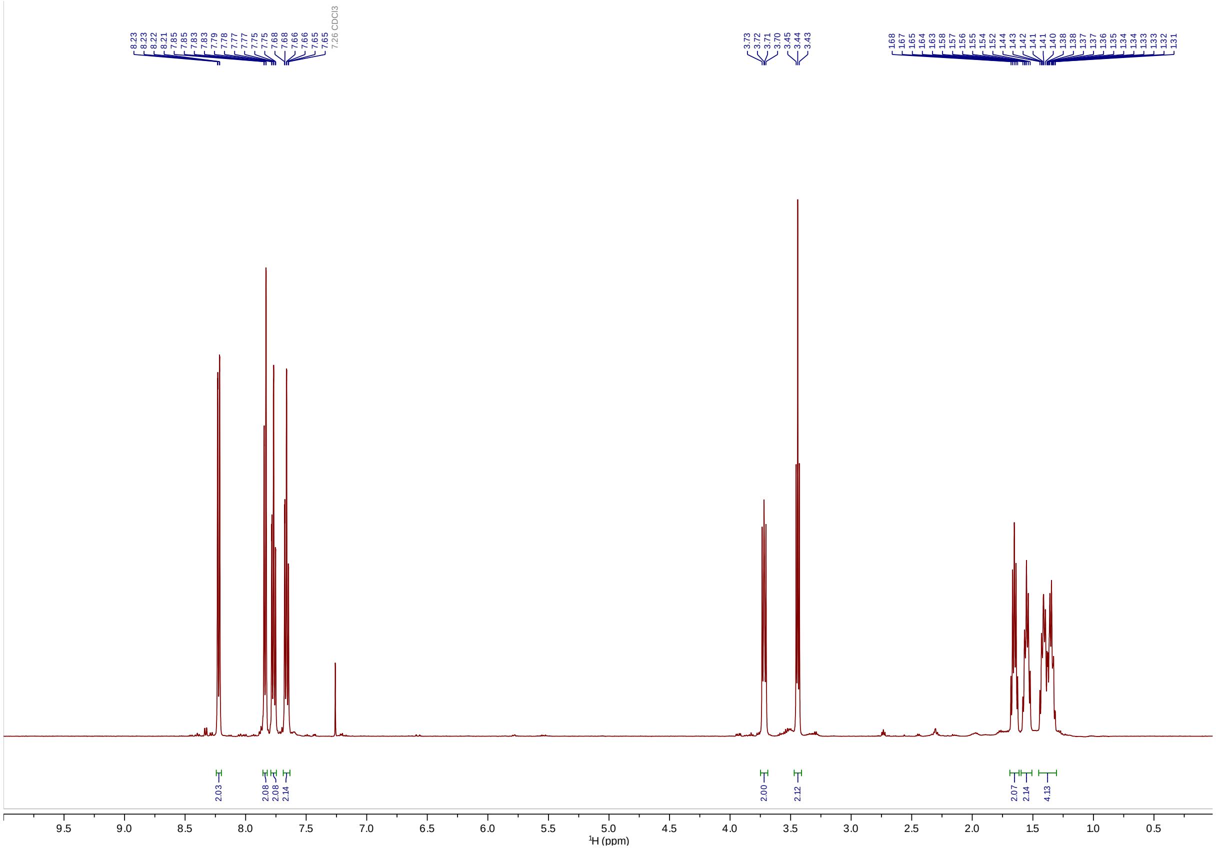

¹³C-NMR (125.65 MHz, $CDCl_3$) of **Compound 15** (see Procedure)

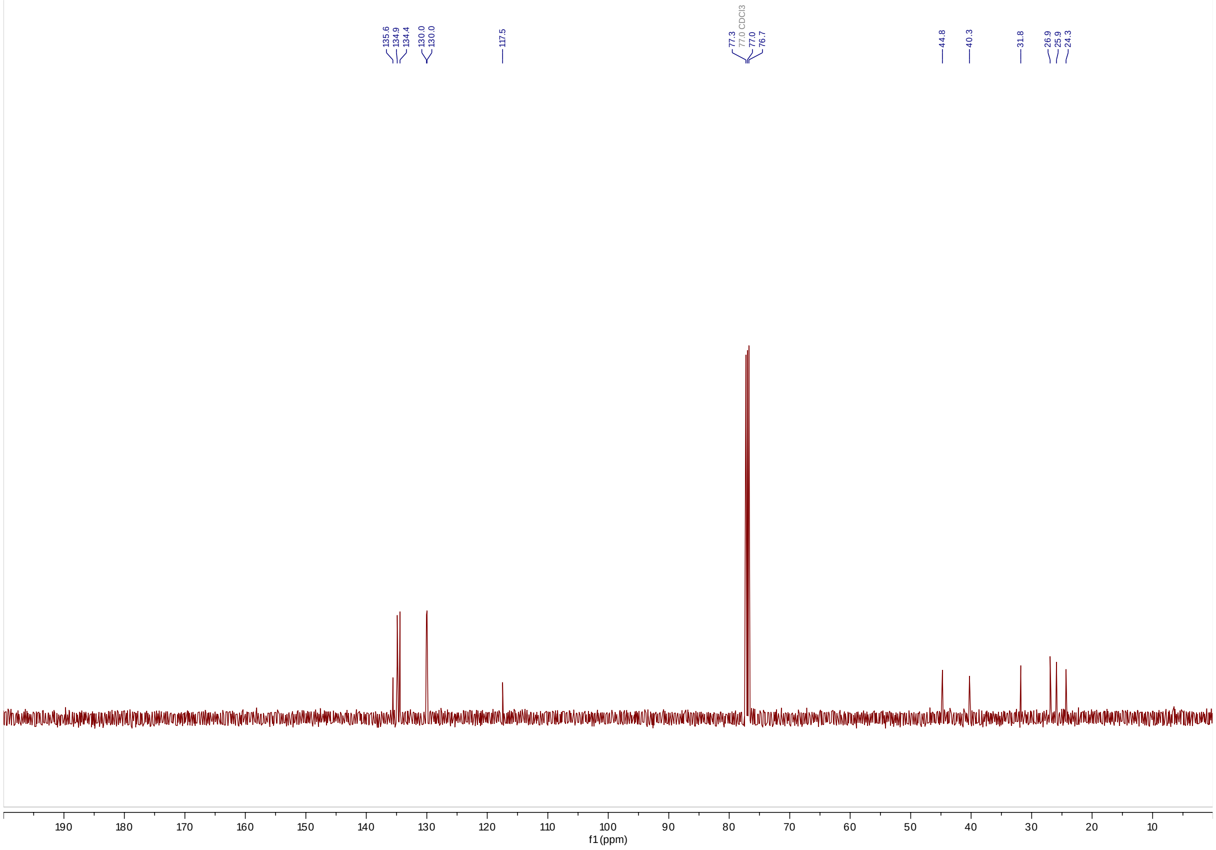

^{19}F-NMR (282.21 MHz, $CDCl_3$) of **Compound 15** (see Procedure)

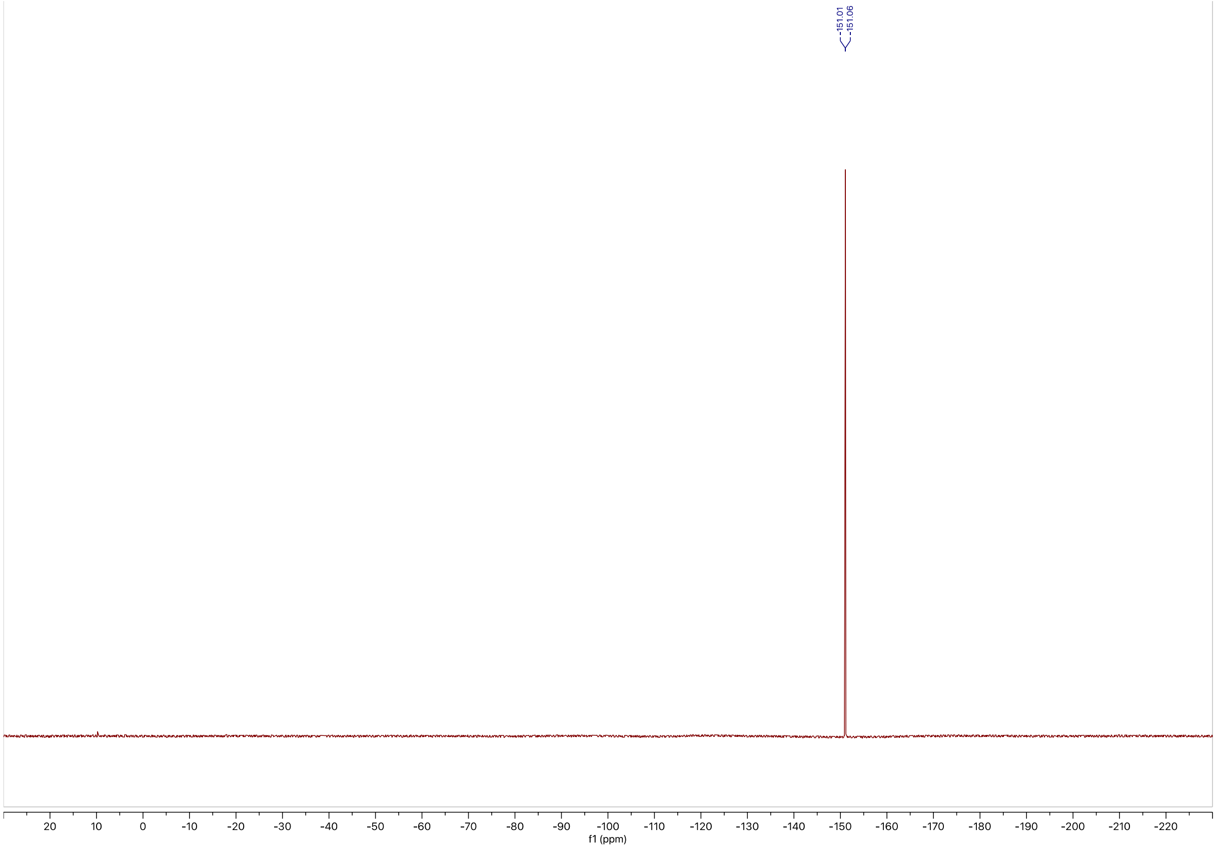

¹H-NMR (499.64 MHz, $CDCl_3$): of **Compound 16** (see Procedure)

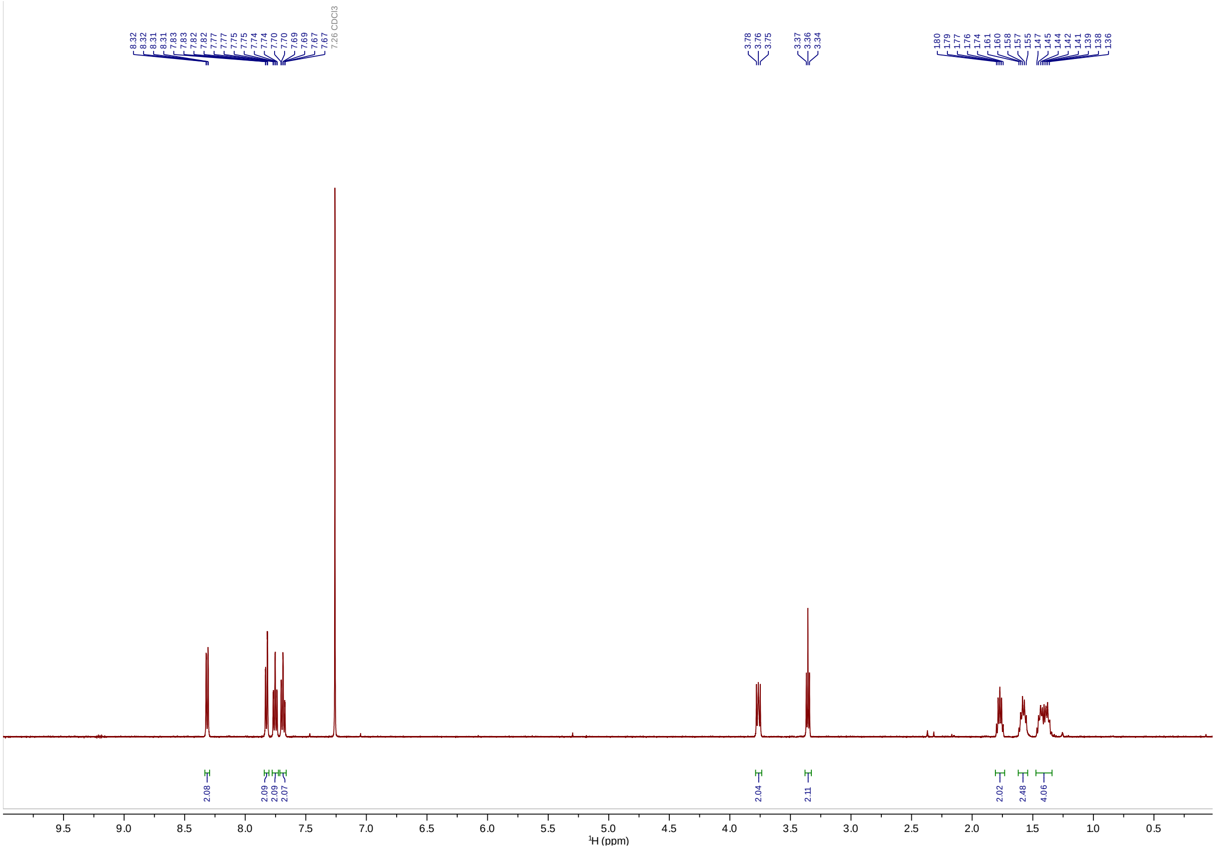

¹³C-NMR (125.65 MHz, $CDCl_3$) of **Compound 16** (see Procedure)

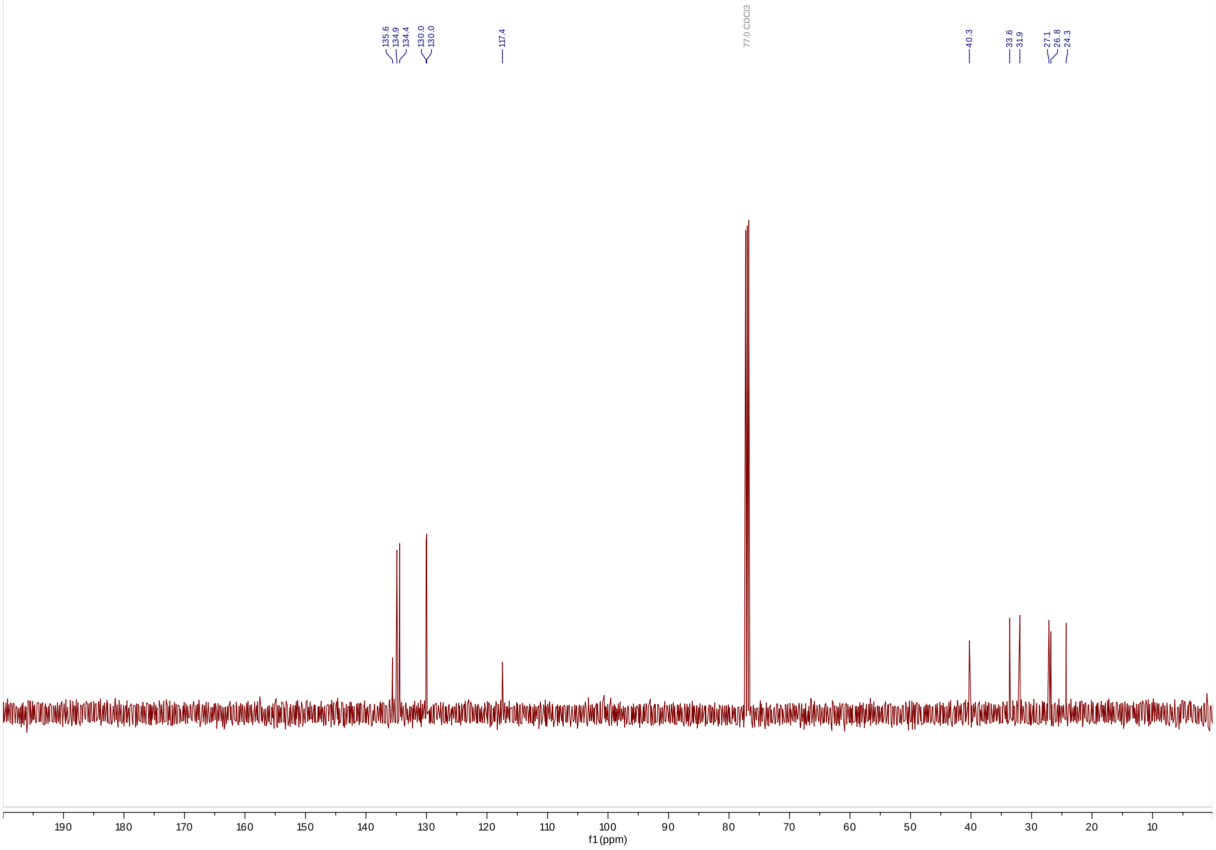

^{19}F-NMR (282.21 MHz, $CDCl_3$) of **Compound 16** (see Procedure)

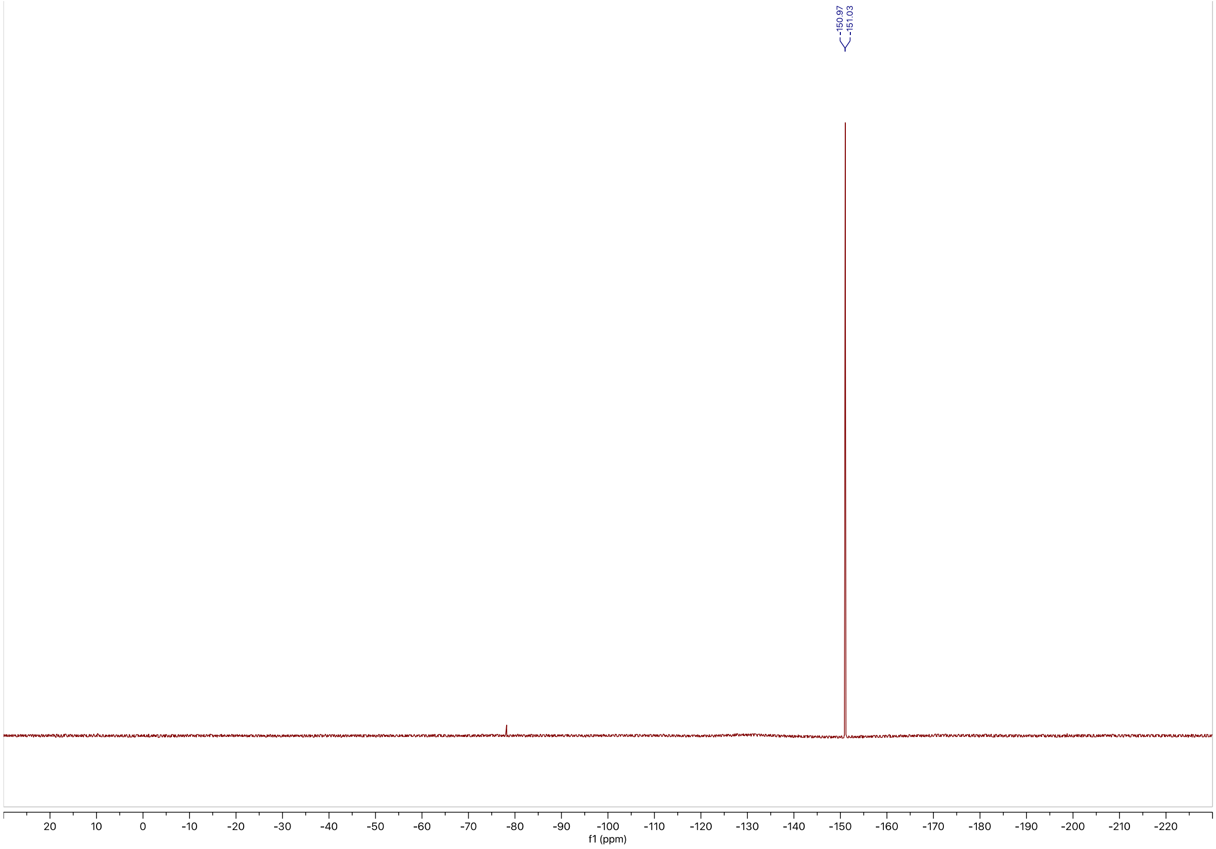

^{1}H-NMR (499.64 MHz, $CDCl_3$): of **Compound 17** (see Procedure)

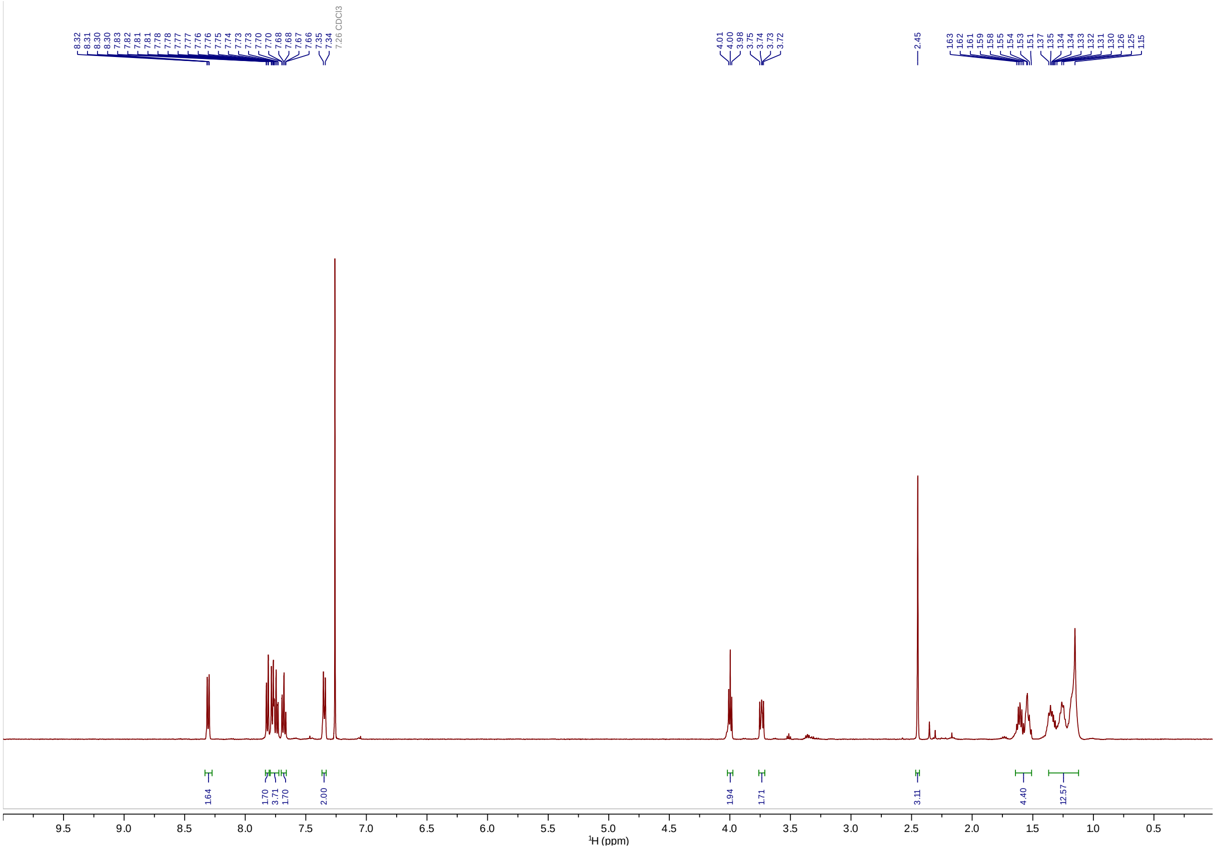

^{13}C-NMR (125.65 MHz, $CDCl_3$) of **Compound 17** (see Procedure)

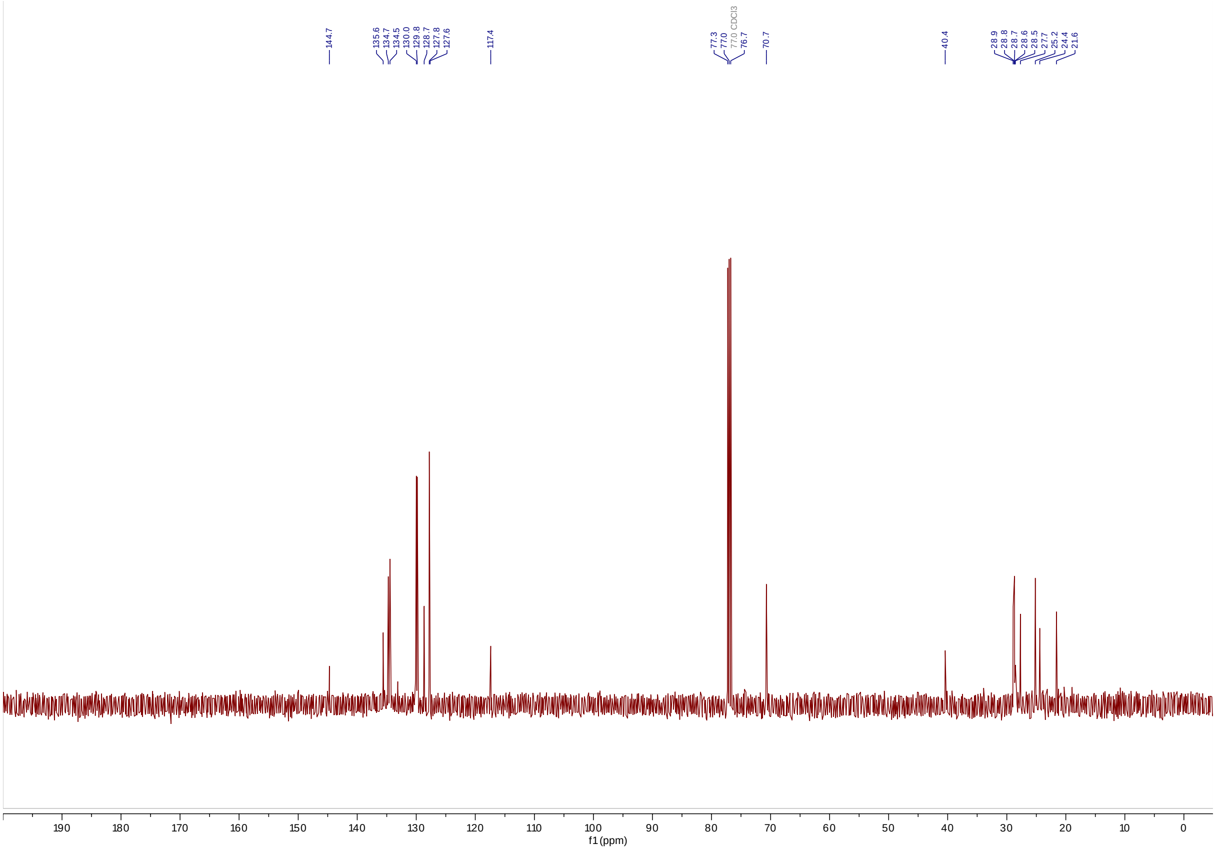

^{19}**F-NMR** (282.21 MHz, $CDCl_3$) of **Compound 17** (see Procedure)

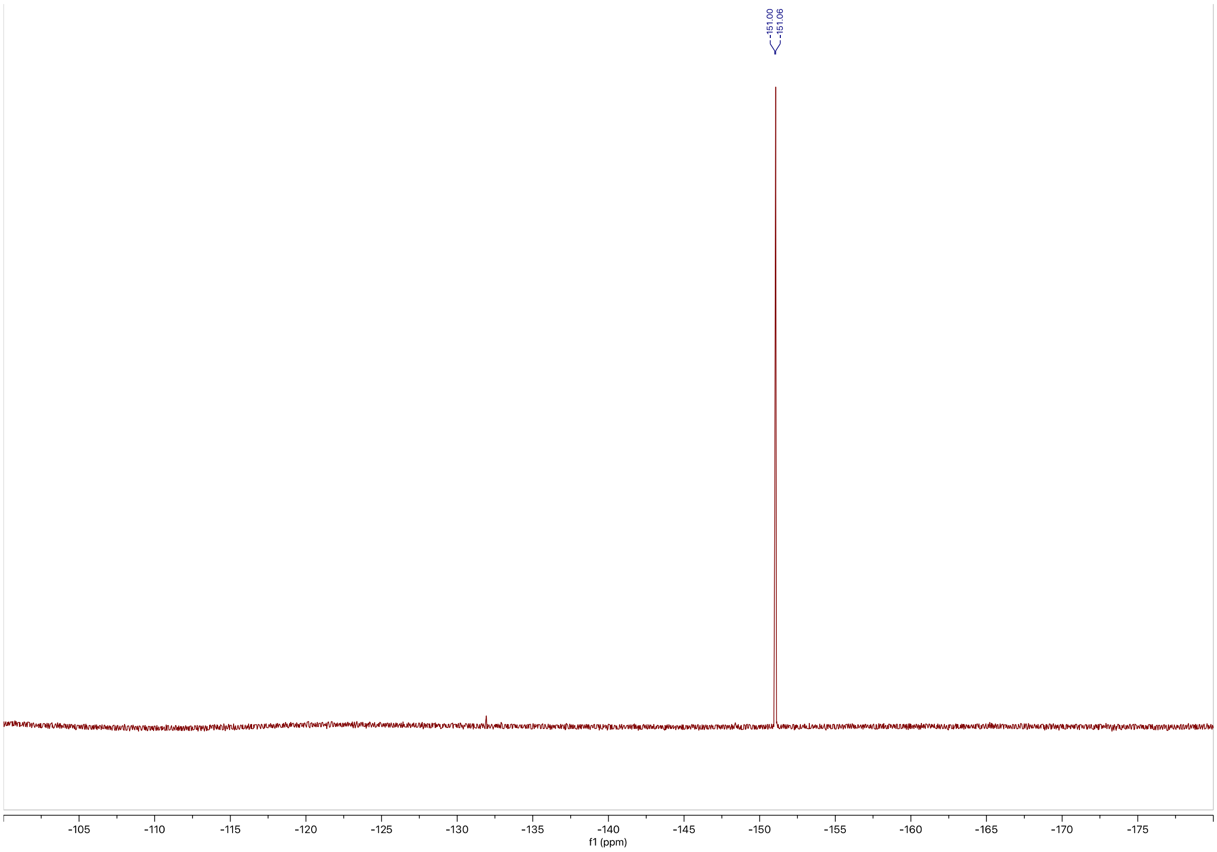

¹H-NMR (499.64 MHz, $CDCl_3$): of **Compound 18** (see Procedure)

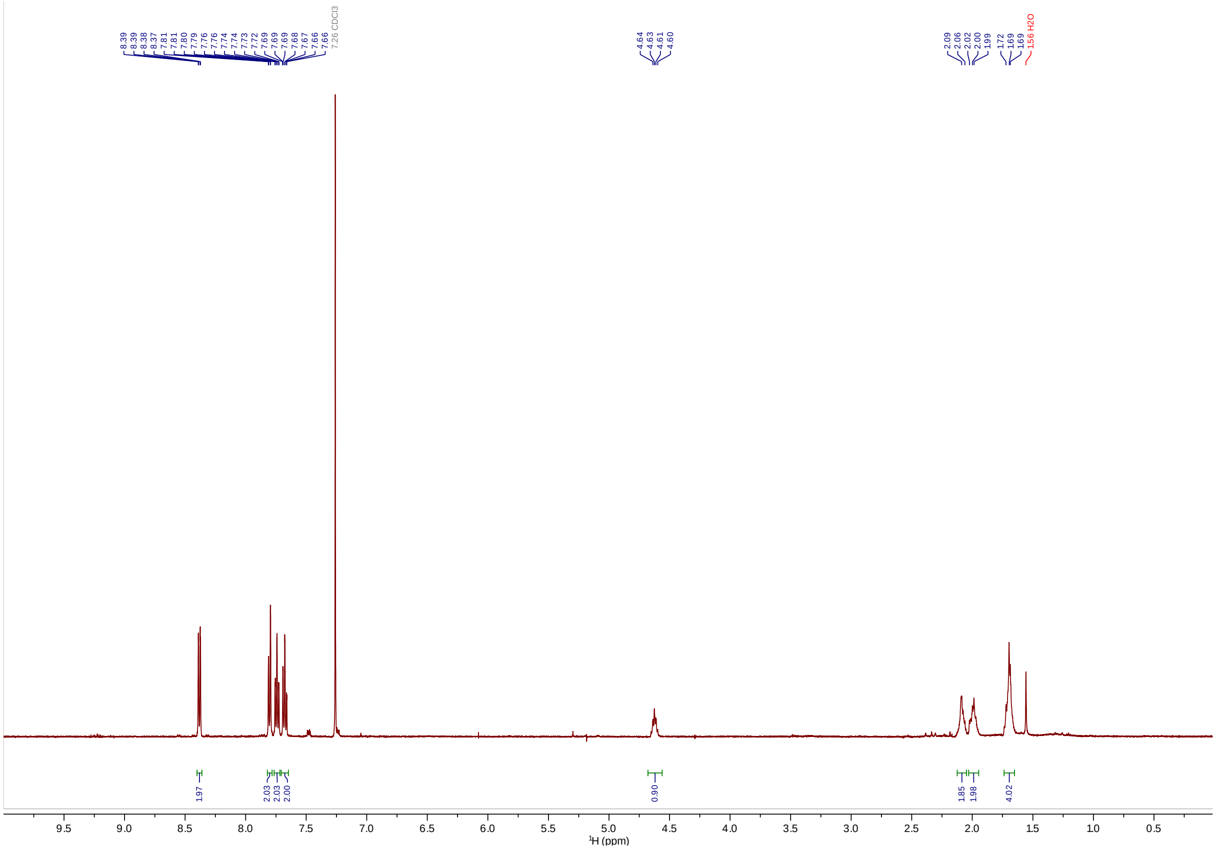

¹³C-NMR (125.65 MHz, $CDCl_3$) of **Compound 18** (see Procedure)

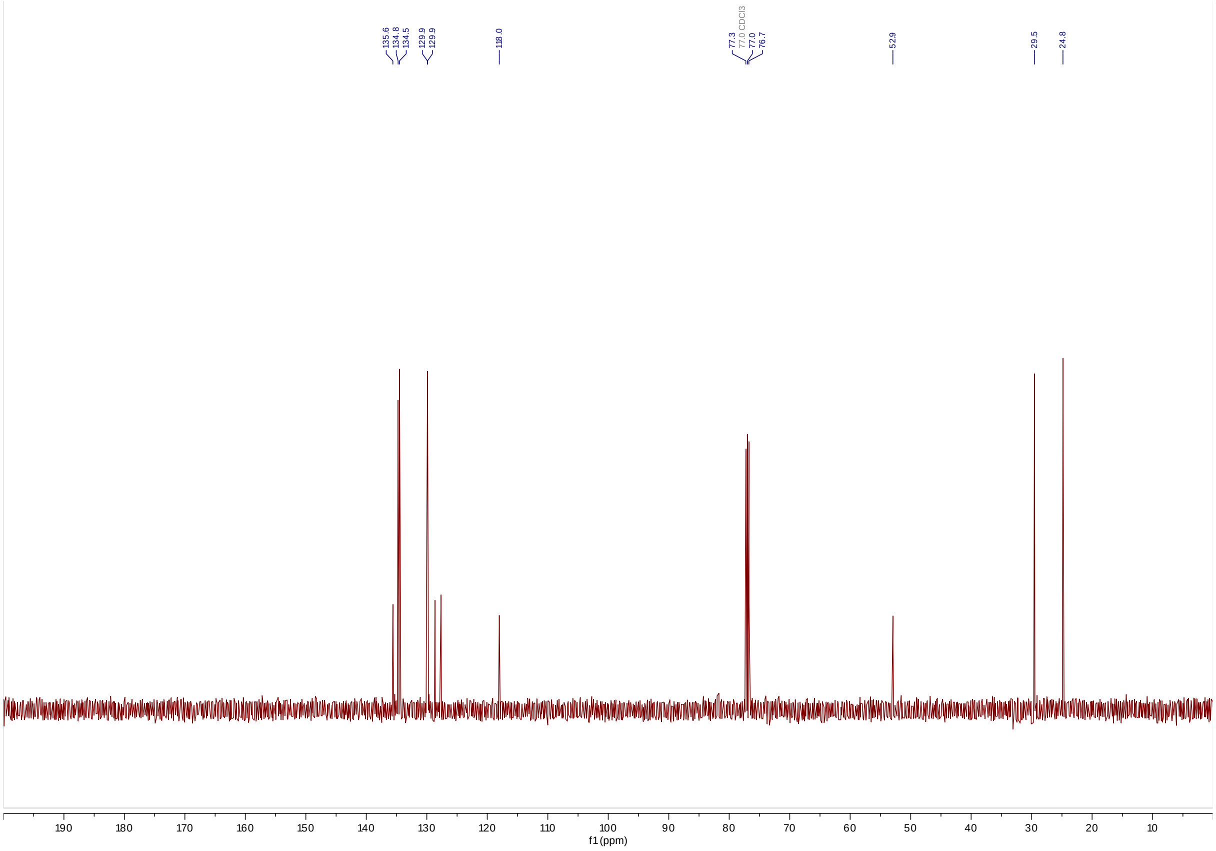

^{19}F-NMR (282.21 MHz, $CDCl_3$) of **Compound 18** (see Procedure)

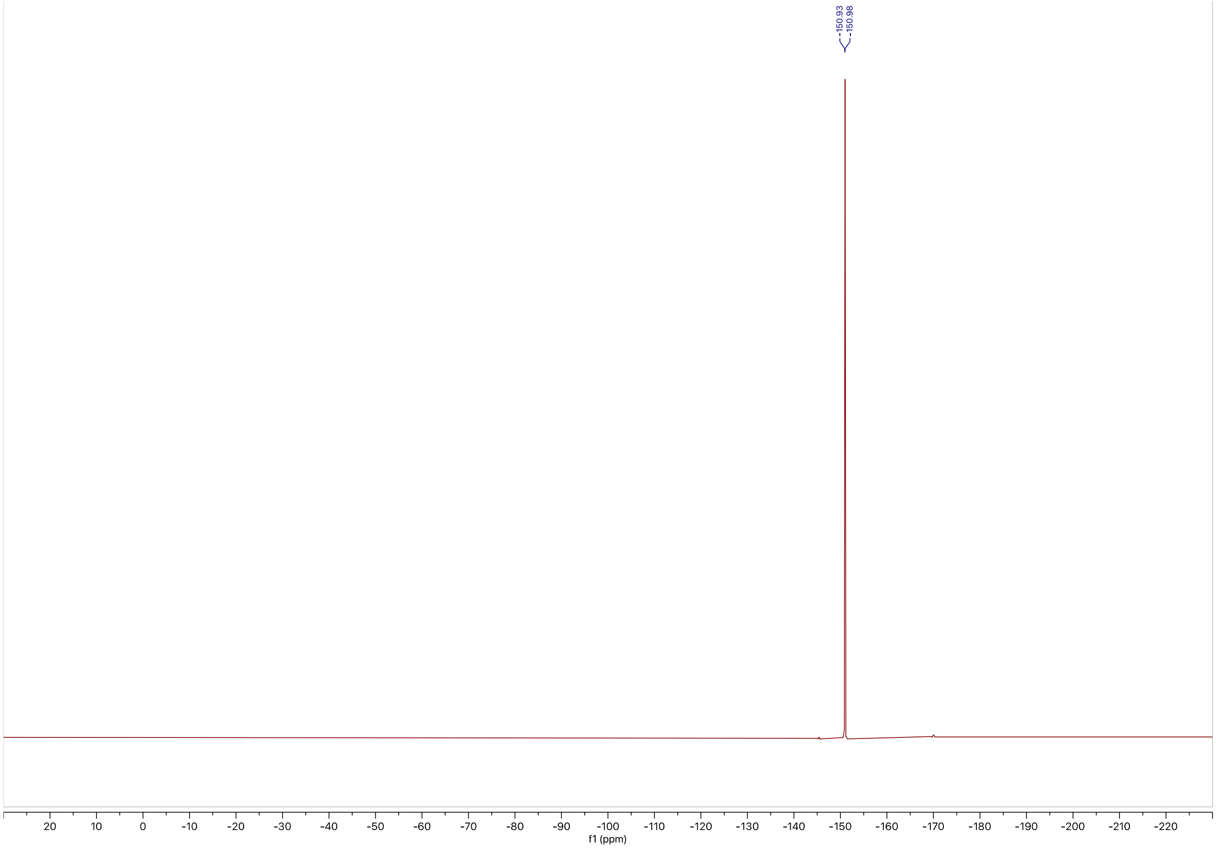

^{1}H-NMR (499.64 MHz, $CDCl_3$): of **Compound 19** (see Procedure)

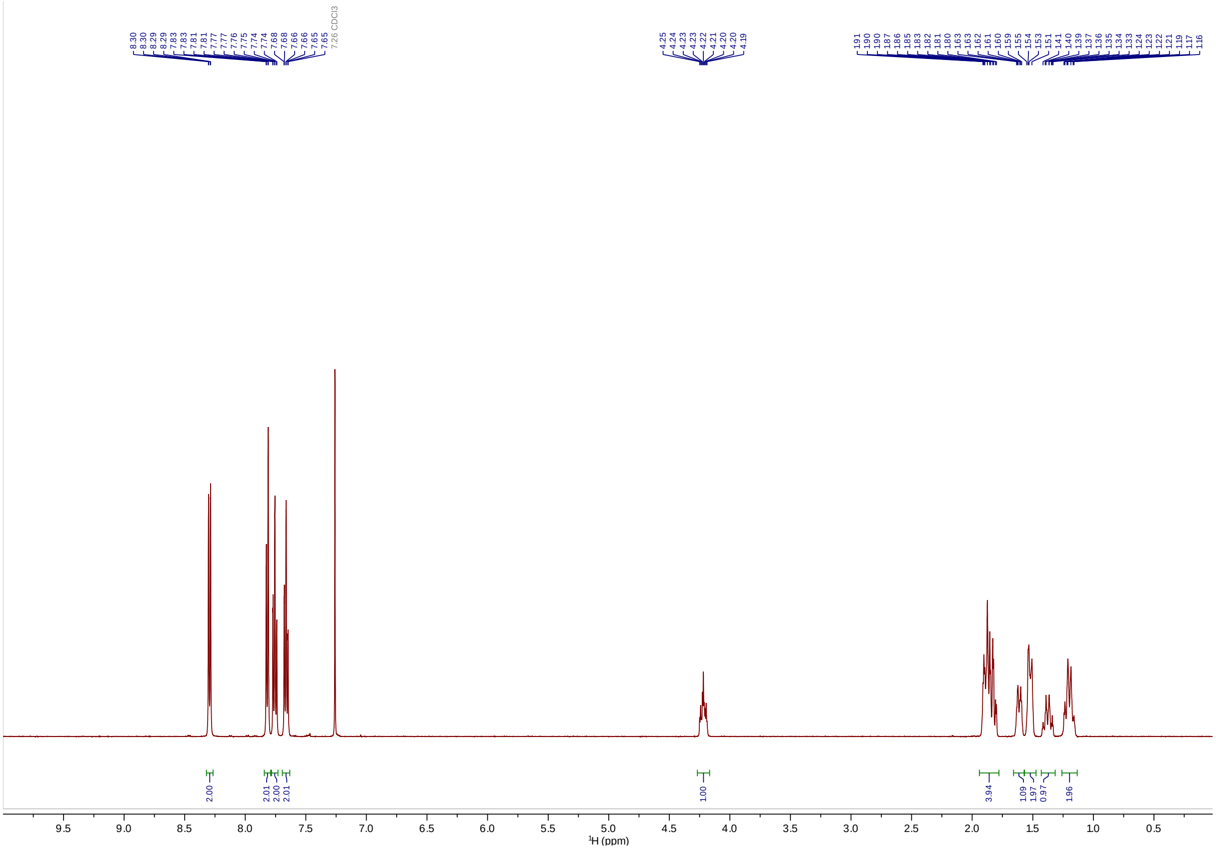

^{13}C-NMR (125.65 MHz, $CDCl_3$) of **Compound 19** (see Procedure)

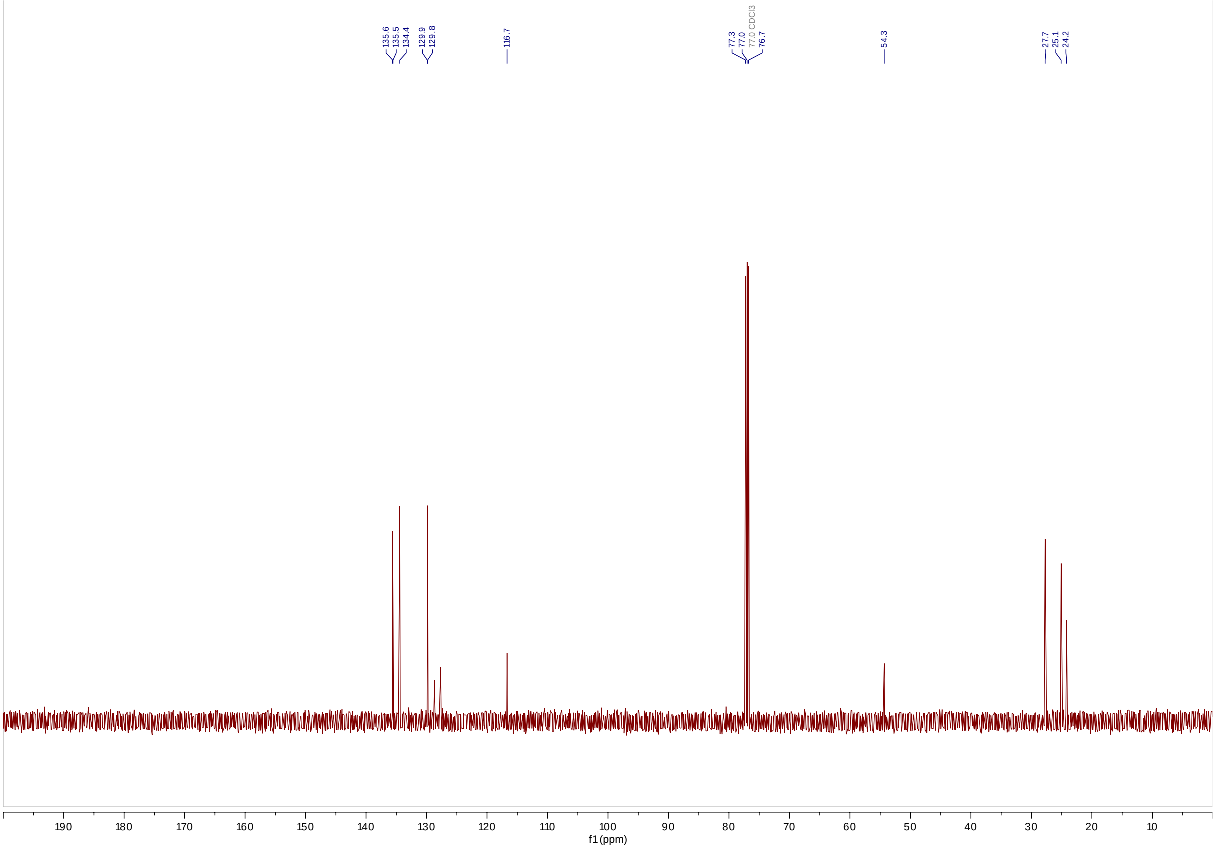

^{19}F-NMR (282.21 MHz, $CDCl_3$) of **Compound 19** (see Procedure)

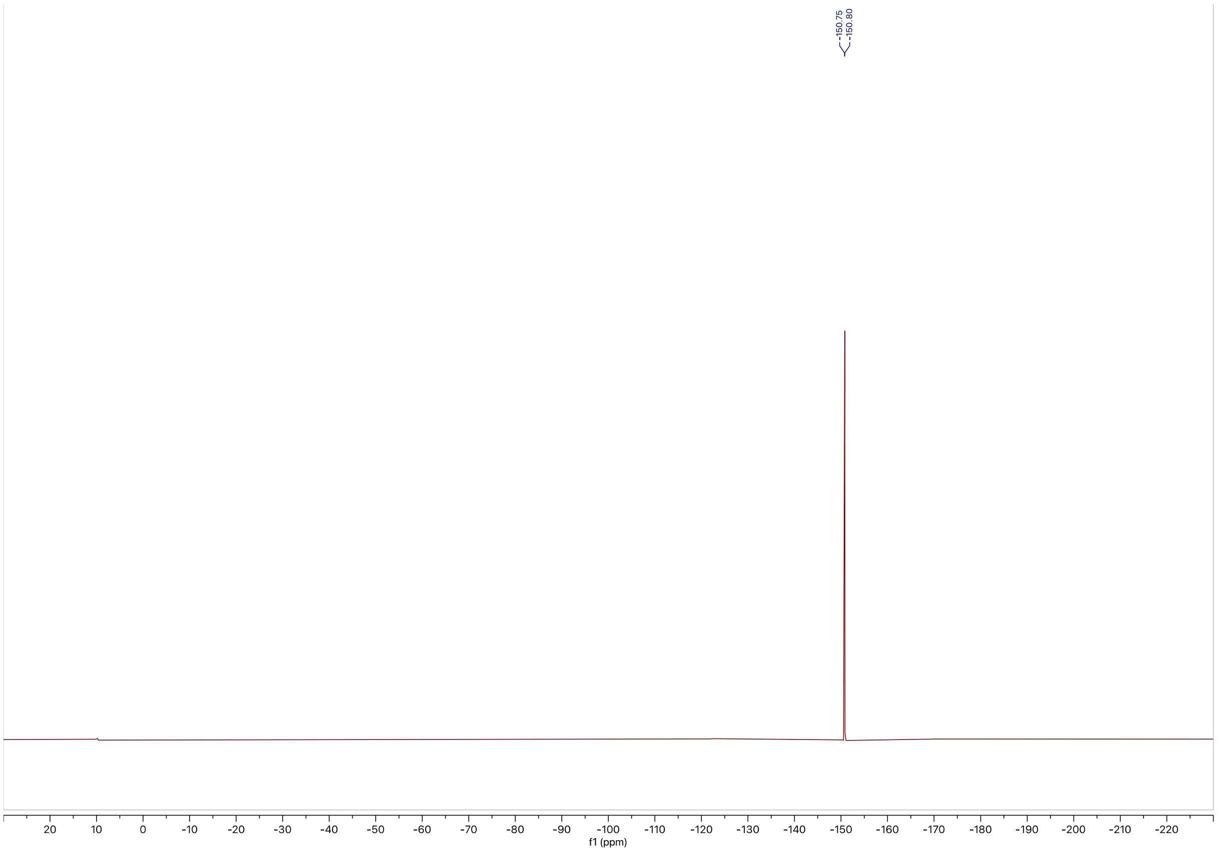

^{1}H-NMR (499.64 MHz, $CDCl_3$): of **Compound 20** (see Procedure)

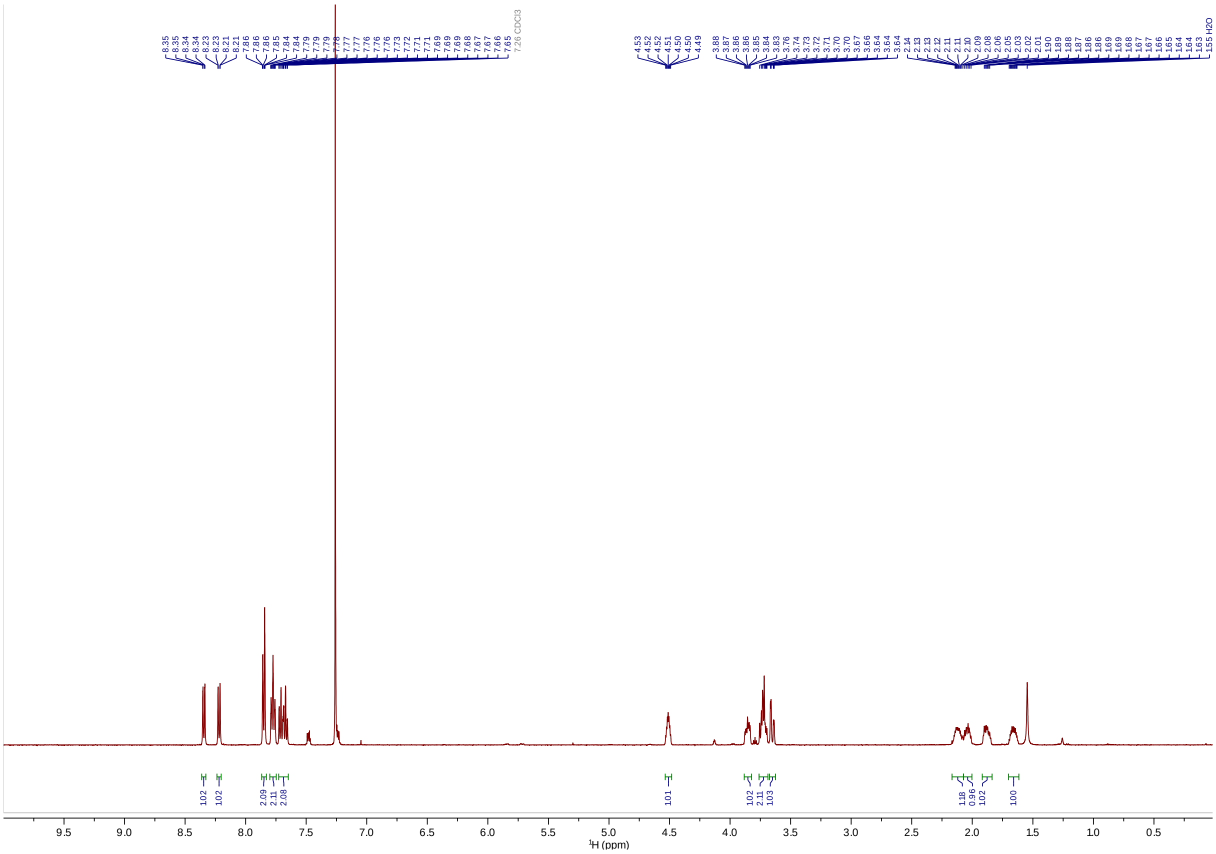

^{13}C-NMR (125.65 MHz, $CDCl_3$) of **Compound 20** (see Procedure)

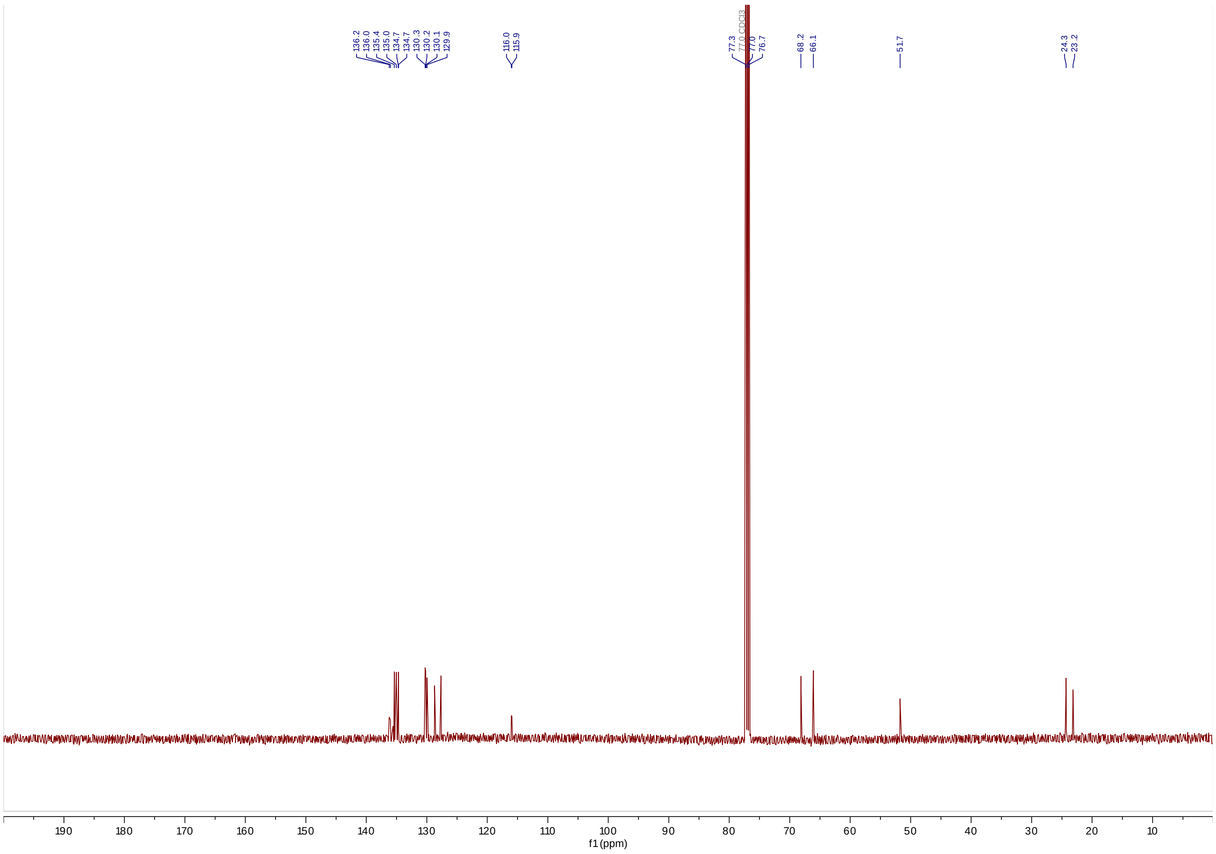

^{19}F-NMR (282.21 MHz, $CDCl_3$) of **Compound 20** (see Procedure)

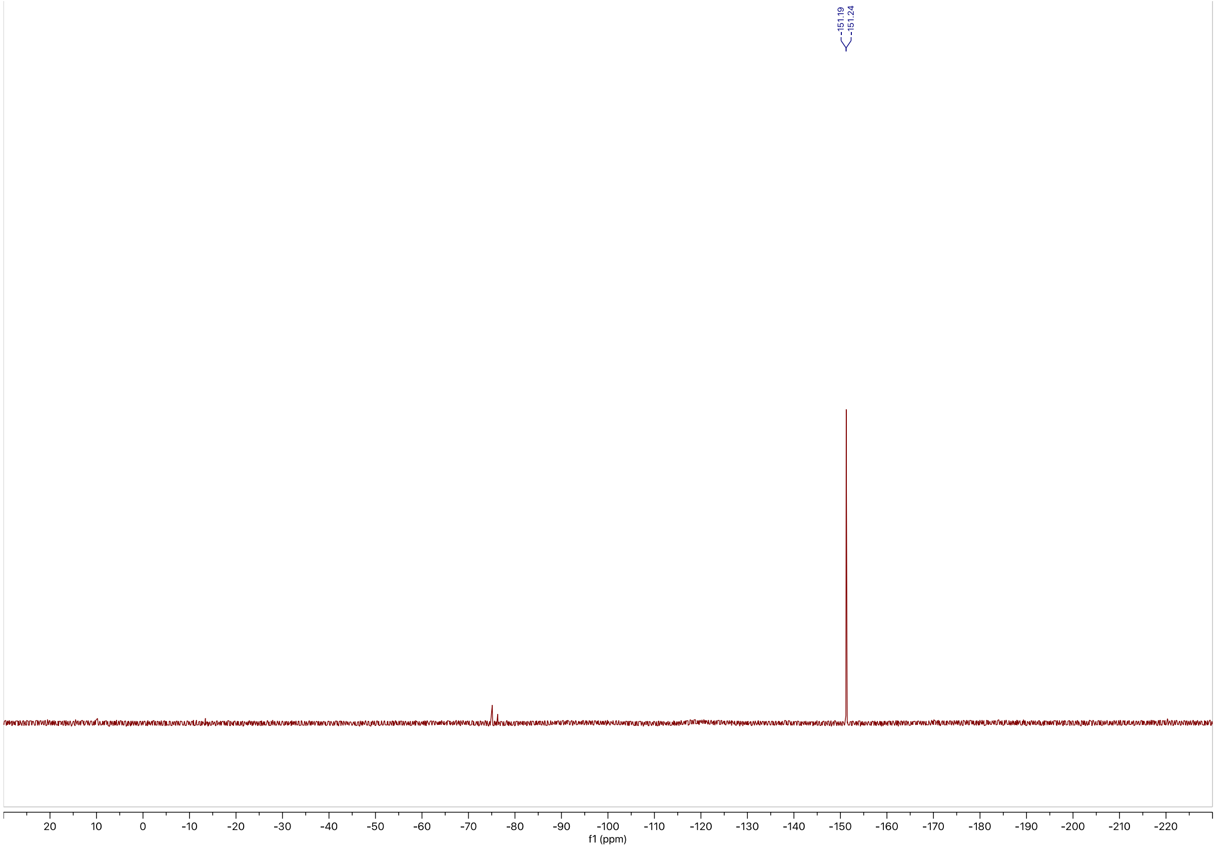

¹H-NMR (499.64 MHz, CDCl3): of **Compound 22** (see Procedure)

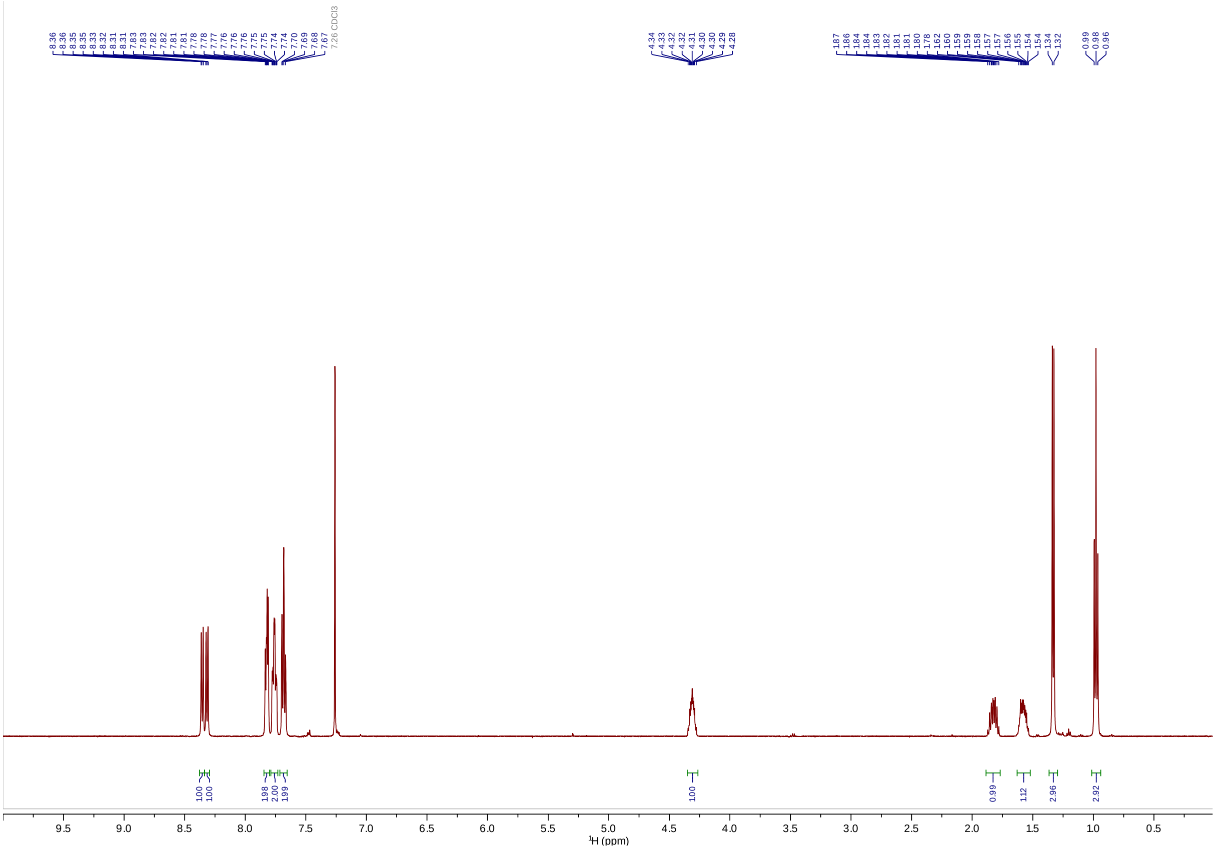

¹³C-NMR (125.65 MHz, CDCl3) of **Compound 22** (see Procedure)

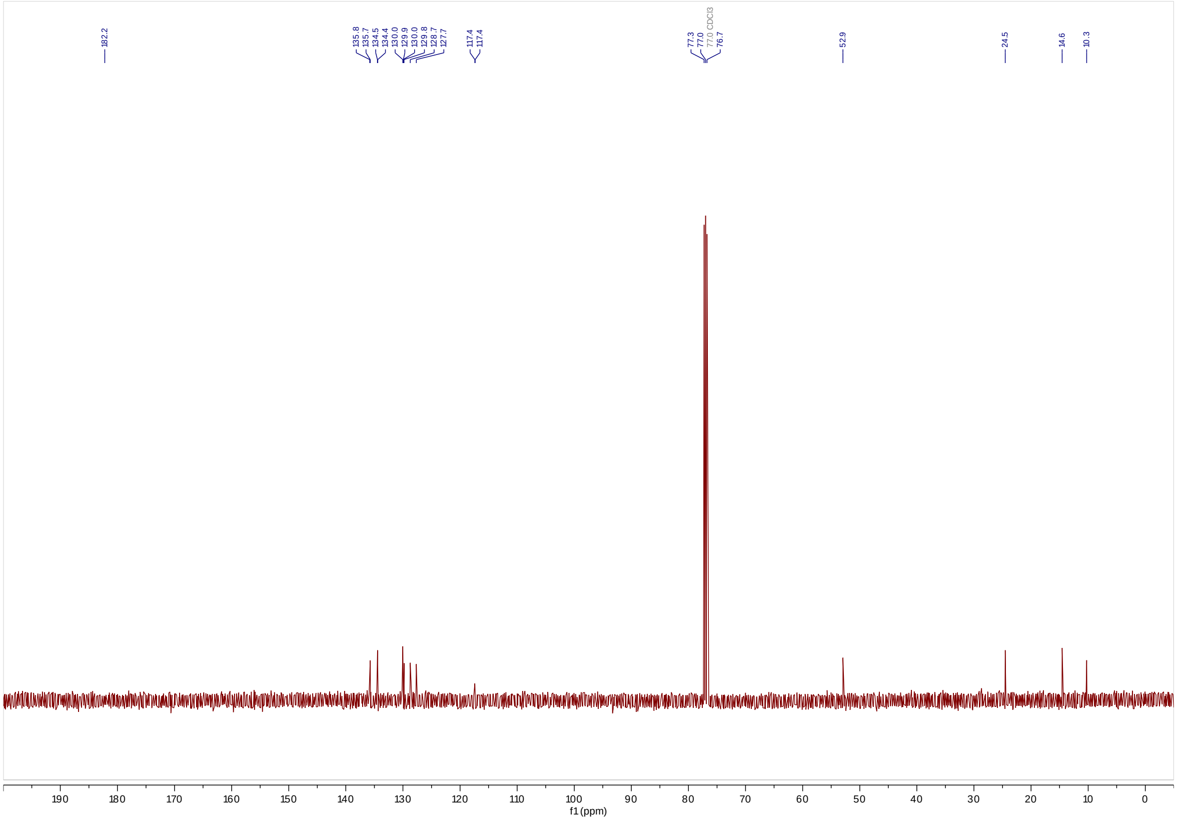

^{19}F-NMR (282.21 MHz, $CDCl_3$) of **Compound 22** (see Procedure)

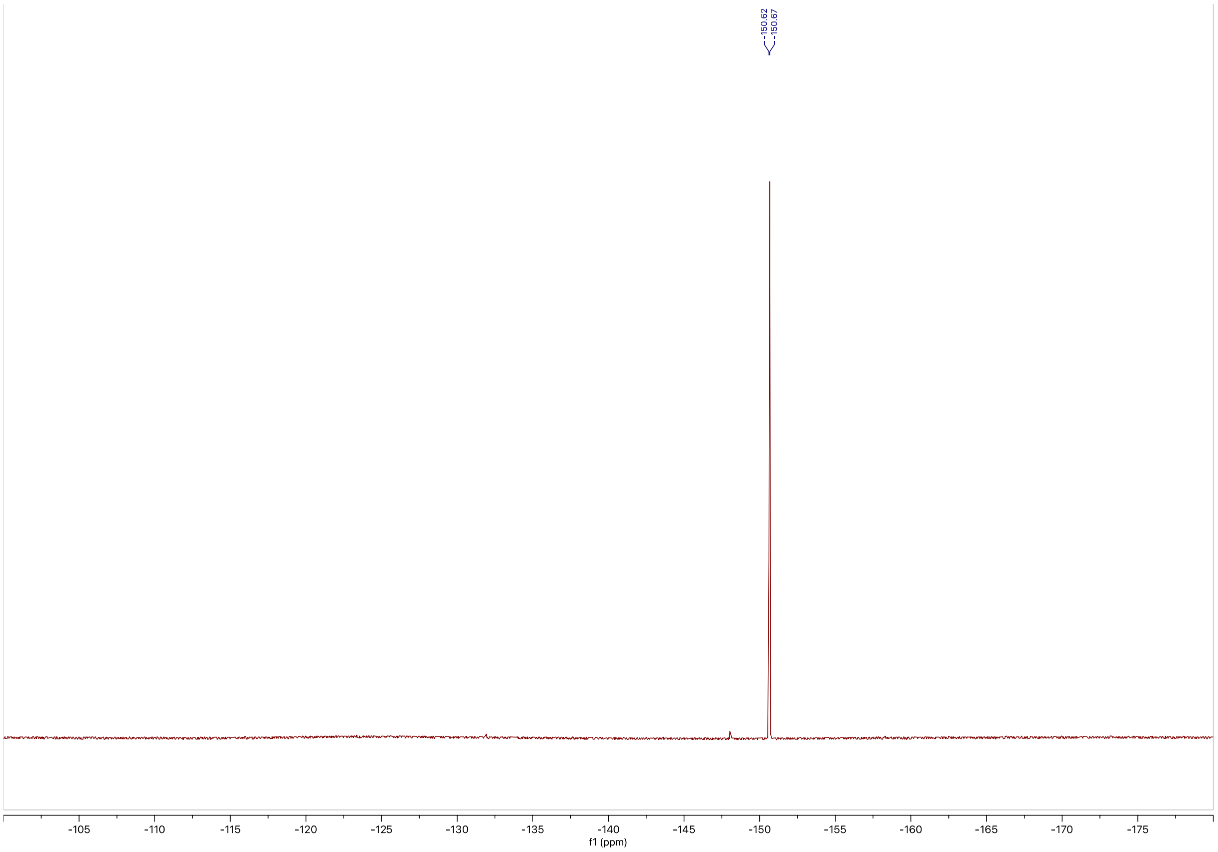

^{1}H-NMR (499.64 MHz, $CDCl_3$): of **Compound 23** (see Procedure)

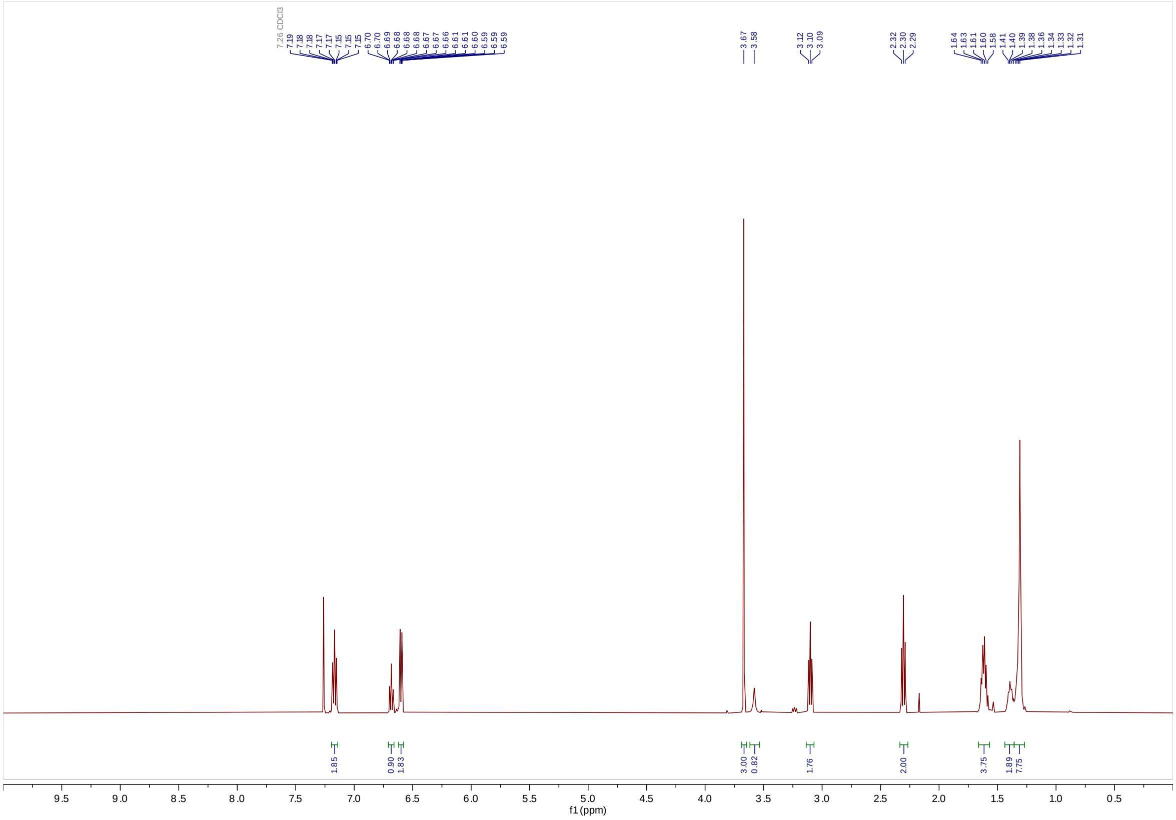

^{13}C-NMR (125.65 MHz, $CDCl_3$) of **Compound 23** (see Procedure)

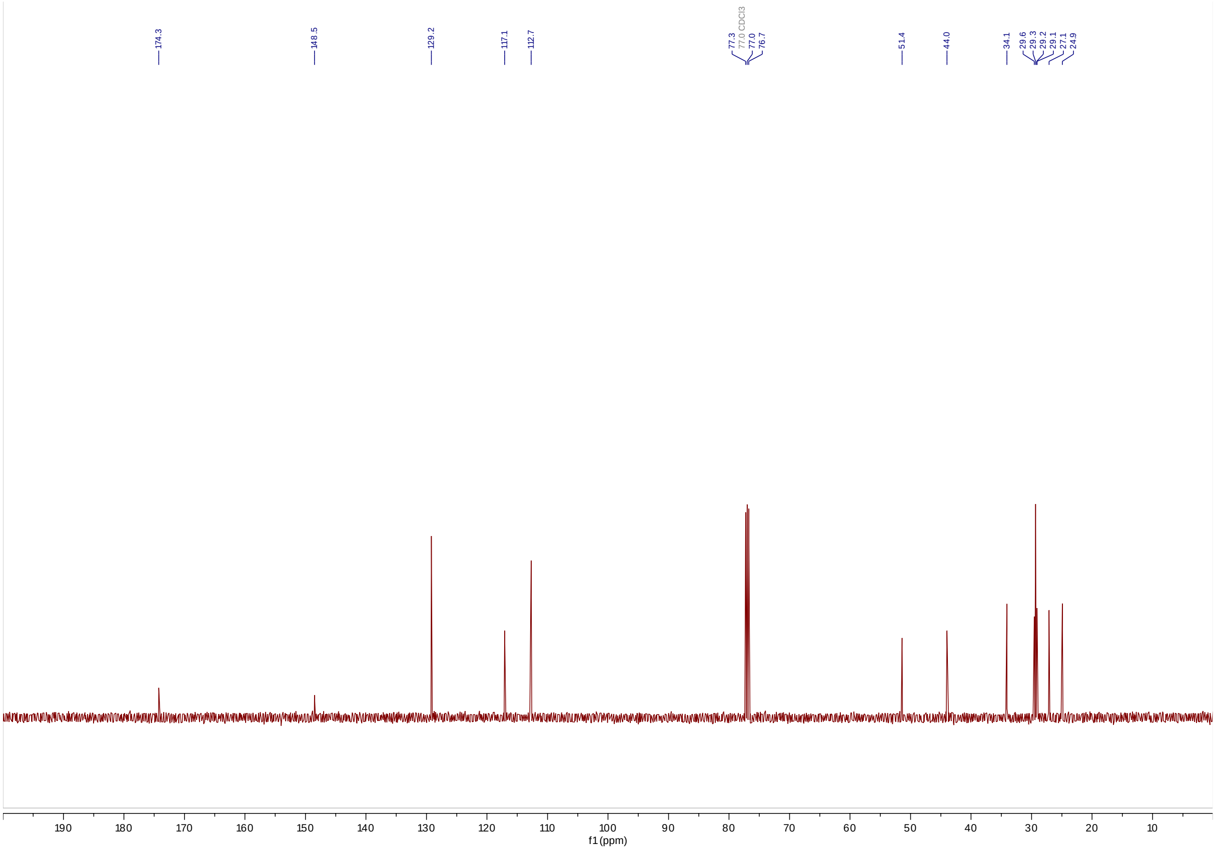

^{1}H-NMR (499.64 MHz, $CDCl_3$): of **Compound 24** (see Procedure)

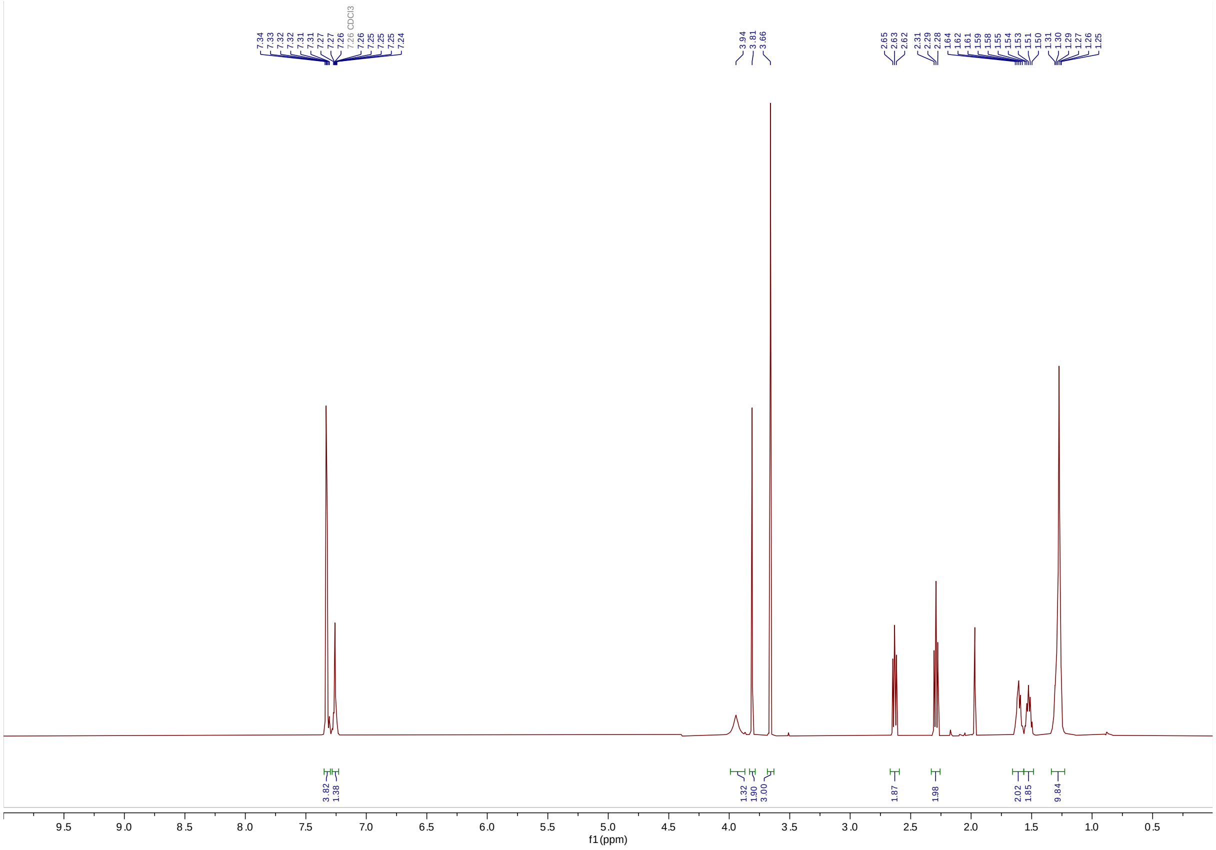

^{13}C-NMR (125.65 MHz, $CDCl_3$) of **Compound 24** (see Procedure)

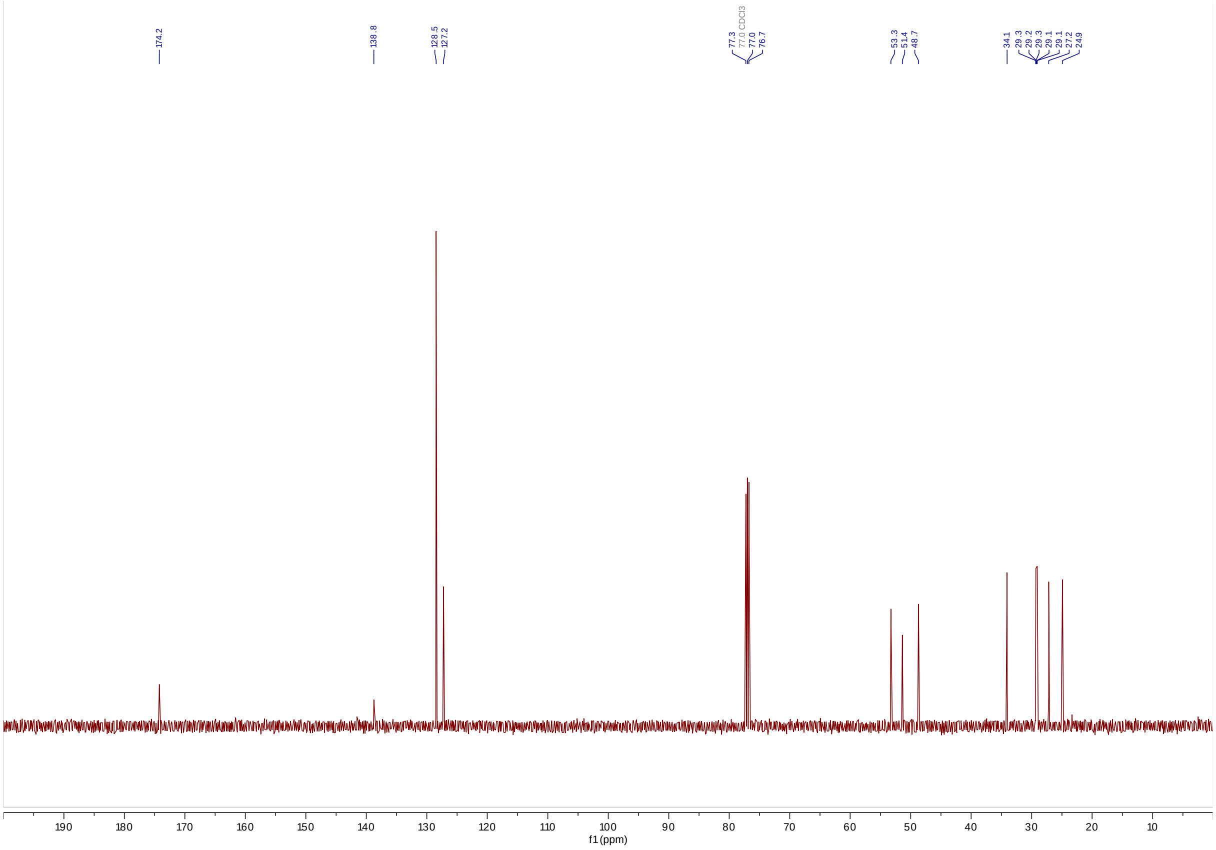

¹H-NMR (499.64 MHz, CDCl₃): of **Compound 25** (see Procedure)

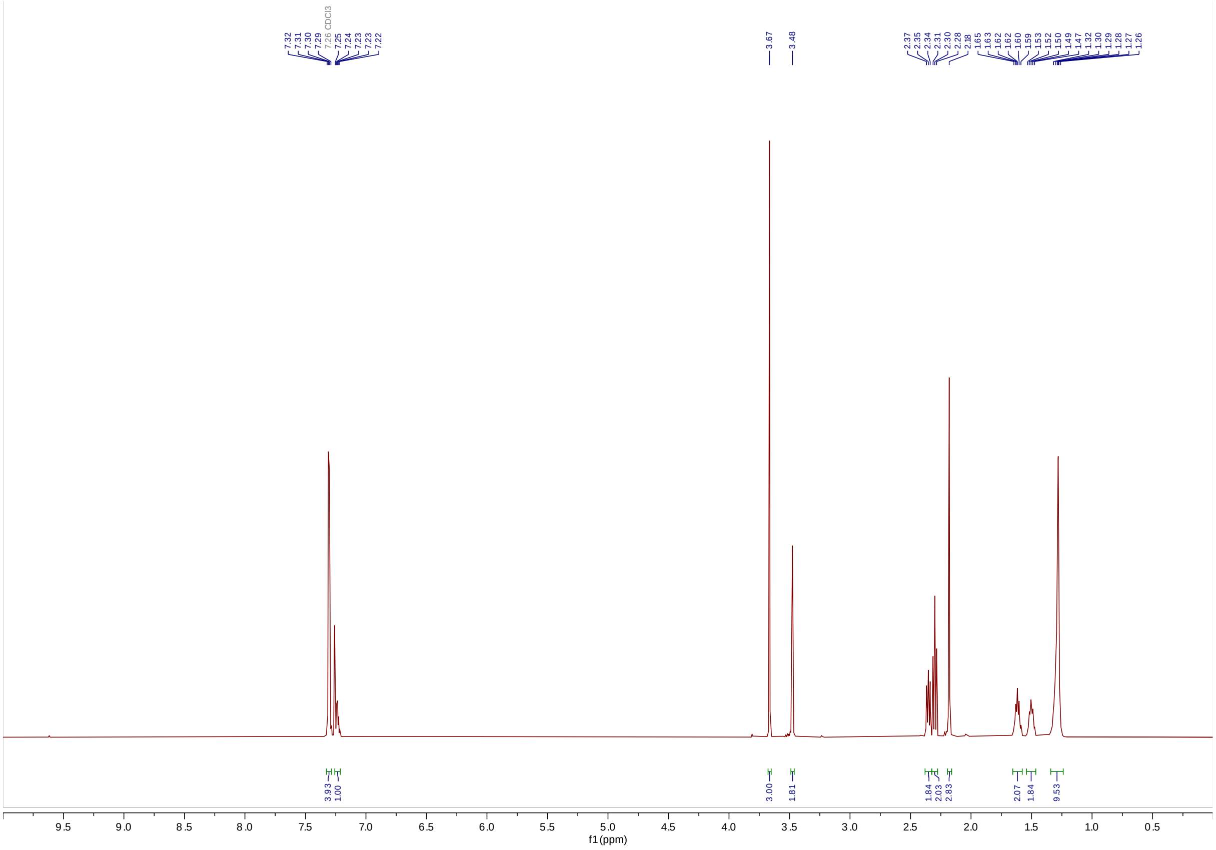

¹³C-NMR (125.65 MHz, CDCl₃) of **Compound 25** (see Procedure)

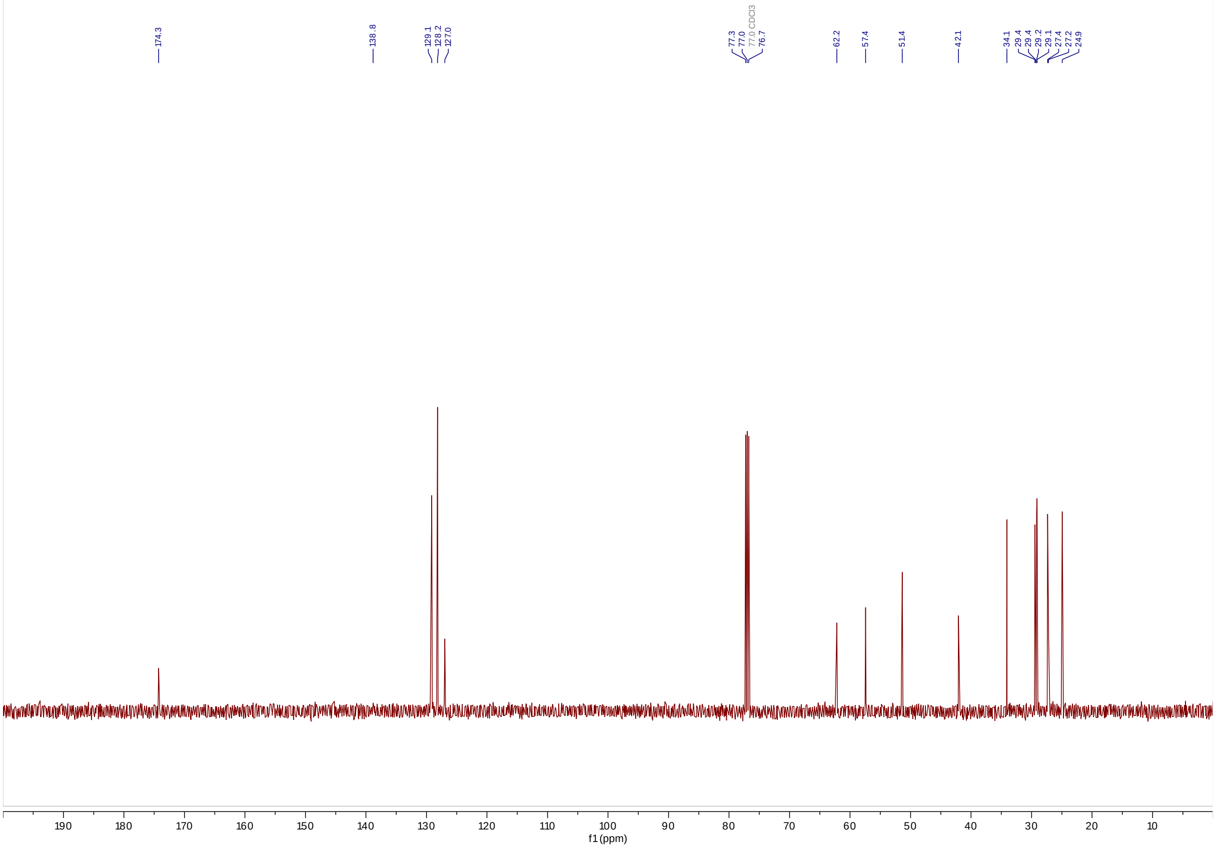

^{1}H-NMR (499.64 MHz, $CDCl_3$): of **Compound 26** (see Procedure)

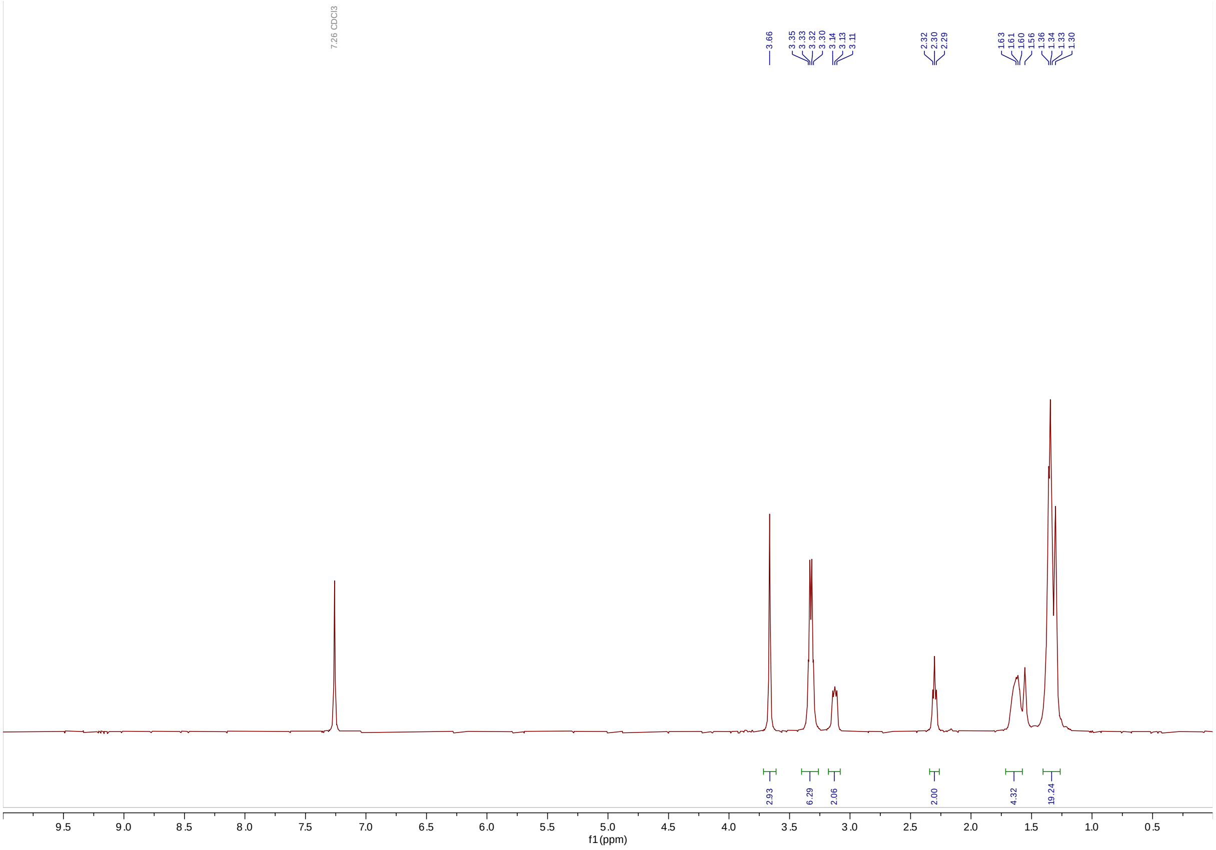

^{13}C-NMR (125.65 MHz, $CDCl_3$) of **Compound 26** (see Procedure)

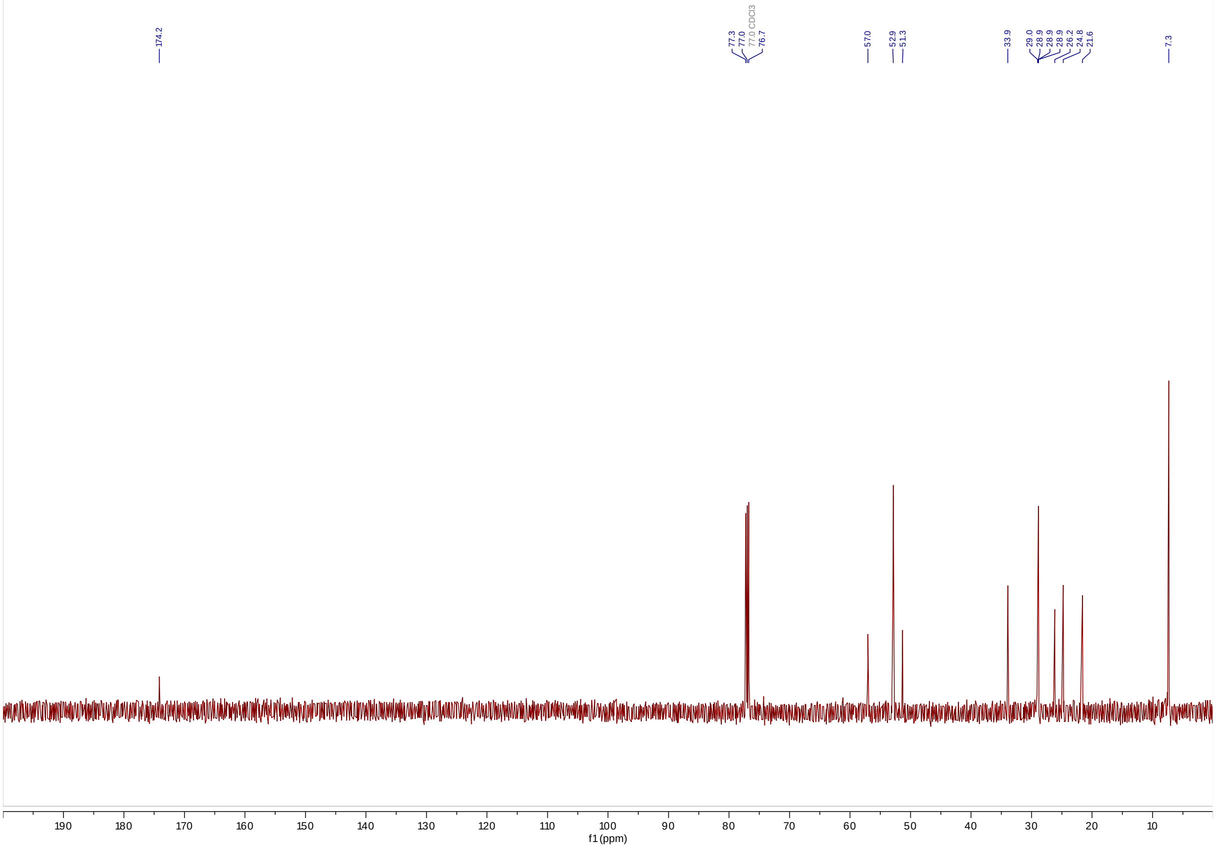

^{19}F-NMR (282.21 MHz, $CDCl_3$) of **Compound 26** (see Procedure)

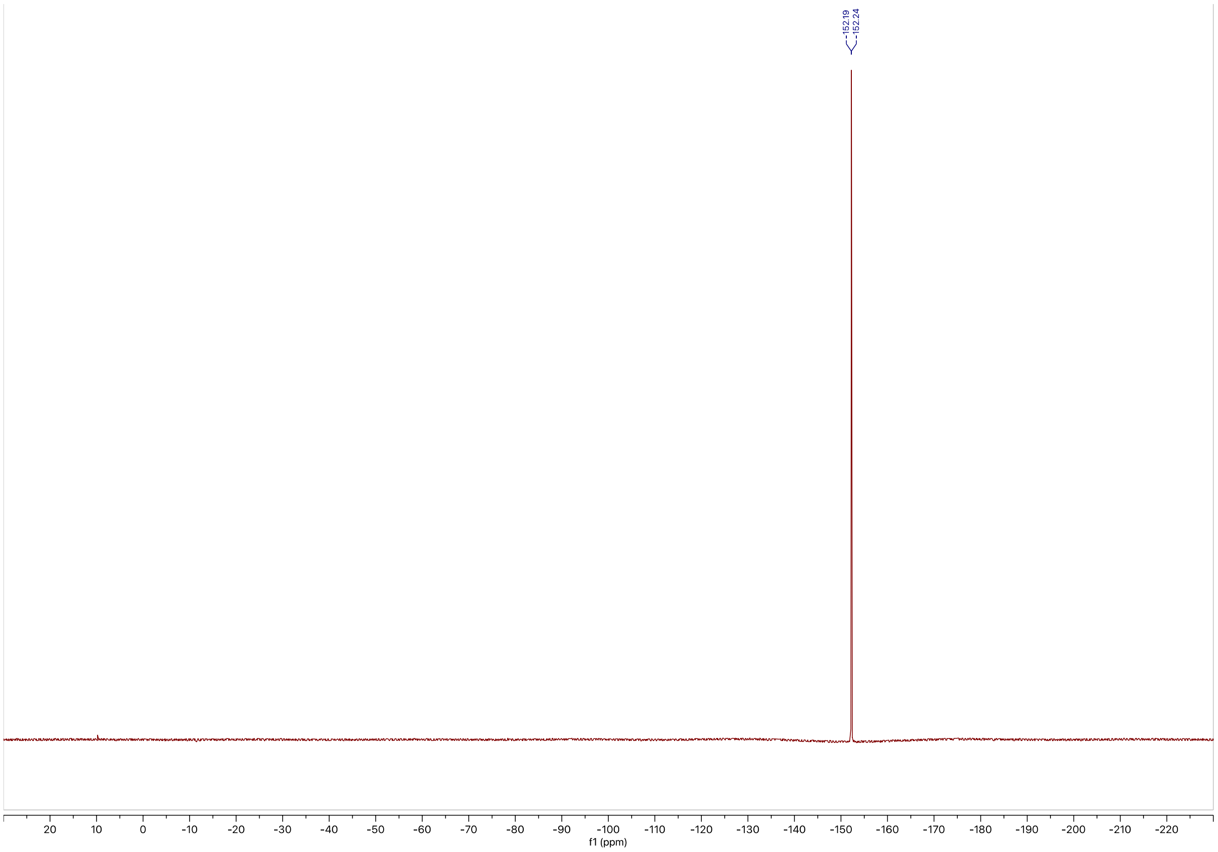

^{1}H-NMR (499.64 MHz, $CDCl_3$): of **Compound 27** (see Procedure)

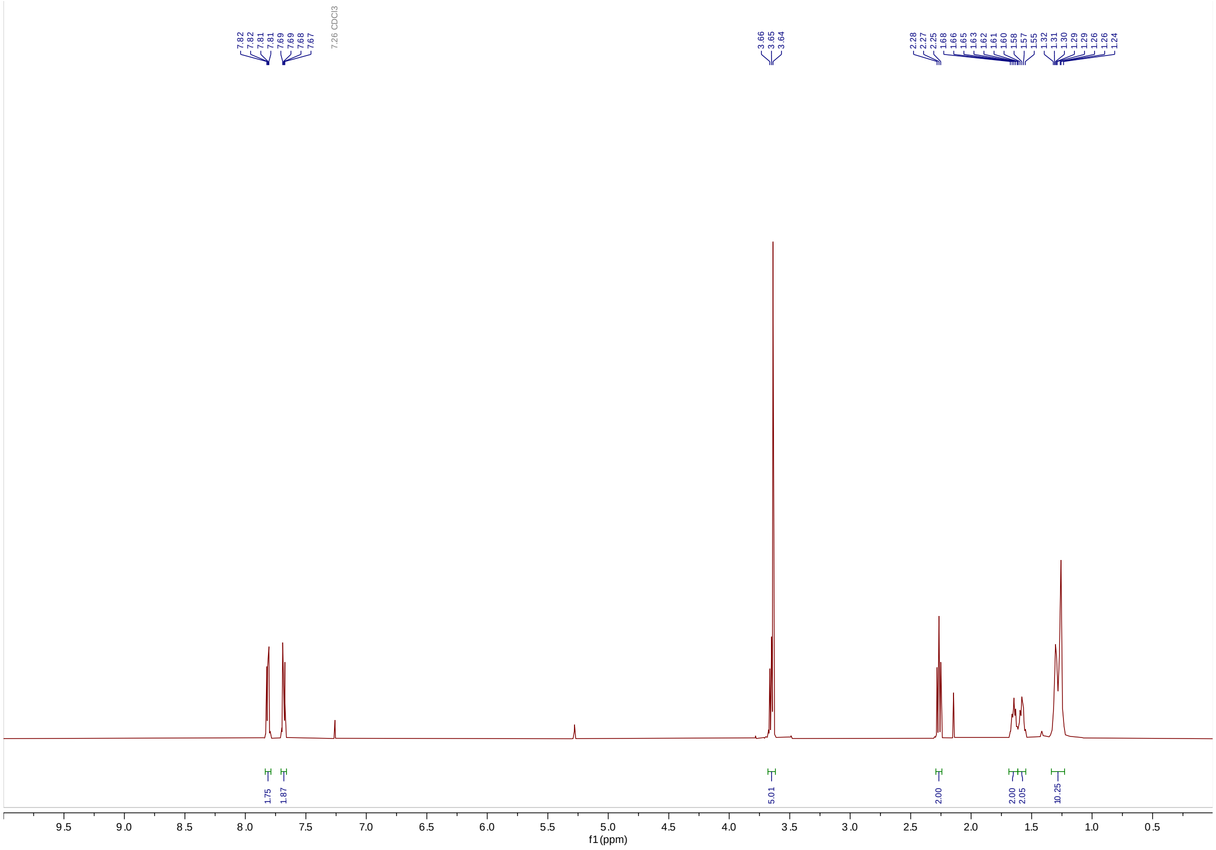

^{13}C-NMR (125.65 MHz, $CDCl_3$) of **Compound 27** (see Procedure)

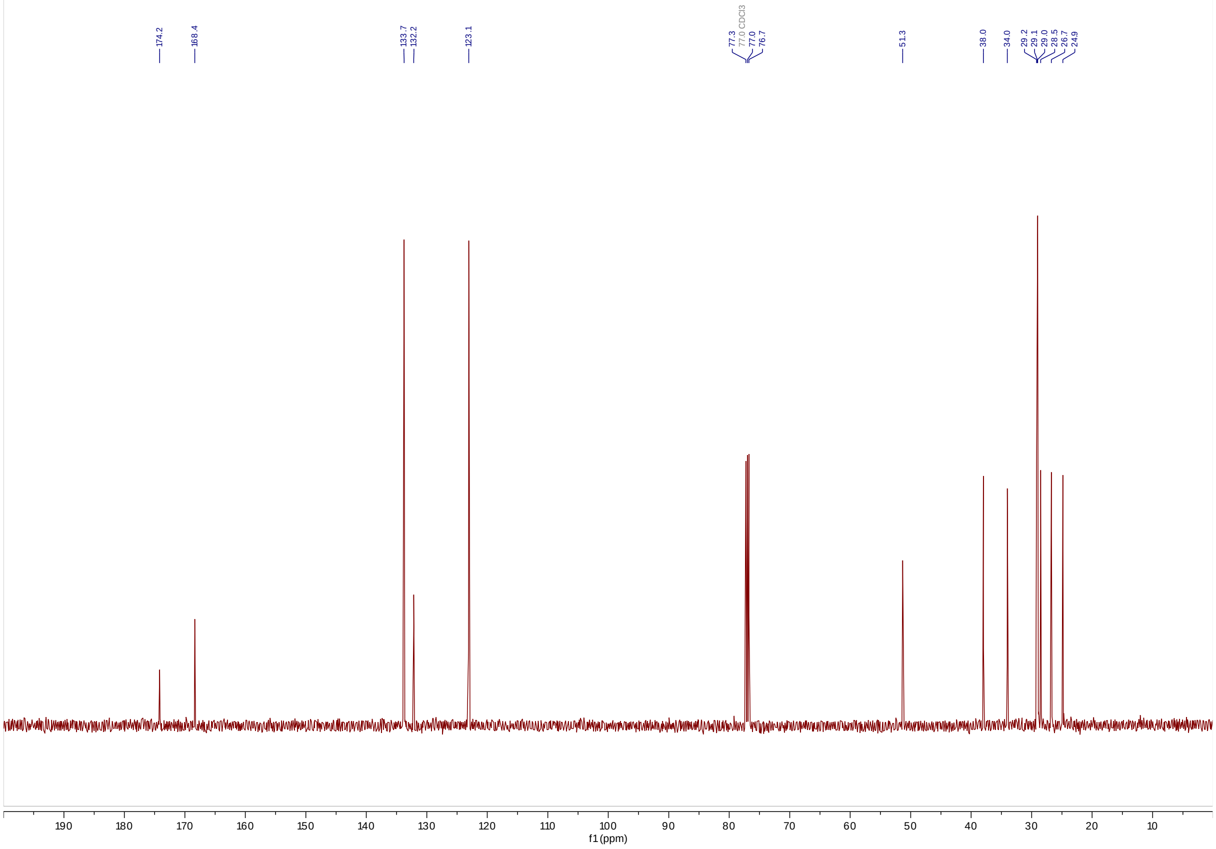

^{1}H-NMR (599.63 MHz, $CDCl_3$): of **Compound 28** (see Procedure)

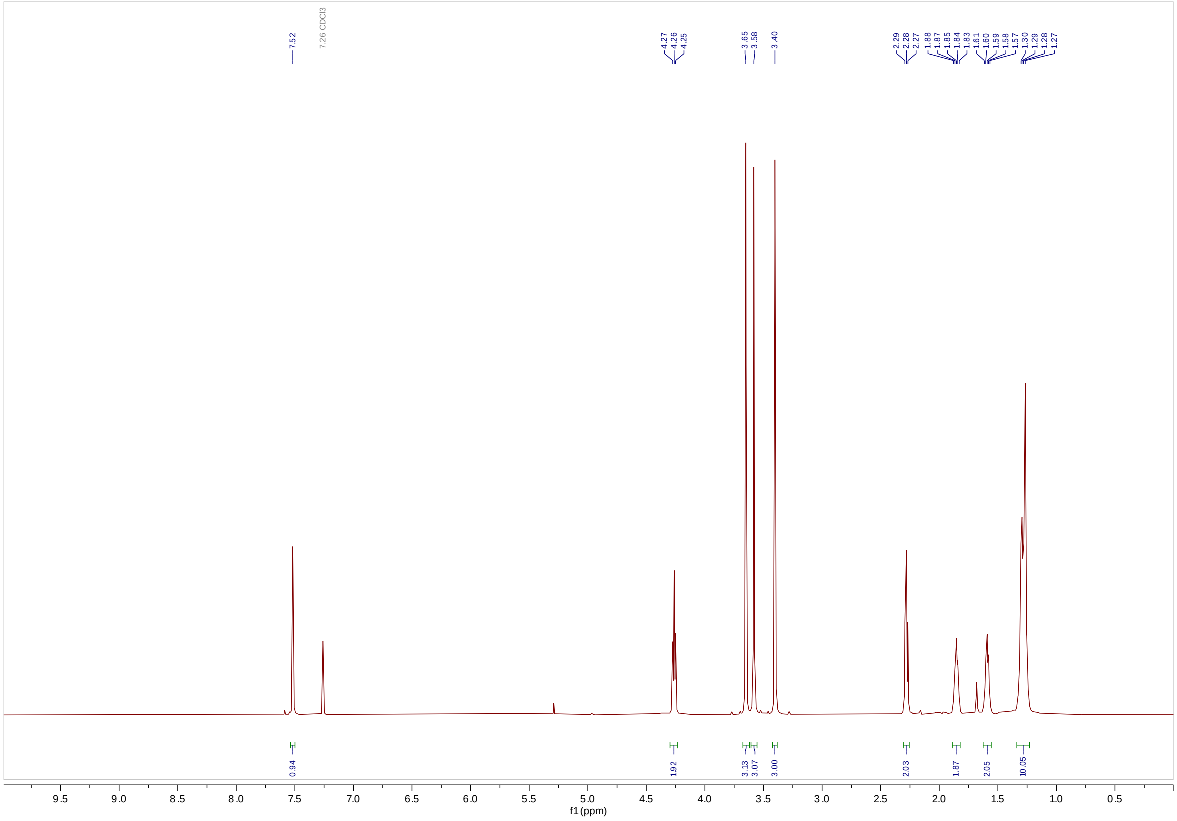

^{13}C-NMR (150.79 MHz, $CDCl_3$) of **Compound 28** (see Procedure)

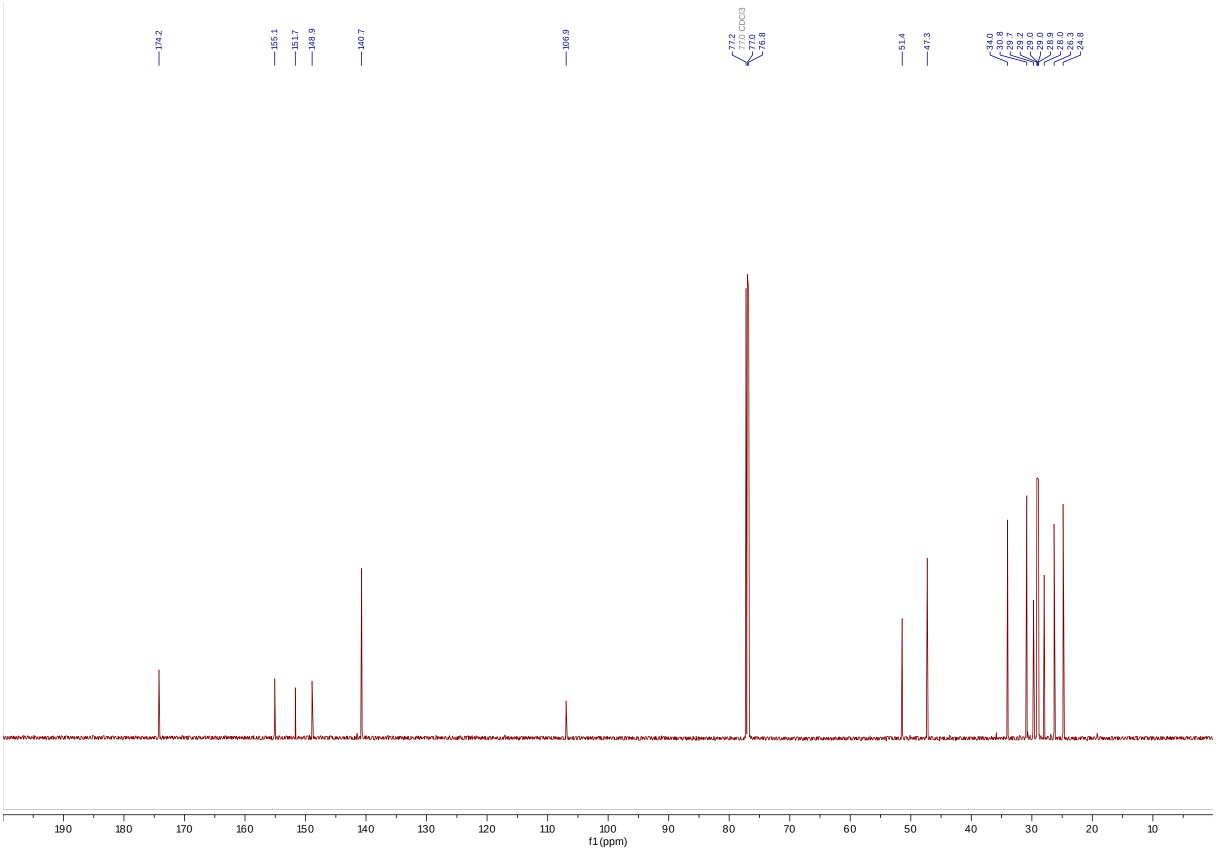

^{1}H-NMR (499.64 MHz, $CDCl_3$): of **Compound 29** (see Procedure)

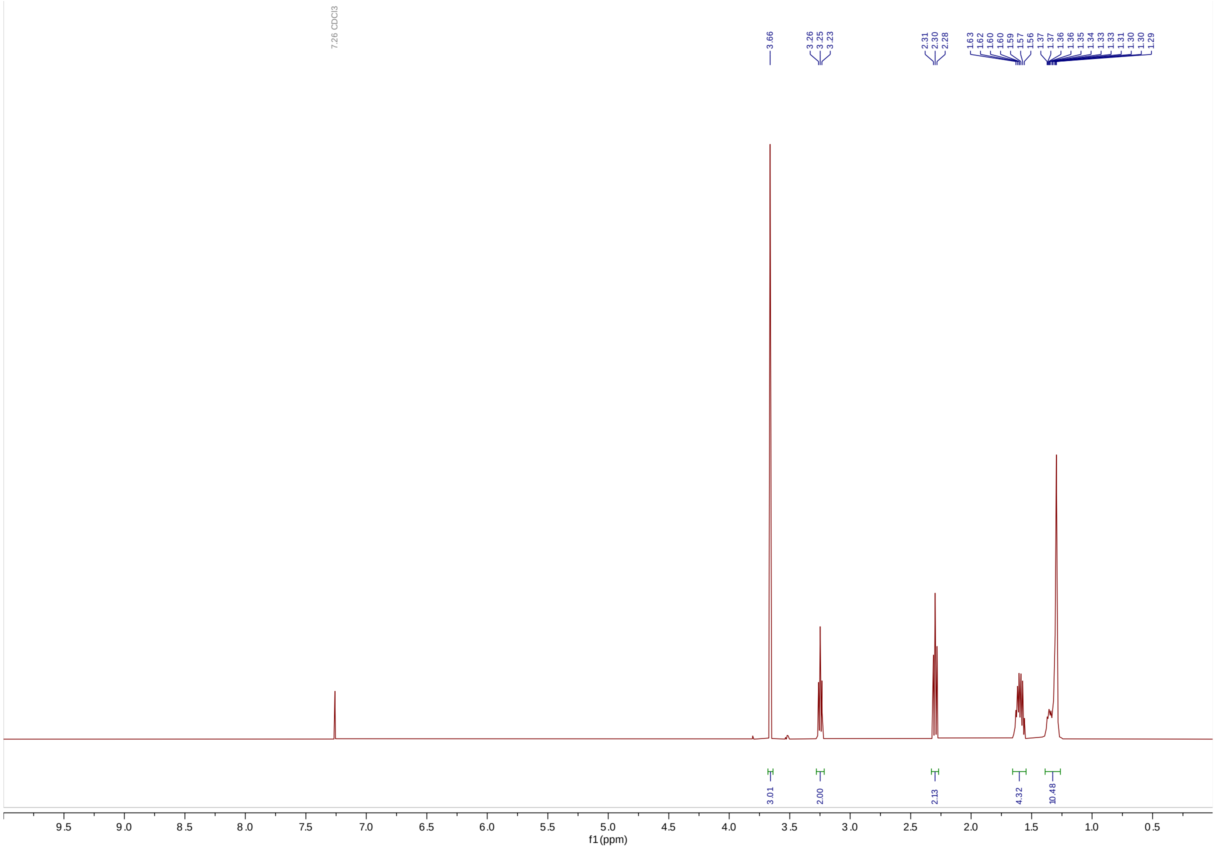

^{13}C-NMR (125.65 MHz, $CDCl_3$) of **Compound 29** (see Procedure)

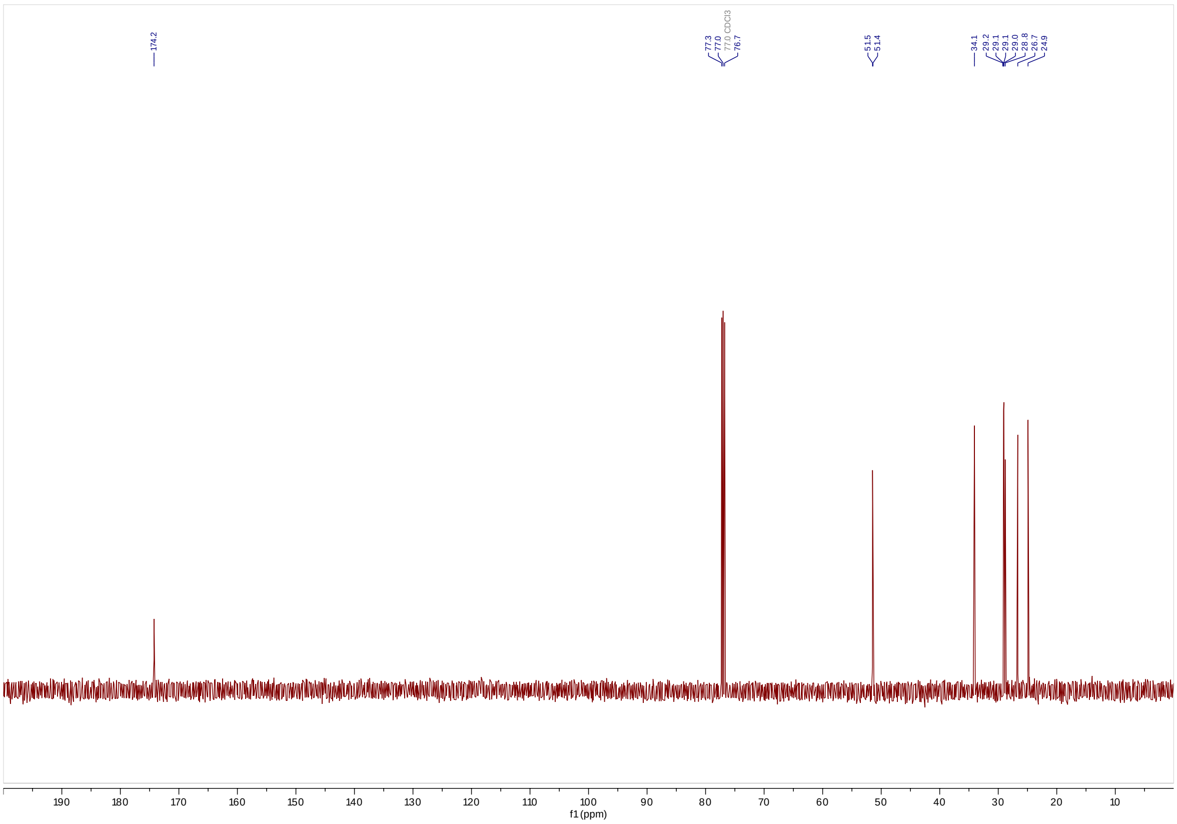

^{1}H-NMR (499.64 MHz, $CDCl_3$): of **Compound 30** (see Procedure)

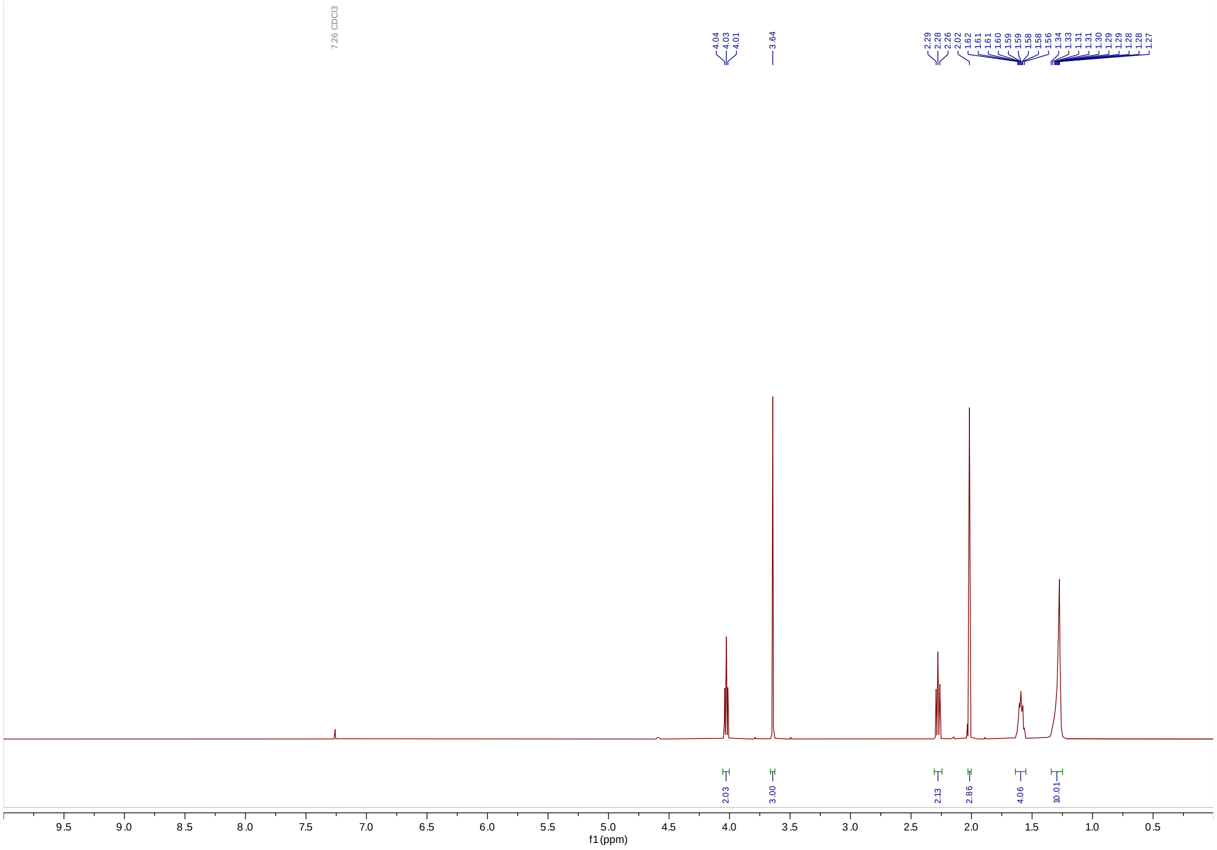

^{13}C-NMR (125.65 MHz, $CDCl_3$) of **Compound 30** (see Procedure)

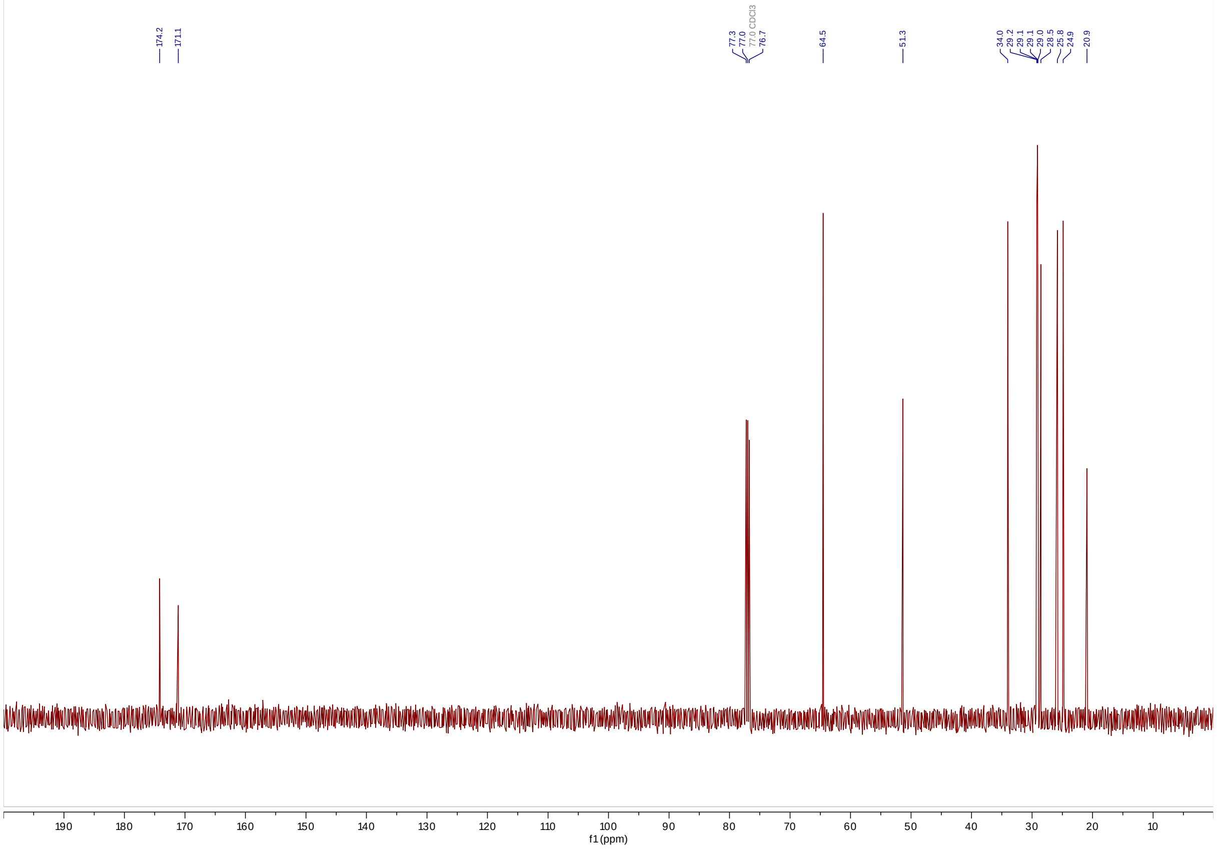

^{1}H-NMR (499.64 MHz, $CDCl_3$): of **Compound 31** (see Procedure)

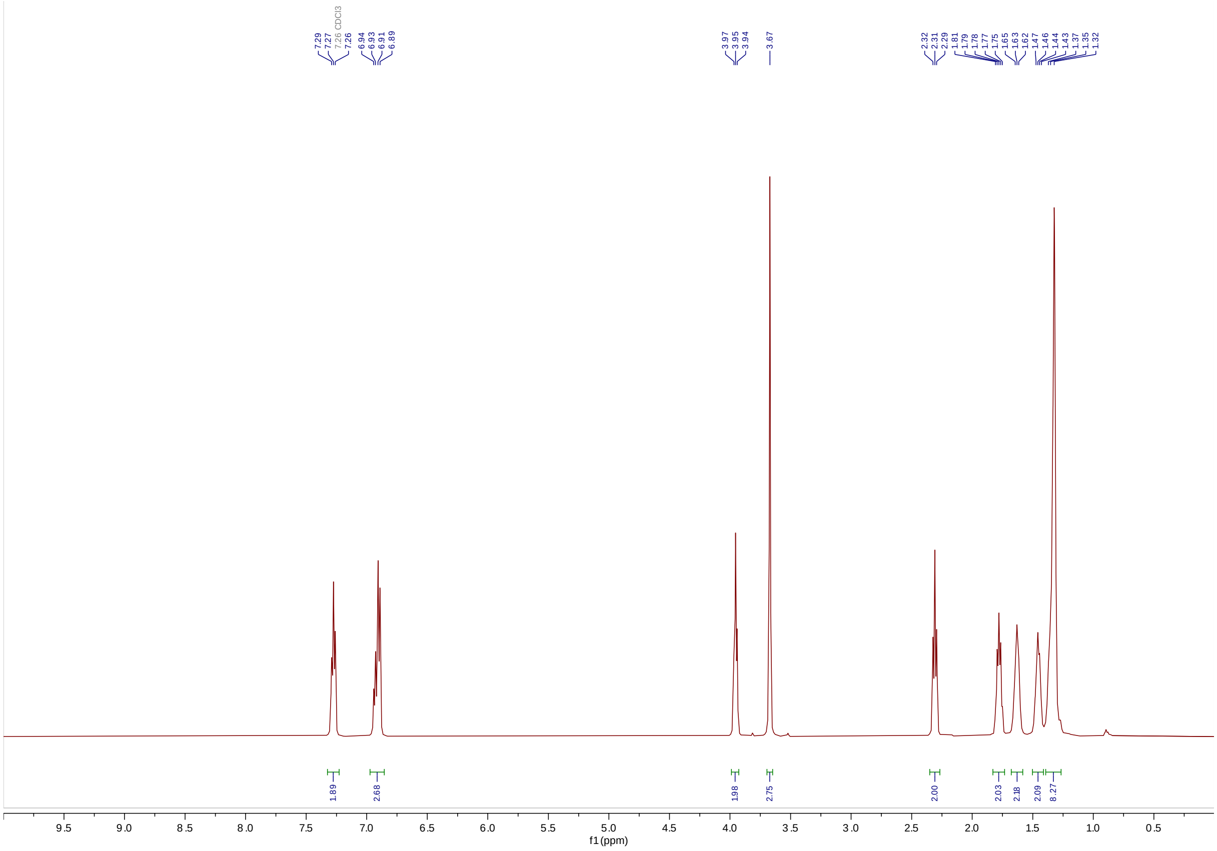

^{13}C-NMR (125.65 MHz, $CDCl_3$) of **Compound 31** (see Procedure)

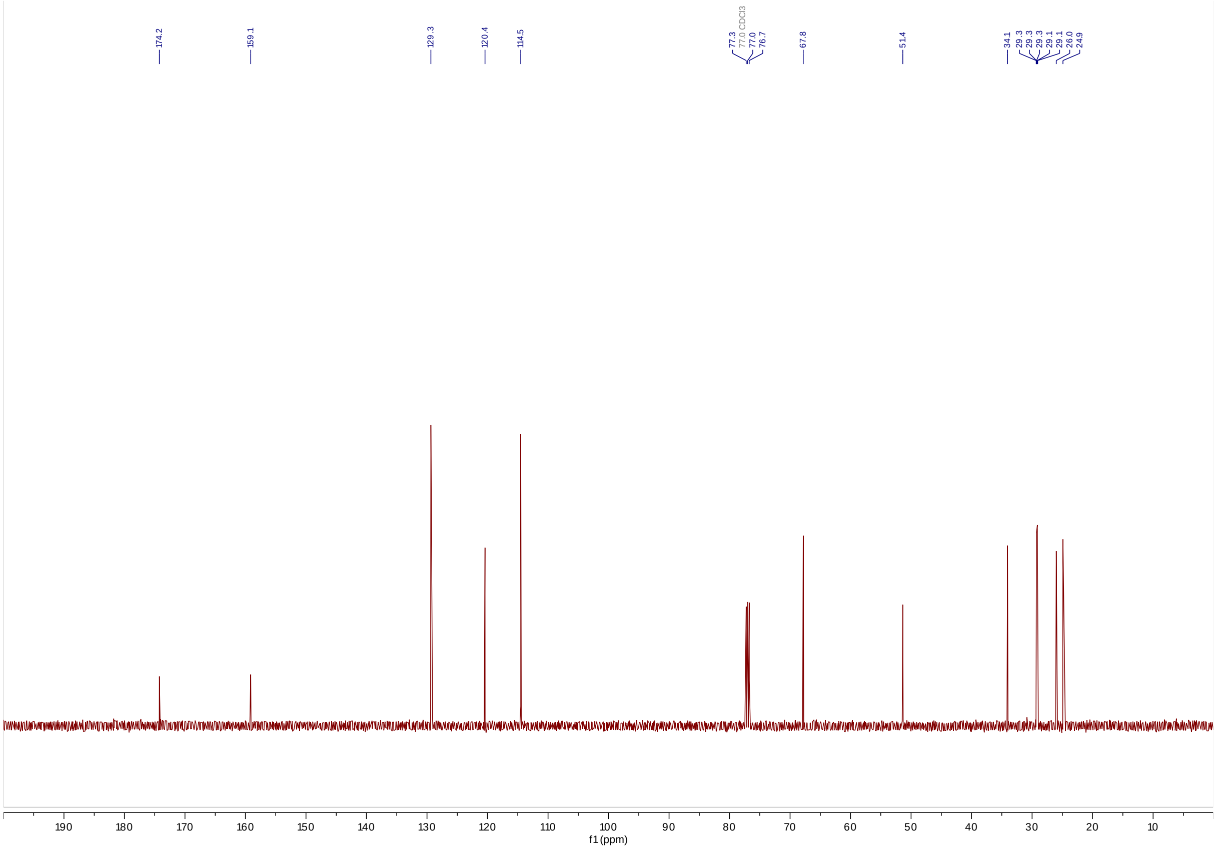

^{1}H-NMR (499.64 MHz, $CDCl_3$): of **Compound 32** (see Procedure)

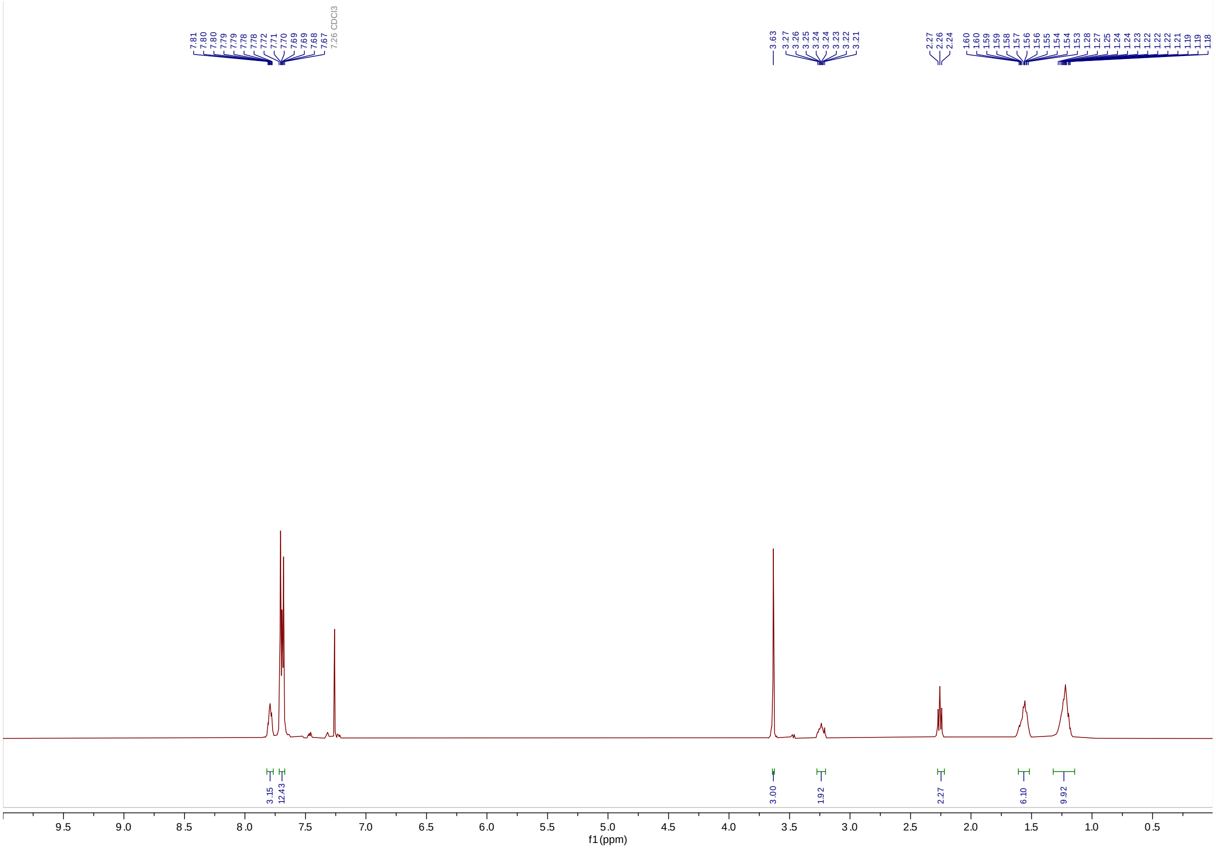

^{13}C-NMR (125.65 MHz, $CDCl_3$) of **Compound 32** (see Procedure)

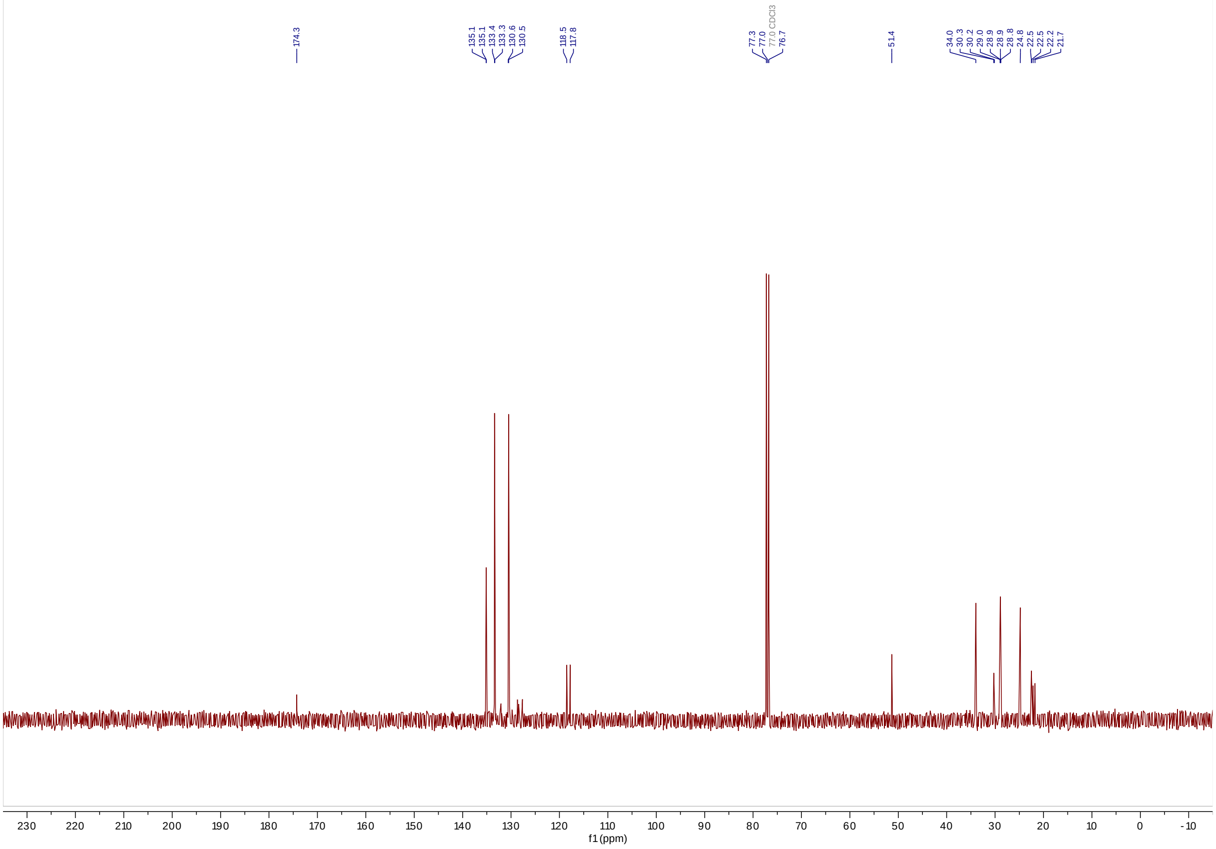

^{19}**F-NMR** (282.21 MHz, $CDCl_3$) of **Compound 32** (see Procedure)

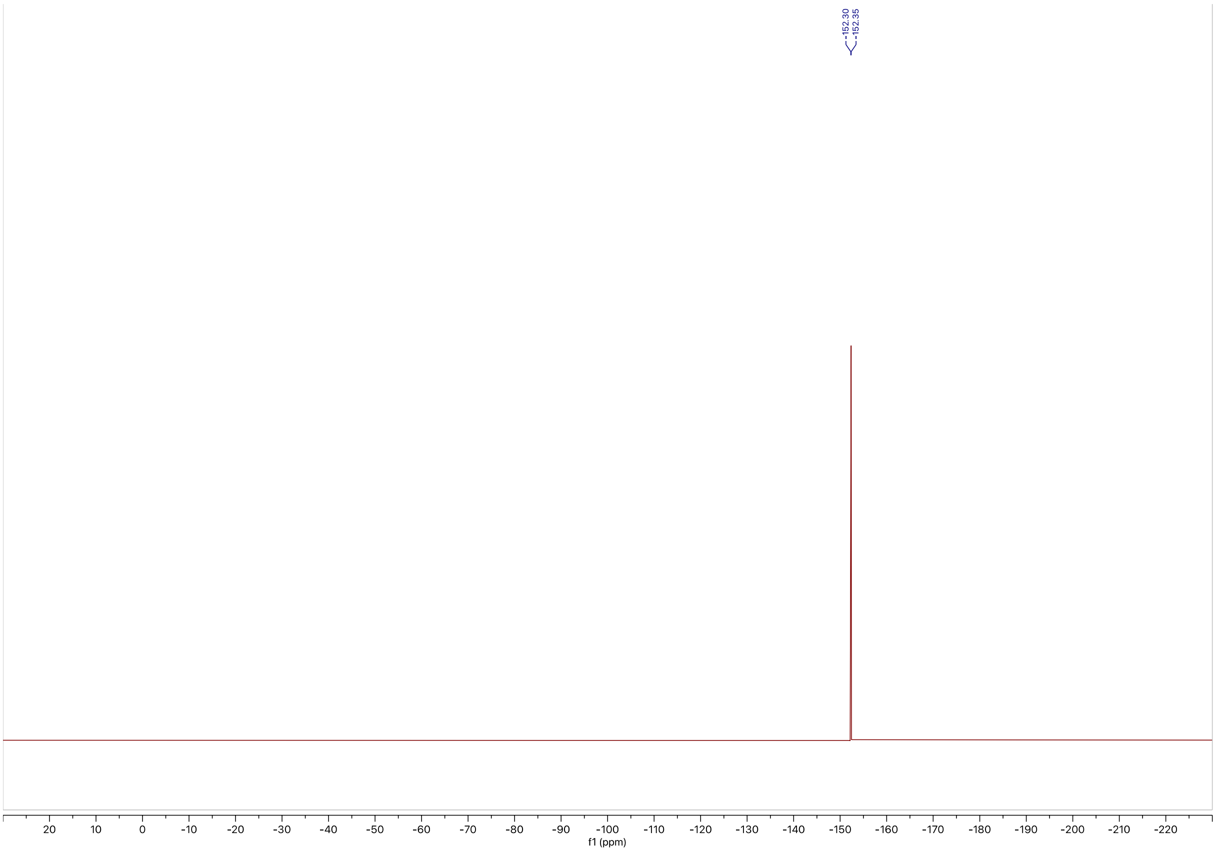

^{31}**P-NMR** (202.27 MHz, $CDCl_3$) of **Compound 32** (see Procedure)

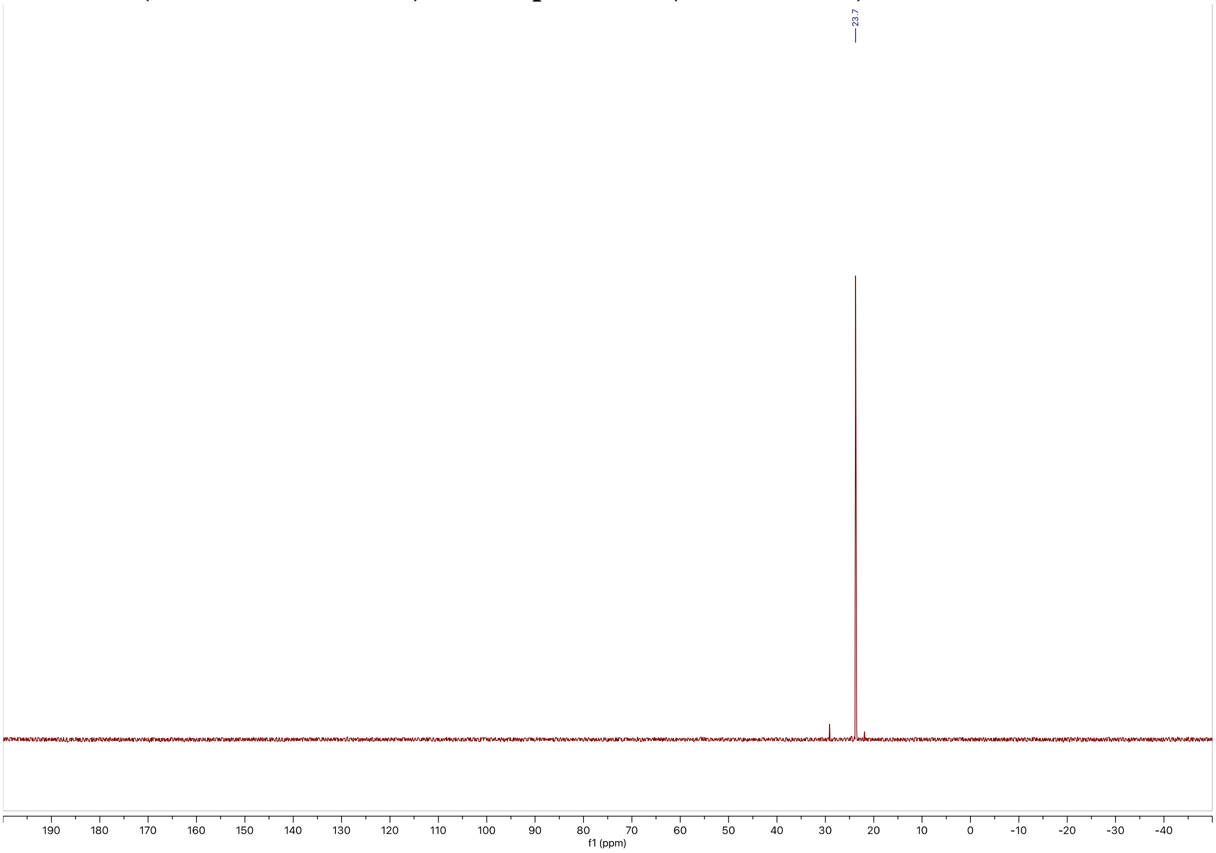

¹H-NMR (499.64 MHz, $CDCl_3$): of **Compound 33** (see Procedure)

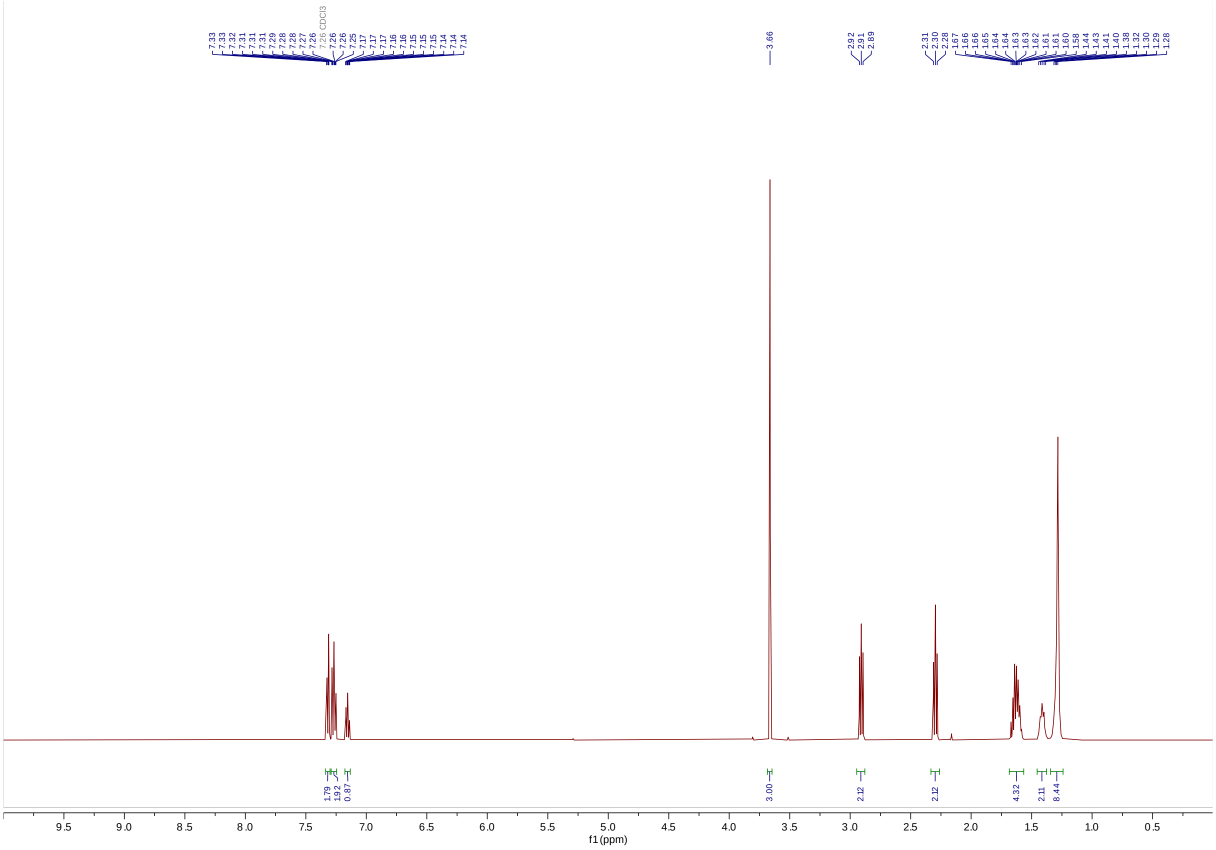

¹³C-NMR (125.65 MHz, $CDCl_3$) of **Compound 33** (see Procedure)

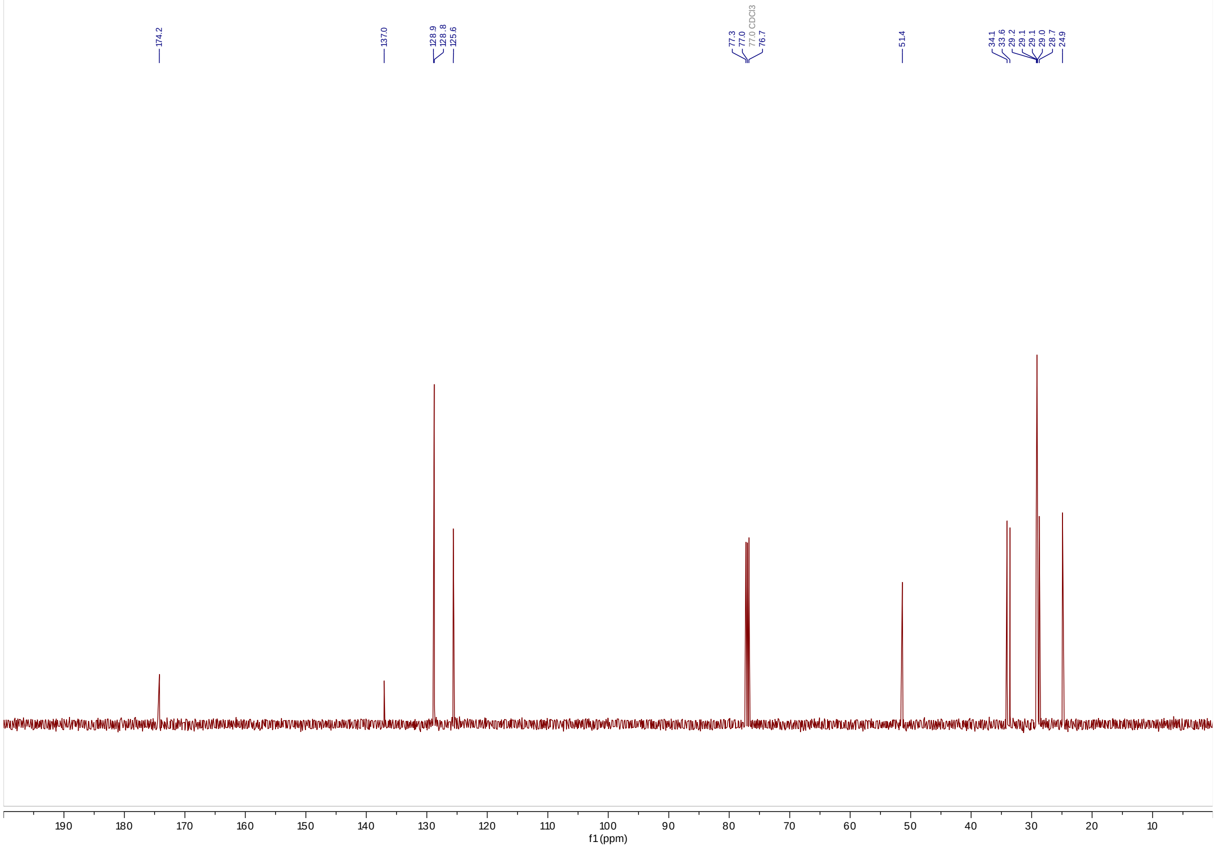

¹H-NMR (499.64 MHz, $CDCl_3$): of **Compound 34** (see Procedure)

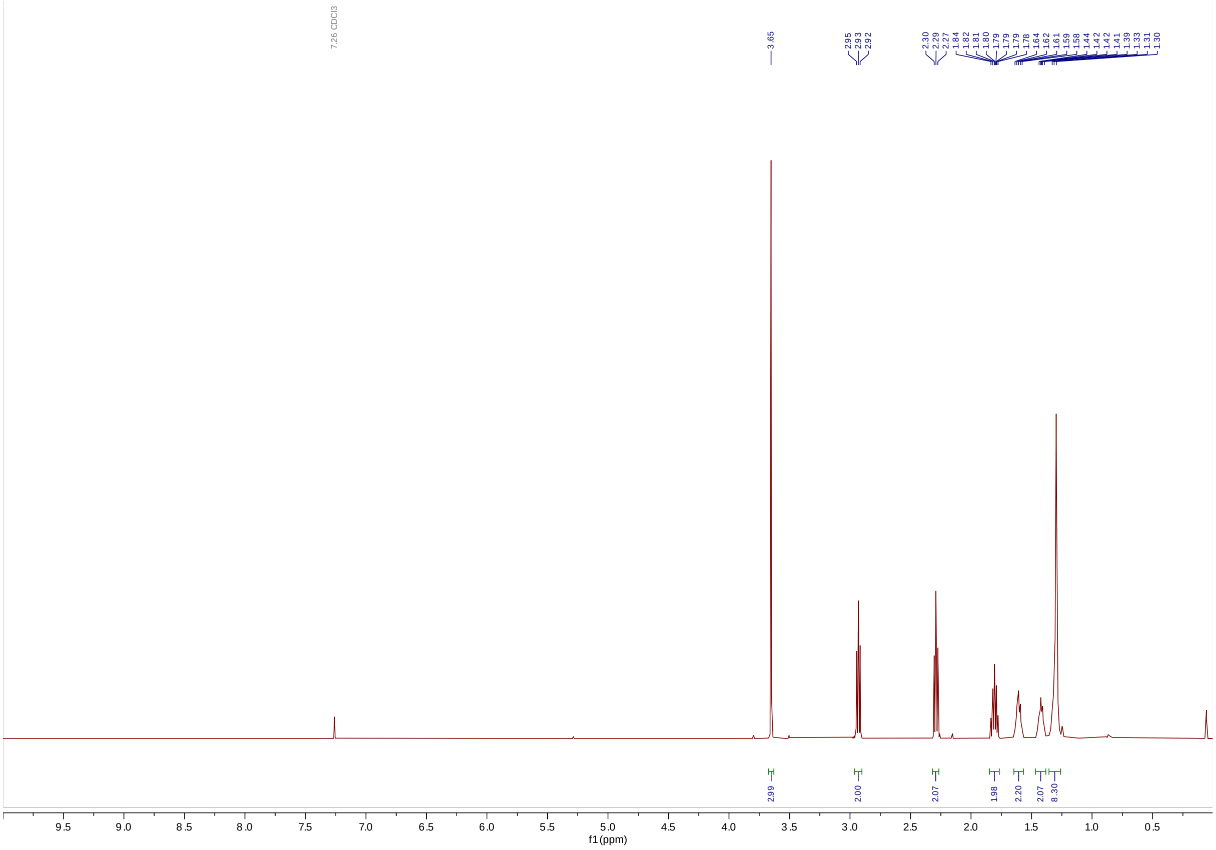

¹³C-NMR (125.65 MHz, $CDCl_3$) of **Compound 34** (see Procedure)

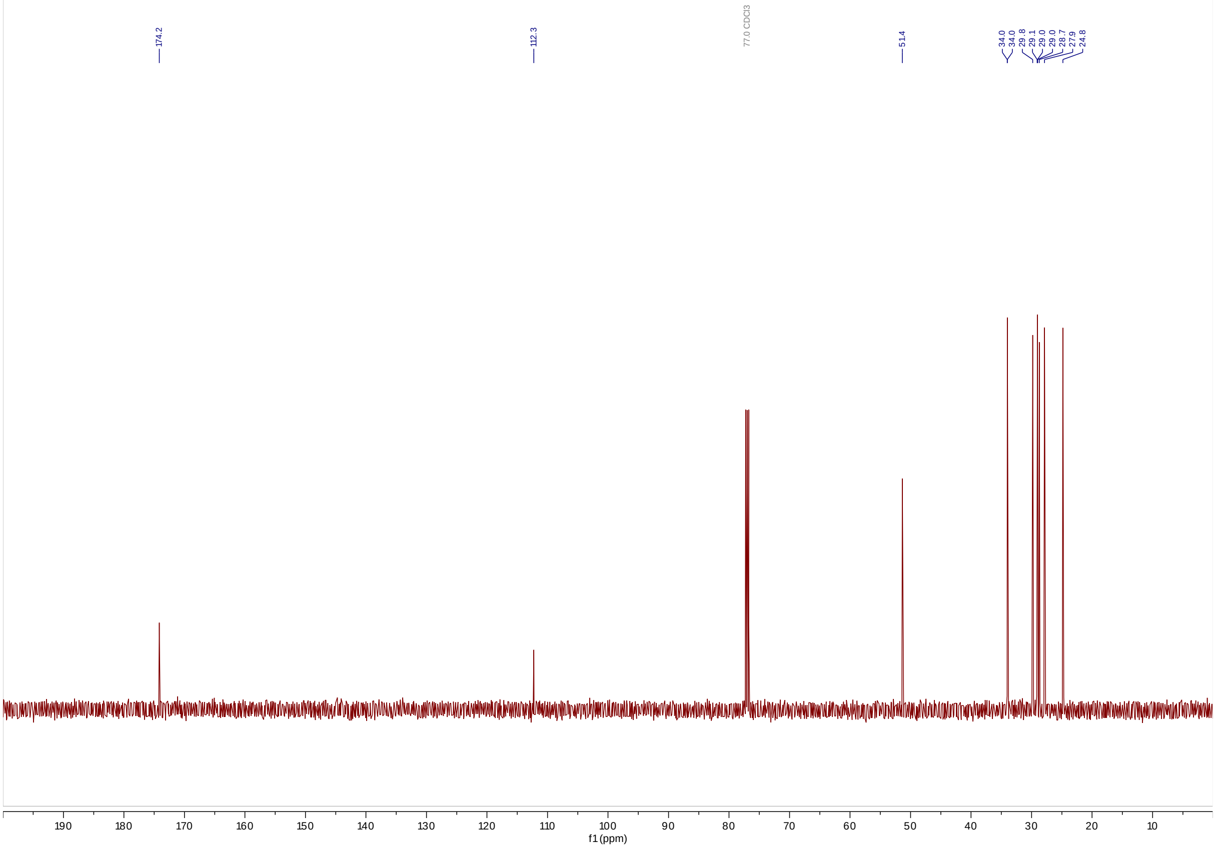

^{1}H-NMR (499.64 MHz, $CDCl_3$): of **Compound 35** (see Procedure)

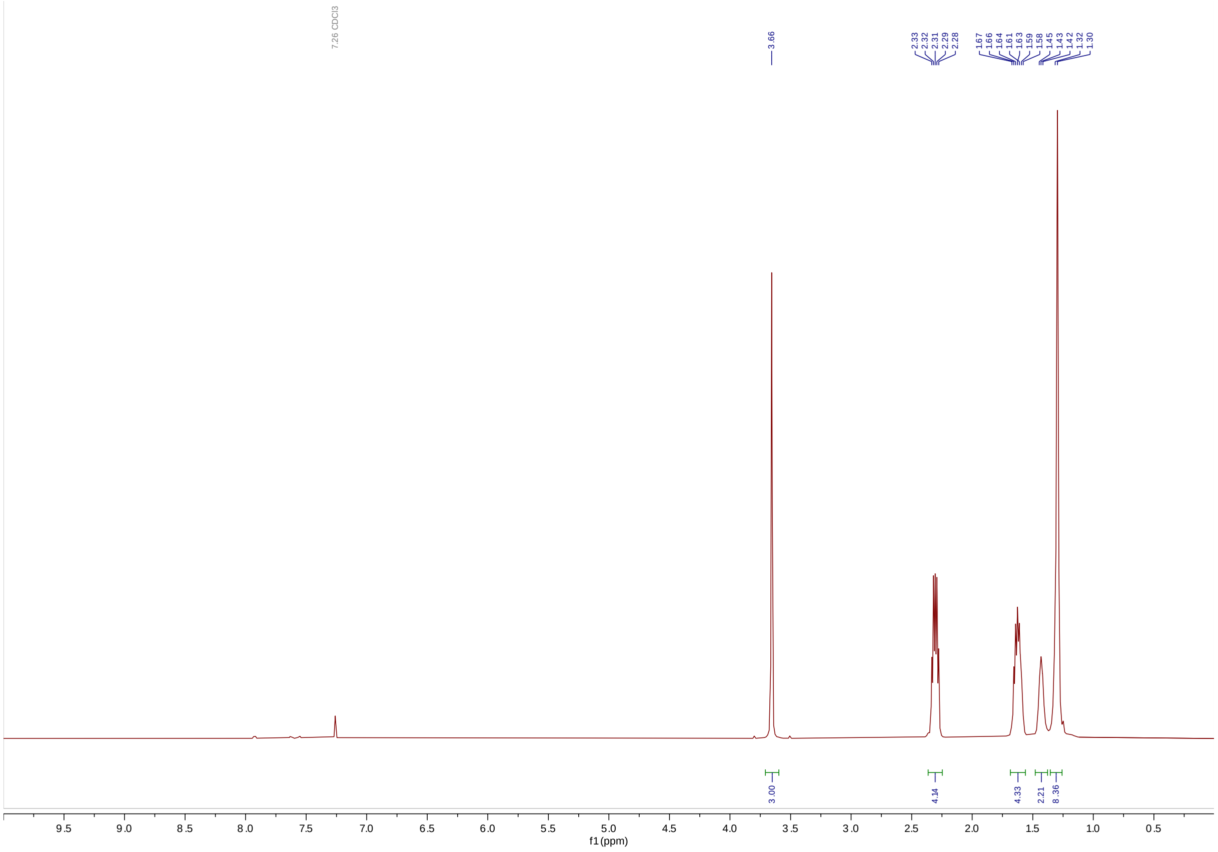

^{13}C-NMR (125.65 MHz, $CDCl_3$) of **Compound 35** (see Procedure)

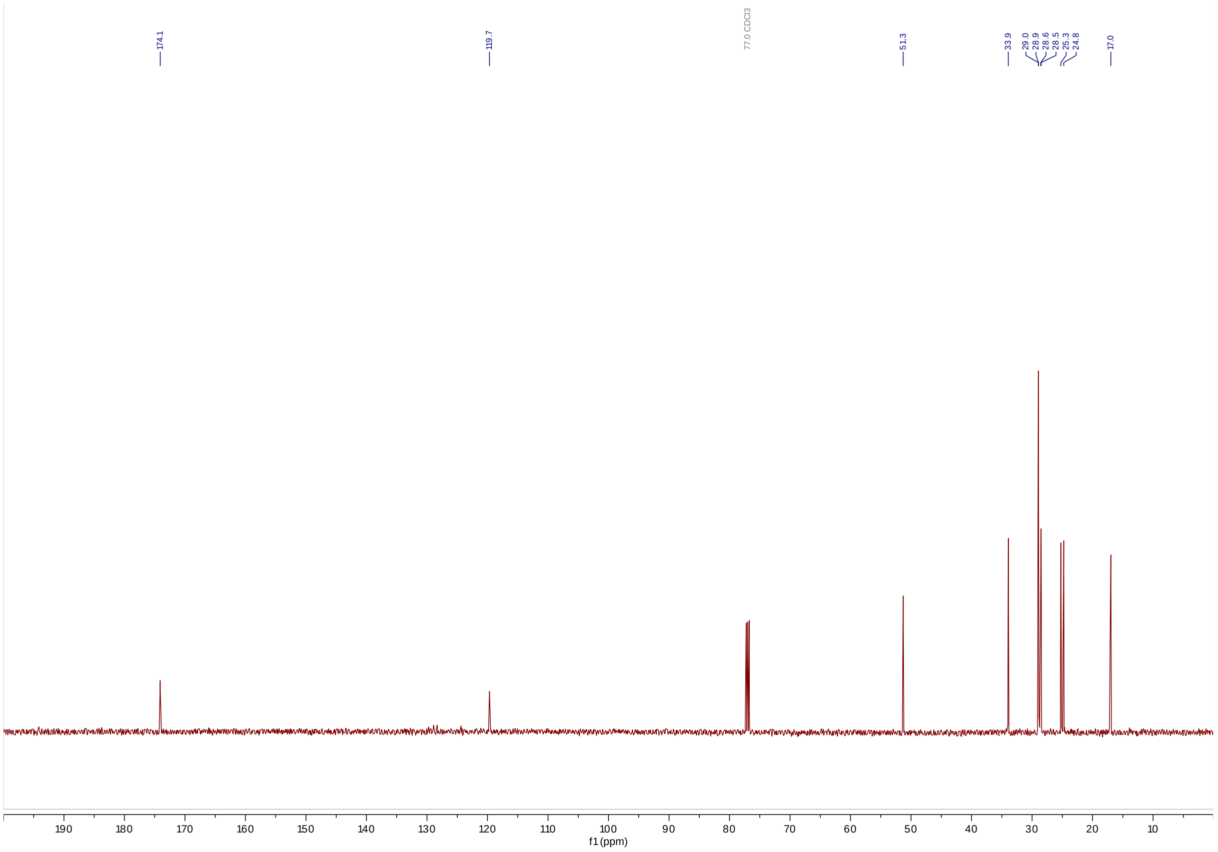

¹H-NMR (499.64 MHz, $CDCl_3$): of **Compound 36** (see Procedure)

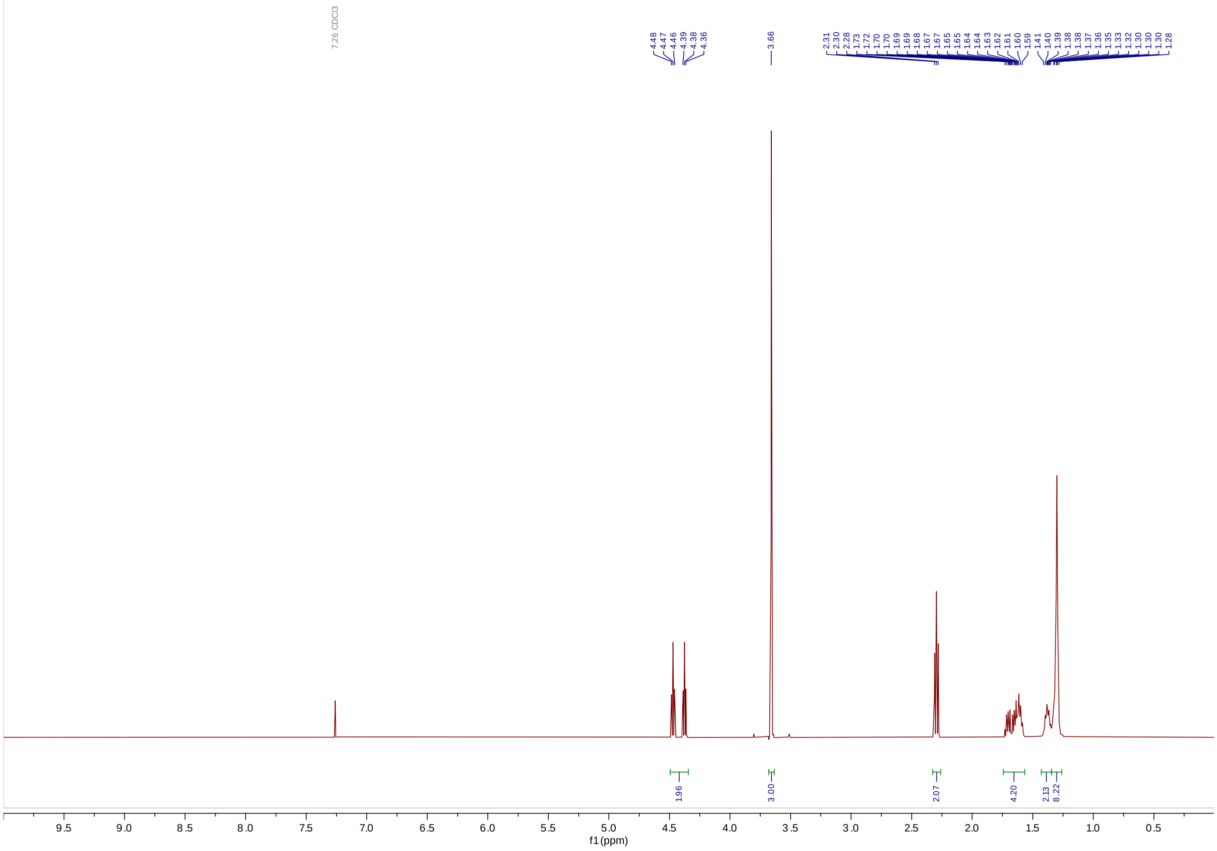

¹³C-NMR (125.65 MHz, $CDCl_3$) of **Compound 36** (see Procedure)

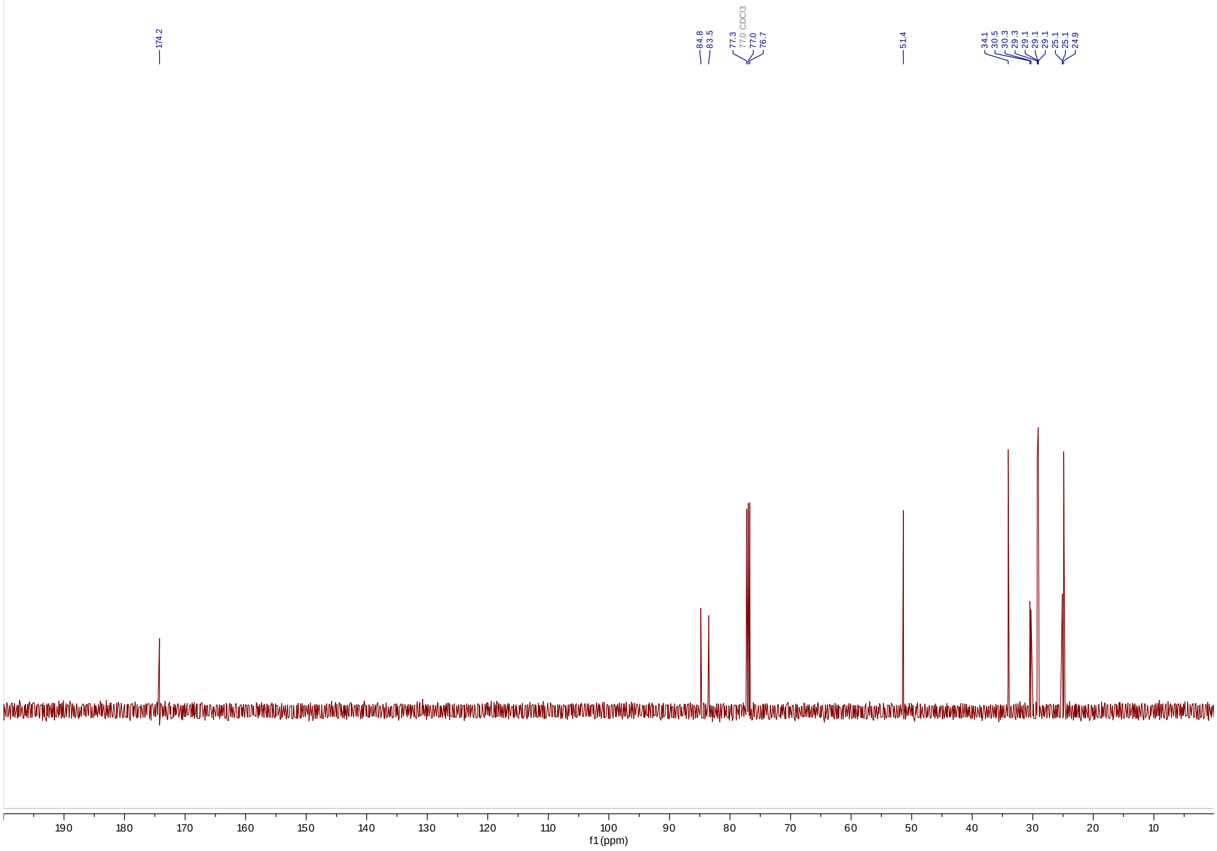

^{19}F-NMR (282.21 MHz, $CDCl_3$) of **Compound 36** (see Procedure)

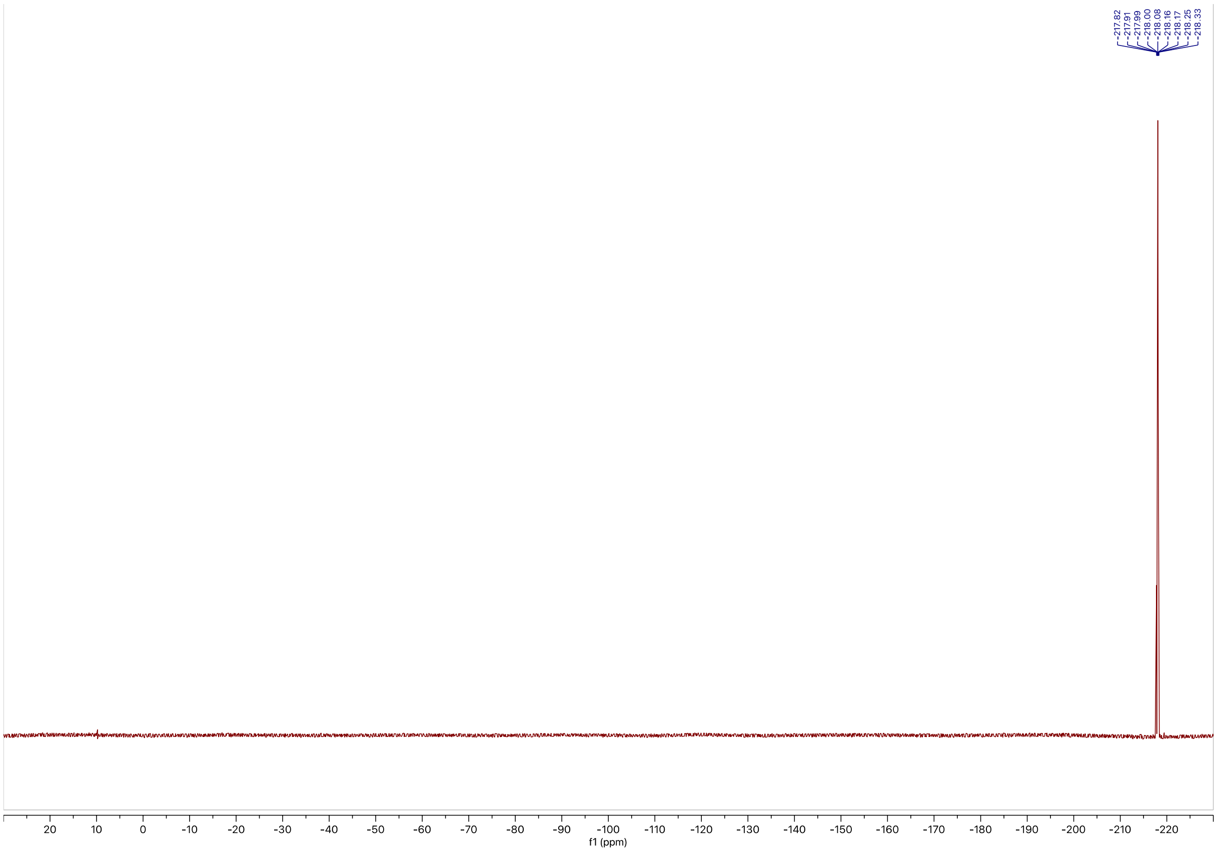

^{1}H-NMR (499.64 MHz, $CDCl_3$): of **Compound 37** (see Procedure)

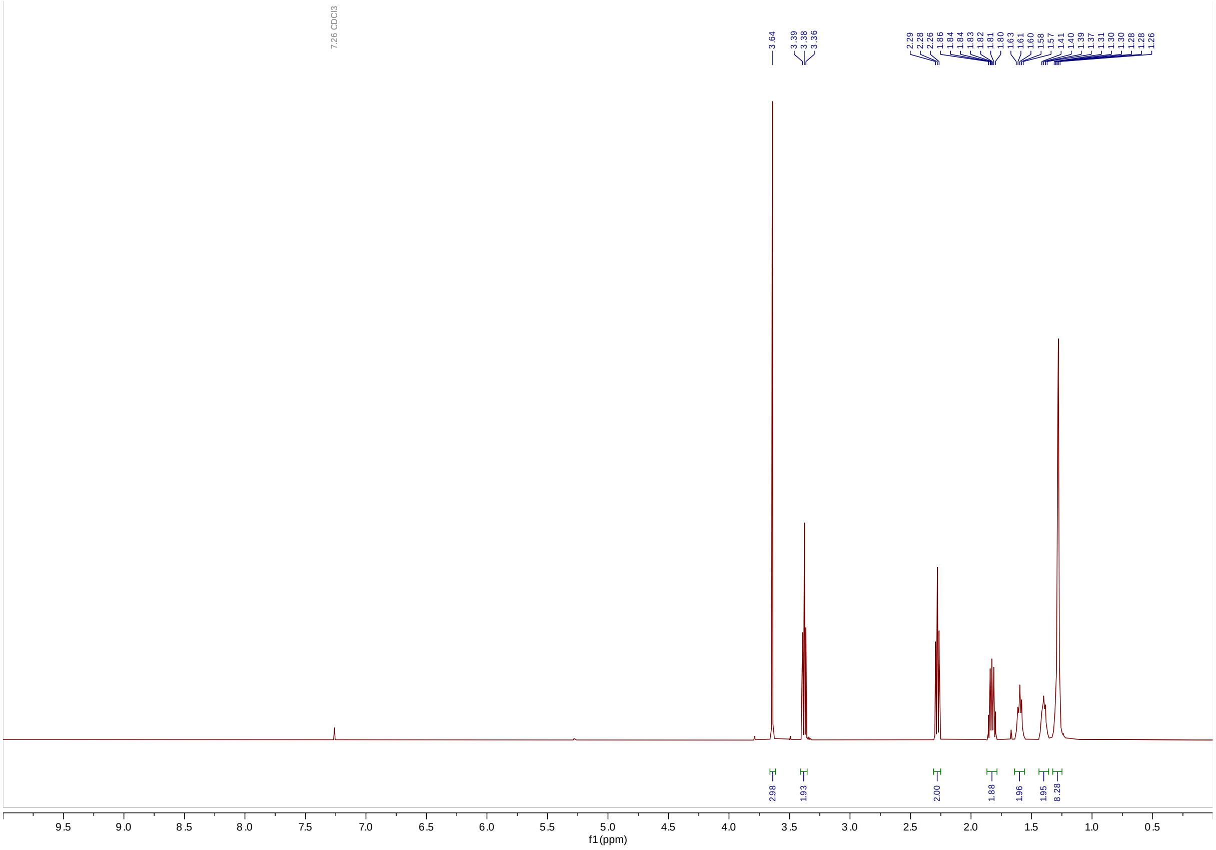

^{13}C-NMR (125.65 MHz, $CDCl_3$) of **Compound 37** (see Procedure)

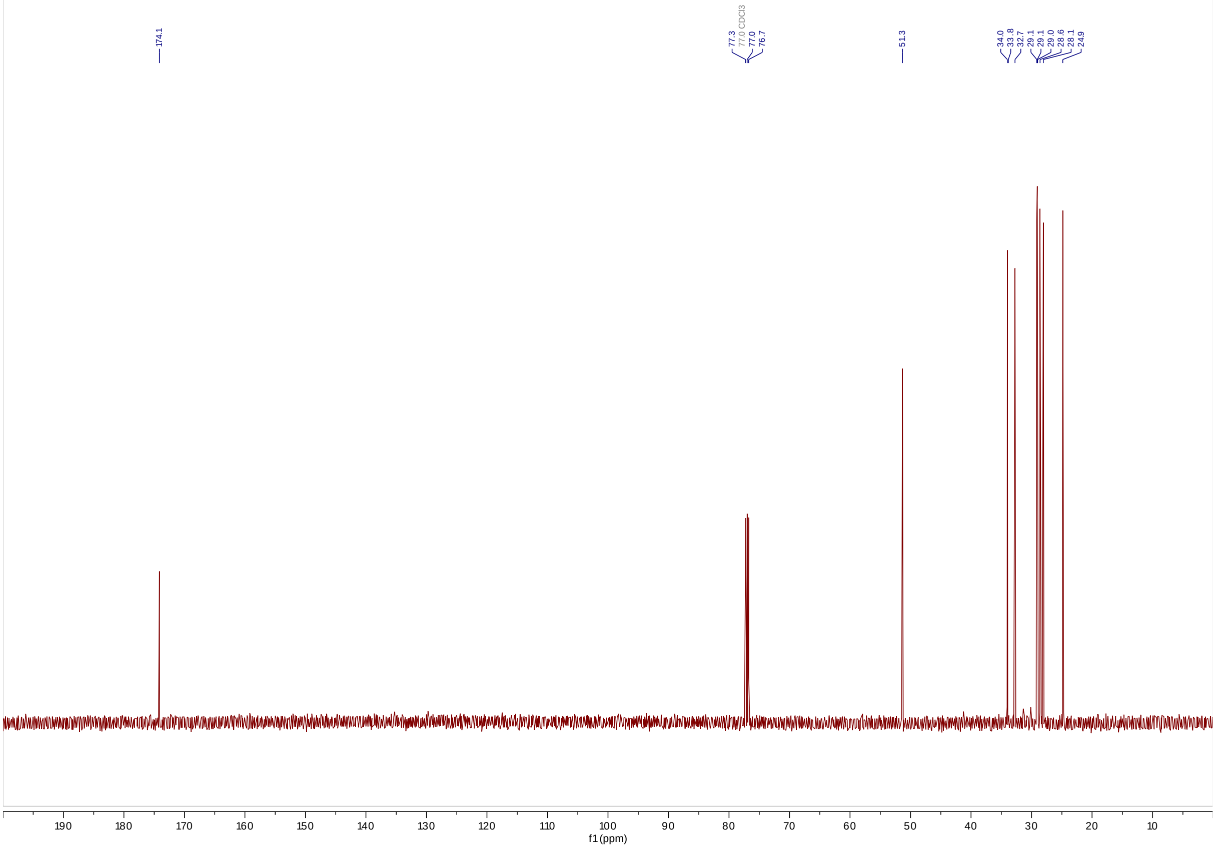

¹H-NMR (499.64 MHz, $CDCl_3$): of **Compound 38** (see Procedure)

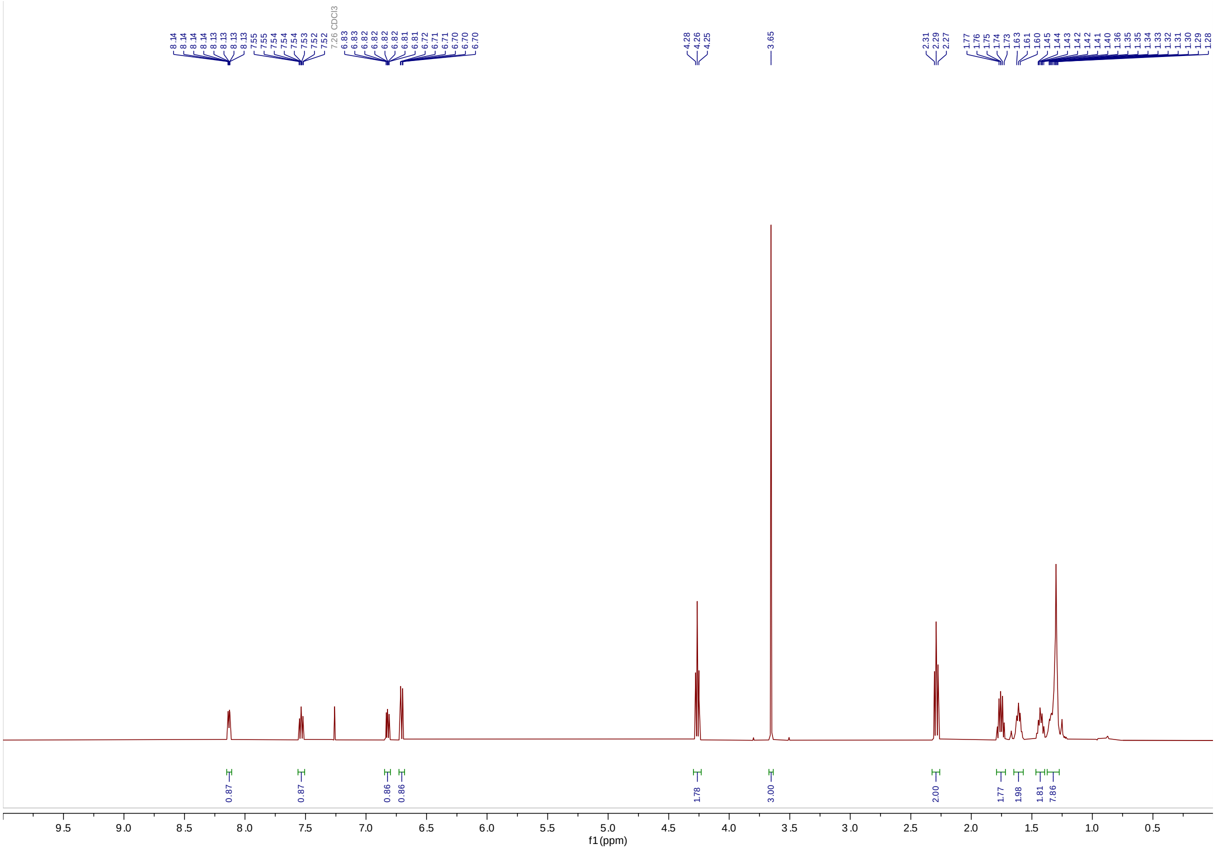

¹³C-NMR (125.65 MHz, $CDCl_3$) of **Compound 38** (see Procedure)

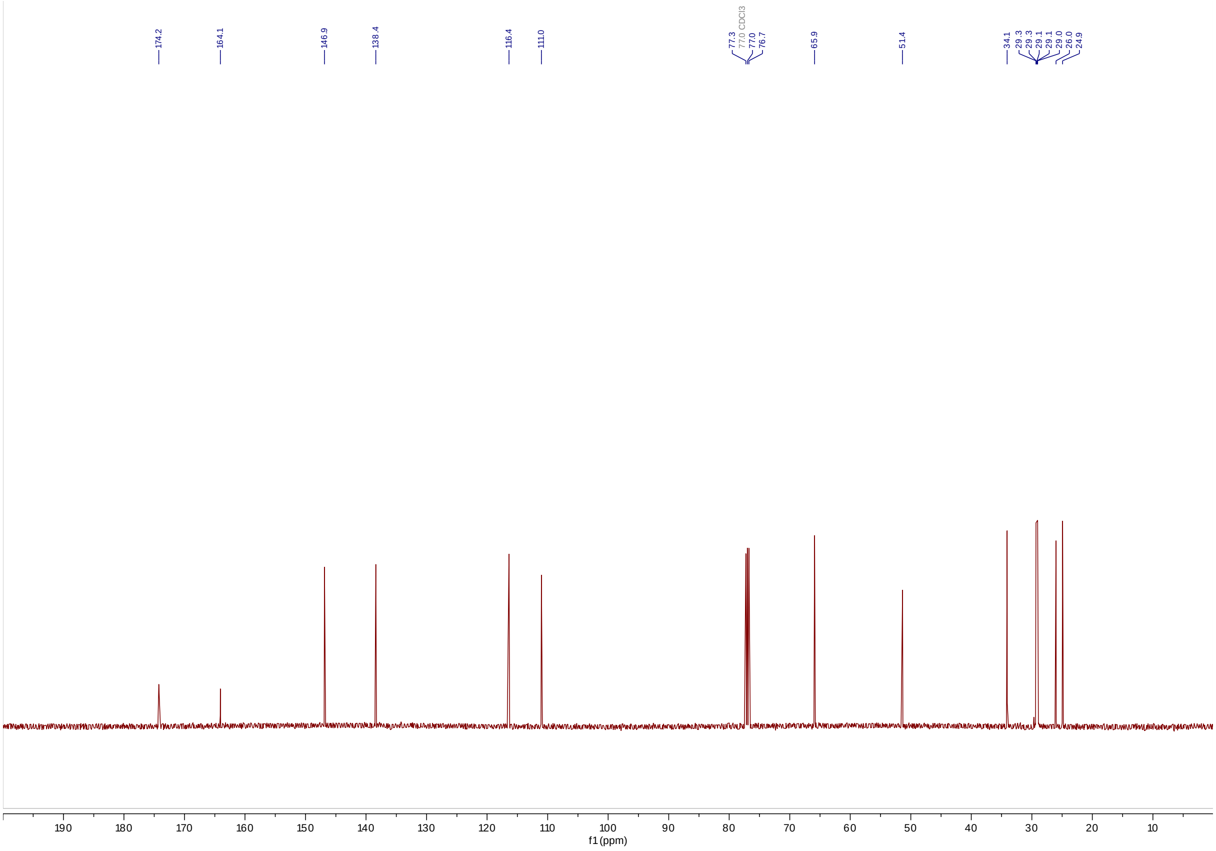

^{1}H-NMR (599.63 MHz, $CDCl_3$): of **Compound 39** (see Procedure)

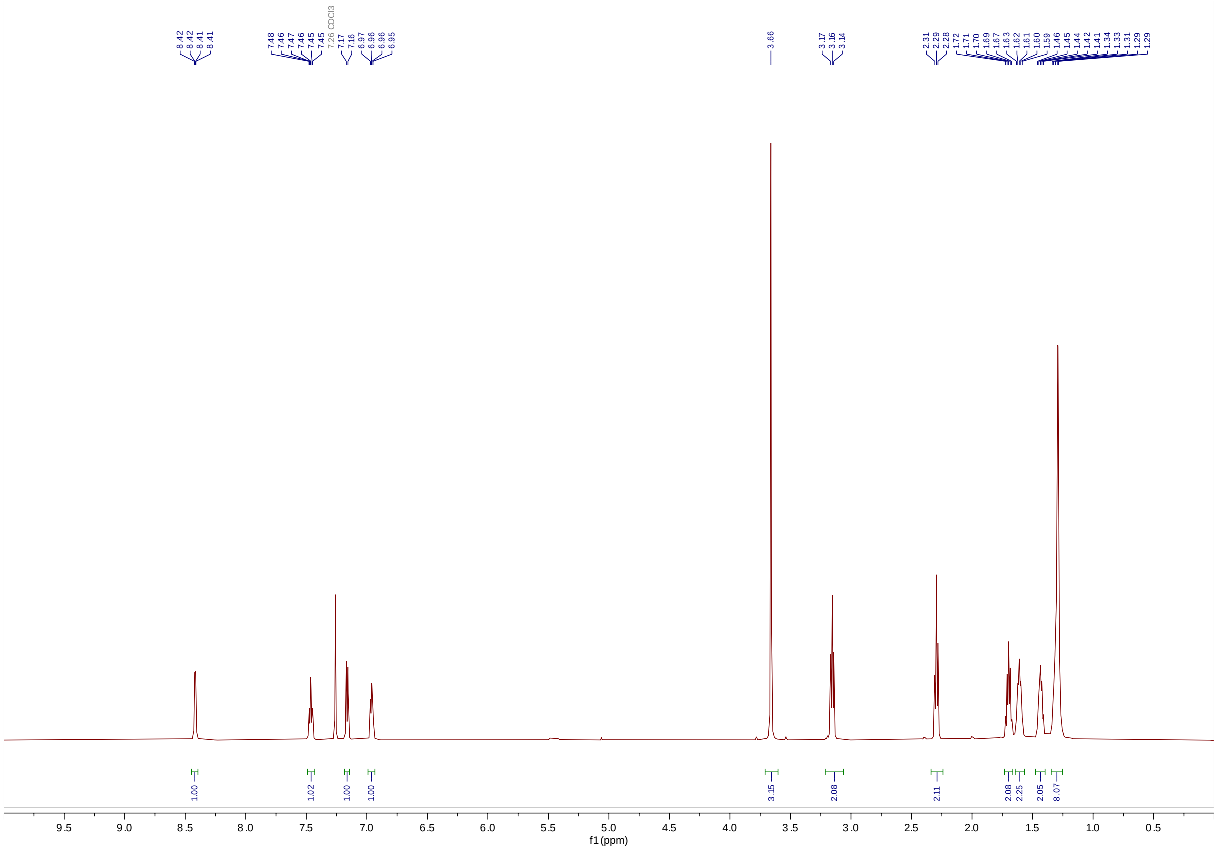

^{13}C-NMR (150.79 MHz, $CDCl_3$) of **Compound 39** (see Procedure)

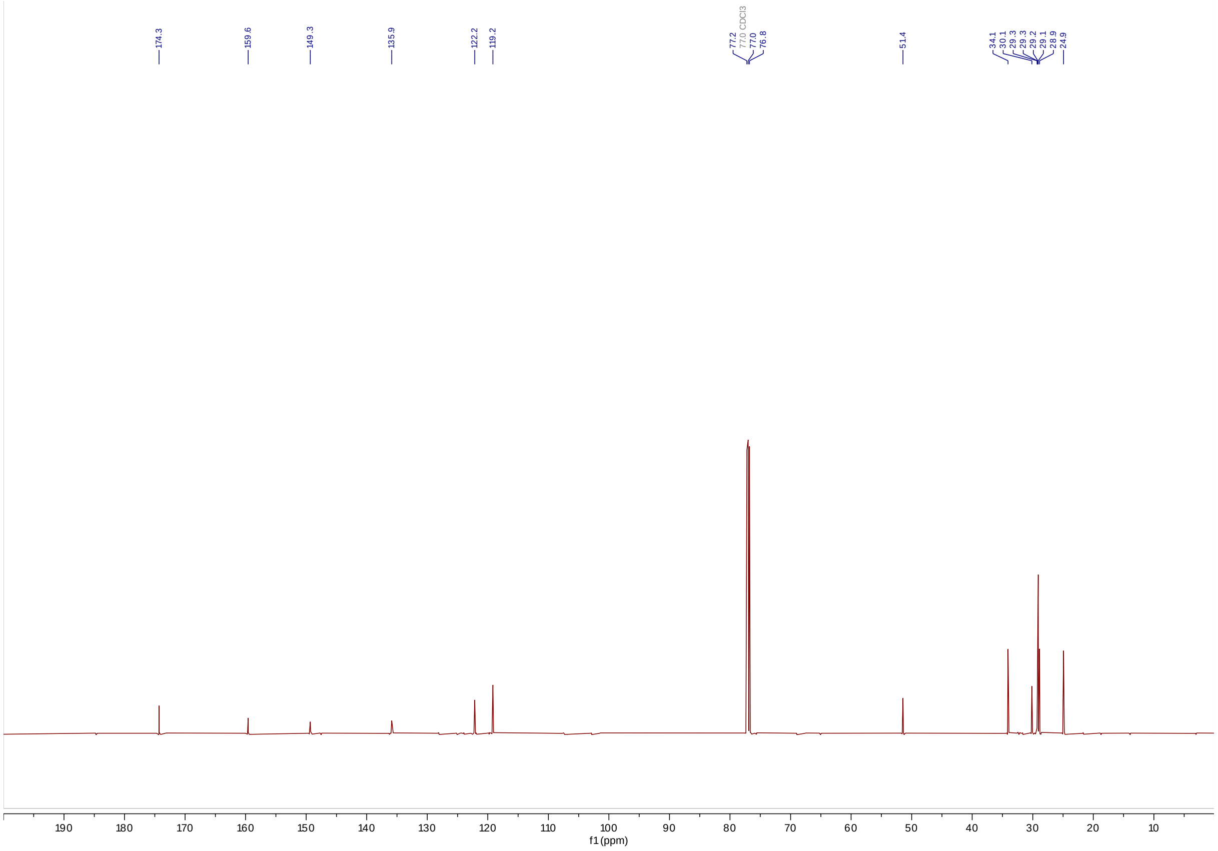

^{1}H-NMR (399.87 MHz, $CDCl_3$): of **Compound 40a** (see Procedure)

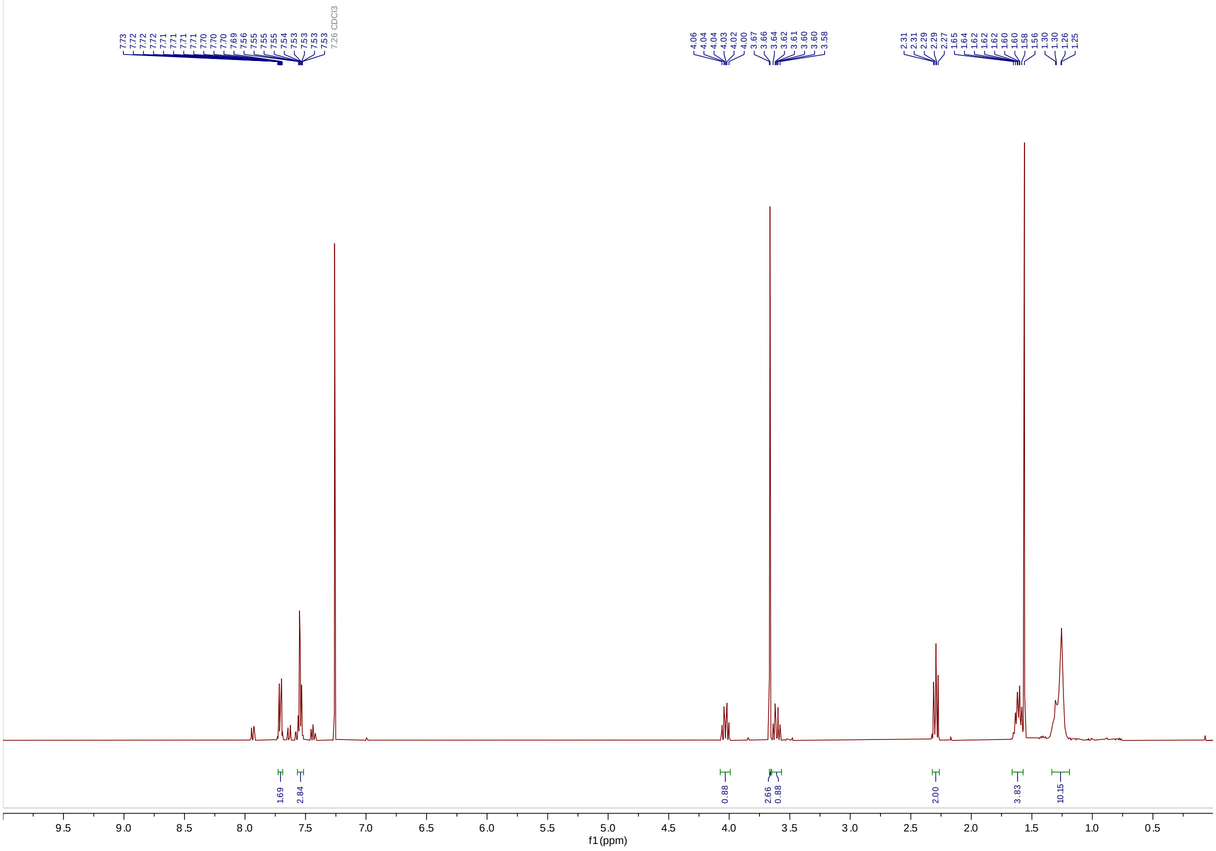

^{13}C-NMR (100.56 MHz, $CDCl_3$) of **Compound 40a** (see Procedure)

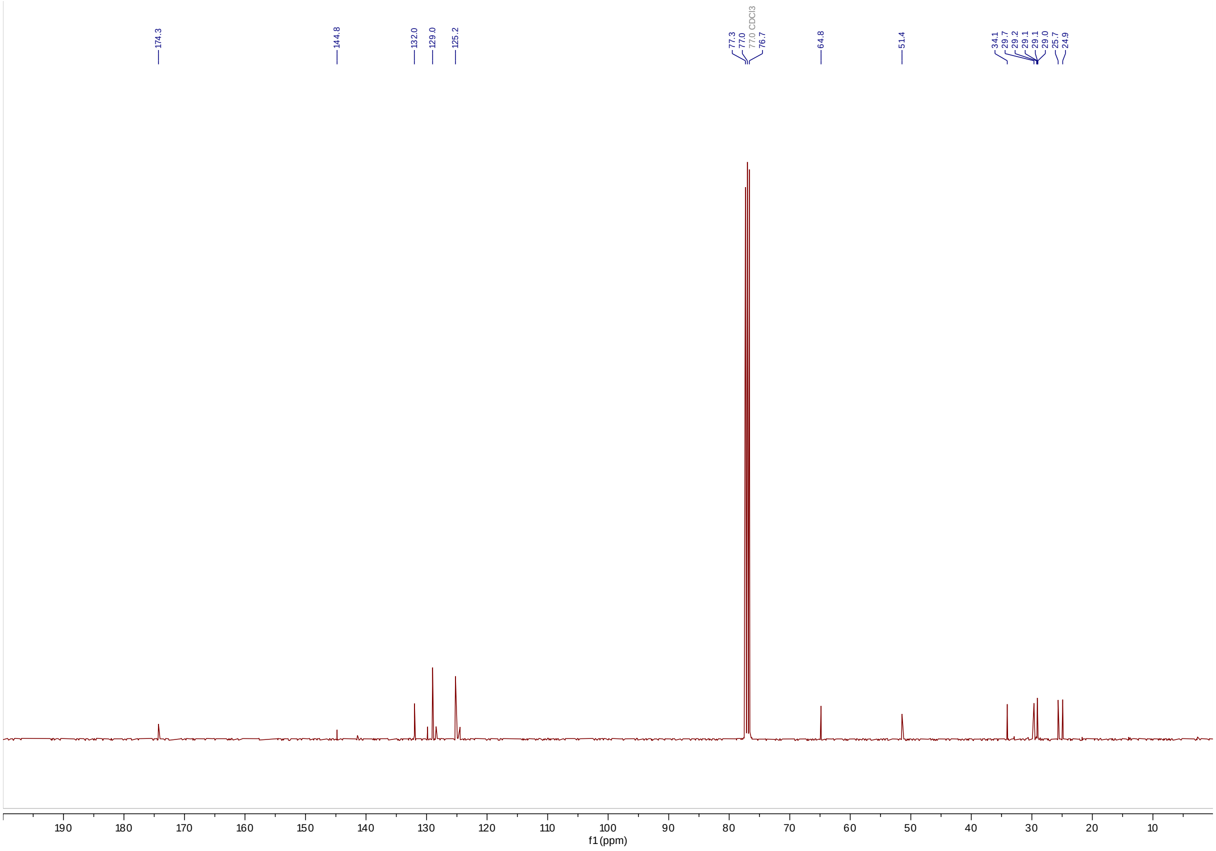

^{1}H-NMR (499.64 MHz, $CDCl_3$): of **Compound 40b** (see Procedure)

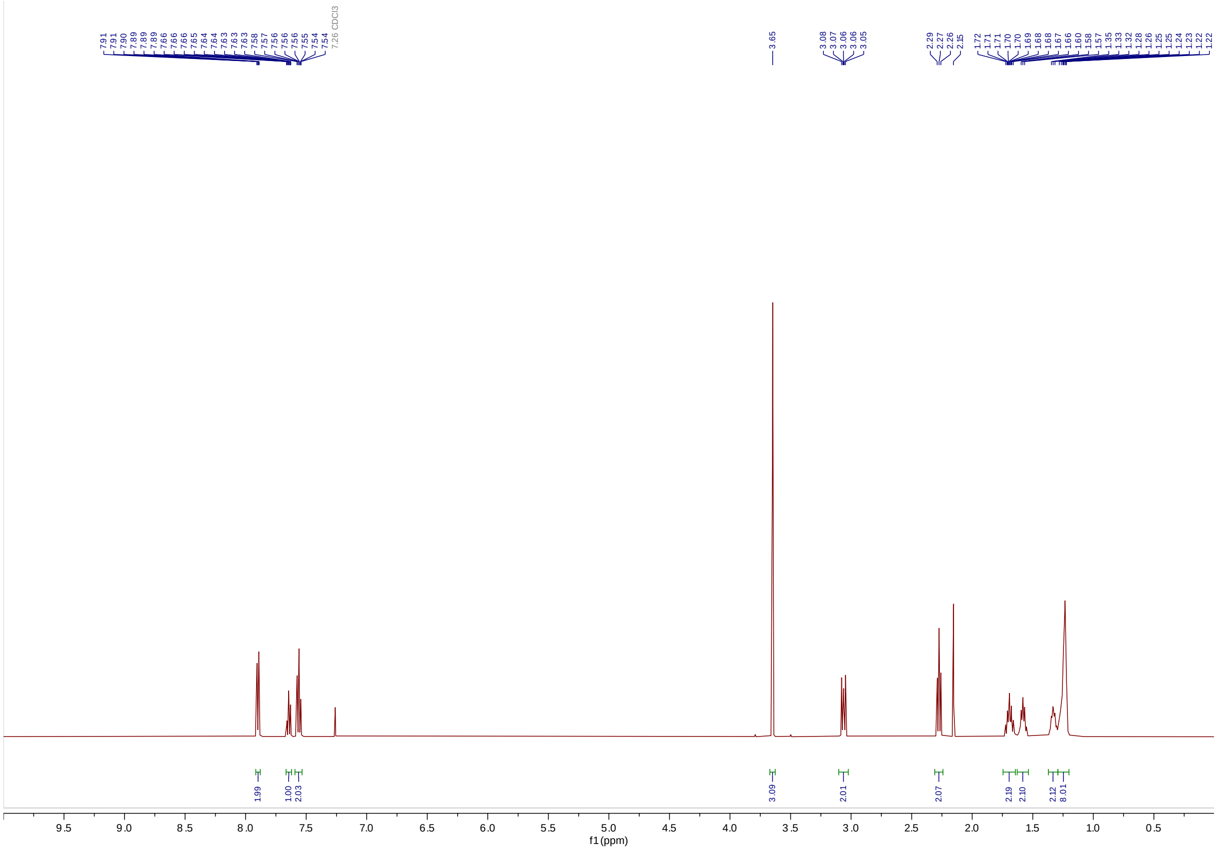

^{13}C-NMR (125.65 MHz, $CDCl_3$) of **Compound 40b** (see Procedure)

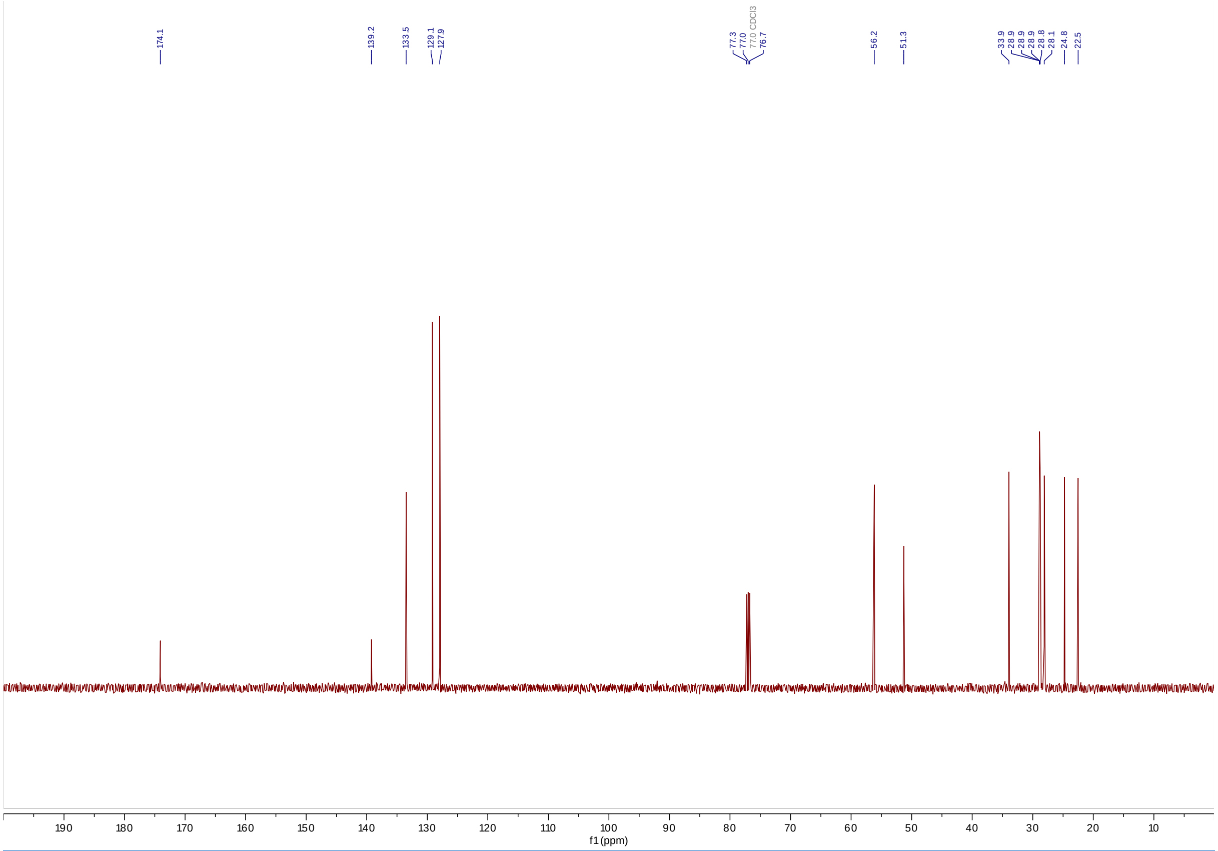

^{1}H-NMR (499.64 MHz, $CDCl_3$): of **Compound 41** (see Procedure)

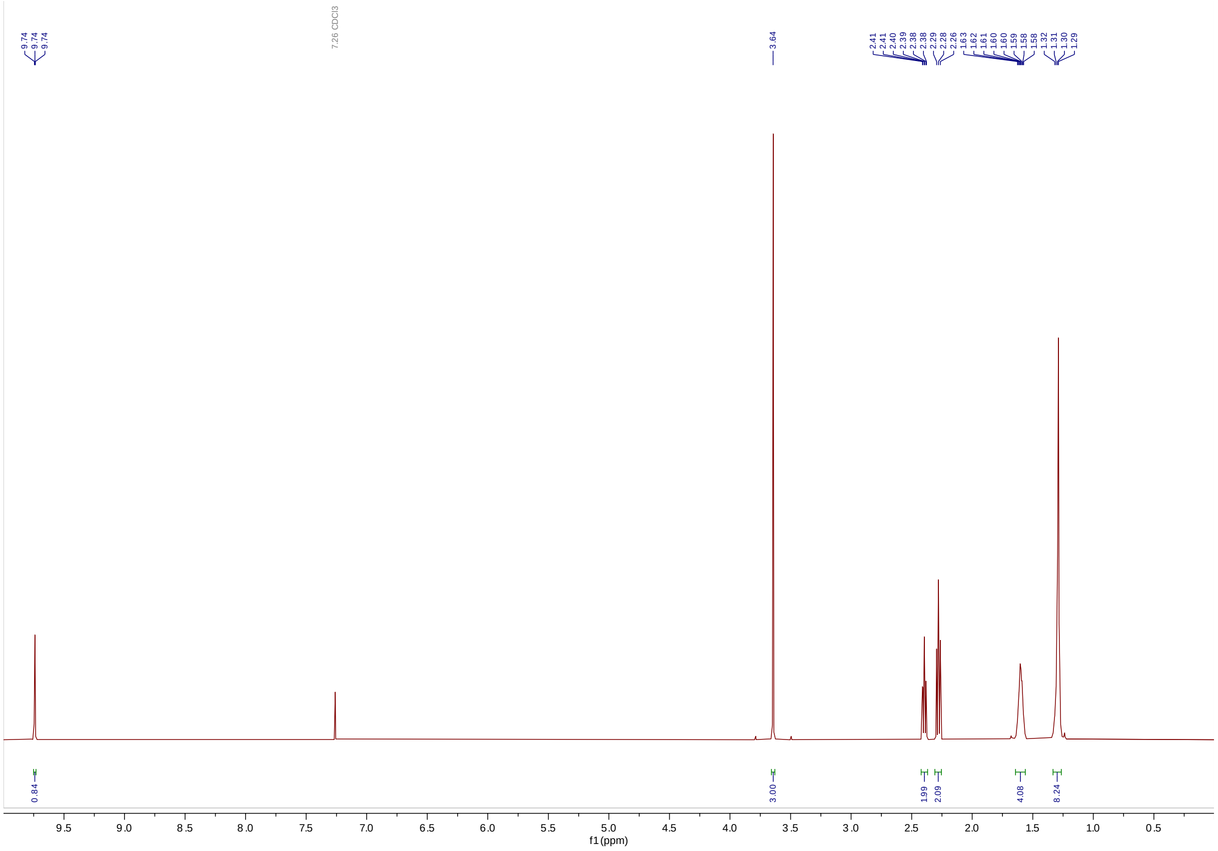

^{13}C-NMR (125.65 MHz, $CDCl_3$) of **Compound 41** (see Procedure)

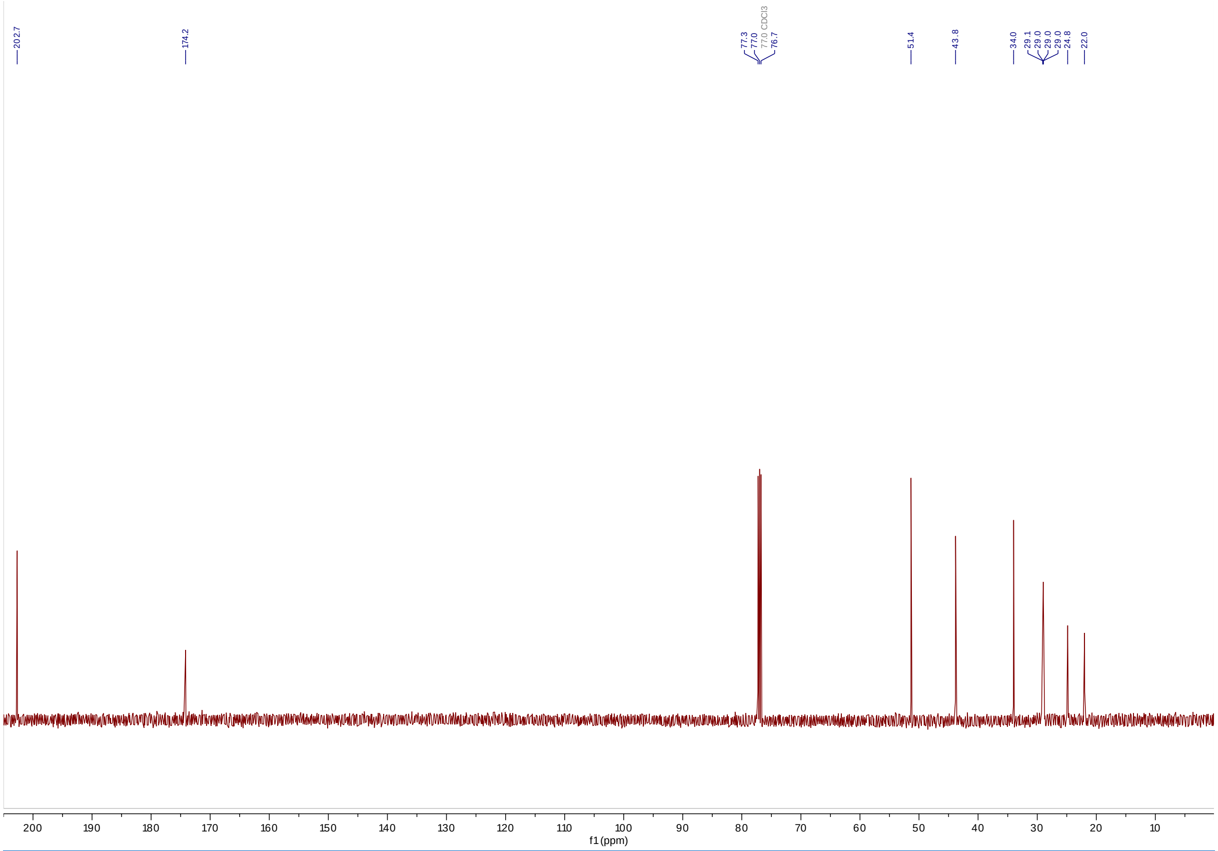

^{1}H-NMR (499.64 MHz, $CDCl_3$): of **Compound 46** (see Procedure)

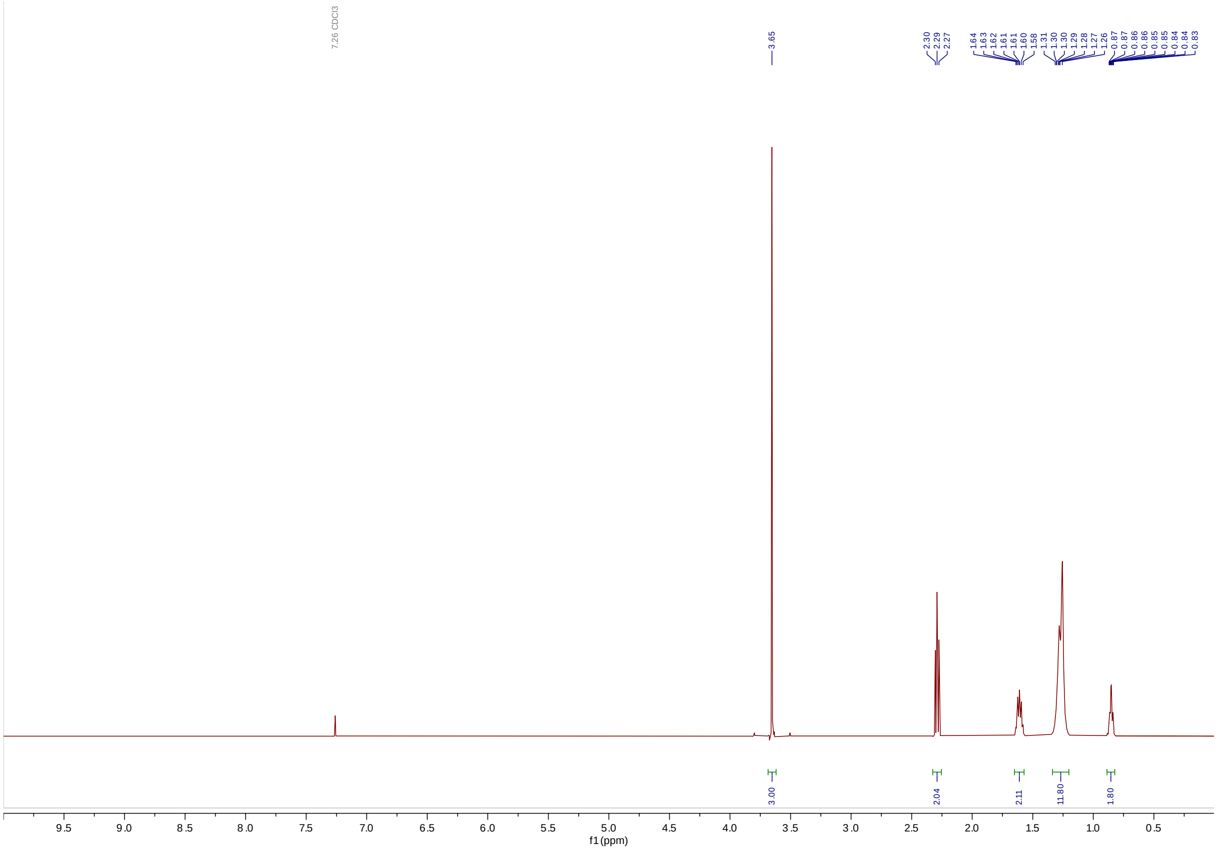

^{13}C-NMR (125.65 MHz, $CDCl_3$) of **Compound 46** (see Procedure)

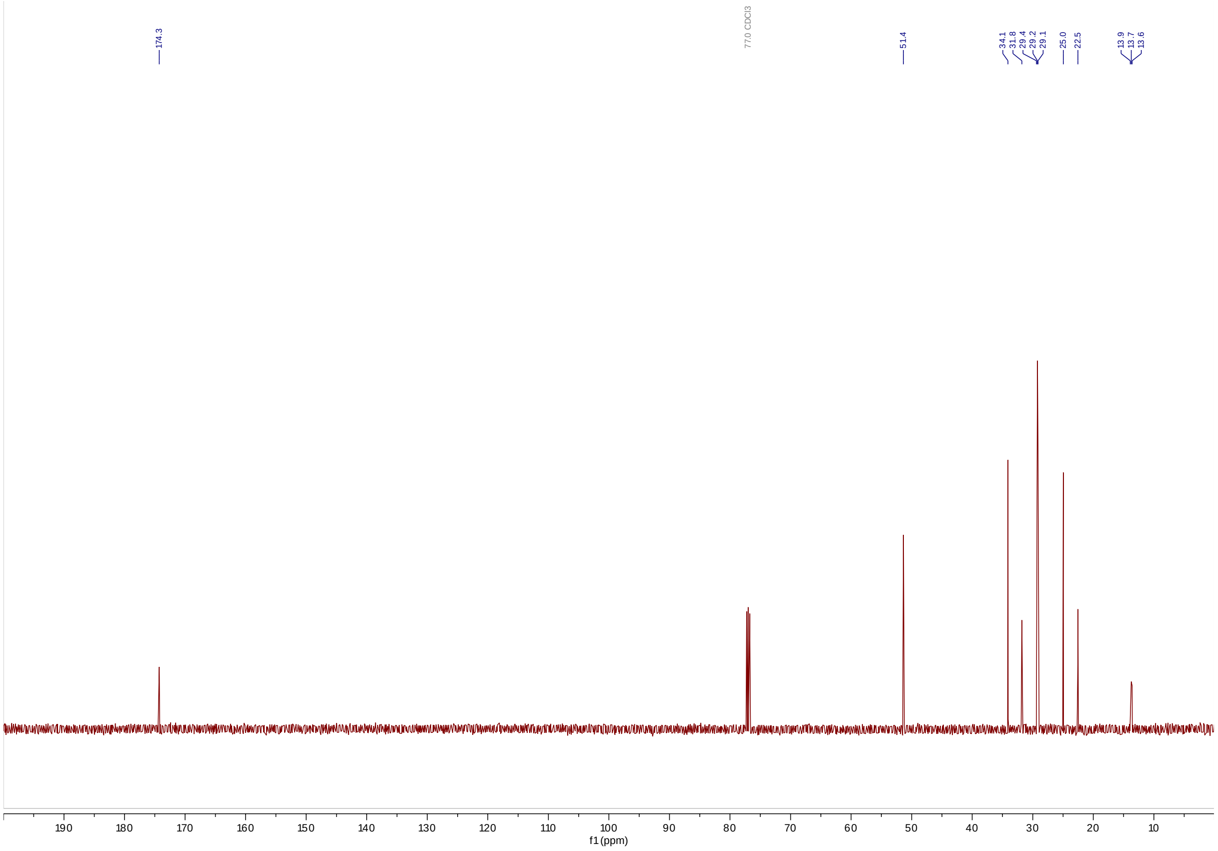

^{1}H-NMR (499.64 MHz, $CDCl_3$): of **Compound 47** (see Procedure)

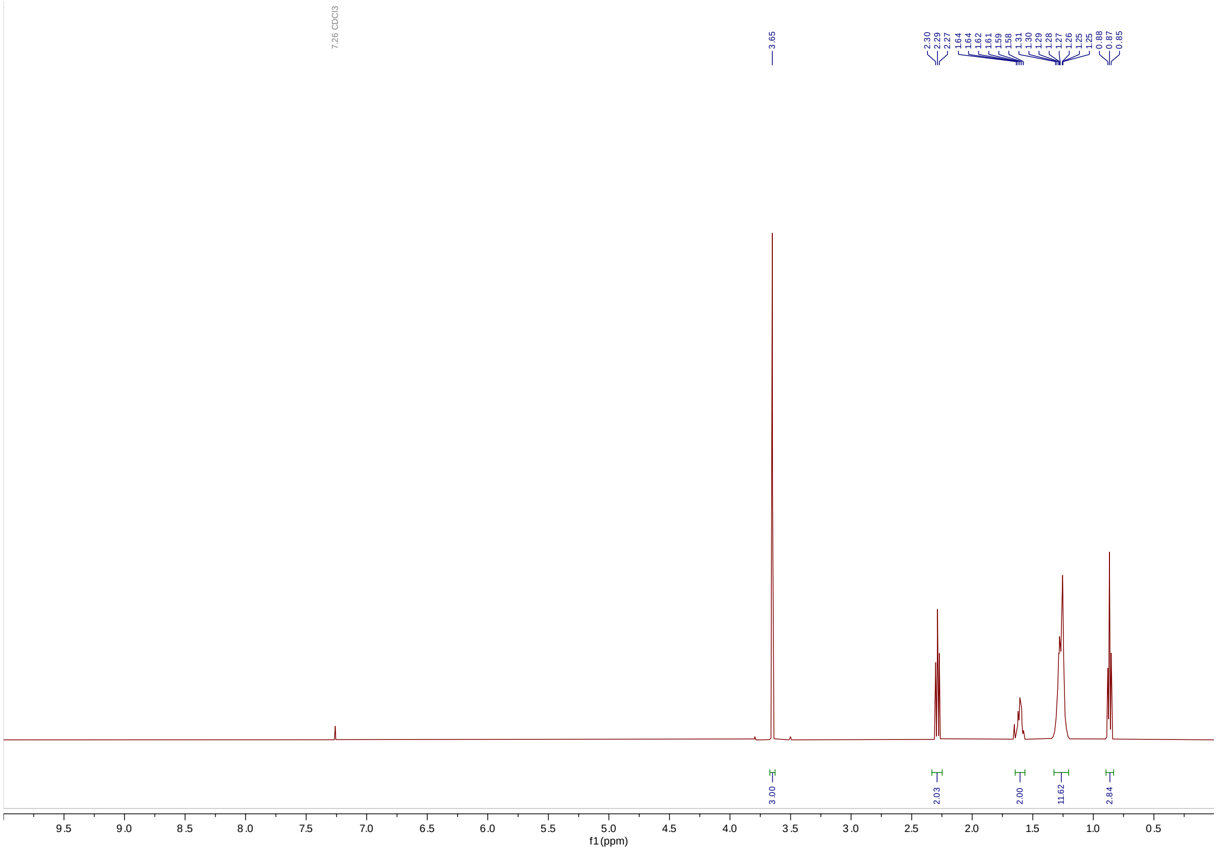

^{13}C-NMR (125.65 MHz, $CDCl_3$) of **Compound 47** (see Procedure)

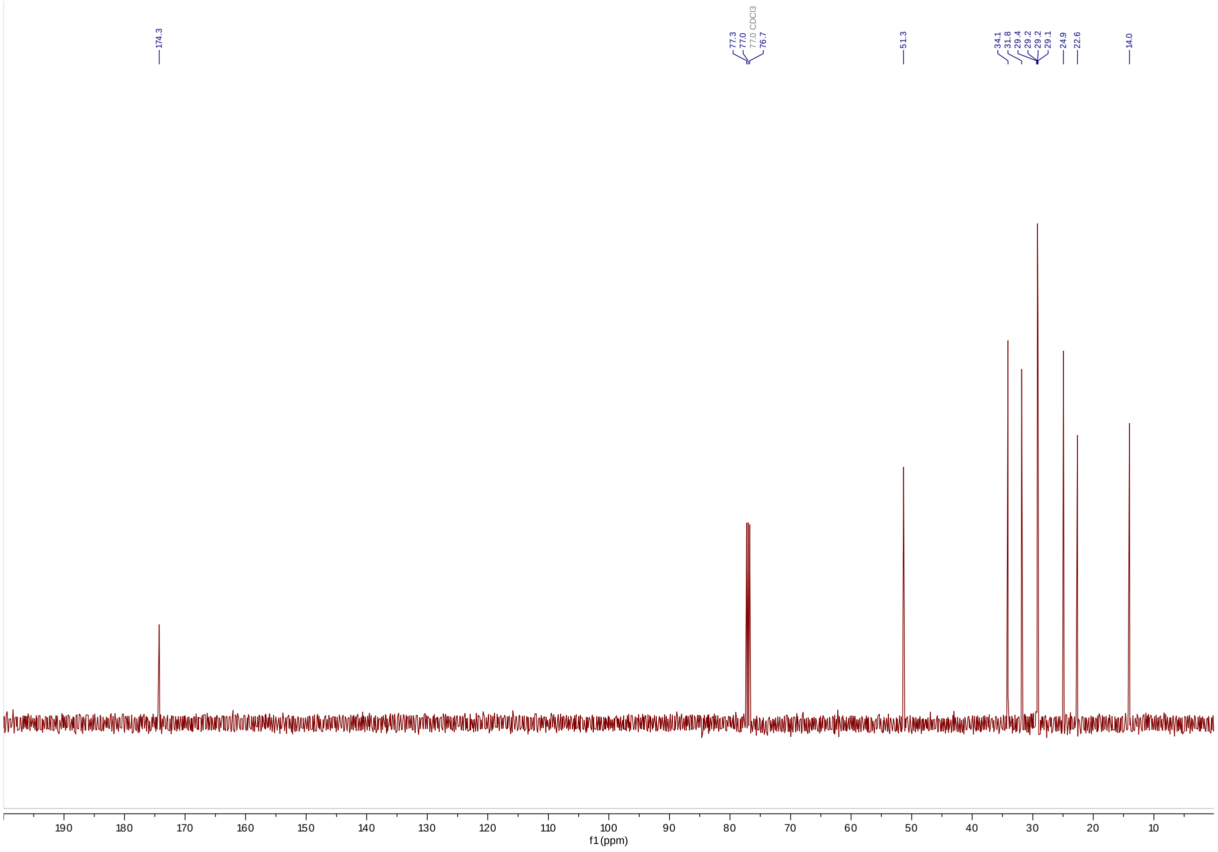

^{1}H-NMR (499.64 MHz, $CDCl_3$): of **Compound 48** (see Procedure)

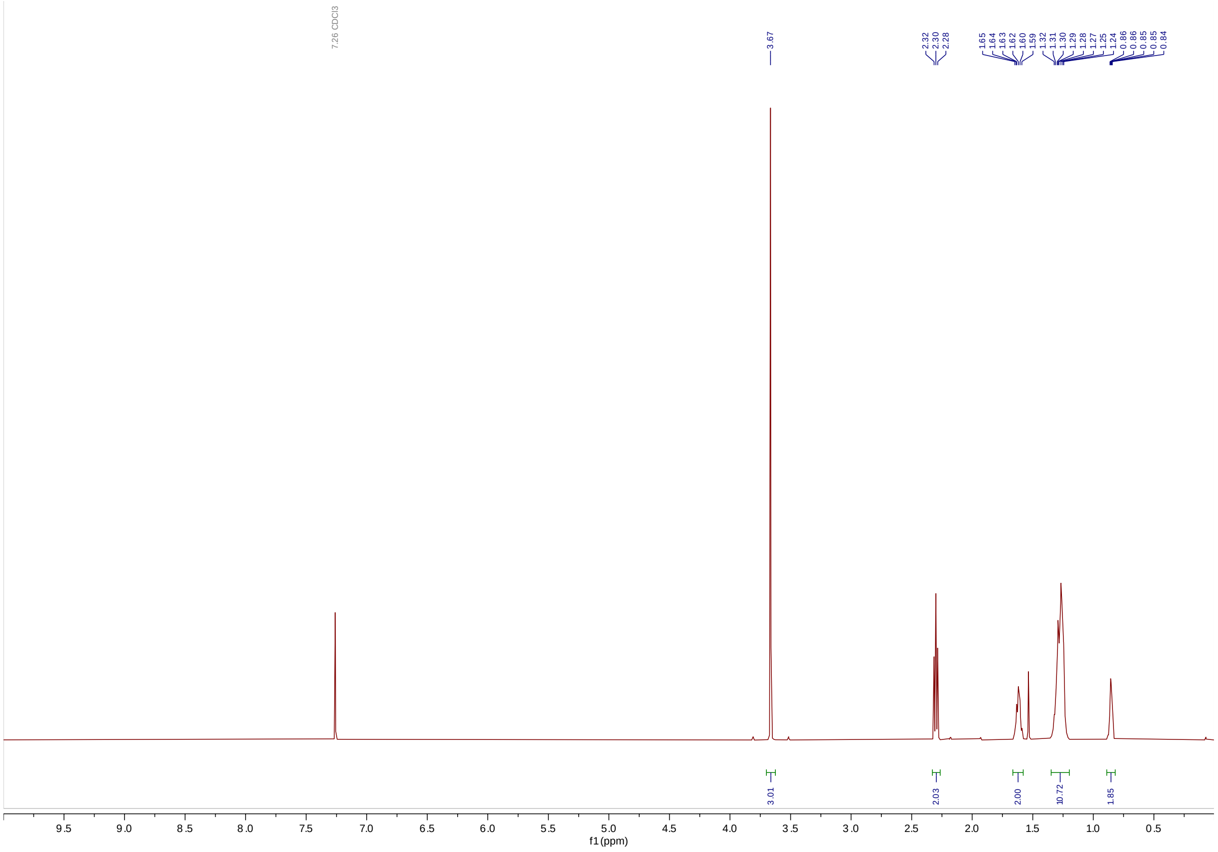

^{13}C-NMR (125.65 MHz, $CDCl_3$) of **Compound 48** (see Procedure)

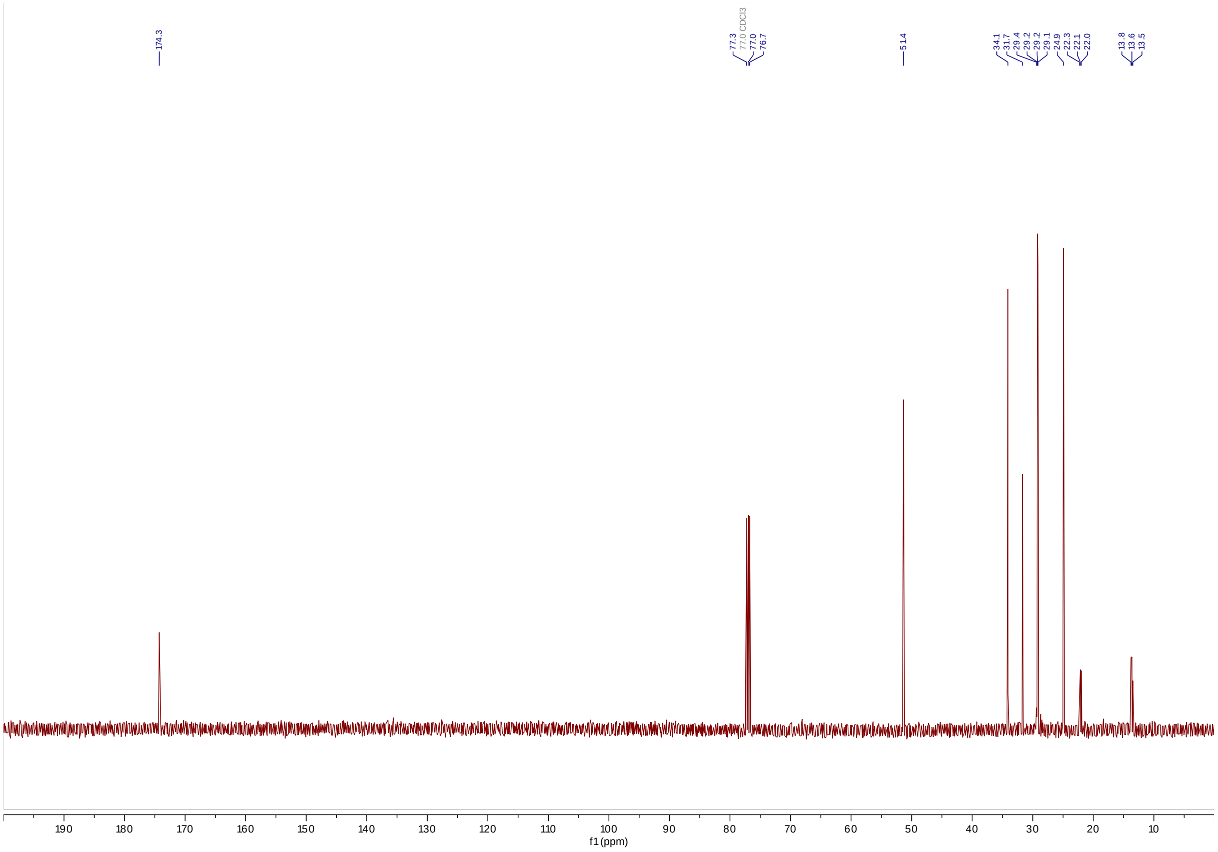

^{1}H-NMR (499.64 MHz, $CDCl_3$): of **Compound 43** (see Procedure)

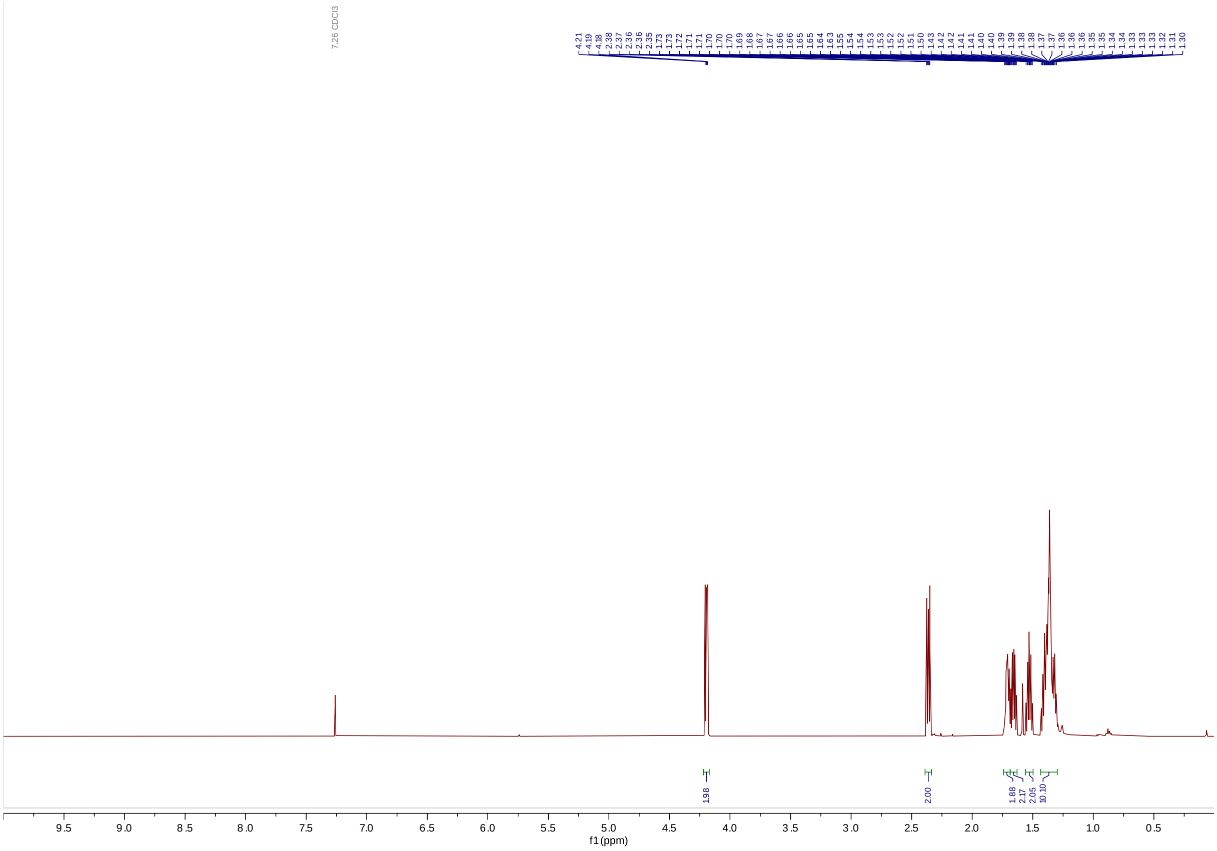

^{13}C-NMR (125.65 MHz, $CDCl_3$) of **Compound 43** (see Procedure)

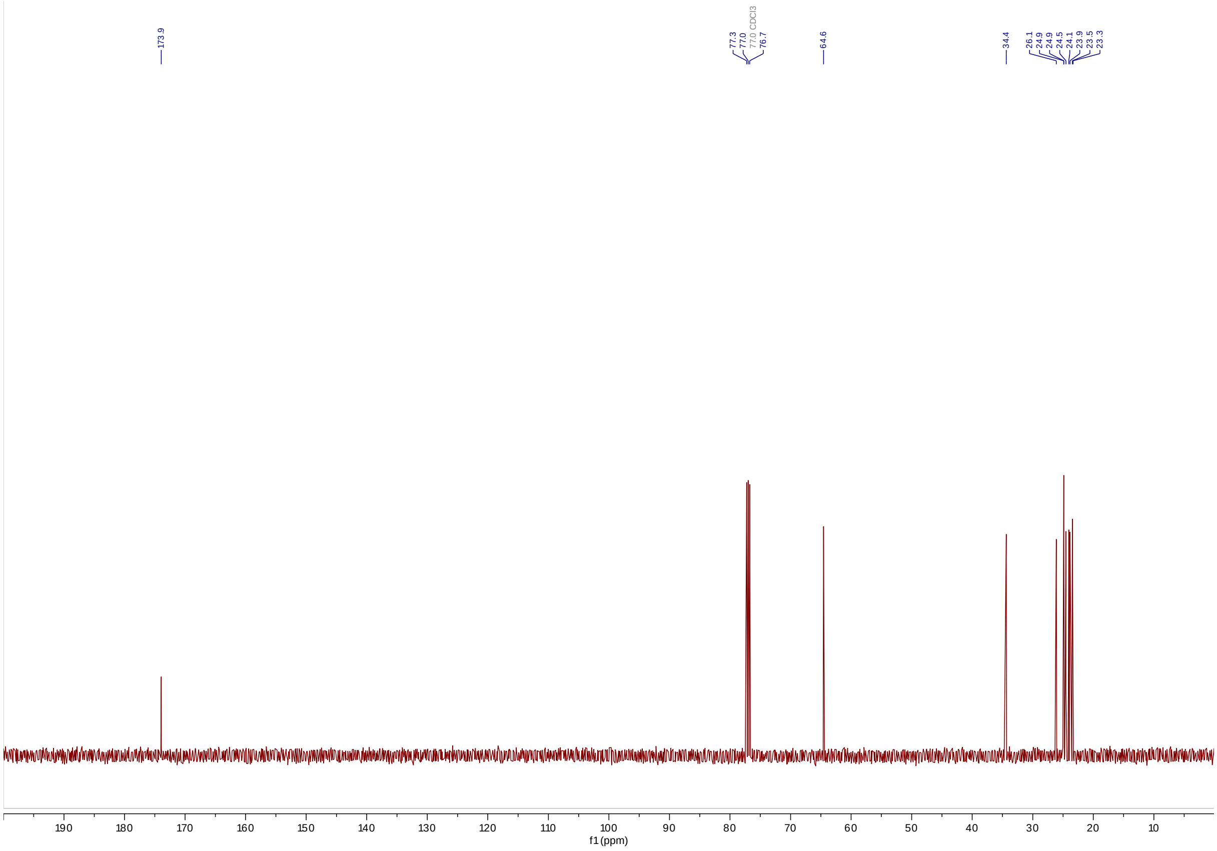

1H-NMR (499.64 MHz, CDCl3): of Compound 44 (see Procedure)

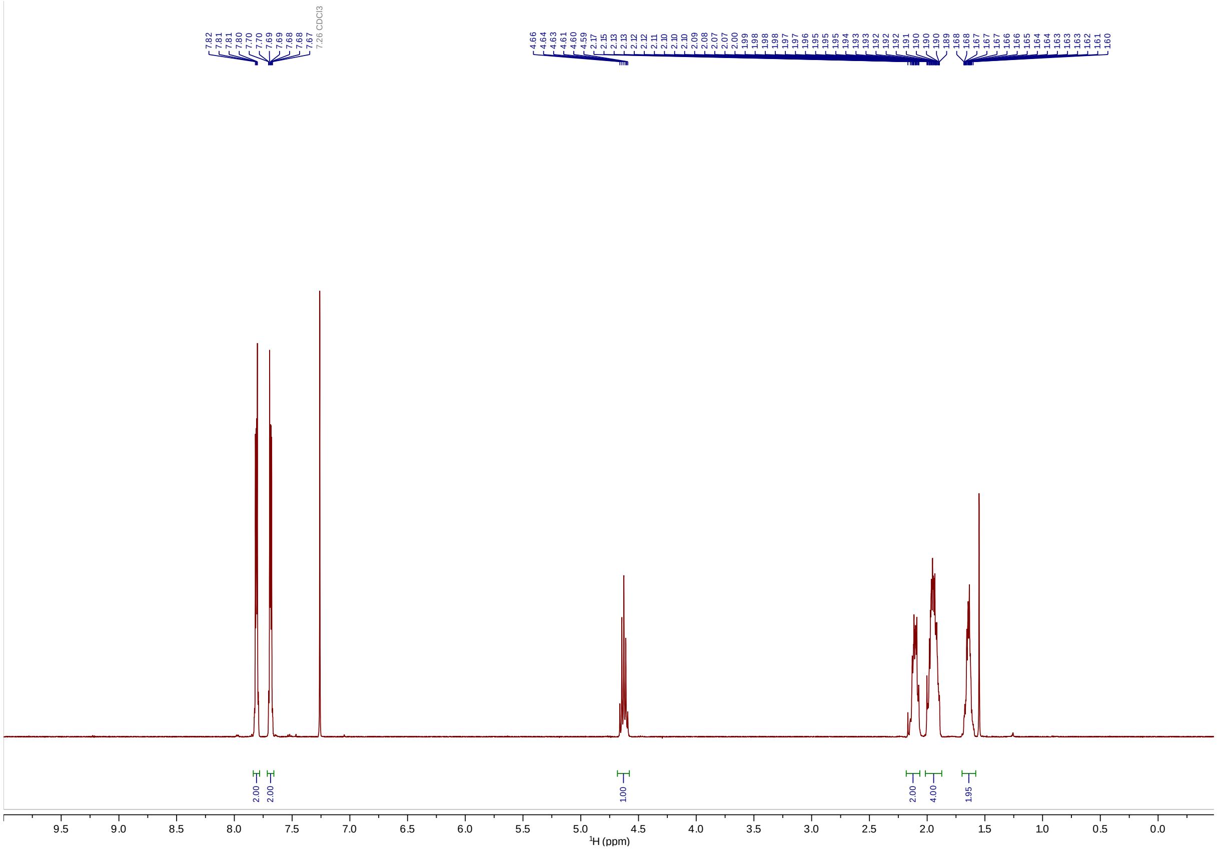

13C-NMR (125.65 MHz, CDCl3) of Compound 44 (see Procedure)

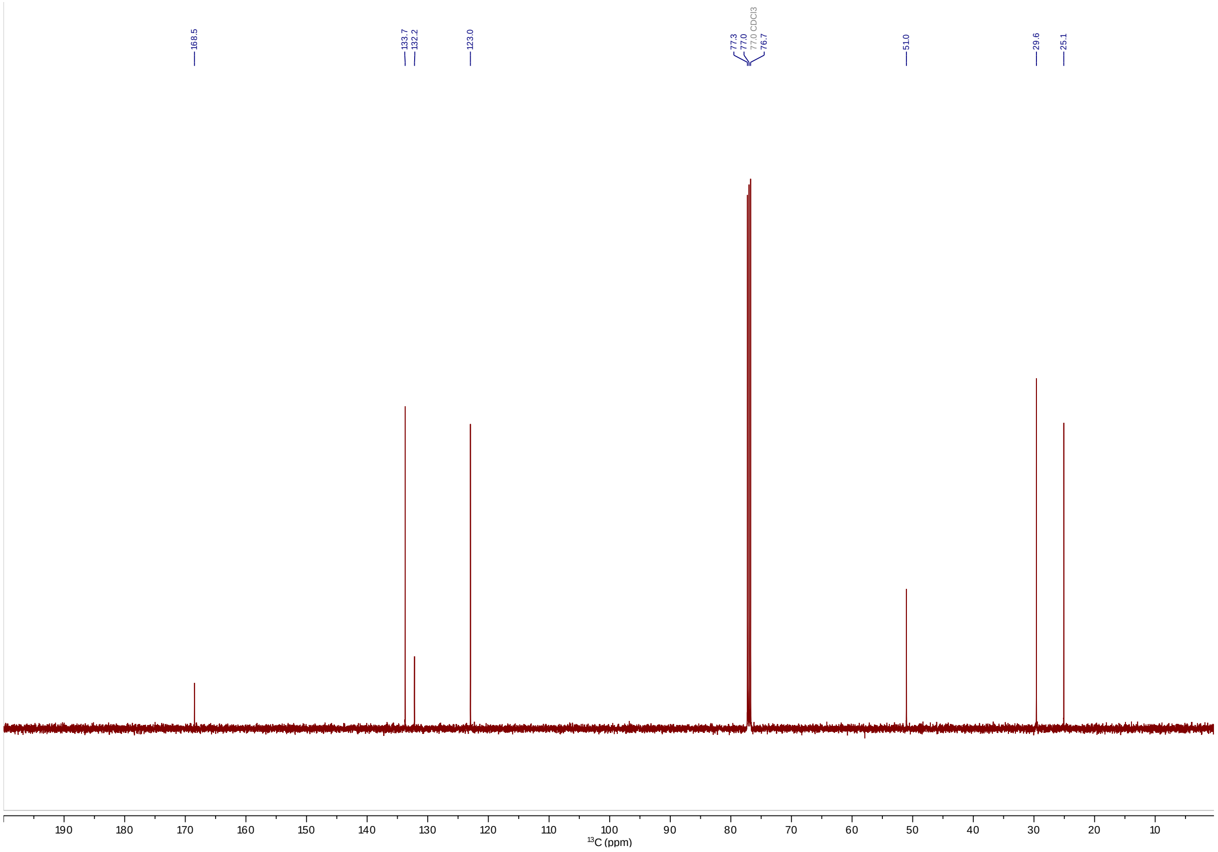

^{1}H-NMR (499.64 MHz, $CDCl_3$): of **Compound 45** (see Procedure)

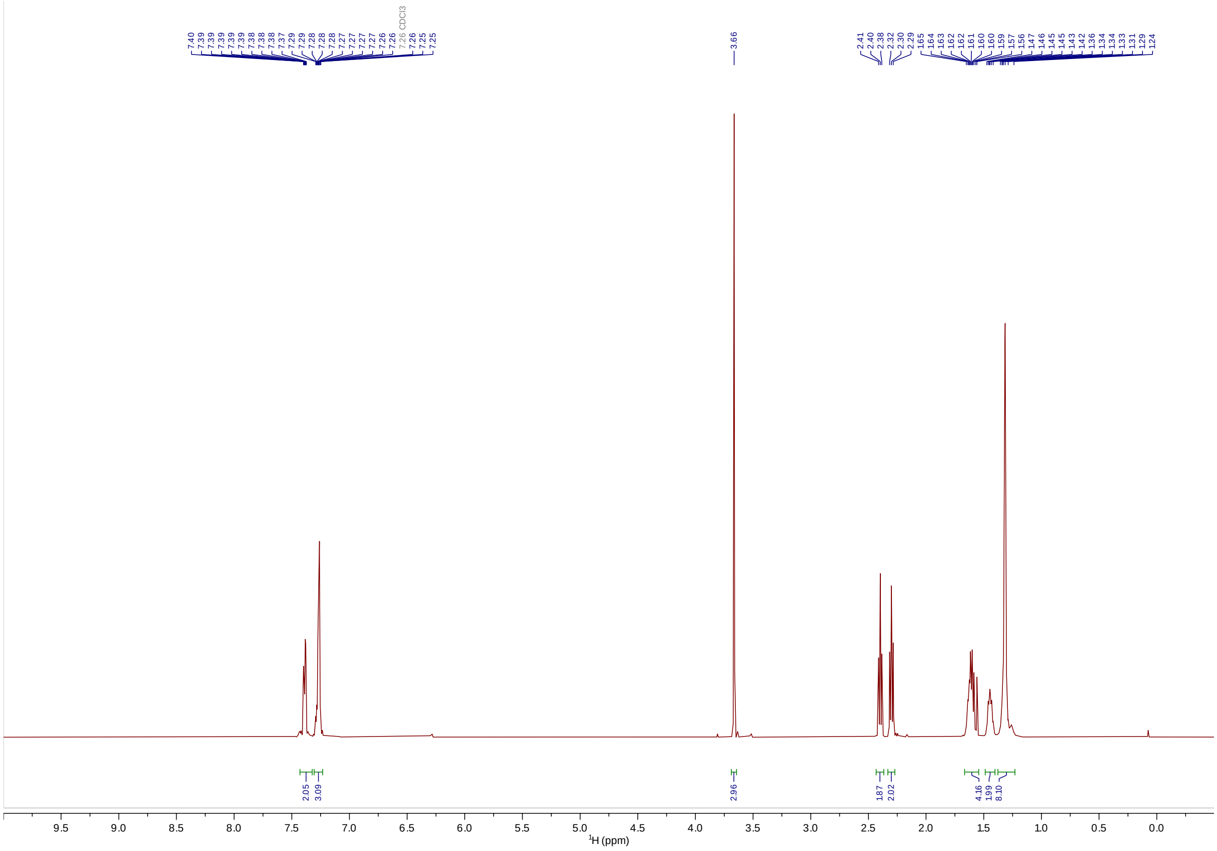

^{13}C-NMR (125.65 MHz, $CDCl_3$) of **Compound 45** (see Procedure)

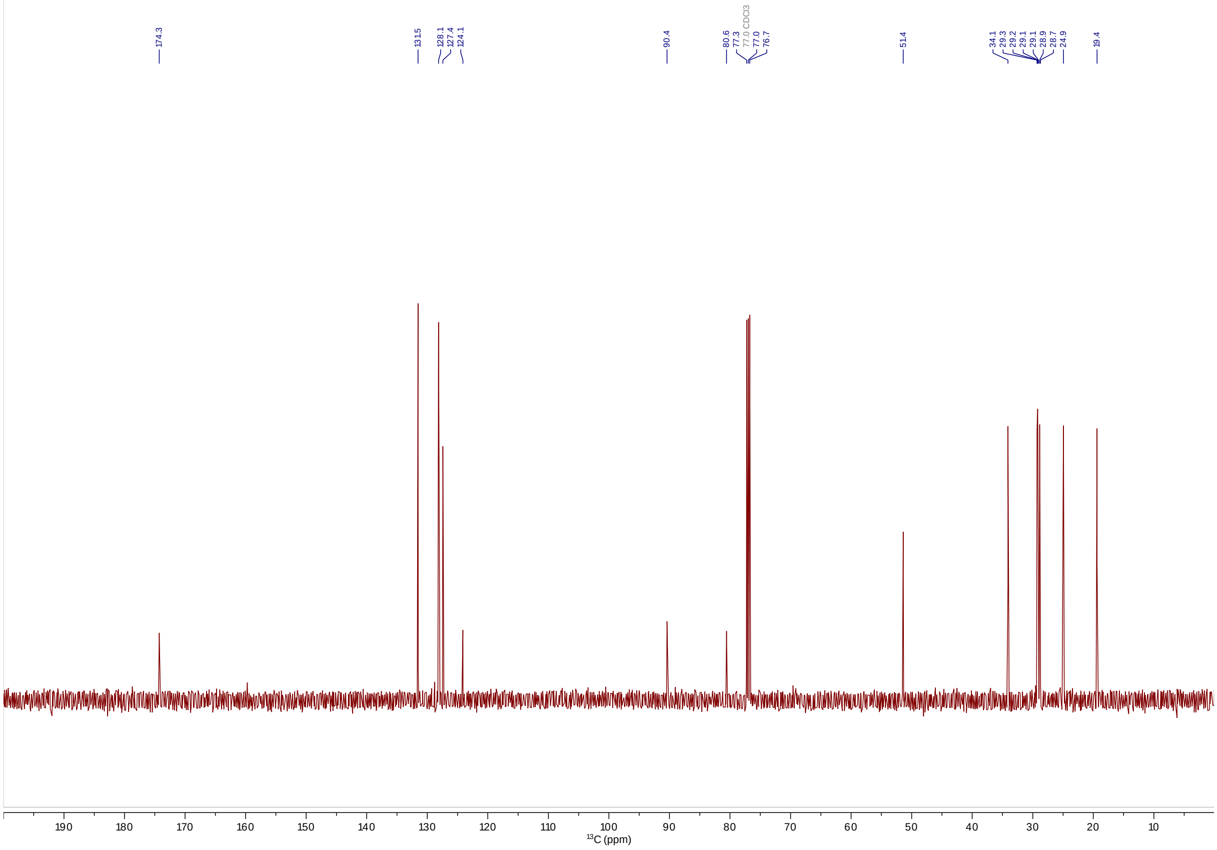

6. References

[43] J. Chen, J. Li, M. B. Plutschack, F. Berger, T. Ritter, *Angew. Chem. Int. Ed*. **2020**, *59*, 5616–5620.

[52] M.-S. Liu, H.-W. Du, W. Shu, *Chem. Sci*. **2022**, *13*, 1003–1008.

[54] P. Angyal, A. M. Kotschy, Á. Dudás, Sz. Varga, T. Soós, *Angew. Chem. Int. Ed*. 2023, *62*, e202214096.

[61] C. Chen, M. Wang, H. Lu, B. Zhao, Z. Shi, *Angew. Chem. Int. Ed*. 2021, *60*, 21756–21760.

[72] X. Li, S. He, Q. Song, *Org. Lett*. 2021, *23*, 2994–2999.

[73] S. W. Lardy, V. A. Schmidt, *J. Am. Chem. Soc.* **2018**, *140*, 12318–12322.

[74] P. P. Lampkin, B. J. Thompson, S. H. Gellman, *Org. Lett*. **2021**, *23*, 5277–5281.
